# Discovery of a Potent Fluorescence Polarization Probe for Identifying USP1 Allosteric Inhibitors

**DOI:** 10.1002/advs.75350

**Published:** 2026-04-22

**Authors:** Jiawei Cheng, Peipei Wang, Pengfei Wang, Jiamin Wang, Baiyang Li, Daqing Fang, Jian He, Xiaobei Hu, Weijuan Kan, Yubo Zhou, Chunpu Li, Jia Li, Hong Liu

**Affiliations:** ^1^ Key Laboratory of Structure‐Based Drug Design and Discovery, Ministry of Education Shenyang Pharmaceutical University Shenyang China; ^2^ State Key Laboratory of Drug Research, Shanghai Institute of Materia Medica Chinese Academy of Sciences Shanghai China; ^3^ School of Chinese Materia Medica Nanjing University of Chinese Medicine Nanjing China; ^4^ School of Pharmaceutical Science and Technology Hangzhou Institute for Advanced Study, UCAS Hangzhou China; ^5^ University of Chinese Academy of Sciences Beijing China; ^6^ Shandong Laboratory of Yantai Drug Discovery Bohai Rim Advanced Research Institute for Drug Discovery Yantai China; ^7^ Air Force Hospital of Western Theater Command Chengdu China; ^8^ Zhongshan Institute for Drug Discovery, Shanghai Institute of Materia Medica Chinese Academy of Sciences Zhongshan China

**Keywords:** Allosteric inhibitor, Anti‐lymphoma, FP‐based assay, THIQ skeleton, USP1

## Abstract

The deubiquitinating enzyme, ubiquitin‐specific protease 1 (USP1), is overexpressed in various tumor types, making it a promising target for cancer therapy. The development of USP1 allosteric inhibitors has progressed rapidly owing to their high selectivity and potency. However, the lack of appropriate chemical tools for developing a binding screen for this site has hampered the discovery of novel ligands. Herein, we developed the first allosteric fluoroprobe and fluorescence polarization (FP) assay for direct validation of USP1 allosteric inhibitors. The FP assay based on allosteric tracer **6‐2** enabled the differentiation of known allosteric and catalytic site inhibitors, providing a robust and scalable tool for high‐throughput screening. In addition, a novel class of potent tetrahydroisoquinoline USP1 inhibitors was identified, and the representative compound **14a** possessed superior enzymatic and cellular activity compared with the clinical candidate **KSQ‐4279,** with potent in vivo anti‐DLBCL efficacy and good druggability. Collectively, this study provides a valuable fluoroprobe, FP assay platform, and lead compounds targeting USP1 for further structural optimization and antitumor mechanism studies.

## Graphical abstract

This study presents the first ubiquitin‐specific protease 1 (USP1) allosteric fluoroprobe and fluorescence polarization assay, enabling the differentiation of allosteric and catalytic site inhibitors. Further, a novel class of tetrahydroisoquinoline‐based USP1 inhibitors is designed, with compound **14a** (USP1 IC_50_ = 29.9 nM) showing strong selectivity and promising antitumor efficacy. Collectively, this study provides a valuable platform and lead compounds for targeting USP1.

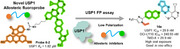

## 1 Introduction

Ubiquitin is covalently attached to the lysine residues of cellular proteins by activating (E1), conjugating (E2), and ligating (E3) enzymes, a process that represents a common regulatory mechanism in eukaryotic cells [1, 2]. Ubiquitination is a reversible process, and the human genome encodes over 90 deubiquitinating enzymes (DUBs) that catalyze the removal of ubiquitin moieties from target proteins [2, 3]. Among these enzymes, ubiquitin‐specific proteases (USPs) constitute an important subfamily of DUBs. The USP family comprises multiple subtypes, including USP1, USP2, and USP12 [4]. Notably, USP1 contains a highly conserved catalytic domain composed of three structural subdomains, featuring an N‐terminal Cys box motif and two C‐terminal His box motifs [5]. The catalytic activity of USP1 is significantly enhanced by USP1‐associated factor 1 (UAF1), and the heterodimeric USP1/UAF1 complex plays a critical role in deubiquitination [6]. Importantly, USP1 has been extensively characterized as an attractive antitumor target due to its involvement in DNA damage response (DDR) pathways [7]. In 2013, Rodríguez et al. reported that USP1 inhibition could reverse cisplatin resistance in an in vitro non‐small cell lung cancer model [8]. Subsequently, Dandrea et al. (2018) demonstrated that USP1 is overexpressed in breast cancer, with the overexpression correlating with BRCA1 gene deletion [9]. More recently, Telegeev et al. (2023) reported that USP1 expression reaches its highest levels in cervical squamous cell carcinoma and endocervical adenocarcinoma, and is significantly elevated compared to normal tissues in several other malignancies, including breast invasive carcinoma, sarcoma, and cholangiocarcinoma [10]. In addition, Wang et al. observed aberrant USP1 expression in specimens of patients with diffuse large B‐cell lymphoma (DLBCL) and cell lines, and found that USP1 overexpression was associated with poor prognosis [11]. Mechanistically, USP1 stabilizes the MAX/MYC protein complex by deubiquitinating MYC, thereby prolonging its half‐life (Figure S1) [11]. As a transcription factor, MYC regulates the expression of numerous genes involved in cellular metabolism, proliferation, apoptosis, and differentiation, and plays a pivotal oncogenic role in lymphoma [12]. Collectively, these findings indicate that USP1 expression is dysregulated in multiple cancer types, suggesting that USP1 may be a promising therapeutic target for cancer treatment.

**FIGURE 1:**
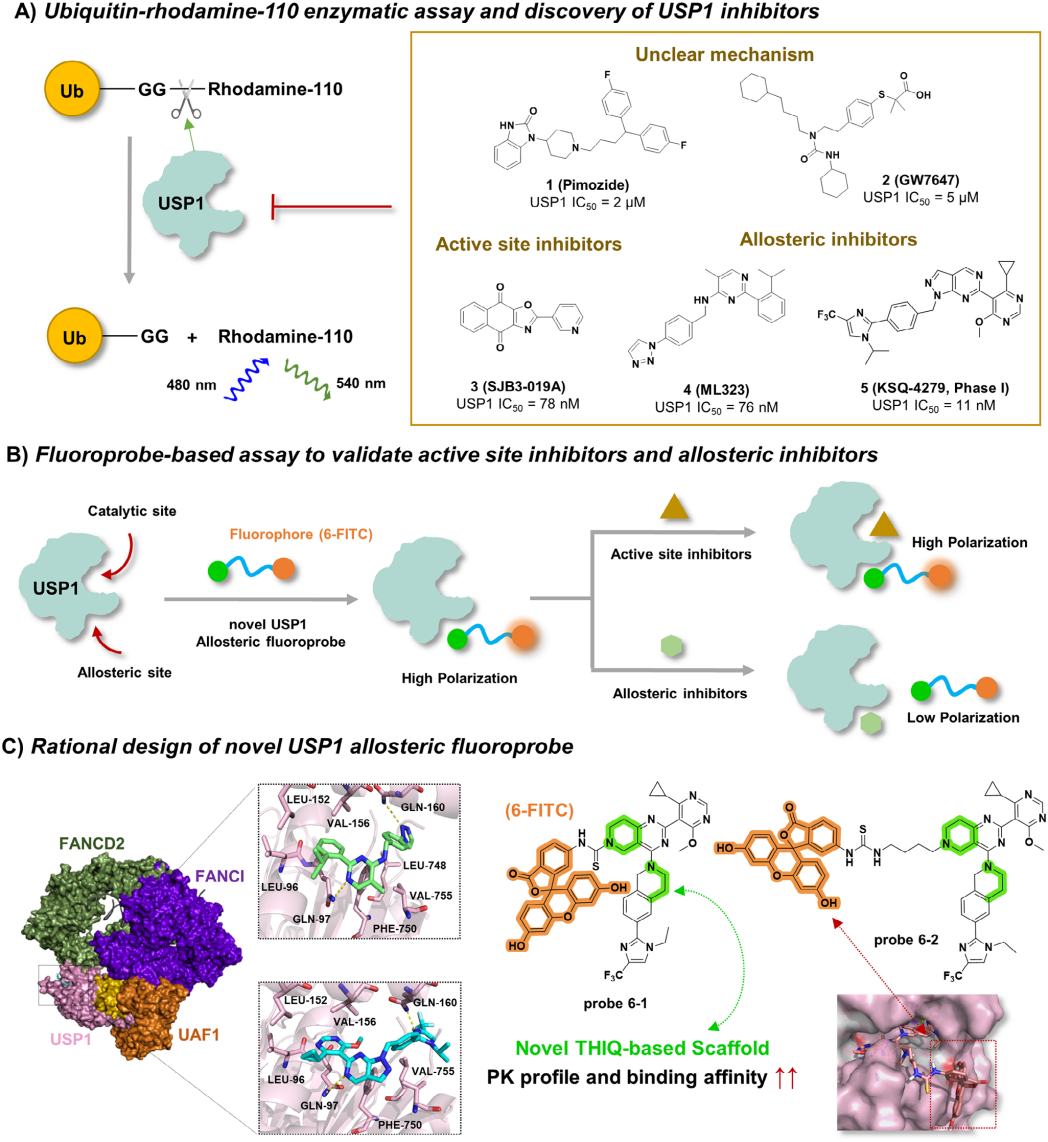
Development of a novel fluorescence polarization assay for USP1 allosteric inhibitor discovery. (A) Ubiquitin‐rhodamine‐110 HTS and representative USP1 inhibitors. (B) USP1 FP assay design principle. (C) Rational design of a novel USP1 allosteric fluoroprobe. **4** (green) or **5** (cyan) in complex with USP1 (PDB ID 8A9K, 9FCI).

Various USP1 inhibitors have been identified through high‐throughput screening (HTS) followed by structural optimization, including **pimozide**, **ML323**, and **KSQ‐4279** [13]. The ubiquitin‐rhodamine‐110 (Ub‐Rho) fluorometric assay has proven to be an effective HTS platform for hit compound identification because of the favorable fluorescence properties of the substrate, achieving considerable success in discovering USP1 inhibitors (Figure 1A). As this screening platform relies on the substrate‐cleaving activity of DUBs, it can detect small molecules with diverse mechanisms of action (MOA), including both allosteric and catalytic site inhibitors. However, this underscores the necessity for more in‐depth studies to fully elucidate the MOA of the identified compounds. Historically, Ub‐Rho‐based HTS has led to the identification of numerous small‐molecule ligands with varying MOA, some of which are pan‐assay interference compounds or otherwise non‐specific. In 2011, Zhuang et al. conducted a Ub‐Rho‐based HTS and identified two reversible inhibitors, **pimozide** (**1**, IC_50_ = 2 µM) and **GW7647** (**2**, IC_50_ = 5 µM) [14]. Although both compounds effectively inhibited the USP1‐UAF1 complex, their poor selectivity and off‐target activities severely limited their further development. In 2013, D'Andrea et al. identified **SJB3‐019A** (**3**) as a sub‐micromolar USP1 inhibitor using a Ub‐Rho USP1/UAF1 assay with DTT as a reducing agent. However, **SJB3‐019A** is a redox cycling compound (RCC) that may inhibit USP1 and other DUBs by oxidizing the catalytic cysteine, which is not an attractive mechanism for DUB inhibition [15]. In 2014, Maloney et al. reported **ML323** (**4**) and related analogs exhibiting potent nanomolar inhibition of USP1‐UAF1 activity (IC_50_ = 76 nM) with minimal activity against 18 other DUBs, 70 unrelated proteases, and 451 kinases [16]. In subsequent research, compound **4** was confirmed to be an allosteric inhibitor following cryo‐electron microscopy (cryo‐EM), marking the beginning of the discovery of novel USP1 inhibitors. A series of clinical candidates, including **KSQ‐4279** (**5**), was derived based on compound **4**. Notably, **5** has progressed to Phase I clinical trials, demonstrating an acceptable safety profile, either as a single agent or in combination with therapy. Although the Ub‐Rho HTS system has made significant contributions to the discovery of USP1 inhibitors, it is unable to discriminate between active‐site and allosteric inhibitors, which may lead to the identification of undesired non‐selective inhibitors, such as RCCs. Therefore, a more direct, precise, and applicable binding assay is urgently needed to characterize novel USP1 allosteric inhibitors with higher clinical value.

Fluorescence polarization (FP) assays have emerged as powerful tools for measuring the binding affinities of small‐molecule ligands [17, 18, 19, 20, 21]. The advantages of FP assays, including high speed, operational simplicity, accuracy, and cost‐effectiveness, make them particularly suitable for HTS of small molecules. Small‐molecule fluorescent probes have emerged as powerful tools for advancing cell biology and drug discovery. Despite the intense efforts in USP1 inhibitor discovery, the development of a practical USP1 FP probe has yet to be achieved, which has severely hampered precise screening and drug development of USP1 inhibitors. The design concept of the FP probe is illustrated in Figure 1B. This competitive FP assay enables the screening of USP1 allosteric inhibitors with well‐defined MOA. In the FP assay, an unbound FP probe rotates freely, resulting in low polarization. Upon binding to the allosteric site of USP1, the rotational mobility of the probe is restricted, leading to an increase in polarization. When the test compound competes for the same binding pocket and displaces the probe, the polarization signal decreases. Herein, we designed novel USP1 fluorescence probes (**6‐1** and **6‐2**) based on the potent USP1 allosteric inhibitors **4** and **5**. Using these probes, we established a rapid, cost‐effective, and reliable FP assay to quantify the binding affinities of allosteric inhibitors to USP1, enabling a clear differentiation between known allosteric and catalytic‐site inhibitors. The HTS platform based on the probes allows for the rapid and precise identification of USP1 allosteric inhibitors. Furthermore, a series of novel USP1 inhibitors containing a tetrahydroisoquinoline (THIQ) scaffold was developed, resulting in the discovery of lead compound **14a**, which exhibited strong USP1 inhibitory activity (IC_50_ = 29.9 ± 1.5 nM), ideal selectivity, favorable pharmacokinetics (PKs), and good pharmacological efficacy in a DLBCL xenograft mouse model.

## 2 Results and Discussion

USP1 FP probes were designed using structure‐based drug design (SBDD). Recently, Walden et al. published the cocrystal structures of compounds **4** (PDB: 8A9K) and **5** (PDB: 9FCI) bound to USP1 using cryo‐EM [22, 23]. Cryo‐EM analysis revealed that both inhibitors bound to a cryptic pocket, occupying the hydrophobic tunnel and inducing structural disruption, thereby allosterically inhibiting USP1‐UAF1 activity [23]. For the rational design of USP1 FP probes, we analyzed the binding modes of compounds **4** and **5** (Figure 1C). Structural analysis revealed that both ligands maintained identical hydrogen‐bonding patterns; the pyrimidine *N*_1–_GLN97 and azole *N*_2–_GLN160 interactions were fully conserved. Additionally, the 4‐cyclopropyl‐6‐methoxypyrimidin‐5‐yl group of compound **5** formed hydrophobic interactions within a tunnel‐like pocket, occupying a position similar to that of the 2‐propan‐2‐ylphenyl group in compound **4**. Occupancy of the hydrophobic tunnel resulted in a significant increase in the thermal stability of USP1 [23]. Overall, these interactions are crucial for maintaining high activity levels. However, the methyl group in the pyrimidine scaffold of compound **4** did not interact with USP1 and was oriented toward the solvent site. Although compound **4** exhibits potency and selectivity as an allosteric USP1 inhibitor, it suffers from a critical drawback: the formation of *N*‐hydroxylamine via *N*‐oxidation of the secondary amine, which is a key intermediate implicated in toxicity and carcinogenesis. This presents a significant barrier to lead compound discovery. Furthermore, compound **4** has a short half‐life of 15 min in mouse liver microsomes (MLM). The THIQ framework, both naturally occurring and synthesized, has demonstrated diverse biological activities, remarkable cytotoxicity, and potency against human cancer cell lines [24]. To address these limitations, we introduced a THIQ skeleton (Figure 1C) that directs metabolism toward detoxification via N‐oxidation, yielding polar tertiary N‐oxides with reduced toxicity and improved excretion. Furthermore, the THIQ constraint led to additional benefits, including increased rigidity, enhanced metabolic stability, and potentially improved activity. For fluorophore conjugation, the methyl group was replaced with a piperidine ring in the solvent‐exposed region (Figure 1C). Fluorescein isothiocyanate (FITC), a fluorophore widely used in FP probes, was used for labeling [25, 26]. We hypothesized that the fragment conjugated via a flexible linker would retain binding affinity. Accordingly, based on the privileged motifs of compounds **4** and **5**, the fluorescent probes **6‐1** and **6‐2** with varying linker lengths were designed and synthesized to enable customizable FP‐based in vitro HTS of USP1 allosteric inhibitors.

**FIGURE 2:**
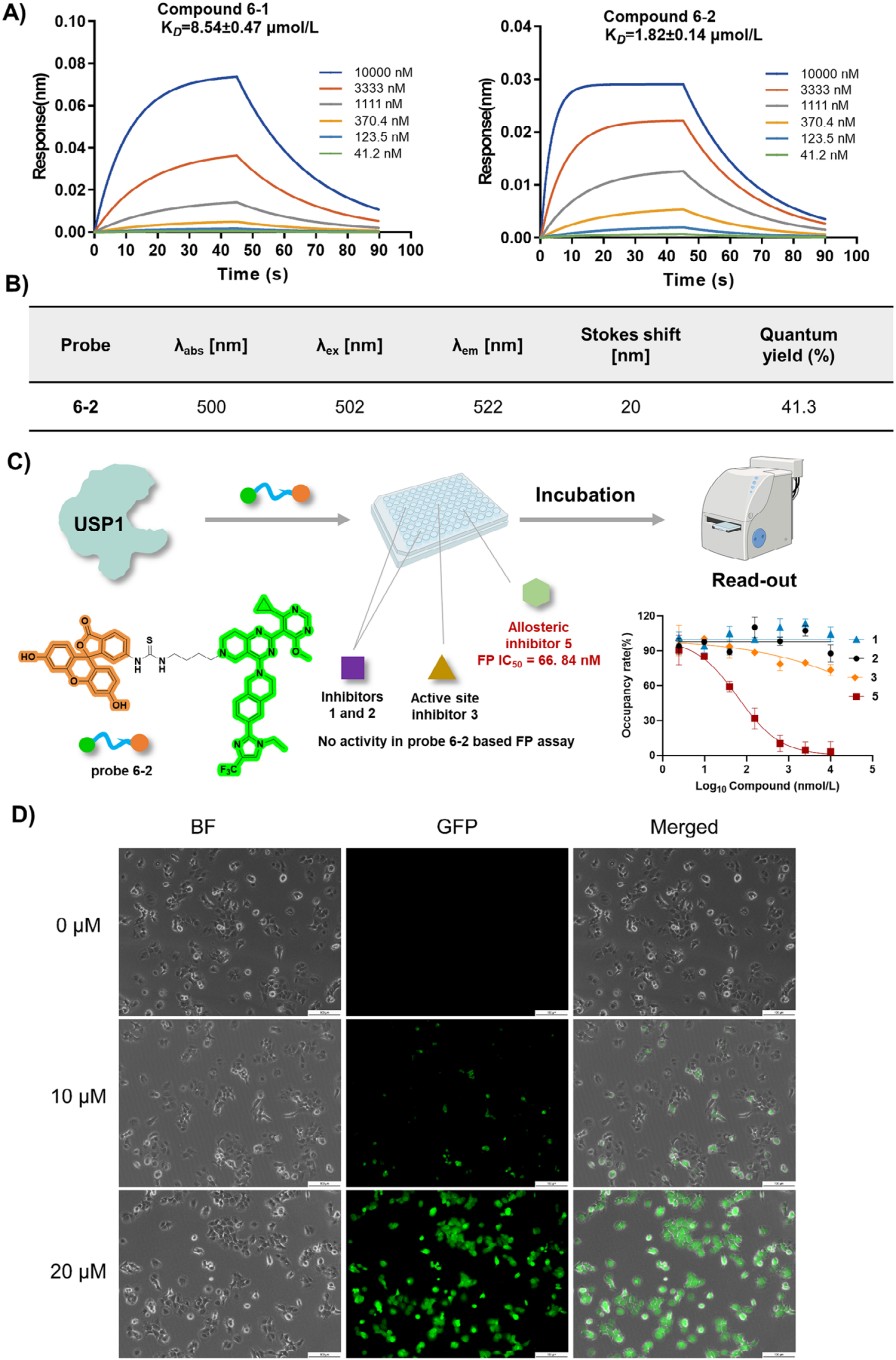
The novel FP assay was validated using probe **6‐2**. (A) Binding affinity of probes with USP1. (B) Absorption and fluorescence properties of probe **6‐2** in PBS. (C) Validation of the USP1 FP assay using probe **6‐2**. (D) Application of **6‐2** as a tool to visualize cellular target engagement via fluorescence microscopy with fixed CAOV‐3 cells.

Using these two probes, a bio‐layer interferometry (BLI) binding assay was performed to quantitatively assess their binding affinities for USP1 (Figure 2A). Dose‐dependent interactions were observed for both probes. Analysis revealed that probe **6‐2** exhibited higher affinity, with an equilibrium dissociation constant (*K*_d_) of 1.82 µM, compared to 8.54 µM for probe **6‐1**. Therefore, probe **6‐2** was selected for further characterization of its absorption and fluorescence properties. The absorption and fluorescence spectra of probe **6‐2** were measured (Figure S2), revealing maximum absorption at 500 nm, an excitation wavelength of 502 nm, and an emission wavelength of 522 nm, corresponding to a Stokes shift of 20 nm. In addition, probe **6‐2** exhibited a high fluorescence quantum yield (Φ = 41.3%) in PBS (Figure 2B), indicating its suitability for subsequent in vitro biological applications. To assess whether the FP assay can accurately reflect in vitro screening outcomes for allosteric USP1 inhibitors, we first determined the enzymatic inhibitory activity of the known allosteric inhibitor **5** using a classical Ub–Rho 110 fluorometric assay (IC_50_ = 39.74 ± 9.6 nM). Next, we evaluated the binding occupancy of inhibitor **5** and that of non‐allosteric inhibitors **1–3** using an FP assay with probe **6‐2**. Encouragingly, inhibitor **5** exhibited an IC_50_ of 66.84 ± 17.03 nM in the FP assay, whereas the non‐allosteric inhibitors **1–3** failed to compete with probe **6‐2** in the competitive binding assay. These findings support the reliability of the FP assay for identifying compounds that target the allosteric site of USP1 (Figure 2C). Furthermore, we investigated the potential of probe **6‐2** as a fluorescent tracer to visualize cellular target engagement. No detectable cytotoxicity was observed in CAOV‐3 cells at concentrations up to 4 µM (Figure S3A). Using fluorescence microscopy of fixed CAOV‐3 cells with high USP1 expression, the fluorescence pattern of probe **6‐2** clearly demonstrated specific binding to USP1 (Figure 2D). To further enhance membrane permeability, the fluorescent moiety was switched from FITC to tetramethylrhodamine (TRITC), and probe **6‐3** was developed. Similarly, the probe **6‐3** specifically bound to USP1 in live CAOV‐3 cells (Figure S4). Collectively, these results indicate that FP probe **6‐2** is well‐suited for characterizing USP1 inhibitors targeting the allosteric pocket.

**FIGURE 3:**
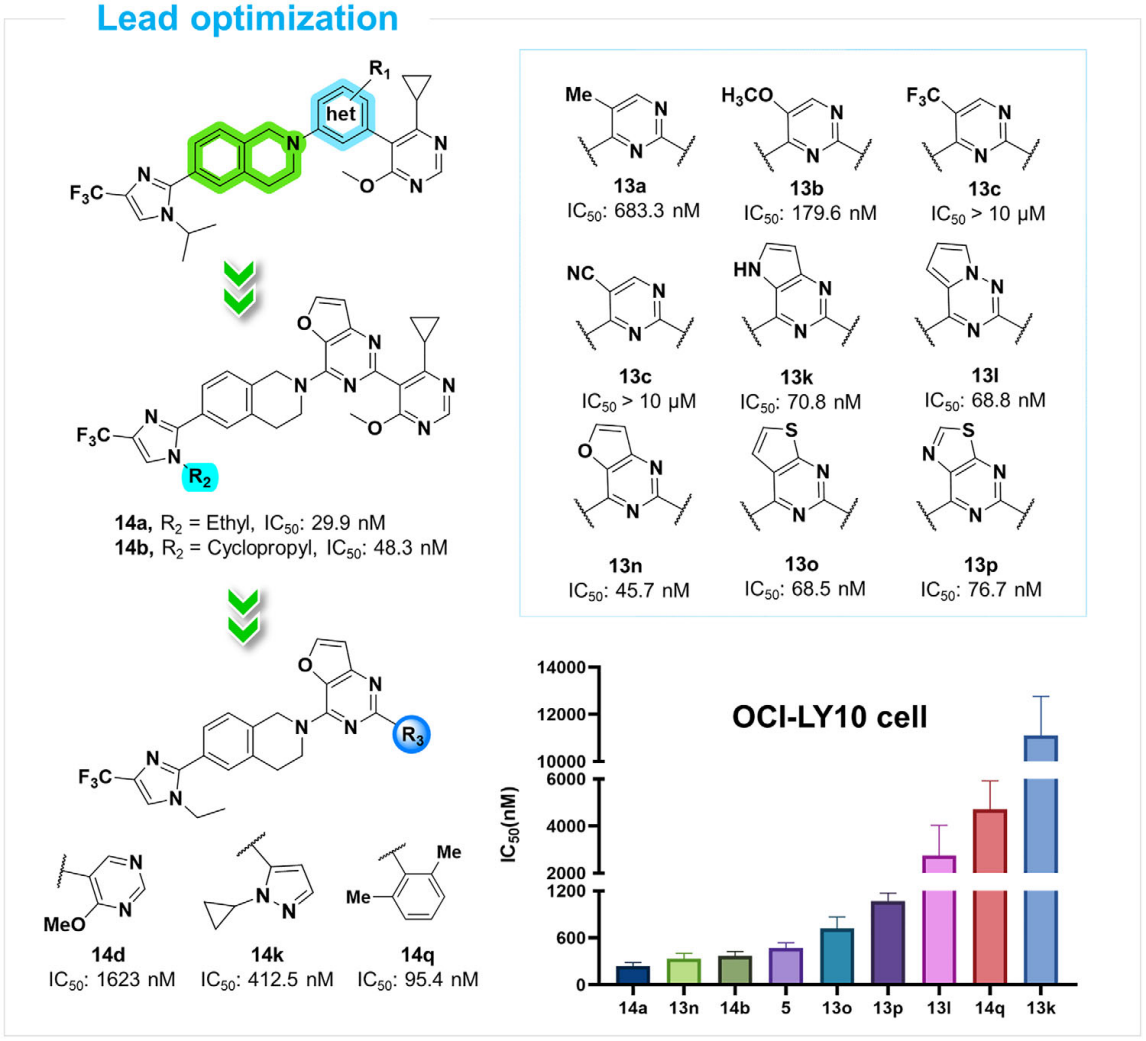
Evolution and SARs of THIQ‐based USP1 inhibitors.

Following probe design and validation, we initiated a medicinal chemistry campaign to explore structure–activity relationships (SARs) and identify novel therapeutic agents targeting USP1 (Figure 3). Forty compounds were synthesized, and comprehensive SAR analysis results are available in the Supporting Information (Tables S1 and S2). First, we explored the R_1_ substituents with diverse electron properties. Among them, compounds with methoxy (**13b**) substitution at R_1_ exhibited good inhibitory activity, indicating that the donation of electrons from the oxygen atom may enhance compound potency. Conversely, the electron‐withdrawing trifluoromethyl (**13c**) and cyano (**13d**) groups induced a loss of inhibitory activity, probably because of their strong electronegativity. The results demonstrate that electron‐donating groups enhanced activity and that the THIQ scaffold contributed to sustained potency. Considering the 5‐methylpyrimidine of **4**, which may afford low inhibitory activity compared with **5**, we initiated our optimization by replacing 5‐methylpyrimidine with fused rings to prepare **13e–13p** with aliphatic and aromatic rings as the core moiety. Most compounds with electron‐rich fused rings exhibited inhibitory activities superior to **4**, demonstrating the success of our drug design strategy. Notably, compound **13n** containing a furo[*3,2‐d*]pyrimidine moiety (IC_50_ = 45.7 ± 1.9 nM) was the most potent compound in the series **13a–13p**, demonstrating greater than fivefold USP1 inhibitory activity over **4**, comparable to **5**. These results supported the premise of 4‐(3,4‐dihydroisoquinolin‐2(1*H*)‐yl)furo[*3,2‐d*]pyrimidine moiety as a favorable framework for further optimization. Next, we fixed the furo[*3,2‐d*]pyrimidine moiety and explored R_2_ to provide compounds **14a** and **14b**. Unexpectedly, the enzyme activity results showed that the substitution of the ethyl group (**14a**, IC_50_ = 29.9 ± 1.5 nM) in R_2_ was better than that of the isopropyl group (**13n**), cyclopropyl group (**14b**), and the positive control **5**, thus confirming that the steric hindrance effect of bulky groups was detrimental to inhibitory activity. After exploring the modifications of the R_2_ groups, we synthesized compounds **14c–14y** to further optimize the hydrophobic fragment (R_3_). The introduction of *ortho/para* substituents or unsubstituted analogs led to a marked decrease in potency. However, compound **14q** with 4,6‐dimethylbenzene significantly restored the inhibitory activity, suggesting that the steric effects conferred by the *ortho*‐disubstituted architecture may be key determinants in maintaining potency. The nine most promising compounds exhibiting good USP1 enzymatic activity (IC_50_ < 100 nM) were selected for further evaluation of their cellular antiproliferative activity. All of the tested compounds showed antiproliferative activities against the DLBCL cell line OCI‐LY10, while compounds **13n** (IC_50_ = 334.27 ± 67.49 nM), **14a** (IC_50_ = 240.93 ± 43.35 nM), and **14b** (IC_50_ = 369.87 ± 56.82 nM) showed potent antiproliferative activity, indicating that the furo[*3,2‐d*]pyrimidine framework was vital for this activity (Figure 3). Notably, **14a** showed better antiproliferative activity than the positive control **5** (IC_50_ = 469.87 ± 66.37 nM).

**FIGURE 4:**
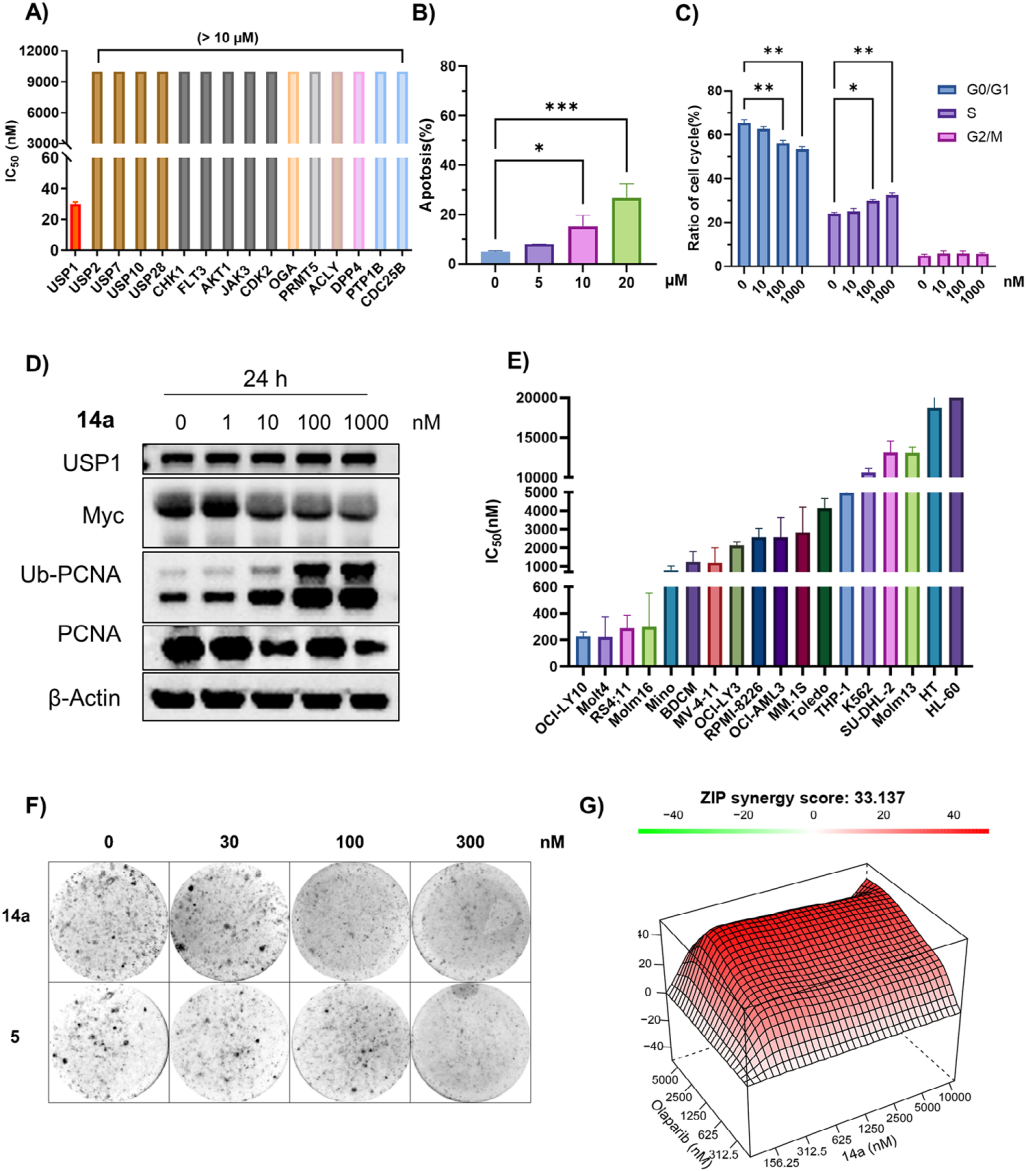
**14a** exerts an anti‐DLBCL effect by targeting USP1 at the cellular level. (A) The effect of **14a** on the inhibition of various enzymes. (B) Quantification of apoptosis in OCI‐LY10 cells induced by compound **14a** treatment for 72 h. (C) Quantification of OCI‐LY10 cell cycle arrest induced by compound **14a** treatment for 72 h. (D) Quantification of USP1, MYC, Ub‐PCNA, and PCNA expression levels in OCI‐LY10 cells after 24 h of treatment with compound **14a**. (E) Antiproliferative activity of **14a** against a panel of blood cancer cell lines. (F) Colony formation assay of MDA‐MB‐436 cells after treatment with **14a**. (G) SynergyFinder 3.0 was used to assess the combined effects of olaparib and **14a** on CAOV‐3 cells. Statistical significance is denoted by ^*^ *p* < 0.05, ^**^ *p* < 0.01, and ^***^ *p* < 0.001 using the unpaired Student's *t*‐test. The error bars represent the standard deviation (SD) of n = 3.

USP1 has been implicated in promoting tumor progression in DLBCL [11]. Based on the promising potential of compound **14a** as an anticancer agent, further studies were performed. To this end, we conducted a selectivity study of compound **14a** against the USP class of deubiquitinases to investigate target selectivity. Compound **14a** showed negligible inhibition of structurally related deubiquitinases (including USP2, USP7, USP10, and USP28) (Figure 4A). In addition, we tested the effects of **14a** on a variety of enzymes, including kinases, phospholipases, proteases, and methyltransferases. The results showed that **14a** exhibited no inhibitory activity against these enzymes at a concentration of 10 µM, suggesting that **14a** selectively inhibited USP1 (Figure 4A). To further elucidate the anticancer effects of **14a**, we treated OCI‐LY10 cells with increasing concentrations of **14a**, which resulted in a notable induction of apoptosis (Figure 4B; Figure S3B). Subsequently, dose‐dependent treatment with **14a** for 72 h induced S phase accumulation in OCI‐LY10 cells, which may have contributed to their death (Figure 4C; Figure S3C). To evaluate the cellular consequences of USP1 inhibition, we treated OCI‐LY10 cells with compound **14a** at concentrations of 1, 10, 100, or 1000 nM for 24 h, and found that **14a** treatment dose‐dependently reduced the oncogenic protein levels of MYC in OCI‐LY10 cells (Figure 4D). Furthermore, we monitored the levels of Ub‐PCNA and observed a dose‐dependent increase in Ub‐PCNA levels following **14a** treatment (Figure 4D). These results show that compound **14a** exerts a significant anti‐DLBCL effect by targeting USP1 at the cellular level. We profiled compound **14a** in a panel of blood cancer cell lines for its antitumor efficacy. In addition, we profiled it in normal cells for toxicity. The findings of these experiments showed that compound **14a** has antiproliferative activity against multiple lymphocytic cell lines and negligible activity against normal cells. (Figure 4E; Table S3). Moreover, compound **14a** showed strong potency against OCI‐LY10 (IC_50_ = 240.93 ± 43.35 nM) and TMD‐8 (IC_50_ = 29.9 ± 8.8 nM) cells (Figure 4E; Figure S3D). Herein, we report, for the first time, that a specific USP1 inhibitor (**14a**) exhibits potent antiproliferative activity against DLBCL cell lines. In addition to the tested blood cancer cell lines, studies were conducted using the classic USP1‐overexpressing MDA‐MB‐436 breast cancer cell line and the ovarian cancer CAOV‐3 cell line to evaluate the in vitro antitumor activity of **14a**. In the clonogenic assay using MDA‐MB‐436 cells, treatment with **14a** alone potently suppressed colony formation, reaching near elimination at a concentration of 300 nM, which was comparable to **5** (Figure 4F). Notably, the combination of **14a** and olaparib resulted in a greater synergistic inhibition of MDA‐MB‐436 cell proliferation than the **5**/olaparib combination at various concentrations (Figure S3E). The heat maps depicting the Bliss synergy scores demonstrated that the combination of **14a** and olaparib potently inhibited the proliferation of CAOV‐3 cells, with an IC_50_ value of 149.50 nM (Figure S3F) and a high synergy score of 33.14, comparable to **5** (Figure 4G). In summary, compound **14a** not only exhibited comparable potency to **5** in USP1‐overexpressing cell lines but also presented a promising novel strategy for DLBCL via selective USP1 inhibition. These findings underscore the potential of **14a** as a promising candidate for in vitro and in vivo studies.

**FIGURE 5:**
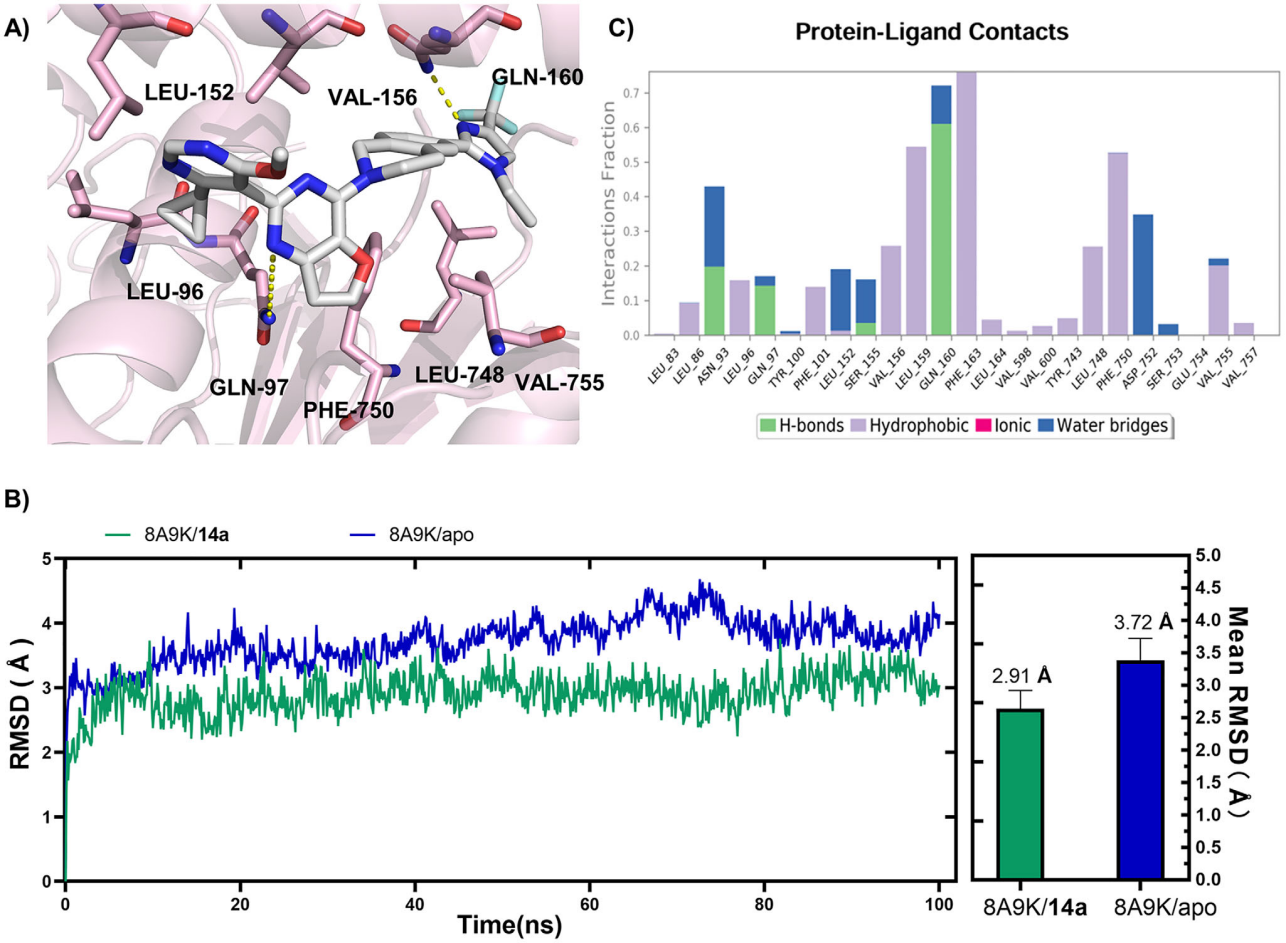
Molecular simulation of compound **14a** with the USP1 protein (PDB code: 8A9K). (A) Putative binding modes of compound **14a**. (B) Normalized stacked bar chart representation of interactions and contacts over the course of the molecular dynamics trajectories for the protein complex and **14a**. (C) RMSD curves of **14a** and apo are in green and blue, respectively.

To gain insight into the molecular basis of the observed inhibitory activity, we performed molecular docking of compound **14a** and the USP1 protein (PDB code: 8A9K). The docking program Glide in the Schrödinger Suite molecular modeling software package was used for docking. Initially, the docked conformation of compound **14a** formed similar interactions with conserved amino acids (Figure 5A). Consistent with the hydrogen‐bonding patterns of **4** and **5**, the nitrogen atoms of the pyrimidine and pyrazole motifs formed hydrogen bonds with GLN97 and GLN160, respectively. Additionally, the 4‐cyclopropyl‐6‐methoxypyrimidin motif occupied a hydrophobic tunnel‐like pocket formed by residues LEU96, LEU152, and VAL156. Meanwhile, the rigid THIQ skeleton engaged in extensive hydrophobic contact with the surrounding residues, VAL156, LEU748, PHE750, and VAL755. The binding mode also provided a rationale for the improved potency of **14a**. Molecular dynamics (MD) simulations on a 100 ns timescale achieved equilibrium as indicated by the root‐mean‐square deviations (RMSDs) of the α‐carbon (Cα) atoms in the complex, which verified the stability of the system. The interaction fraction of the complex was also monitored as shown in Figure 5B. The H‐bond interaction was the primary contact between the nitrogen atom of pyrazole and the residues of GLN160. Additionally, the THIQ skeleton formed a primary hydrophobic interaction with residues PHE163 and PHE750 in the simulated process. These results suggest that compound **14a** has a mode similar to that of the positive control. To derive the dynamic behavior of the residues at the active sites of 8A9K/compound **14a**, the complexes were simulated in a real solution environment using MD simulations. The RMSD values of most of the residues were below 3 Å, indicating no large fluctuations. Comparative analysis of Cα RMSDs revealed that the complex exhibits a lower RMSD value than the apo protein, indicating that **14a** forms a stable complex with the protein, reducing its conformational fluctuations and thereby enhancing stability (Figure 5C).

**FIGURE 6:**
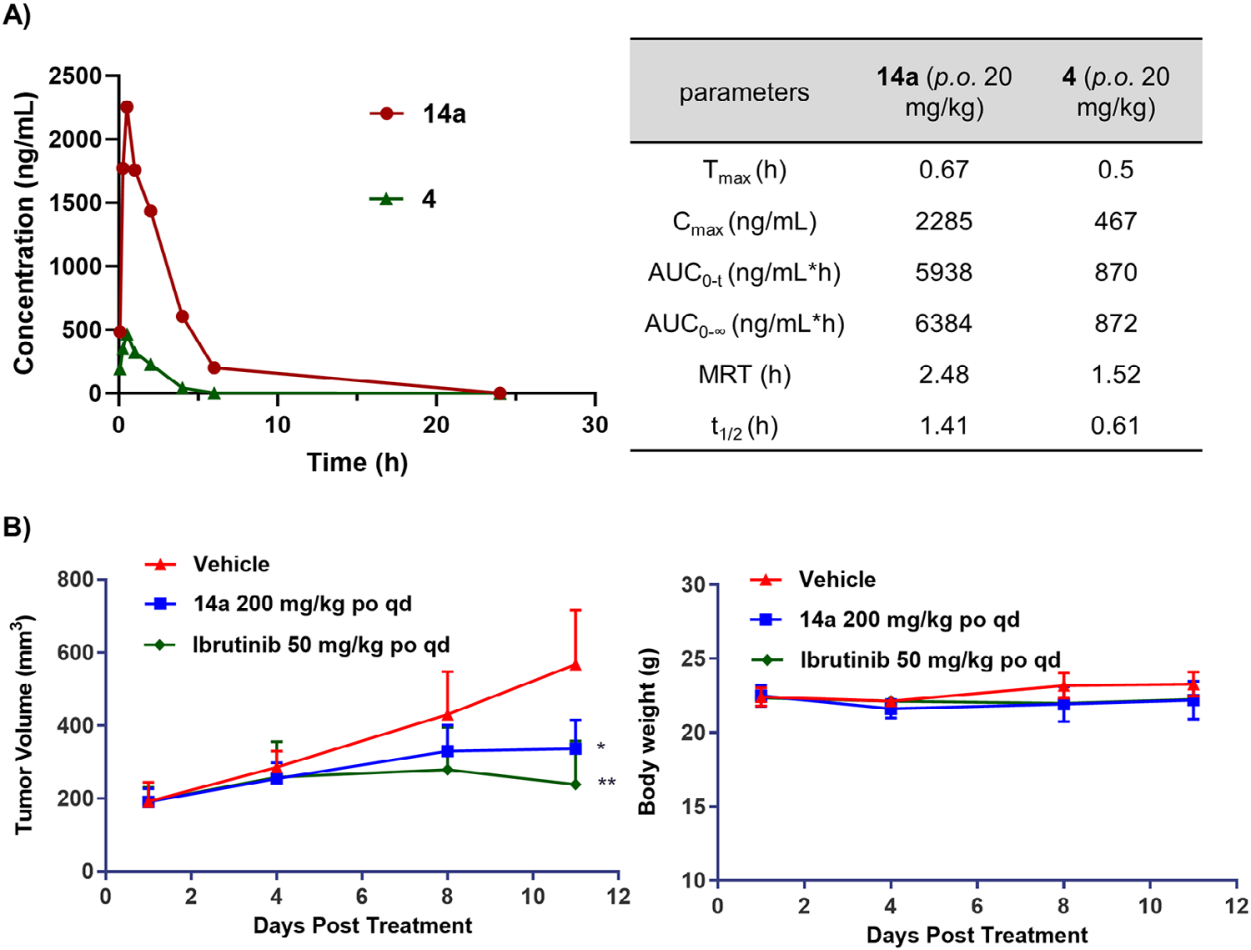
In vivo PK parameters and anti‐DLBCL efficacy of compound **14a**. (A) PK profiles of **14a** and **4** in ICR mice (n = 3). Data are shown as mean values. (B) Tumor volume and body weight changes of NOD‐SCID mice treated with **14a, ibrutinib,** or vehicle control in a subcutaneous transplantation model of OCI‐LY10 cells (n = 5). Data are presented as mean ± SD. ^*^ *p* < 0.05, ^**^ *p* < 0.01 versus vehicle, by two‐tailed Student's *t*‐test.

Given their potent activities in biochemical and cellular assays, we performed an in vivo PK study in ICR mice to investigate the PK profiles of **14a** and **4** (Figure 6A). As expected, the structural optimization of **14a** yielded improved PK properties relative to **4**. Following oral administration to ICR mice at doses of 20 mg/kg, compound **14a** exhibited a moderate exposure (AUC_0−∞_ = 6384 ng mL^−1^ h^−1^), high maximum concentration (C_max_ = 2285 ng/mL), and a desired half‐life (t_1/2_ = 1.41 h). In addition, **14a** possesses acceptable metabolic stability (MF = 28.4%) and exhibits no significant inhibition of the tested CYP450 enzymes (IC_50_ > 25 µM) (Tables S4 and S5). Overall, the encouraging PK properties of **14a** motivated further evaluation of its in vivo antitumor efficacy. OCI‐LY10 xenografts were used for in vivo efficacy evaluation. NOD‐SCID mice bearing OCI‐LY10 tumors were randomized and treated with compound **14a** by oral administration at doses of 200 mg/kg for 11 consecutive days. As shown in Figure 6B, the T/C ratio of compound **14a** at the treatment endpoint was 56.77%. We also verified the in vivo safety profiles of the compound **14a** in mice. A single‐dose toxicity was conducted at doses of 400 mg/kg and 800 mg/kg in comparison with the vehicle group. No significant changes were observed in serum biochemical parameters or body weight (Figure S5A,B). Notably, the toxicity test results and the absence of adverse effects (diarrhea, emaciation, or body weight reduction) in treated mice support the favorable safety profile of compound **14a** (Figure 6B). Taken together, these in vivo results indicate that **14a** effectively suppresses tumor growth with good tolerance.

## 3 Conclusions

In summary, we developed the first allosteric fluoroprobe and FP assay, allowing for the direct validation of USP1 allosteric inhibitors. Allosteric tracer **6‐2** showed favorable fluorescence properties and was suitable for further biological evaluation in vitro. The FP assay, based on allosteric tracer **6‐2**, enabled the differentiation of known allosteric and catalytic site inhibitors. Furthermore, **6‐3** served as a tool for visualizing target engagement with USP1 in live CAOV‐3 cells. Highly selective USP1 inhibitors represent a novel and promising approach for the treatment of DLBCL. In this study, we designed and synthesized a novel class of potent THIQ‐based USP1 inhibitors. The representative compound **14a** (IC_50_ = 29.9 ± 1.5 nM) exhibited more potent enzymatic activity than the clinical candidate **5** (IC_50_ = 47.1 ± 3.0 nM) and high selectivity. Furthermore, **14a** showed particularly strong potency in OCI‐LY10 (IC_50_ = 240.93 ± 43.35 nM) and TMD‐8 (IC_50_ = 29.9 ± 8.8 nM) cells and had negligible activity on normal cells. Moreover, compound **14a** effectively induced apoptosis in OCI‐LY10 cells in a dose‐dependent manner; accumulation of OCI‐LY10 cells in the S phase reduced the oncogenic protein levels of MYC and led to the accumulation of Ub‐PCNA. Moreover, the combination of **14a** and **olaparib** showed potent antiproliferative activity in classic USP1‐overexpressing MDA‐MB‐436 and CAOV‐3 cell lines, comparable to **5**. We successfully probed the interactions between **14a** and the protein using molecular simulations. **14a** displayed favorable PK properties and repressed the growth of OCI‐LY10 cells in a xenograft tumor model with no significant toxicity. Collectively, our findings provide information about a valuable fluoroprobe, FP assay platform, and lead compounds targeting USP1 for further structural optimization and antitumor mechanism studies.

## 4 Experimental Section

### 4.1 Ethics Statement

All animal experiments were approved by the Animal Experiments Ethics Committee of Zhongshan Institute for Drug Discovery (IACUC: 2025‐02‐LJ‐01) and Shanghai Institute of Materia Medica (IACUC: 2025‐01‐LJ‐195 and 2026‐01‐LJ‐216). All animal experiments were conducted in strict accordance with the guidelines for the management and use of experimental animals by these institutions. This article does not contain any studies with human participants performed by any of the authors.

### 4.2 Enzyme Activity and Inhibition Studies

USP1 activity and its inhibition by **14a** were determined by detection Ub‐Rho hydrolysis in an endpoint method. In the absence of an inhibitor, USP1 hydrolyzes Ub‐Rho, resulting in the release of free Rho and a concomitant increase in fluorescence. Compounds were pre‐incubated with enzyme buffer in a 384‐well plate (ProxiPlate‐384 Plus, PerkinElmer) for 30 min at room temperature. The reaction was initiated by adding substrate to a final volume of 10 µL, containing 2% DMSO, 0.08 nM USP1, 250 nM Ub‐Rho 110, 50 mM HEPES (pH 7.8), 100 mM NaCl, 0.5 mM EDTA, 1 mM DTT, 0.1 mg/mL BSA, and 0.01% Tween‐20. Control groups included a blank (no enzyme), a vehicle control (DMSO instead of compound), and a positive control. After 1 h incubation at room temperature, fluorescence signals were detected using an Envision reader, with excitation and emission wavelengths set to 480 and 540 nm, respectively. In the data processing, the ratio of the concentration to the active percentage was plotted, and then the fitting and curve were calculated using nonlinear regression, and the IC_50_ value of the compound was calculated using the software GraphPad Prism 10.4.2. Protein USP1 and Ub‐Rho were purchased from R&D Systems.

### 4.3 Other USP Family Members Inhibition Assay

To measure the activity of other USP family members, inhibition against Ubiquitin‐specific proteases (USP2, USP7, USP10, USP28) were carried out as follows: the indicating enzymes (USP2, USP7, USP10 or USP28) were diluted into 50 nM final concentration in a buffer containing 50 mM HEPES pH 8.0, 100 mM NaCl, 0.5 mM EDTA, 1 mM DTT, 0.1 mg/mL BSA and 0.01% Tween‐20, then 4 µL the enzymes were incubated with 2 µL compounds (2% final DMSO concentration) for 30 min at room temperature in 384‐well black polystyrene plate, then the enzymatic reaction was initiated by adding 4 µL of 250 nM final concentration of substrate Ub‐AMC (R&D Systems) and incubated at room temperature for 1 h. The fluorescence signal was monitored using Envision (Perkin‐Elmer) using excitation and emission wavelengths of 355 nm and 460 nm, and the inhibition rates were calculated by normalized to the DMSO control group. USP2, USP7, and USP28 were obtained from Sino Biological Inc, and USP10 was purchased from BPS Bioscience.

### 4.4 Other Proteins Inhibition Assays

Kinase inhibition assays were carried out using HTRF KinEASE kit (62TK0PEB or 62ST1PEB, PerkinElmer) according to the protocol supplied by the manufacturer. Different protein kinases, peptide substrates (0.06 ng/µL CHK1, 1 µM S1, 50 µM ATP for the CHK1 kinase assay; 0.5 ng/µL FLT3‐WT, 1 µM TK‐S, 2 µM ATP for the FLT3‐WT kinase assay; 0.8 ng/µL AKT1, 1 µM S1, 20 µM ATP for the AKT1 kinase assay and 0.2 ng/µL JAK3, 1 µM TK‐S, 2 µM ATP for the JAK3 kinase assay), and compounds were added to a 384‐well reaction plate (Proxiplate‐384 Plus, PerkinElmer). The final volume of the reactions was 10 µL in reaction buffer (1×kinase buffer, 5 mM MgCl_2_, and 1 mM DTT). The kinase reactions were incubated at room temperature for 1 h, and 5 µL of the detection buffer (SA‐XL665 and antibody Eu (K)) was added to the mixture for detection. Fluorescence signals were collected on an EnVision multimode microplate reader (PerkinElmer) with excitation at 340 nm and emission at 620 nm and 665 nm, the inhibition rates were calculated by normalized to DMSO control group. CHK1 was purchased from Sino Bioligical Inc., FLT3‐WT was purchased from Carna Biosciences, Inc, JAK3 was purchased from Eurofins Scientific, and AKT1 was expressed using an E. coli expression system.

CDK2 inhibition assay was carried out using ADP‐Glo kinase kit (V6930, Promega). Briefly, the enzyme CDK2 (Eurofins Scientific) and substrate Ulight‐4EBP‐1(PerkinElmer) were diluted into 2 ng/µL and 50 nM final concentration, respectively, in a buffer containing 50 mM HEPES pH 7.5, 10 mM MgCl_2_, 3 mM MnCl_2_, 0.01%Tween20 and 2 mM DTT. Then 2 µL enzyme, 2 µL substrate, and 1 µL compounds were added to a 384‐well reaction plate (Proxiplate‐384 Plus, PerkinElmer), followed by incubation for 2 h at room temperature. In the reaction, ATPs were converted to ADPs by CDK2. The levels of ADPs in the well, representing the activity of CDK2, were detected using the ADP‐Glo kit according to the manufacturer's instructions, the inhibition rates were calculated by normalized to DMSO control group.

ACLY activity detection using an ADP‐Glo dependent assay. Briefly, the reaction was performed in a 384‐well plate, containing substrates (5 µM ATP, 30 µmol/L citrate, 15 µM CoA) within assay buffer (40 mM Tris ‐HCl, pH 7.5, 10 mM MgCl_2_, 5 mM DTT). The reaction was initiated by adding 20 nM recombinant human ACLY protein (Sino Bioligical Inc.) and compounds into the well, followed by incubation for 30 min at 37°C. In the reaction, ATPs were converted to ADPs by ACLY. The levels of ADPs in the well, representing the activity of ACLY, were detected using the ADP‐Glo kit (V9102, Promega) according to the manufacturer's instructions. The inhibition rates were calculated by normalized to DMSO control group.

OGA inhibition assay was carried out in a 10 µL volume containing 0.5 ng/µL OGA (OGA was expressed using an E. coli expression system) and 100 µM substrate MU‐GlcNAc (Sigma‐Aldrich) in 50 mM Na_2_HPO_4_, pH 7.0, 100 mM NaCl, 0.1% BSA reaction buffer. After incubating at room temperature for 1 h, the fluorescence signal was monitored using Envision (Perkin‐Elmer) using excitation and emission wavelengths of 320 and 450 nm, the inhibition rates were calculated by normalized to DMSO control group.

PRMT5 inhibition assay was carried out using MTase‐Glo Methyltransferase assay kit (V7602, Promega). Briefly, the reaction was performed in a 384‐well plate, containing 1 ng/µL PRMT5 enzyme (Active Motif) and substrates(1 µM H4, 5 µM SAM) within assay buffer (20 mM Tris‐HCl, pH 8.0, 50 mM NaCl, 3 mM MgCl_2_, 0.1 mg/mL BSA and 1 mM EDTA). After incubating at room temperature for 2 h, the MTase‐Glo Reagent is added to convert SAH to ADP. The MTase‐Glo Detection Solution is then added to convert ADP to ATP, which is detected via a luciferase reaction. The luminescent signal was monitored using Envision (Perkin‐Elmer), the inhibition rates were calculated by normalized to DMSO control group.

PTP1B, CDC25B, and DPP4 inhibition assays were assayed by a continuous fluorometric assay, which is cleaved by the enzyme to release fluorescence. The indicating enzymes (PTP1B, CDC25B and DPP4 were expressed using an E. coli expression system) were diluted into 2 nM final concentration in a buffer containing 60 mM HEPES, pH 7.2, 75 mM NaCl, 75 mM KCl, 1 mM EDTA, 0.05% Tween‐20 and 5 mM DTT, then 20 µL the enzymes were incubated with 10 µL compounds for 20 mins at room temperature in 384‐well black polystyrene plate, then the enzymatic reaction was initiated by adding 20 µL of 20 µM final concentration of substrates (6,8‐Difluoro‐4‐Methylumbelliferyl Phosphate (DiFMUP) for PTP1B and CDC25B, Gly‐Pro‐AMC for DPP4). The fluorescence signal was monitored using an excitation wavelength of 355 nm and an emission wavelength of 460 nm using Envision (PerkinElmer), the inhibition rates were calculated by normalized to DMSO control group. Substrates, DiFMUP, were obtained from MCE, Gly‐Pro‐AMC, were obtained from GL Biochem (Shanghai, China).

### 4.5 CYP450 Inhibition Assays

CYP450 inhibition assays were carried out using P450‐Glo Assays kit (V9770, V9890, V9910, V9790 or V9880, Promega) according to the protocol supplied by the manufacturer. A CYP enzyme and a P450‐Glo substrate are combined in 100 mM potassium phosphate (K_3_PO_4_) buffer (10 nM CYP1A2, 100 µM Luciferin‐ME for the CYP1A2 assay; 5 nM CYP2D6, 30 µM Luciferin‐ME EGE for the CYP2D6 assay and 2 nM CYP3A4, 25 µM Luciferin‐PPXE for the CYP3A4 assay), then 2.5 µL the CYP450 reaction mixture were incubated with 2.5 µL compounds (0.25% final DMSO concentration) for 30 mins at room temperature in 384‐well plate (Proxiplate‐384 Plus, PerkinElmer), and the reaction is initiated by adding 5 µL NADPH regenerating system, followed by incubation for 30 mins at 37°C. Then, add an equal volume of Luciferin Detection Reagent. This reagent simultaneously stops the CYP reaction and initiates a luminescent signal that is proportional to the amount of CYP activity. Signals are then allowed to stabilize for 20 mins at room temperature before reading luminescence using Envision (Perkin‐Elmer). In the data processing, the ratio of the concentration to the active percentage was plotted, and then the fitting and curve were calculated using nonlinear regression, and the IC_50_ value of the compound was calculated using the software GraphPad Prism 10.4.2.

### 4.6 Bio‐Layer Interferometry Analysis

BLI analyses were performed at 27 °C using a ForteBio Octet Red96 biosensor system (ForteBio) with Ni‐NTA Biosensors (Sartorius, 18–5101). The His‐USP1 protein (50 µg/mL) was immobilized onto the NTA biosensor (Sartorius,18‐5101) for 10 min. The tips were washed with the buffer (50 mM HEPES, pH 7.8, 100 mM NaCl, 0.5 mM EDTA, 1 mM DTT, 0.01% Tween‐20, 0.1 mg/mL BSA) for 30 s to obtain a baseline reading, then the biosensors were dipped into wells containing various concentrations of compounds for 45 s, which was followed by a 45 s buffer wash to allow the dissociation of compounds from the sensor. Global fitting of the binding curves generated a best fit with the 1:1 model, and the kinetic association and dissociation constants were calculated. The systematic baseline drifts were corrected by double subtracting the shifts recorded from sensors loaded with ligands but incubated with no analytes and another reference sensor loaded without ligands but running the same assay. All binding experiments were performed in black 96‐well plates (Gerner) containing 200 µL of assay buffer in each well at 25°C with an agitation speed of 1000 rpm. Curve fitting, steady state analysis, and calculation of kinetic parameters (*K*_on_, *K*_off_, and *K*_d_) and Rmax parameters were done using Octet software version 9.0 (ForteBio). The goodness of fit for the binding data was assessed by evaluation of the χ^2^ and R^2^ values generated from all the fitting analyses.

### 4.7 Instrument

The UV–vis spectra were carried out on a SHIMADZU UV–vis spectrophotometer (UV‐2600i). The fluorescence spectra were measured with an Agilent Cary Eclipse fluorescence spectrophotometer.

### 4.8 The Measurement of Optical Properties

The probe was first dissolved in DMSO to obtain 5 mM stock solution, and then the stock solution was diluted to be 5 µM with PBS buffer solution (pH = 7.4). The UV–vis and fluorescence spectra were acquired with 5 µM probe on a SHIMADZU UV–vis spectrophotometer and an Agilent fluorescence spectrophotometer, respectively. The quantum yield of the probe was calculated in PBS buffer (pH = 7.4) and selected fluorescein in 0.1 M NaOH aqueous solution as the reference by the following equation:

ΦX=ΦST(AST/AX)(FX/FST)(ηx/ηST)2

where the subscripts ST and X denote standard and test, respectively, *Φ* is the quantum yield, F is the integrated area under the fluorescence spectra, A is the absorbance, and η is the refractive index of the solvent.

### 4.9 Fluorescence Polarization (FP) Assay

The FP assay was performed with the buffer (50 mM HEPES, pH 7.8, 100 mM NaCl, 0.5 mM EDTA, 1 mM DTT, 0.01% Tween‐20, 0.1 mg/mL BSA), which was also used to dilute protein and probe to the required concentration. Compounds were pre‐incubated with enzyme buffer in a 384‐well plate (Corning, 3575) for 20 min at room temperature. The reaction was initiated by adding probe to a final volume of 50 µL, containing 100 nM USP1, 0.5 nM probe in the assay buffer. Change in fluorescence polarization was monitored at 535 nm after excitation at 480 nm using the Envison protocol. The ratio of the concentration to the occupancy percentage was plotted, and then the fitting and curve were calculated using nonlinear regression, and the IC_50_ value of the compound was calculated using the software GraphPad Prism 10.4.2

### 4.10 Fluorescence Microscopy

Caov3 in the logarithmic growth phase were seeded in 24‐well culture plates, 6 × 10^4^ cells per well, and cultured with 0.5 mL culture medium overnight at 37°C and 5% CO_2_. Cells were washed twice with PBS. The cells were fixed with 4% paraformaldehyde solution for 1 h, washed with PBS, and then cultured with 0.5 mL PBS containing the corresponding concentrations of the probe **6‐2**. After processing for 24 h, the fluorescence images of differently treated cells were observed by fluorescence microscopy after being washed twice with PBS. Caov3 in the logarithmic growth phase were seeded in 24‐well culture plates, 6 × 10^4^ cells per well, and cultured with 0.5 mL culture medium containing the corresponding concentrations of the probe **6‐3**. After processing for 4 h, cells were washed twice with PBS. The fluorescence images of differently treated cells were observed by Incucyte.

### 4.11 Cell Lines and Cell Culture

OCI‐LY10 (IMDM+20% FBS) was donated by doctor Jef De Brabander (UT Southwestern Medical Center). TMD‐8 (RPMI 1640+10% FBS) was donated by doctor Lynn's group (The University of Chicago). Molm16 (1640+15%FBS) was donated by doctor Zhou hu's group (Shanghai Institute of Materia Medica). Molm13(RPMI 1640+20%FBS) was donated by doctor Geng meiyu's group (Shanghai Institute of Materia Medica). OCI‐LY3 (RPMI 1640+10%FBS) was donated by doctor Dong xiaowu's group (Zhejiang University). OCI‐AML3 (RPMI 1640+20%FBS) was purchased from Nanjing Cobioer Biotechnology Co., Ltd. Molt4, THP‐1 (RPMI 1640+10%FBS), and K562 (IMDM+10%FBS) were purchased from the National Collection of Authenticated Cell Cultures. PBMC (DMEM+10%) was purchased from the National Collection of Authenticated Cell Cultures. RS4;11, RPMI‐8226, MM.1S, BDCM, Toledo, SU‐DHL‐2, HT (1640+10%FBS), Mino (RPMI 1640+15%FBS), MV‐4‐11 (IMDM+10%FBS), HL‐60 (IMDM+20 %FBS), HS‐5 (DMEM+10%) was purchased from the American Type Culture Collection. Cell cultures were maintained at 37°C in an incubator containing 5% carbon dioxide.

### 4.12 Colony Formation Assay

MDA‐MB‐436 in the logarithmic growth phase were seeded in 6‐well culture plates, 1 × 10^4^ cells per well and cultured with 1 mL culture medium containing the corresponding concentrations of the compounds. After processing for 14 days, cells were washed twice with PBS, fixed with 4% paraformaldehyde solution for 1 h, washed with PBS, and then treated with 1% crystal violet solution. The wells were then oven‐dried. Images were taken using the Bio‐Rad ChemiDoc Imaging System.

### 4.13 Synergy Analyses

To assess the synergistic effects of drug combinations, we utilized the online tool SynergyFinder 3.0 (https://synergyfinder.fimm.fi/) to calculate the synergy score based on the ZIP algorithm for the given dose combinations. The system reported the average combination index for each dose combination. A synergy score greater than 10 indicates a synergistic effect, a synergy score between −10 and 10 indicates an additive effect, and a synergy score less than −10 indicates an antagonistic effect. The magnitude of the combination index (in absolute value) correlates with the degree of synergy.

### 4.14 Cellular Viability Assay

Cells in the logarithmic growth phase were seeded in 96‐well culture plates, and the appropriate number of cells was seeded according to the growth rate of the cells. Each well contained 200 µL of culture medium with the corresponding concentration of compounds. After 7 days of culture, 10 µL of CCK‐8 (Cat NO: SB‐CCK8, Share‐bio) was added to each well. After incubation for 2 h, the values were read using a SpectraMax 190 (Molecular Device) microplate reader. The difference in absorbance values between 450 nm and 650 nm was used as the final data. Finally, the IC_50_ value of the compound was calculated by the nonlinear regression method using GraphPad 10.1.2 software. Data results are from three independent replicate experiments.

### 4.15 Western Blot Assay

Cells in the logarithmic growth phase were seeded in 12‐well culture plates, 5 × 10^5^ cells per well, and cultured with 1 mL culture medium containing the corresponding concentrations of the compounds. After processing for 24 h, the cells were collected by centrifugation. Cells were washed once with precooled PBS followed by centrifugation to remove PBS, 100 µL loading buffer was added, cells were resuspended, and samples were boiled and denatured at 100°C for 20 min. Appropriate amounts of protein samples were subjected to Tricine‐SDS‐PAGE for electrophoresis. After electrophoresis, the proteins were transferred to nitrocellulose membranes, followed by blocking, overnight incubation of the primary antibody at 4°C, incubation of the secondary antibody in the next day, and finally exposure. Image Lab 6.0.1 was used to perform grayscale analysis. USP1 (Abclonal, Cat: A6785, Lot: 0202290101), MYC (CST, Cat: 13987, Lot: 6), Ub‐PCNA (CST, Cat: 13439, Lot: 4), PCNA (CST, Cat: 2586, Lot: 8), and β‐Actin (abcepta, Cat: AM1021B, Lot: SG210806W01) were used in the experiment.

### 4.16 Apoptosis and Cell Cycle Assay

OCI‐LY10 cells were seeded into a 12‐well plate at a density of 5 × 10^5^ cells/well; After 2 h later, OCI‐LY10 cells were incubated with different concentrations of **14a** for 72 h. Then, the cells were harvested and analyzed by an Annexin V‐FITC/PI apoptosis detection kit (Cat NO: KGA1102, KeyGEN) or Cell Cycle and Apoptosis Analysis Kit (Cat NO: C1052, Beyotime) according to the manufacturer's instructions. Flow cytometry analysis was performed using a CytoFLEX Flow Cytometer (Beckman) and quantified with FlowJo software.

### 4.17 Molecular Docking

The crystal structures of USP1 (PDB: 8A9K and 9FCI) were obtained from the Protein Data Bank. Compounds were optimized by the module of the Protein Preparation Wizard at pH 7.0. Next, prepared ligands were docked to the optimized protein by Glide with Standard Precision mode. And all of the other parameters for the above process were the default parameters. All docking studies were performed by Maestro of Schrödinger Suites (version 2018‐4), and the obtained poses were analyzed with PyMOL (version 2.5.5).

### 4.18 Molecular Dynamics Simulation

The molecular dynamics simulations were applied to investigate the binding mode given by molecular docking. Multiple stages were involved in the molecular dynamics: system building, simulation, and results analysis. First, the resulting structures from the docking process were introduced into the Maestro (version 2018‐4) and processed with the Protein Preparation Wizard in the Schrödinger package (version 2018‐4). After that, the refined structures were embedded in a simple point charge (SPC) water model, and the complexes were neutralized with an appropriate number of counter ions. The system was set in an orthorhombic box, which extended approximately 10 Å in each direction. The OPLS_3e force field was used in the protein‐ligand system. Subsequently, a minimization step was subjected to the system with the largest interaction set to 2000 and the convergence threshold set to 1.0 kcal/mol/Å in a hybrid method of the steepest descent and the limited‐memory Broyden–Fletcher–Goldfarb–Shanno algorithms (LBFGS). The system energy was reduced to a maximum of 5000 steps until it reached the gradient threshold of 25 kcal/mol/Å. The protein‐ligand systems were minimized at a temperature of 300 K in NPT ensemble using a Nose‐Hover thermostat at 300 K and Martyna–Tobias–Klein barostat at 1.01325 bar pressure. Finally, the systems were subjected to MD simulations for 100 ns, and the results were investigated using protein–ligand RMSD.

### 4.19 Single‐Dose Toxicity Study

Animal procedures were approved by the Institutional Animal Care and Use Committee of the Shanghai Institute of Materia Medica (approval No. 2026‐01‐LJ‐216). The ICR mice were randomly divided into three groups and received compound **14a** (400 mg/kg, 800 mg/kg, i.g.) or vehicle (5%DMSO+5%Solutol HS15+90% Normal Saline, i.g.) for Single‐dose. Compound **14a** was dissolved in 5%DMSO+5%Solutol HS15+90% Normal Saline. Six animals per group, with an equal number of males and females. Observe for 6 days following a single dose, establish clinical observation, body weight, and food intake monitoring parameters. At the end of the experiment, the mice were sacrificed, and the serum biochemistry parameters were recorded on Sysmex XT‐2000i and Roche Cobas c 311, respectively.

### 4.20 In Vivo Pharmacokinetic PK Studies

Animal procedures were approved by the Institutional Animal Care and Use Committee of the Shanghai Institute of Materia Medica (approval No. 2025‐01‐LJ‐195). The female ICR mice (6–8 weeks, 18–22 g, Shanghai Lingchang BioTech Co., Ltd., China) were used in the pharmacokinetic study. Mice received the test compounds orally at a dose of 20 mg/kg. Plasma samples were collected at indicated time points (0.083, 0.25, 0.5, 1, 2, 4, 6, and 24 h) before they were deproteined with 200 µL of MeCN/MeOH (1/1, v/v). After further centrifugation, supernatant was collected and diluted with an equal volume of water. The concentration of test compounds was analyzed by LC‐MS/MS.

### 4.21 Mouse Tumor Model

Female NOD‐SCID mice were purchased from Guangdong Weitonglihua Laboratory Animal Technology Co., Lt. All animal studies were performed in strict accordance with the institutional guidelines as defined by the Institutional Animal Care and Use Committee, approved by the Animal Care and Use Committee, Zhongshan Institute for Drug Discovery (Zhongshan, China).

6‐ to 8‐week‐old female NOD‐SCID mice were sc injected into the right flank with human OCI‐LY10 cells (1× 10^7^ cells/mouse). After the tumor grew to 100−300 mm^3^, mice were divided into a negative control group and an administration group. Then, mice were orally administered the test compound **14a** (200 mg/kg), **ibrutinib** (50 mg/kg), or vehicle once daily for 11 days.

During the experiment, the tumor volume was measured twice a week, and the mice were weighed. Tumor diameter was measured using a caliper, and tumor volume was determined by calculating the volume of an ellipsoid using the formula: length × width 2 × 0.5. Body weight was measured using a standard balance. Data are expressed as mean ± SEM from n = 5 mice/group (IACUC: 2025‐02‐LJ‐01).

### 4.22 Statistical Analysis

All data are expressed as the mean ± standard deviation (SD). The numbers of animals, cells, and experimental replicates can be found in the materials and methods or figure legends. Statistical differences between experimental groups were determined using Student's t‐test. Significance was set at P < 0.05. The software used for statistical analysis was GraphPad Prism 10 and Excel.

## Author Contributions

J.W.C. contributed to methodology, investigation, data curation, visualization, and writing – original draft; P.P.W. contributed to methodology and visualization; P.F.W. contributed to methodology, investigation, and data curation, visualization, and writing – original draft; J.M.W., B.Y.L., D.Q.F., and J.H. contributed to investigation; X.B.H. and W.J.K. contributed to investigation and validation; Y.B.Z. contributed to supervision; C.P.L. contributed to conceptualization, writing – review & editing, supervision, and funding acquisition; J.L. contributed to funding acquisition, and resources; H.L. contributed to conceptualization, writing – review & editing, supervision, resources, and funding acquisition. All authors have read and approved the final version of the manuscript.

## Conflicts of Interest

The authors declare no conflict of interest.

## Supporting information




**Supporting File**: advs75350‐sup‐0001‐SuppMat.docx.

## Data Availability

All data generated or analyzed during this study are included in the manuscript and supporting files. All data that support the findings of the study are available from the corresponding authors upon reasonable request.
