# Supplementary material for: Discovery of a Potent Fluorescence Polarization Probe for Identifying USP1 Allosteric Inhibitors
Source: Adv Sci (Weinh). 2026 Apr 22;13(39):e75350. doi: 10.1002/advs.75350 (PMC13335693; doi:10.1002/advs.75350)
Supplement: Supplementary file 1 — Supporting File: advs75350‐sup‐0001‐SuppMat.docx. [file ADVS-13-e75350-s001.docx]

**Supporting information**

# Discovery of a Potent Fluorescence Polarization Probe for Identifying USP1 Allosteric Inhibitors

*Jiawei Cheng,^[a, b, ‡]^ Peipei Wang,^[d, ‡]^ Pengfei Wang,^[b, c, h ‡]^ Jiamin Wang,^[b]^ Baiyang Li, ^[d, b]^ Daqing Fang,^[e]^ Jian He,^[b, g]^ Xiaobei Hu,^[f]^ Weijuan Kan,^[b]^ Yubo Zhou,^* [f]^ Chunpu Li,^* [c, b, h]^ Jia Li,^* [d, b, f]^ and Hong Liu^* [a, b, c, d, e, h]^*

^[a]^ Key Laboratory of Structure-Based Drug Design and Discovery, Ministry of Education, Shenyang Pharmaceutical University, Shenyang 110016, China.

E-mail: hliu@simm.ac.cn

^[b]^ State Key Laboratory of Drug Research, Shanghai Institute of Materia Medica, Chinese Academy of Sciences, Shanghai 201203, China.

^[c]^ School of Pharmaceutical Science and Technology, Hangzhou Institute for Advanced Study, UCAS, Hangzhou 310024, China.

^[d]^ School of Chinese Materia Medica, Nanjing University of Chinese Medicine, Nanjing 210023, China.

^[e]^ Shandong Laboratory of Yantai Drug Discovery, Bohai Rim Advanced Research Institute for Drug Discovery, Yantai 264117, China.

^[f]^ Zhongshan Institute for Drug Discovery, Shanghai Institute of Materia Medica, Chinese Academy of Sciences, Zhongshan 528400, China.

^[g]^ Air Force Hospital of Western Theater Command, Chengdu 610065, China.

^[h]^ University of Chinese Academy of Sciences, Beijing 100049, China.

^[‡]^ These authors contributed equally to this work.

^*^ Corresponding Author For Y.-B. Z., ybzhou@simm.ac.cn; C.-P. L., E-mail, lichunpu@simm.ac.cn; For J. L., email: jli@simm.ac.cn; For H. L., email: hliu@simm.ac.cn.

**Table of Contents**

1. **Synthesis Route of Compounds…………………………………………………………S3**
2. **Structure-Activity Relationships (SARs) of THIQ Derivatives……………S5**
3. **Experimental section…………………………………………………………………………S8**
4. **Figure S1. ……………………………………………………………………………………S34**
5. **Figure S2. ……………………………………………………………………………………S34**
6. **Figure S3. ……………………………………………………………………………………S35**
7. **Figure S4. ……………………………………………………………………………………S36**
8. **Figure S5. ……………………………………………………………………………………S36**
9. **Nuclear magnetic resonance (NMR) traces of target compounds**

**……………………………………………………………………………………………………S38**

1. **High performance liquid chromatography (HPLC) traces of target compounds……………………………………………………………………………………………S130**

**Synthesis Route of Compounds**

Compounds **13a**−**13p** and **14a**−**14y** were synthesized according to the synthetic protocols in **Scheme 1**. The commercially available *tert*-butyl 6-(hydroxymethyl)-3,4-dihydroisoquinoline-2(1*H*)-carboxylate **7** underwent an oxidation reaction to produce intermediate **8**. The intermediate **8** was subjected to the Debus-Radziszewski reaction with 1,1-dibromo-3,3,3-trifluoroacetone to form intermediate **9**. The intermediate was subsequently reacted with various alkyl iodides to yield intermediates **10a**-**10c**. **10a**-**10c** were converted to **11a**-**11c** through the deprotection of the *tert*-butoxycarbonyl (Boc) group and subsequently underwent a nucleophilic substitution reaction with various dichlorinated derivatives to afford **12a**-**12s**. Finally, **12a**−**12s** was reacted with various boronic acids or boronic esters in a Suzuki coupling to yield compounds **13a**−**13p** and **14a**−**14y**. The synthesis of intermediate **15** were using the same procedures. Intermediate **15** was converted to **16** through the deprotection of the Boc group, and then condensed with FITC in the presence of TEA to yield **6-1**. Meanwhile, the intermediate **16** was reacted with *tert*-butyl (4-bromobutyl)carbamate or *tert*-butyl(6-bromohexyl)(methyl)carbamate to form **17** or **19**, which was followed by deprotection of the boc group and condensed with FITC or TRITC in the presence of TEA to yield **6-2** and **6-3**.

**Scheme 1.** Synthesis of Desired Compounds *^a^*

*^a^*Reagents and conditions: (a) MnO_2_, DCM, r.t., 12 h; (b) NaOAc, H_2_O, MeOH, NH_3_·H_2_O, 90 ℃, 30 min, r.t., 12 h; (c) R_3_I, Cs_2_CO_3_, DMF, 90 ℃, 48 h; (d) TFA, DCM, rt, 3 h; (e) DMF, Et_3_N, 75 ℃, 12 h; (f) Pd(PPh_3_)_4_, K_2_CO_3_, dioxane/H_2_O, reflux, 12 h; (g) TFA, DCM, 40 ℃, 12 h; (h) *tert*-butyl (4-bromobutyl)carbamate, DMF, Et_3_N, 75 ℃, 12 h; (i) 6-FITC, Et_3_N, EtOH/THF, 0 ℃, 12 h. (j) *tert*-butyl(6-bromohexyl) (methyl)carbamate, DMF, Et_3_N, 75 ℃, 12 h; (k) 6-TRITC (Tetramethylrhodamine-6-isothiocyanate), Et_3_N, EtOH/THF, 0℃, 12 h.

# Structure-Activity Relationships (SARs) of THIQ derivatives

**Table S1.** Structural Comparison and in Vitro Enzyme Inhibition Activities in Core Region R_1_.

| **Compd.** | **R_2_** | **R_1_** | **USP1**  **IC_50_ (nM)** | | **Compd.** | **R_2_** | | **R_1_** | | **USP1**  **IC_50_ (nM)** | |
| --- | --- | --- | --- | --- | --- | --- | --- | --- | --- | --- | --- |
| **13a** |  |  | 683.3 ± 59.5 | | **13i** |  | |  | | ＞ 10 μM | |
| **13b** |  |  | 179.6 ± 17.5 | | **13j** |  | |  | | 5734.5±1788.3 | |
| **13c** |  |  | ＞ 10 μM | | **13k** |  | |  | | 70.8 ± 3.2 | |
| **13d** |  |  | ＞ 10 μM | | **13l** |  | |  | | 68.8 ± 5.1 | |
| **13e** |  |  | 177.0±0.3 | | **13m** |  | |  | | 102.5 ± 3.1 | |
| **13f** |  |  | 7545.0±219.2 | | **13n** |  | |  | | 45.7 ± 1.9 | |
| **13g** |  |  | 1197.5 ± 37.4 | | **13o** |  | |  | | 68.5 ± 17.5 | |
| **13h** |  |  | 510.4 ± 54.0 | | **13p** |  | |  | | 76.7 ± 1.1 | |
|  |  |  |  | **ML323** | | | - | | 180.0 ± 20.0 | |  |
|  |  |  |  | **KSQ-4279** | | | - | | 47.1 ± 3.0 | |  |

**Table S2.** Structural Comparison and in Vitro Enzyme Inhibition Activities in Solvent Region R_2_ and Hydrophobic Region R_3_.

| **Compd.** | **R_2_** | **R_3_** | **USP1**  **IC_50_ (nM)** | **Compd.** | **R_2_** | **R_3_** | **USP1**  **IC_50_ (nM)** |
| --- | --- | --- | --- | --- | --- | --- | --- |
| **14a** |  |  | 29.9 ± 1.5 | **14m** |  |  | 2422.0 ± 131.5 |
| **14b** |  |  | 48.3 ± 1.7 | **14n** |  |  | 1418.5 ± 60.1 |
| **14c** |  |  | 9307.5 ± 1093.9 | **14o** |  |  | ＞10 μM |
| **14d** |  |  | 1623.0 ± 125.8 | **14p** |  |  | 7915.5 ± 1635.5 |
| **14e** |  |  | 4750.5 ± 43.1 | **14q** |  |  | 95.4 ± 14.0 |
| **14f** |  |  | ＞10 μM | **14r** |  |  | 419.4 ± 70.7 |
| **14g** |  |  | ＞10 μM | **14s** |  |  | 1057.7±96.5 |
| **14h** |  |  | ＞10 μM | **14t** |  |  | ＞10 μM |
| **14i** |  |  | 2197.5 ± 28.9 | **14u** |  |  | 8553.0 ± 1444.6 |
| **14j** |  |  | 409.4 ± 26.5 | **14v** |  |  | 3221.0 ± 591.1 |
| **14k** |  |  | 412.5 ± 286.5 | **14w** |  |  | ＞10 μM |
| **14l** |  |  | 380.8 ± 78.9 | **14y** |  |  | ＞10 μM |
|  |  |  |  | **KSQ-4279** | | - | 47.1 ± 3.0 |

**Schem S2.** SARs summary.

After the completion of evaluation for USP1 enzymatic activity of all compounds, SARs were developed and are summarized subsequently. To sum up, (1) the electronic donation effect of fused rings enhances inhibitory activity, whereas electron-withdrawing groups lose activity; the target compounds with the furo[*3,2-d*]pyrimidine core at R_1_ exhibited the most potent inhibitory activity against USP1; (2) small groups improve inhibitory activity, whereas bulky groups reduce the inhibitory activity; the target compounds with ethyl group at R_2_ exhibited the most potent inhibitory activity against USP1; (3) the *ortho*-disubstitution at R_3_ shows a decisive effect on USP1 inhibitory activity; the target compound with the 2-cyclopropyl-6 methoxypyrimidine group on the R_3_ group possesses the most potent inhibitory activity against USP1.

# Experimental section

**Chemistry Materials and Data.**

The chemical reagents from commercial sources were used without further purification. Preparative thin layer chromatography (PTLC) was HSGF 254 (0.4−0.5 mm thickness).^1^H and ^13^C NMR spectra were recorded on a Bruker Avance III 400MHz, 500 or 600 MHz Spectrometer with tetramethylsilane (TMS) as an internal standard. Chemical shifts were reported in parts per million (ppm, *δ*) downfield from tetramethylsilane. Proton coupling patterns are described as singlet (s), doublet (d), triplet (t), quartet(q), multiplet (m), and broad (br). Low-resolution mass spectra(LRMS) were obtained with a Finnigan LCQ Deca XP mass spectrometer. High-resolution mass spectra (HRMS) were measured on a Micromass Ultra Q-TOF spectrometer. High-performance liquid-chromatography (HPLC) analysis of all final compounds was performed on an Agilent 1260 infinity series with an Agilent Extend-C18 column (150 × 4.6 mm, 3.5 μm) or Agilent XBridge-C18 column (150 × 4.6 mm, 3.5 μm) at 1 mL/min flow with a two-solvent system: MeOH/H_2_O. HPLC Conditions were as follows: (A) MeOH % (0-20 min: 75%); (B) MeOH % (0-10 min: 55-60%, 10-20min: 60-95%, 20-25 min: 95-95%); (C) MeOH % (0-10 min: 75%); (D) MeOH % (0-15 min: 75%); (E) MeOH % (0-30 min: 70%). All final compounds had an at least 95% purity.

*tert-butyl 6-formyl-3,4-dihydroisoquinoline-2(1H)-carboxylate (****8****).* Compound **7** (3.0 g, 11.39 mmol) was dissolved in dichloromethane, followed by the addition of manganese(IV) oxide (3.96 g, 45.57 mmol), stirred at room temperature for 12 h. After the reaction was completed, it was filtered to remove manganese(IV) oxide. The filter cake was washed with dichloromethane (3 × 50 mL) and concentrated to obtain a yellow oil. The oil was separated by column chromatography with petroleum ether/ethyl acetate (20/1 v/v) as an eluent to obtain a yellow oil as compound **8** (2.22 g, yield 74%).^1^H NMR (500 MHz, DMSO-*d6*) δ 9.96 (s, 1H), 7.72 (dt, *J* = 3.9, 2.3 Hz, 2H), 7.41 (d, *J* = 8.2 Hz, 1H), 4.60 (s, 2H), 3.58 (t, *J* = 5.8 Hz, 2H), 2.88 (t, *J* = 5.9 Hz, 2H), 1.44 (s, 9H). ESI-MS m/z: 262.3 [M+H]^+^.

*tert-butyl6-(4-(trifluoromethyl)-1H-imidazol-2-yl)-3,4-dihydroisoquinoline-2(1H)-carboxylate (****9****).* 1,1-dibromo-3,3,3-trifluoroacetone (2.29 g, 8.49 mmol) was dissolved in 15% sodium acetate solution and stirred at 90 ℃ for 0.5 h. Subsequently, it was cooled to room temperature, followed by the addition of compound **8** (2.0 g, 7.65 mmol), methyl alcohol (100 mL), NH_3_·H_2_O (75 mL), stirred at room temperature for 12 h. Upon completion, it was concentrated to remove methyl alcohol, and then was extracted three times with dichloromethane (3 × 100 mL). The combined organic phase was washed with brine (100 mL) and dried with anhydrous sodium sulfate. The solution was concentrated to give a yellow oil. The oil was separated by column chromatography with petroleum ether/ethyl acetate (20/3 v/v) as an eluent to obtain a yellow oil as compound **9** (1.52 g, yield 54%). ^1^H NMR (600 MHz, Chloroform-*d*) δ 9.97 (s, 1H), 7.70 (d, *J* = 7.9 Hz, 1H), 7.66 (s, 1H), 7.28 (s, 1H), 4.65 (s, 2H), 3.68 (s, 2H), 2.92 (t, *J* = 6.0 Hz, 2H), 1.50 (s, 9H). ESI-MS m/z: 368.3 [M +H]^+^.

*tert-butyl6-(1-ethyl-4-(trifluoromethyl)-1H-imidazol-2-yl)-3,4-dihydroisoquinoline-2(1H)-carboxylate (****10a****).* Compound **9** (3.0 g, 8.16 mmol) was dissolved in DMF (50 mL), followed by the addition of cesium carbonate (26.6 g, 81.6 mmol) and ethyl iodide (2.54 g, 16.33 mmol), stirred at 90 °C for 48 h. After the reaction was completed, it was filtered and filter cake was washed with ethyl acetate (3 × 50 mL). Subsequently, it was extracted three times with ethyl acetate (3 × 50 mL). The combined organic phase was washed with brine (100 mL) and dried with anhydrous sodium sulfate. The solution was concentrated to give a red oil. The oil was separated by column chromatography with petroleum ether/ethyl acetate (20/3 v/v) as an eluent to obtain a yellow oil as compound **10a**. ^1^H NMR (600 MHz, Chloroform-*d*) δ 9.97 (s, 1H), 7.70 (d, *J* = 7.9 Hz, 1H), 7.66 (s, 1H), 7.35 (d, *J* = 84.8 Hz, 1H), 4.65 (s, 2H), 4.53 – 4.43 (m, 2H), 3.68 (s, 2H), 2.92 (t, *J* = 5.9 Hz, 2H), 1.50 (s, 9H), 1.50 – 1.41 (m, 3H). ESI-MS m/z: 396.3 [M +H]^+^.

*6-(1-ethyl-4-(trifluoromethyl)-1H-imidazol-2-yl)-1,2,3,4-tetrahydroisoquinoline (****11a****)*

To a solution of **10a** (2 g, 5.06 mmol) in 30 mL of dichloromethane was added TFA (2.88 g, 25.31 mmol). The resulting mixture was stirred at room temperature for 3 h. Upon completion, the reaction mixture was concentrated and re-dissolved in dichloromethane (20 mL), followed by washing with 1.5 M NaOH (20 mL). The water phase was extracted three times with dichloromethane (3 × 50 mL). The combined organic phase was washed with brine (100 mL) and dried with anhydrous sodium sulfate. The solution was concentrated to give a yellow oil as compound **11a** (1.3 g, yield 87%), which was used without purification in the next reaction. ESI-MS m/z: 296.2 [M +H]^+^.

*2-chloro-4-(6-(1-ethyl-4-(trifluoromethyl)-1H-imidazol-2-yl)-3,4-dihydroisoquinolin-2(1H)-yl)furo[3,2-d]pyrimidine (****12a****).* To a solution of compound **11a** (1 g, 3.38 mmol) in DMF (20 mL) was added trimethylamine (0.69 g, 6.67 mmol) and 2,4-dichlorofuro[3,2-*d*] pyrimidine (0.64 g, 3.38 mmol), then stirred at 75 ℃ for 12 h. After the reaction was completed, it was extracted three times with ethyl acetate (3 × 50 mL). The combined organic phase was washed with 1.5 M HCl (20 mL) and brine (20 mL) and dried with anhydrous sodium sulfate. The solution was concentrated to give a yellow oil. The oil was separated by column chromatography with petroleum ether/ethyl acetate (2/1 v/v) as an eluent to obtain a white solid as compound **12a** (0.8 g, yield 53%).^1^H NMR (500 MHz, Chloroform-*d*) δ 7.81 (d, *J* = 2.2 Hz, 1H), 7.50 (d, *J* = 1.7 Hz, 1H), 7.43 (dd, *J* = 7.9, 1.8 Hz, 1H), 7.39 (q, *J* = 1.2 Hz, 1H), 7.35 (d, *J* = 8.0 Hz, 1H), 6.82 (d, *J* = 2.1 Hz, 1H), 5.18 (s, 2H), 4.31 (t, *J* = 5.9 Hz, 2H), 4.11 (q, *J* = 7.3 Hz, 2H), 3.10 (t, *J* = 5.9 Hz, 2H), 1.46 (t, *J* = 7.3 Hz, 3H). HRMS (ESI) m/z: calculated for C_21_H_17_ClF_3_N_5_O [M + H]^+^: 448.1074 found:448.1164.

*2-(4-cyclopropyl-6-methoxypyrimidin-5-yl)-4-(6-(1-ethyl-4-(trifluoromethyl)-1H-imidazol-2-yl)-3,4-dihydroisoquinolin-2(1H)-yl)furo[3,2-d]pyrimidine* *(****14a****).* Under argon, compound **12a** (0.2 g, 0.446 mmol), (4-cyclopropyl-6-methoxypyrimidin-5-yl) boronic acid (0.12 g, 0.580 mmol), were dissolved in 1.4-dioxane/H_2_O (20.0 mL), followed by the addition of Pd(PPh_3_)_4_ (0.046 g, 0.040 mmol) and potassium carbfonate (0.246 g, 1.786 mmol), stirred at 100 °C for 12 h. Upon completion, the mixture was filtered and the filter cake was washed with ethyl acetate (3 × 10 mL), and then extracted with ethyl acetate (3 × 20 mL) and water (H_2_O). The combined organic phase was washed with brine (20 mL) and dried with anhydrous sodium sulfate. The solution was concentrated to give a yellow solid. The solid was separated by column chromatography with petroleum ether/ethyl acetate (1/1 v/v) as an eluent to obtain a white solid as target compound **14a** (0.15 g, yield 60%). ^1^H NMR (600 MHz, Chloroform-*d*) δ 8.64 (s, 1H), 7.84 (d, *J* = 2.1 H z, 1H), 7.48 (d, *J* = 1.8 Hz, 1H), 7.39 (dd, *J* = 7.9, 1.8 Hz, 1H), 7.37 (d, *J* = 1.2 Hz, 1H), 7.31 (d, *J* = 7.9 Hz, 1H), 6.96 (s, 1H), 5.20 (s, 2H), 4.33 (t, *J* = 5.9 Hz, 2H), 4.08 (q, *J* = 7.3 Hz, 2H), 3.93 (s, 3H), 3.09 (t, *J* = 5.9 Hz, 2H), 1.81 (dq, *J* = 8.3, 4.6, 4.2 Hz, 1H), 1.44 (t, *J* = 7.3 Hz, 3H), 1.21 (dt, *J* = 6.5, 3.3 Hz, 2H), 0.88 (dq, *J* = 6.9, 3.9 Hz, 2H). 13C NMR (151 MHz, Chloroform-*d*) δ 168.70, 166.09, 156.53, 148.00, 147.88, 147.81, 135.02, 134.55, 132.86, 131.69 (q, *J* = 9.9 Hz), 131.33, 129.30, 128.14, 128.06, 127.69, 126.29, 126.23, 121.33(d, *J* = 266.9 Hz), 119.06 (d, *J* = 4.1 Hz), 107.66, 53.77, 41.81, 28.53, 24.44, 15.88, 13.62, 10.44. Condition A: HPLC purity 95.75 %, t_R_ = 7.853 min. HRMS (ESI) m/z: calculated for C_29_H_27_F_3_N_7_O_2_ [M + H]^+^: 562.2173 found: 562.2175.

*2-(4'-cyclopropyl-6'-methoxy-5-methyl-[2,5'-bipyrimidin]-4-yl)-6-(1-isopropyl-4-(trifluoromethyl)-1H-imidazol-2-yl)-1,2,3,4-tetrahydroisoquinoline (****13a****).* Compound **13a** was prepared in a manner similar to that described for compound **14a**: yield 63%; ^1^H NMR (500 MHz, Chloroform-*d*) δ 8.62 (s, 1H), 8.30 (s, 1H), 7.47 – 7.42 (m, 1H), 7.42 – 7.40 (m, 1H), 7.31 (dd, *J* = 7. 9, 1.8 Hz, 1H), 7.24 (d, *J* = 7.9 Hz, 1H), 4.78 (s, 2H), 4.58 (p, *J* = 6.7 Hz, 1H), 3.93 (s, 3H), 3.82 (t, *J* = 5.7 Hz, 2H), 3.08 (t, *J* = 5.6 Hz, 2H), 2.39 (s, 3H), 1.81 (dt, *J* = 8.2, 3.7 Hz, 1H), 1.45 (d, *J* = 6.7 Hz, 6H), 1.20 (dt, *J* = 6.5, 3.4 Hz, 2H), 0.88 (dq, *J* = 7.2, 4.0 Hz, 2H). ^13^C NMR (126 MHz, Chloroform-*d*) δ 169.34, 166.76, 164.41, 159.88, 158.24, 157.25, 135.92, 135.61, 132.78, 132.47, 132.32 (q, *J* = 38.5 Hz) ,132.17, 130.26, 128.35, 126.98, 126.85, 122.04 (q, *J* = 267.1 Hz), 119.39, 116.31 (q, *J* = 4.0 Hz), 115.37, 54.37, 49.66, 48.87, 45.78, 29.37, 24.18, 17.86, 14.18, 11.03. Condition A: HPLC purity 95.74 %, t_R_ = 5.690 min. HRMS (ESI) m/z: calculated for C_29_H_31_F_3_N_7_O [M + H]^+^: 550.2537 found: 550.2536.

*2-(4'-cyclopropyl-5,6'-dimethoxy-[2,5'-bipyrimidin]-4-yl)-6-(1-isopropyl-4-(trifluoromethyl)-1H-imidazol-2-yl)-1,2,3,4-tetrahydroisoquinoline (****13b****).* Compound **13b** was prepared in a manner similar to that described for compound **14a**: yield 65%; ^1^H NMR (500 MHz, Chloroform-*d*) δ 8.62 (s, 1H), 8.06 (s, 1H), 7.41 (t, *J* = 1.4 Hz, 2H), 7.31 (dd, *J* = 7.9, 1.8 Hz, 1H), 7. 23 (d, *J* = 7.9 Hz, 1H), 5.00 (s, 2H), 4.57 (p, *J* = 6.7 Hz, 1H), 4.10 (t, *J* = 5.8 Hz, 2H), 3.97 (s, 4 H), 3.94 (s, 3H), 3.04 (t, *J* = 5.8 Hz, 2H), 1.81 (td, *J* = 8.0, 4.0 Hz, 1H), 1.45 (d, *J* = 6.7 Hz, 7H), 1.20 (dt, *J* = 6.4, 3.3 Hz, 2H), 0.92 – 0.86 (m, 2H). ^13^C NMR (126 MHz, Chloroform-*d*) δ 169.00, 166.30, 156.76, 153.97, 153.56, 135.46, 135.31, 132.31 (q, *J* = 38.6 Hz), 129.71, 127.80, 126.38, 126.31, 122.04 (q, *J* = 267.1 Hz),118.43, 116.30 (q, *J* = 4.0 Hz), 56.05, 53.91, 48.46, 48.35, 44.55, 29.42, 28.72, 23.67, 13.69, 10.55. Condition A: HPLC purity 100.00 %, t_R_ = 5.822 min. HRMS (ESI) m/z: calculated for C_29_H_31_F_3_N_7_O_2_ [M + H]^+^: 566.2486 found: 566.2484.

*2-(4'-cyclopropyl-6'-methoxy-5-(trifluoromethyl)-[2,5'-bipyrimidin]-4-yl)-6-(1 isopropyl-4-(trifluoromethyl)-1H-imidazol-2-yl)-1,2,3,4-tetrahydroisoquinoline (****13c****).* Compound **13c** was prepared in a manner similar to that described for compound **14a**: yield 59%; ^1^H NMR (500 MHz, Chloroform-*d*) δ 8.66 (d, *J* = 3.3 Hz, 2H), 7.46 – 7.40 (m, 2H), 7.33 (t, *J* = 13.0 Hz, 2H), 5.03 (d, *J* = 27.8 Hz, 2H), 4.59 (p, *J* = 6.7 Hz, 1H), 4.15 (s, 2H), 3.91 (s, 3H), 3.01 (s, 2H), 1.53 (td, *J* = 8.2, 4.2 Hz, 1H), 1. 46 (d, *J* = 6.7 Hz, 6H), 1.29 – 1.24 (m, 2H), 0.92 (dd, *J* = 9.2, 5.2 Hz, 2H). ^13^C NMR (126 MHz, Chloroform-*d*) δ 168.33, 165.78, 161.64, 161.17, 156.28, 147.74, 135.50, 134.84, 131.85 (q, *J* = 38.7 Hz),129.52, 127.96, 126.52, 121.52 (q, *J* = 266.9 Hz), 121.50 (q, *J* = 266.9 Hz), 116.18 (q, J = 4.0 Hz), 115.82, 112.78, 53.80, 48.38, 45.91, 41.37, 29.42, 28.50, 24.59, 23.69, 14.06, 10.78, 10.34. Condition A: HPLC purity 100.00 %, t_R_ = 6.560 min. HRMS (ESI) m/z: calculated for C_29_H_28_F_6_N_7_O [M + H]^+^: 604.2254 found: 604.2256.

*4'-cyclopropyl-4-(6-(1-isopropyl-4-(trifluoromethyl)-1H-imidazol-2-yl)-3,4-dihydroisoquinolin-2(1H)-yl)-6'-methoxy-[2,5'-bipyrimidine]-5-carbonitrile (****13d****).* Compound **13d** was prepared in a manner similar to that described for compound **14a**: yield 62%; ^1^H NMR (500 MHz, Chloroform-*d*) δ 8.68 (s, 1H), 8.65 (d, *J* = 3.7 Hz, 1H), 7.45 (d, *J* = 5.1 Hz, 1H), 7.42 (d, *J* = 1.3 Hz, 1H), 7.40 – 7.27 (m, 2H), 5.05 (d, *J* = 31.6 Hz, 2H), 4.58 (p, *J* = 6.7 Hz, 1H), 4.25 – 4.07 (m, 2 H), 4.00 (s, 3H), 3.09 – 2.96 (m, 2H), 1.81 (s, 1H), 1.46 (d, *J* = 6.7 Hz, 6H), 1.36 – 1.29 (m, 2H), 0.97 (d, *J* = 6.6 Hz, 2H). ^13^C NMR (126 MHz, Chloroform-*d*) δ 169.31, 165.80, 165.26, 161.30, 160.19, 158.09, 147.61, 135.28, 134.38, 131.85 (q, *J* = 38.8 Hz), 129.55, 128.14, 126.70, 126.43, 121.48 (q, *J* = 267.0 Hz), 116.12 (q, *J* = 4.0 Hz), 115.88, 115.41, 97.43, 54.06, 48.41, 45.93, 41.57, 28.42, 24.58, 23.68, 13.81. Condition A: HPLC purity 100.00 %, t_R_ = 5.165 min. HRMS (ESI) m/z: calculated for C_29_H_28_F_3_N_8_O [M + H]^+^: 561.2333 found: 561.2332.

*2-(4-cyclopropyl-6-methoxypyrimidin-5-yl)-4-(6-(1-ethyl-4-(trifluoromethyl)-1H-imidazol-2-yl)-3,4-dihydroisoquinolin-2(1H)-yl)-5,6,7,8-tetrahydroquinazoline(****13e****).*

Compound **13e** was prepared in a manner similar to that described for compound **14a**: yield 67%; ^1^H NMR (600 MHz, Chloroform-*d*) δ 8.61 (s, 1H), 7.46 (s, 1H), 7.36 (s, 1H), 7.34 (d, *J* = 6.9 Hz, 1H), 7.23 (d, *J* = 7.9 Hz, 1H), 4.70 (s, 2H), 4.08 (q, *J* = 7.3 Hz, 2H), 3.93 (s, 3H), 3.72 (s, 2H), 3.09 (t, *J* = 5.8 Hz, 2H), 2.94 (s, 2H), 2.72 (d, *J* = 6.0 Hz, 2H), 1.94 (d, *J* = 6.1 Hz, 2H), 1.81 (q, *J* = 5.7 Hz, 3H), 1.44 (t, *J* = 7.3 Hz, 3H), 1.20 (q, *J* = 4.3, 3.7 Hz, 2H), 0.88 (dd, *J* = 7.8, 3.2 Hz, 2H). ^13^C NMR (151 MHz, Chloroform-*d*) δ 166.57, 148.46, 135.33, 131.88 (q, *J* = 39.0 Hz), 129.81, 127.83, 126.79, 126.32, 121.76 (q, *J* = 267.0 Hz), 119.36 (d, *J* = 4.2 Hz), 77.22, 77.01, 76.80, 54.08, 50.15, 46.22, 42.19, 29.69, 29.23, 26.78, 22.87, 16.26, 13.99, 10.77. Condition C: HPLC purity 100.00 %, t_R_ = 4.506 min. HRMS (ESI) m/z: calculated for C_31_H_32_F_3_N_7_O [M + H]^+^: 576.2693 found: 576.2693.

*2-(4-cyclopropyl-6-methoxypyrimidin-5-yl)-4-(6-(1-ethyl-4-(trifluoromethyl)-1H-imidazol-2-yl)-3,4-dihydroisoquinolin-2(1H)-yl)-6,7,8,9-tetrahydro-5H-cyclohepta[d]pyrimidine (****13f****).* Compound **13f** was prepared in a manner similar to that described for compound **14a**: yield 61%; ^1^H NMR (600 MHz, Chloroform-*d*) δ 8.61 (s, 1H), 7.45 (s, 1H), 7.36 (d, *J* = 9.2 Hz, 2H), 7.24 (d, *J* = 7.9 Hz, 1H), 4.96 (s, 2H), 4.12 (q, *J* = 7.2, 6.4 Hz, 1H), 4.07 (q, *J* = 7.9, 7.3 Hz, 2H), 4.02 (t, *J* = 5.9 Hz, 2H), 3.93 (d, *J* = 1.5 Hz, 3H), 3.20 (t, *J* = 7.3 Hz, 2H), 3.02 (t, *J* = 5.9 Hz, 2H), 2.98 (t, *J* = 7.9 Hz, 2H), 2.14 (t, *J* = 7.6 Hz, 2H), 2.04 (s, 1H), 1.82 (td, *J* = 8.4, 8.0, 3.9 Hz, 1H), 1.43 (t, *J* = 7.3 Hz, 3H), 1.26 (t, *J* = 6.3 Hz, 2H), 1.20 (s, 2H), 0.86 (t, *J* = 5.8 Hz, 2H). ^13^C NMR (151 MHz, Chloroform-*d*) δ 171.09, 169.45, 166.67, 165.15, 157.43, 148.33, 135.12, 131.77 (q, *J* = 38.9 Hz), 129.76, 128.00, 126.78, 126.45, 121.77 (q, *J* = 267.1 Hz), 119.47 (q, *J* = 3.8 Hz), 67.06, 60.34, 54.20, 50.96, 47.75, 42.20, 31.87, 28.93, 28.43, 26.96, 25.53, 20.98, 14.16, 14.06, 10.95. Condition C: HPLC purity 96.46 %, t_R_ = 6.349 min. HRMS (ESI) m/z: calculated for C_32_H_34_F_3_N_7_O [M + H]^+^: 590.2850 found: 590.2852.

*2-(2-(4-cyclopropyl-6-methoxypyrimidin-5-yl)-6,7-dihydro-5H-pyrrolo[3,4-d]pyrimidin-4-yl)-6-(1-isopropyl-4-(trifluoromethyl)-1H-imidazol-2-yl)-1,2,3,4-tetrahydroisoquinoline (****13g****).* Compound **13g** was prepared in a manner similar to that described for compound **14a**: yield 57%; ^1^H NMR (600 MHz, Chloroform-*d*) δ 8.55 (s, 1H), 7.35 (s, 2H), 7.26 (d, *J* = 7.8 Hz, 1H), 7.18 (s, 1H), 4.86 (s, 2H), 4.54 (s, 2H), 4.52 – 4.48 (m, 1H), 4.16 (s, 2H), 3.89 (t, *J* = 5.7 Hz, 2H), 3.86 (s, 3H), 2.95 (t, *J* = 5.5 Hz, 2H), 1.74 (dq, *J* = 8.4, 4.7, 4.2 Hz, 1H), 1.39 (d, *J* = 6.6 Hz, 6H), 1.14 (dq, *J* = 6.8, 4.0 Hz, 2H), 0.82 (q, *J* = 6.8, 5.0 Hz, 2H). ^13^C NMR (151 MHz, Chloroform-*d*) δ 171.18, 169.04, 166.48, 161.25, 158.77, 156.98, 147.94, 135.46, 135.29, 132.14 (d, *J* = 38.6 Hz), 129.88, 128.38, 126.90, 126.63, 121.78 (d, *J* = 267.1 Hz), 119.45, 116.13 (q, *J* = 4.0 Hz), 111.33, 54.09, 53.19, 52.39, 48.68, 47.25, 43.33, 29.08, 24.33, 13.98, 10.77. Condition C: HPLC purity 96.13 %, t_R_ = 4.133 min. HRMS (ESI) m/z: calculated for C_30_H_32_F_3_N_8_O [M + H]^+^: 577.2646 found: 577.2649.

*2-(4-cyclopropyl-6-methoxypyrimidin-5-yl)-4-(6-(1-isopropyl-4 (trifluoromethyl)-1H-imidazol-2-yl)-3,4-dihydroisoquinolin-2(1H)-yl)-5,7-dihydrofuro[3,4-d] pyrimidine (****13h****).* Compound **13h** was prepared in a manner similar to that described for compound **14a**: yield 62%; ^1^H NMR (600 MHz, Chloroform-*d*) δ 8.65 (s, 1H), 7.69 (dd, *J* = 2.7, 1.4 Hz, 1H), 7.49 (d, *J* = 1.8 Hz, 1H), 7.43 (d, *J* = 1.4 Hz, 1H), 7.37 (dd, *J* = 7.9, 1.7 Hz, 1H), 7.32 (d, *J* = 7.9 Hz, 1H), 6.92 (dd, *J* = 4.5, 1.5 Hz, 1H), 6.74 (dd, *J* = 4.6, 2.7 Hz, 1H), 5.18 (s, 2H), 4.65 – 4.55 (m, 1H), 4.26 (t, *J* = 5.9 Hz, 2H), 3.96 (s, 3H), 3.12 (t, *J* = 5.9 Hz, 2H), 1.99 (tt, *J* = 8.4, 4.6 Hz, 1H), 1.47 (d, *J* = 6. 7 Hz, 7H), 1.28 – 1.25 (m, 2H), 0.94 (dq, *J* = 7.0, 3.9 Hz, 2H). ^13^C NMR (151 MHz, Chloroform-*d*) δ 169.51, 166.67, 156.91, 154.70, 150.97, 147.64, 134.68, 131.76 (q, *J* = 38.9 Hz), 129.16, 128.41, 126.84, 126.53, 121.38 (q, *J* = 266.9 Hz), 119.58, 116.17 (q, *J* = 4.0 Hz), 113.37, 110.49, 104.41, 54.01, 48.45, 47.41, 29.46, 28.79, 23.73, 13.96, 10.80. Condition B: HPLC purity 99.83 %, t_R_ = 10.272 min. HRMS (ESI) m/z: calculated for C_30_H_31_F_3_N_7_O_2_ [M + H]^+^: 578.2486 found: 578.2488.

*2-(4-cyclopropyl-6-methoxypyrimidin-5-yl)-4-(6-(1-ethyl-4-(trifluoromethyl)-1H-imidazol-2-yl)-3,4-dihydroisoquinolin-2(1H)-yl)-5,7-dihydro-6H-pyrrolo[2,3-d]pyrimidin-6-one (****13i****).* Compound **13i** was prepared in a manner similar to that described for compound **14a**: yield 56%; ^1^H NMR (600 MHz, Chloroform-*d*) δ 8.64 (s, 1H), 8.24 (s, 1H), 7.44 (s, 1H), 7.35 (d, *J* = 5.2 Hz, 2H), 4.95 (s, 2H), 4.07 (q, *J* = 7.2 Hz, 3H), 3.96 (s, 2H), 3.10 (q, *J* = 7.3 Hz, 2H), 2.96 (s, 2H), 2.04 (dq, *J* = 8.3, 4.5, 4.1 Hz, 1H), 1.72 (s, 3H), 1.42 (dt, *J* = 17.8, 7.3 Hz, 6H). ^13^C NMR (151 MHz, CDCl_3_) δ 174.64, 169.08, 164.76, 163.54, 159.70, 156.76, 153.61, 147.47, 134.84 (d, *J* = 27.0 Hz), 128.55, 126.74, 125.69, 125.44, 121.79 (d, *J* = 267.1 Hz), 118.34 (d, *J* = 4.0 Hz), 114.96, 104.25, 76.22, 76.00, 75.79, 53.15, 45.29, 44.84, 41.17, 40.54, 32.60, 27.82, 15.23, 13.10, 10.17, 7.60. Condition C: HPLC purity 97.23 %, t_R_ = 5.365 min. HRMS (ESI) m/z: calculated for C_29_H_27_F_3_N_8_O_2_ [M + H]^+^: 577.2282 found: 577.2280.

*2-(4-cyclopropyl-6-methoxypyrimidin-5-yl)-4-(6-(1-ethyl-4-(trifluoromethyl)-1H-imidazol-2-yl)-3,4-dihydroisoquinolin-2(1H)-yl)-5,5,7-trimethyl-5,7-dihydro-6H-pyrrolo[2,3-d]pyrimidin-6-one (****13j****).* Compound **13j** was prepared in a manner similar to that described for compound **14a**: yield 58%; ^1^H NMR (600 MHz, Chloroform-*d*) δ 8.59 (s, 1H), 7.39 (s, 1H), 7.29 (d, *J* = 7.9 Hz, 2H), 7.23 (d, *J* = 7.9 Hz, 1H), 4.93 (s, 2H), 4.06 (d, *J* = 6.1 Hz, 2H), 4.02 (d, *J* = 8.0 Hz, 2H), 3.83 (s, 3H), 3.22 (s, 3H), 2.92 (s, 2H), 1.57 (s, 1H), 1.37 (s, 3H), 1.06 (d, *J* = 11.4 Hz, 6H), 0.88 (d, *J* = 7.8 Hz, 2H), 0.78 (d, *J* = 6.6 Hz, 2H). ^13^C NMR (151 MHz, Chloroform-*d*) δ 181.92, 169.13, 166.08, 164.18, 160.67, 157.78, 148.52, 136.03, 135.91, 131.89 (d, *J* = 38.9 Hz), 129.57, 127.72, 126.74, 126.40, 121.79 (d, *J* = 266.6 Hz), 119.31 (d, *J* = 3.7 Hz), 113.64, 77.21, 77.00, 76.79, 53.79, 46.41, 43.34, 42.18, 28.92, 25.36, 23.46, 22.74, 16.23, 14.36, 11.27, 10.99. Condition C: HPLC purity 96.18 %, t_R_ = 5.775 min. HRMS (ESI) m/z: calculated for C_32_H_33_F_3_N_8_O_2_ [M + H]^+^: 619.2751 found: 619.2752.

*2-(2-(4-cyclopropyl-6-methoxypyrimidin-5-yl)-5H-pyrrolo[3,2-d]pyrimidin-4-yl)-6-(1-isopropyl-4-(trifluoromethyl)-1H-imidazol-2-yl)-1,2,3,4 tetrahydroisoquinoline (****13k****).* Compound **13k** was prepared in a manner similar to that described for compound **14a**: yield 61%; ^1^H NMR (600 MHz, Chloroform-*d*) δ 10.54 (s, 1H), 8.60 (s, 1H), 7.44 (d, *J* = 1.4 Hz, 1H), 7.31 (d, *J* = 1.7 Hz, 1H), 7.27 (s, 1H), 7.24 – 7.16 (m, 2H), 6.54 (d, *J* = 3.1 Hz, 1H), 5.01 (s, 2H), 4.55 (p, *J* = 6.7 Hz, 1H), 4.06 (t, *J* = 5.9 Hz, 2H), 3.84 (s, 3H), 2.93 (t, *J* = 5.9 Hz, 2H), 1.84 (td, *J* = 8.2, 4.2Hz, 1H), 1.45 (d, *J* = 6.7 Hz, 6H), 1.17 (dt, *J* = 6.5, 3.3 Hz, 2H), 0.85 (m, 2H). ^13^C NMR (151 MHz, Chloroform-*d*) δ 168.90, 166.09, 156.40, 150.41, 147.74, 135.58, 135.09, 131.54 (q, *J* = 38.8 Hz),128.87, 128.61, 127.63, 126.40 (d, *J* = 7.2 Hz), 121.37 (q, *J* = 267.1 Hz), 115.86 (q, J = 4.0 Hz), 113.02, 102.12, 53.61, 48.37, 47.16, 43.40, 29.29, 28.60, 23.53, 13.63, 11.04, 10.48. Condition B: HPLC purity 99.55 %, t_R_ = 10.361 min. HRMS (ESI) m/z: calculated for C_30_H_30_F_3_N_8_O [M + H]^+^: 575.2489 found: 575.2485.

*2-(2-(4-cyclopropyl-6-methoxypyrimidin-5-yl)pyrrolo[2,1-f][1,2,4]triazin-4-yl)-6-(1-isopropyl-4-(trifluoromethyl)-1H-imidazol-2-yl)-1,2,3,4-tetrahydroisoquinoline (****13l****).* Compound **13l** was prepared in a manner similar to that described for compound **14a**: yield 60%; ^1^H NMR (600 MHz, Chloroform-*d*) δ 8.65 (s, 1H), 7.69 (dd, *J* = 2.7, 1.4 Hz, 1H), 7.49 (d, *J* = 1.8 Hz, 1H), 7.43 (d, *J* = 1.4 Hz, 1H), 7.37 (dd, *J* = 7.9, 1.7 Hz, 1H), 7.32 (d, *J* = 7.9 Hz, 1H), 6.92 (dd, *J* = 4.5, 1.5 Hz, 1H), 6.74 (dd, *J* = 4.6, 2.7 Hz, 1H), 5.18 (s, 2H), 4.65 – 4.55 (m, 1H), 4.26 (t, *J* = 5.9 Hz, 2H), 3.96 (s, 3H), 3.12 (t, *J* = 5.9 Hz, 2H), 1.99 (tt, *J* = 8.4, 4.6 Hz, 1H), 1.47 (d, *J* = 6. 7 Hz, 7H), 1.28 – 1.25 (m, 2H), 0.94 (dq, *J* = 7.0, 3.9 Hz, 2H). ^13^C NMR (151 MHz, Chloroform-*d*) δ 169.51, 166.67, 156.91, 154.70, 150.97, 147.64, 134.68, 131.76 (q, *J* = 38.9 Hz), 129.16, 128.41, 126.84, 126.53, 121.38 (q, *J* = 266.9 Hz), 119.58, 115.78 (q, *J* = 4.0 Hz), 113.37, 110.49, 104.41, 54.01, 48.45, 47.41, 29.46, 28.79, 23.73, 13.96, 10.80. Condition A: HPLC purity 100.00 %, t_R_ = 8.401 min. HRMS (ESI) m/z: calculated for C_30_H_30_F_3_N_8_O [M + H]^+^: 575.2489 found: 575.2488.

*2-(4-cyclopropyl-6-methoxypyrimidin-5-yl)-4-(6-(1-isopropyl-4-(trifluoromethyl)-1H-imidazol-2-yl)-3,4-dihydroisoquinolin-2(1H)-yl)pyrido[3,2-d] pyrimidine (****13m****).* Compound **13m** was prepared in a manner similar to that described for compound **14a**: yield 65%; ^1^H NMR (500 MHz, Chloroform-*d*) δ 8.84 (dd, *J* = 4.1, 1.8 Hz, 1H), 8.67 (s, 1H), 8.21 (d, *J* = 8.5 Hz, 1H), 7.68 (dd, *J* = 8.5, 4.1 Hz, 1H), 7.47 (d, *J* = 1.6 Hz, 1H), 7.43 (d, *J* = 1.3 Hz, 1H), 7.38 – 7.31 (m, 2H), 5.90 – 5.44 (m, 2H), 4.95 – 4.46 (m, 3H), 3.95 (s, 3H), 3.19 (t, *J* = 5.7 Hz, 2H), 1.94 – 1.77 (m, 1H), 1.47 (d, *J* = 6.7 Hz, 6H), 1.24 (dt, *J* = 6.5, 3.3 Hz, 2H), 0.90 (dt, *J* = 8.0, 3.4 Hz, 2H). ^13^C NMR (126 MHz, Chloroform-*d*) δ 169.01, 166.48, 159.45, 158.89, 157.02, 148.10, 147.69, 146.97, 136.11, 135.91, 132.83, 132.25 – 132.05 (m), 131.94, 129.88, 128.19, 127.08, 126.71, 122.89, 120.77, 119.76, 116.09 (q, *J* = 4.0 Hz), 54.11, 53.44, 48.65, 45.96, 31.51, 30.14, 29.70, 23.95, 14.10, 10. 82. Condition A: HPLC purity 98.90 %, t_R_ = 12.103 min. HRMS (ESI) m/z: calculated for C_31_H_30_F_3_N_8_O [M + H]^+^: 587.2489 found: 587.2491.

*2-(4-cyclopropyl-6-methoxypyrimidin-5-yl)-4-(6-(1-isopropyl-4-(trifluoromethyl)-1H-imidazol-2-yl)-3,4-dihydroisoquinolin-2(1H)-yl)furo[3,2-d]pyrimidine (****13n****).* Compound **13n** was prepared in a manner similar to that described for compound **14a**: yield 66%; ^1^H NMR (600 MHz, Chloroform-*d*) δ 8.56 (s, 1H), 7.77 (d, *J* = 2.1 Hz, 1H), 7.36 (dd, *J* = 12.7, 1.6 Hz, 2H), 7.28 (dd, *J* = 7.8, 1.8 Hz, 1H), 7.25 (d, *J* = 7.9 Hz, 1H), 6.86 (d, *J* = 2.2 Hz, 1H), 5.13 (s, 2H), 4.51 (p, *J* = 6.7 Hz, 1H), 4.26 (t, *J* = 5.9 Hz, 2H), 3.85 (s, 3H), 3.02 (t, *J* = 5.9 Hz, 2H), 1.74 (tt, *J* = 8.1, 4.7 Hz, 1H), 1.39 (d, *J* = 6.7 Hz, 6H), 1.16 – 1.09 (m, 2H), 0.83 – 0.77 (m, 2H). ^13^C NMR (201 MHz, CDCl3) δ 169.05, 166.50, 157.02, 156.86, 148.42, 148.16, 148.00, 135.49, 135.07, 133.31, 132.09 (q, *J* = 9.9 Hz), 131.98, 129.94, 128.55, 128.49, 128.28, 126.86, 126.71, 121.8 (q, *J* = 266.9 Hz), 119.75, 116.15 (q, *J* = 3.8 Hz), 108.13, 60.40, 54.13, 48.68, 23.94, 14.01, 10.78. Condition A: HPLC purity 96.34 %, t_R_ = 7.078 min. HRMS (ESI) m/z: calculated for C_30_H_29_F_3_N_7_O_2_ [M + H]^+^: 576.2329 found: 576.2326.

*2-(4-cyclopropyl-6-methoxypyrimidin-5-yl)-4-(6-(1-isopropyl-4-(trifluoromethyl)-1H-imidazol-2-yl)-3,4-dihydroisoquinolin-2(1H)-yl)thieno[2,3-d]pyrimidine (****13o****).* Compound **13o** was prepared in a manner similar to that described for compound **14a**: yield 67%; ^1^H NMR (500 MHz, Chloroform-*d*) δ 8.65 (s, 1H), 7.55 (d, *J* = 6.1 Hz, 1H), 7.48 (d, *J* = 1.8 Hz, 1H), 7.42 (d, *J* = 1.3 Hz, 1H), 7.36 (dd, *J* = 7.0, 3.2 Hz, 2H), 7.31 (d, *J* = 7.8 Hz, 1H), 5.11 (s, 2H), 4.59 (hept, *J* = 6.7 Hz, 1H), 4.19 (t, *J* = 5.8 Hz, 2H), 3.94 (s, 3H), 3.13 (t, *J* = 5.8 Hz, 2H), 1.87 (tt, *J* = 8.3, 4.6 Hz, 1H), 1.46 (d, *J* = 6.6 Hz, 6H), 1.27 – 1.22 (m, 2H), 0.90 (dt, *J* = 6.3, 3.2 Hz, 2H). ^13^C NMR (126 MHz, Chloroform-*d*) δ 168.86, 166.35, 158.05, 156.67, 156.41, 147.64, 135.74, 135.00, 131.88 (q, *J* = 38.8 Hz),129.29, 128.28, 126.47, 122.57, 121.84, 121.52 (q, *J* = 265.2 Hz),120.46, 119.17, 115.88 (q, *J* = 3.8 Hz), 114.53, 53.89, 48.38, 44.88, 29.41, 28.78, 23.68, 22.34, 19.16, 13.78, 11.15, 10.64. Condition A: HPLC purity 95.37 %, t_R_ = 13.213 min. HRMS (ESI) m/z: calculated for C_30_H_29_F_3_N_7_OS [M + H]^+^: 592.2101 found: 592.2101.

*5-(4-cyclopropyl-6-methoxypyrimidin-5-yl)-7-(6-(1-isopropyl-4-(trifluoromethyl)-1H-imidazol-2-yl)-3,4-dihydroisoquinolin-2(1H)-yl)thiazolo[5,4-d]pyrimidine (****13p****).* Compound **13p** was prepared in a manner similar to that described for compound **14a**: yield 64%; ^1^H NMR (500 MHz, Chloroform-*d*) δ 8.86 (s, 1H), 8.67 (s, 1H), 7.46 (s, 1H), 7.43 (d, *J* = 1.3 Hz, 1H), 7.39 – 7.31 (m, 2H), 5.48 (s, 2H), 4.95 – 4.37 (m, *J* = 7.1 Hz, 3H), 3.95 (s, 3H), 3.12 (t, *J* = 5.9 Hz, 2H), 1.87 (tt, *J* = 8.4, 4.6 Hz, 1H), 1.47 (d, *J* = 6.6 Hz, 6H), 1.25 (dd, *J* = 4.6, 2.7 Hz, 2H), 0.92 (dd, *J* = 8.1, 2.9 Hz, 2H). ^13^C NMR (126 MHz, Chloroform-*d*) δ 169.06, 166.51, 165.13, 158.02, 157.03, 155.20, 148.33, 148.26, 135.64, 135.44, 132.18 (q, *J* = 4.7 Hz), 131.94, 129.93, 129.39, 128.26, 126.80, 126.72, 122.03 (q, *J* = 266.8 Hz), 119.33, 116.12 (q, *J* = 4.0 Hz), 54.20, 48.66, 31.51, 30.14, 29.70, 28.95, 23.95, 14.08, 10.93. Condition A: HPLC purity 99.52 %, t_R_ = 8.752 min. HRMS (ESI) m/z: calculated for C_29_H_28_F_3_N_8_OS [M + H]^+^: 593.2053 found: 593.2056.

*4-(6-(1-cyclopropyl-4-(trifluoromethyl)-1H-imidazol-2-yl)-3,4-dihydroisoquinolin-2(1H)-yl)-2-(4-cyclopropyl-6-methoxypyrimidin-5-yl)furo[3,2-d]pyrimidine (****14b****)*. Compound **14b** was prepared in a manner similar to that described for compound **14a**: yield 62%; ^1^H NMR (600 MHz, Chloroform-*d*) δ 8.64 (s, 1H), 7.84 (d, *J* = 2.1 Hz, 1H), 7.70 (d, *J* = 1.7 Hz, 1H), 7.68 (dd, *J* = 7.9, 1.9 Hz, 1H), 7.34 (t, *J* = 1.3 Hz, 1H), 7.30 (d, *J* = 8.0 Hz, 1H), 6.96 (s, 1H), 5.21 (s, 2H), 4.34 (t, *J* = 5.9 Hz, 2H), 3.93 (s, 3H), 3.51 (tt, *J* = 7.3, 3.9 Hz, 1H), 3.09 (t, *J* = 5.9 Hz, 2H), 1.82 (tt, J = 8.3, 4.7 Hz, 1H), 1.21 (dt, *J* = 6.5, 3.3 Hz, 2H), 1.09 – 1.05 (m, 2H), 0.88 (ddd, *J* = 8.2, 5.3, 2.3 Hz, 4H). ^13^C NMR (151 MHz, Chloroform-*d*) δ 168.88, 166.26, 156.70, 149.09, 148.18, 147.96, 134.81, 134.42, 133.02, 131.88, 131.82, 131.72, 131.70, 130.81 (q, *J* = 38.8 Hz), 128.95, 128.31, 128.23, 128.04, 126.14, 126.11, 121.40 (q, *J* = 266.8 Hz), 121.15 (q, *J* = 4.0 Hz), 107.80, 53.94, 46.73, 42.93, 29.27, 28.71, 13.79, 10.60, 8.54. Condition A: HPLC purity 98.39 %, t_R_ = 14.690 min. HRMS (ESI) m/z: calculated for C_30_H_27_F_3_N_7_O_2_ [M + H]^+^: 574.2173 found: 574.2172.

*4-(6-(1-ethyl-4-(trifluoromethyl)-1H-imidazol-2-yl)-3,4-dihydroisoquinolin-2(1H)-yl)-2-(pyrimidin-5-yl)furo[3,2-d]pyrimidine (****14c****)*. Compound **14c** was prepared in a manner similar to that described for compound **14a**: yield 69%; ^1^H NMR (600 MHz, Chloroform-*d*) δ 9.61 (s, 2H), 9.18 (s, 1H), 7.75 (d, *J* = 2.2 Hz, 1H), 7.40 (d, *J* = 1.8 Hz, 1H), 7.33 (dd, *J* = 7.9, 1.8 Hz, 1H), 7.29 – 7.24 (m, 2H), 6.87 (d, *J* = 2.2 Hz, 1H), 5.18 (s, 2H), 4.29 (t, *J* = 5.9 Hz, 2H), 3.99 (q, *J* = 7.4 Hz, 2H), 3.03 (t, *J* = 5.9 Hz, 2H), 1.35 (t, *J* = 7.3 Hz, 3H). ^13^C NMR (126 MHz, Chloroform-*d*) δ 158.54, 156.07, 148.18, 147.76, 147.74, 134.84, 134.21, 133.39, 131.39 (q, *J* = 38.7 Hz), 131.04, 129.25, 127.74, 126.27, 121.25 (q, *J* = 267.0 Hz), 118.99 (q, *J* = 4.0 Hz), 107.53, 41.74, 29.21, 28.44, 15.78. Condition A: HPLC purity 98.89 %, t_R_ = 3.669 min. HRMS (ESI) m/z: calculated for C_25_H_21_F_3_N_7_O [M + H]^+^: 492.1754 found: 492.1758.

*4-(6-(1-ethyl-4-(trifluoromethyl)-1H-imidazol-2-yl)-3,4-dihydroisoquinolin-2(1H)-yl)-2-(4-methoxypyrimidin-5-yl)furo[3,2-d]pyrimidine (****14d****)*. Compound **14d** was prepared in a manner similar to that described for compound **14a**: yield 67%; ^1^H NMR (600 MHz, Chloroform-*d*) δ 9.03 (s, 1H), 8.84 (s, 1H), 7.84 (d, *J* = 2.2 Hz, 1H), 7.49 (d, *J* = 1.8 Hz, 1H), 7.41 (dd, *J* = 7.9, 1.8 Hz, 1H), 7.37 (q, *J* = 1.2 Hz, 1H), 7.33 (d, *J* = 7.9 Hz, 1H), 6.99 (d, *J* = 2.1 Hz, 1H), 5.24 (s, 2H), 4.36 (t, *J* = 5.8 Hz, 2H), 4.12 (s, 3H), 4.09 (q, *J* = 7.3 Hz, 2H), 3.11 (t, *J* = 5.9 Hz, 2H), 1.45 (t, *J* = 7.3 Hz, 3H). ^13^C NMR (126 MHz, Chloroform-*d*) δ 166.80, 158.37, 157.96, 156.04, 151.98, 148.29, 148.17, 135.37, 134.96, 133.42, 131.80 (q, *J* = 38.8 Hz), 129.71, 128.10, 126.70, 126.67, 121.78 (q, *J* = 266.8 Hz), 121.10, 119.48 (q, *J* = 4.0 Hz), 108.20, 60.38, 54.40, 46.89, 43.06, 42.22, 29.68, 28.90, 16.25. Condition A: HPLC purity 100.00 %, t_R_ = 13.959 min. HRMS (ESI) m/z: calculated for C_26_H_23_F_3_N_7_O_2_ [M + H]^+^: 522.1860 found: 522.1863.

*4-(6-(1-ethyl-4-(trifluoromethyl)-1H-imidazol-2-yl)-3,4-dihydroisoquinolin-2(1H)-yl)-2-(2-fluoropyridin-3-yl)furo[3,2-d]pyrimidine (****14e****)*. Compound **14e** was prepared in a manner similar to that described for compound **14a**: yield 64%; ^1^H NMR (500 MHz, Chloroform-*d*) δ 8.54 (ddd, *J* = 9.5, 7.5, 2.0 Hz, 1H), 8.27 (ddd, *J* = 4.8, 2.0, 1.1 Hz, 1H), 7.83 (d, *J* = 2.2 Hz, 1H), 7.47 (d, *J* = 1.8 Hz, 1H), 7.40 (dd, *J* = 7.8, 1.8 Hz, 1H), 7.37 – 7.32 (m, 2H), 7.30 (ddd, *J* = 7.5, 4.8, 1.6 Hz, 1H), 6.94 (d, *J* = 2.2 Hz, 1H), 5.25 (s, 2H), 4.36 (t, *J* = 5.9 Hz, 2H), 4.07 (q, *J* = 7.3 Hz, 2H), 3.09 (t, *J* = 5.9 Hz, 2H), 1.43 (t, *J* = 7.3 Hz, 3H). ^13^C NMR (126 MHz, Chloroform-*d*) δ 161.85, 159.90, 155.69, 155.62, 151.63, 147.90, 147.61, 147.48, 147.36, 141.70, 141.68, 134.95, 134.55, 132.91, 131.37 (q, *J* = 38.8 Hz), 129.23, 127.63, 126.31, 126.21, 121.33 (q, *J* = 266.9 Hz), 122.15, 121.97, 120.89, 120.85, 119.00 (q, *J* = 4.0 Hz), 107.67, 46.45, 42.57, 41.77, 29.24, 28.46, 15.80. Condition A: HPLC purity 95.05 %, t_R_ = 3.307 min. HRMS (ESI) m/z: calculated for C_26_H_21_F_4_N_6_O [M + H]^+^: 509.1707 found: 509.1710.

*4-(6-(1-ethyl-4-(trifluoromethyl)-1H-imidazol-2-yl)-3,4-dihydroisoquinolin-2(1H)-yl)-2-(2-methylpyrimidin-5-yl)furo[3,2-d]pyrimidine (****14f****)*. Compound **14f** was prepared in a manner similar to that described for compound **14a**: yield 63%; ^1^H NMR (600 MHz, Chloroform-*d*) δ 9.57 (s, 2H), 7.81 (d, *J* = 2.2 Hz, 1H), 7.47 (d, *J* = 1.8 Hz, 1H), 7.40 (dd, *J* = 7.9, 1.8 Hz, 1H), 7.36 (d, *J* = 1.4 Hz, 1H), 7.33 (d, *J* = 7.9 Hz, 1H), 6.92 (d, *J* = 2.2 Hz, 1H), 5.23 (s, 2H), 4.35 (t, *J* = 5.9 Hz, 2H), 4.09 (dq, *J* = 14.7, 7.2 Hz, 2H), 3.09 (t, *J* = 6.0 Hz, 2H), 2.80 (s, 3H), 1.43 (t, *J* = 7.3 Hz, 3H). ^13^C NMR (126 MHz, Chloroform-*d*) δ 168.09, 156.21, 155.00, 151.55, 148.06, 147.82, 147.73, 134.90, 134.35, 133.37, 131.42 (q, *J* = 38.7 Hz), 129.28, 128.18, 127.73, 126.28, 121.29 (q, *J* = 267.0 Hz), 119. 90 (q, *J* = 4.0 Hz) 107.57, 46.46, 42.71, 41.77, 28.46, 25.52, 15.82. Condition D: HPLC purity 97.36 %, t_R_ = 7.098 min. HRMS (ESI) m/z: calculated for C_26_H_23_F_3_N_7_O [M + H]^+^: 506.1911 found: 506.1911.

*5-(4-(6-(1-ethyl-4-(trifluoromethyl)-1H-imidazol-2-yl)-3,4-dihydroisoquinolin-2(1H)-yl)furo[3,2-d]pyrimidin-2-yl)-N,N-dimethylpyrimidin-2-amine (****14g****)*. Compound **14g** was prepared in a manner similar to that described for compound **14a**: yield 63%; ^1^H NMR (600 MHz, Chloroform-*d*) δ 9.31 (s, 2H), 7.75 (d, *J* = 2.2 Hz, 1H), 7.45 (d, *J* = 1.8 Hz, 1H), 7.40 (dd, *J* = 7.8, 1.8 Hz, 1H), 7.37 – 7.35 (m, 1H), 7.33 – 7.28 (m, 1H), 6.85 (d, *J* = 2.2 Hz, 1H), 5.19 (s, 2H), 4.31 (t, *J* = 5.9 Hz, 2H), 4.08 (q, *J* = 7.3 Hz, 2H), 3.27 (s, 6H), 3.07 (t, *J* = 5.9 Hz, 2H), 1.43 (t, *J* = 7.3 Hz, 3H). ^13^C NMR (126 MHz, Chloroform-*d*) δ 161.80, 157.36, 156.39, 151.55, 147.90, 147.54, 147.43, 134.98, 134.66, 132.87, 131.26 (q, *J* = 38.7 Hz), 129.16, 127.50, 126.26, 126.18, 121.34 (q, *J* = 266.9 Hz), 119.30, 119.01 (q, *J* = 4.0 Hz), 107.39, 46.28, 42.51, 41.76, 36.86, 29.22, 28.46, 15.76. Condition A: HPLC purity 98.18 %, t_R_ = 4.067 min. HRMS (ESI) m/z: calculated for C_27_H_26_F_3_N_8_O [M + H]^+^: 535.2176 found: 535.2173.

*2-(2-cyclopropylpyrimidin-5-yl)-4-(6-(1-ethyl-4-(trifluoromethyl)-1H-imidazol-2-yl)-3,4-dihydroisoquinolin-2(1H)-yl)furo[3,2-d]pyrimidine (****14h****)*. Compound **14h** was prepared in a manner similar to that described for compound **14a**: yield 61%; ^1^H NMR (600 MHz, Chloroform-*d*) δ 9.51 (s, 1H), 7.80 (d, *J* = 2.2 Hz, 1H), 7.47 (d, *J* = 1.9 Hz, 1H), 7.40 (dd, *J* = 7.9, 1.8 Hz, 1H), 7.36 (d, *J* = 1.4 Hz, 1H), 7.32 (d, *J* = 7.9 Hz, 1H), 6.91 (d, *J* = 2.2 Hz, 1H), 5.23 (s, 2H), 4.34 (t, *J* = 5.9 Hz, 2H), 4.08 (q, *J* = 7.3 Hz, 2H), 3.09 (t, *J* = 5.9 Hz, 2H), 2.34 (tt, *J* = 8.4, 4.7 Hz, 1H), 1.43 (t, *J* = 7.3 Hz, 3H), 1.22 (dd, *J* = 4.6, 2.7 Hz, 2H), 1.16 – 1.10 (m, 2H). ^13^C NMR (126 MHz, Chloroform-*d*) δ 172.11, 156.03, 155.26, 151.68, 147.95, 147.86, 147.65, 134.92, 134.45, 133.30, 132.36, 131.92, 131.51, 131.14 (q, *J* = 38.2 Hz), 129.25, 127.96, 127.63, 126.27, 121.30 (q, *J* = 267.0 Hz), 119.02 (q, *J* = 4.0 Hz), 107.57, 41.78, 29.24, 28.46, 17.85, 15.80, 15.71, 10.87. Condition A: HPLC purity 96.61 %, t_R_ = 4.153 min. HRMS (ESI) m/z: calculated for C_28_H_25_F_3_N_7_O [M + H]^+^: 532.2067 found: 532.2069.

*4-(6-(1-ethyl-4-(trifluoromethyl)-1H-imidazol-2-yl)-3,4-dihydroisoquinolin-2(1H)-yl)-2-(2-methoxy-4-methylpyrimidin-5-yl)furo[3,2-d]pyrimidine (****14i****)*. Compound **14i** was prepared in a manner similar to that described for compound **14a**: yield 64%; ^1^H NMR (600 MHz, Chloroform-*d*) δ 8.97 (s, 1H), 7.77 (d, *J* = 2.2 Hz, 1H), 7.43 – 7.40 (m, 1H), 7.34 (dd, *J* = 7.9, 1.8 Hz, 1H), 7.30 (d, *J* = 1.3 Hz, 1H), 7.26 (d, *J* = 7.9 Hz, 1H), 6.86 (d, *J* = 2.2 Hz, 1H), 5.16 (s, 2H), 4.28 (t, *J* = 5.9 Hz, 2H), 4.01 (d, *J* = 15.6 Hz, 5H), 3.03 (t, *J* = 5.9 Hz, 2H), 2.75 (s, 3H), 1.38 (t, *J* = 7.3 Hz, 3H). ^13^C NMR (126 MHz, Chloroform-*d*) δ 168.98, 164.69, 160.62, 157.94, 151.68, 148.44, 148.28, 148.06, 135.35, 134.84, 133.11, 131.86 (q, *J* = 38.7 Hz), 129.73, 128.17, 126.73, 126.31, 121.75 (q, *J* = 266.8 Hz), 119.48 (q, *J* = 4.0 Hz), 107.96, 54.91, 47.01, 43.16, 42.24, 29.70, 28.94, 24.38, 16.27. Condition A: HPLC purity 98.82 %, t_R_ = 16.133 min. HRMS (ESI) m/z: calculated for C_28_H_27_F_3_N_7_O [M + H]^+^: 536.2016 found: 536.2018.

*2-(4,6-dimethylpyrimidin-5-yl)-4-(6-(1-ethyl-4-(trifluoromethyl)-1H-imidazol-2-yl)-3,4-dihydroisoquinolin-2(1H)-yl)furo[3,2-d]pyrimidine (****14j****)*. Compound **14j** was prepared in a manner similar to that described for compound **14a**: yield 65%; ^1^H NMR (600 MHz, Chloroform-*d*) δ 8.98 (s, 1H), 7.87 (d, *J* = 2.1 Hz, 1H), 7.51 – 7.48 (m, 1H), 7.40 (dd, *J* = 8.0, 1.8 Hz, 1H), 7.37 (q, *J* = 1.2 Hz, 1H), 7.31 (d, *J* = 8.0 Hz, 1H), 6.92 (d, *J* = 2.1 Hz, 1H), 5.19 (s, 2H), 4.34 (t, *J* = 6.0 Hz, 2H), 4.09 (q, *J* = 7.3 Hz, 2H), 3.10 (t, *J* = 5.9 Hz, 2H), 2.41 (s, 6H), 1.44 (t, *J* = 7.3 Hz, 3H). ^13^C NMR (126 MHz, Chloroform-*d*) δ 164.45, 158.56, 156.50, 151.53, 148.56, 148.34, 148.23, 135.30, 134.68, 133.09, 132.90, 131.90 (q, *J* = 39.0 Hz), 129.74, 129.67, 128.24, 126.74, 126.72, 121.73 (q, *J* = 267.0 Hz), 119.50 (q, *J* = 4.0 Hz), 107.96, 42.24, 29.70, 28.93, 22.80, 22.59, 22.46, 16.28, 13.62. Condition A: HPLC purity 98.41 %, t_R_ = 7.728 min. HRMS (ESI) m/z: calculated for C_27_H_25_F_3_N_7_O [M + H]^+^: 520.1994 found: 520.2071.

*2-(1-cyclopropyl-1H-pyrazol-5-yl)-4-(6-(1-ethyl-4-(trifluoromethyl)-1H-imidazol-2-yl)-3,4-dihydroisoquinolin-2(1H)-yl)furo[3,2-d]pyrimidine (****14k****)*. Compound **14k** was prepared in a manner similar to that described for compound **14a**: yield 69%; ^1^H NMR (600 MHz, Chloroform-*d*) δ 7.81 (d, *J* = 2.1 Hz, 1H), 7.45 (d, *J* = 1.6 Hz, 1H), 7.43 (d, *J* = 1.9 Hz, 1H), 7.39 (dd, *J* = 7.9, 1.8 Hz, 1H), 7.35 (t, *J* = 1.2 Hz, 1H), 7.29 (d, *J* = 7.9 Hz, 1H), 6.89 (dd, *J* = 4.3, 2.0 Hz, 2H), 5.21 (s, 2H), 4.59 (tt, *J* = 7.5, 4.0 Hz, 1H), 4.32 (t, *J* = 5.9 Hz, 2H), 4.06 (q, *J* = 7.3 Hz, 2H), 3.07 (t, *J* = 5.9 Hz, 2H), 1.42 (t, *J* = 7.3 Hz, 3H), 1.29 – 1.19 (m, 2H), 1.00 – 0.92 (m, 2H). ^13^C NMR (126 MHz, Chloroform-*d*) δ 152.25, 150.80, 150.53, 147.34, 147.26, 146.94, 141.61, 136.41, 134.34, 133.91, 132.02, 130.76 (q, *J* = 38.8 Hz), 128.68, 127.11, 125.69, 125.63, 120.75 (q, *J* = 266.9 Hz), 118.48 (q, *J* = 4.0 Hz), 107.86, 107.05, 45.94, 42.04, 41.20, 32.40, 28.66, 27.87, 15.22, 6.48. Condition A: HPLC purity 98.22 %, t_R_ = 5.393 min. HRMS (ESI) m/z: calculated for C_27_H_25_F_3_N_7_O [M + H]^+^: 520.2067 found: 520.2065.

*2-(1-(cyclopropylmethyl)-1H-pyrazol-5-yl)-4-(6-(1-ethyl-4-(trifluoromethyl)-1H-imidazol-2-yl)-3,4-dihydroisoquinolin-2(1H)-yl)furo[3,2-d]pyrimidine (****14l****)*. Compound **14l** was prepared in a manner similar to that described for compound **14a**: yield 68%; ^1^H NMR (500 MHz, Chloroform-*d*) δ 7.84 (d, *J* = 2.1 Hz, 1H), 7.56 (d, *J* = 1.9 Hz, 1H), 7.51 (d, *J* = 1.8 Hz, 1H), 7.44 (dd, *J* = 7.9, 1.8 Hz, 1H), 7.39 (q, *J* = 1.2 Hz, 1H), 7.35 (d, *J* = 8.0 Hz, 1H), 7.10 (s, 1H), 7.01 – 6.91 (m, 1H), 5.26 (s, 2H), 4.75 (s, 2H), 4.37 (t, *J* = 5.9 Hz, 2H), 4.10 (q, *J* = 7.3 Hz, 2H), 3.13 (t, *J* = 5.9 Hz, 2H), 1.46 (t, *J* = 7.3 Hz, 4H), 0.56 – 0.49 (m, 2H), 0.45 (td, *J* = 4.5, 2.5 Hz, 2H). ^13^C NMR (126 MHz, Chloroform-*d*) δ 148.50, 148.26, 148.06, 138.11, 135.36, 134.77, 132.90, 131.95 (q, *J* = 50.4 Hz), 132.06, 129.74, 128.56, 128.46, 128.25, 126.77, 126.70, 121.76 (q, *J* = 266.9 Hz), 119.50 (q, *J* = 4.0 Hz), 109.18, 107.99, 56.11, 47.11, 43.28, 42.24, 29.71, 28.96, 16.29, 12.12. Condition A: HPLC purity 97.37 %, t_R_ = 11.907 min. HRMS (ESI) m/z: calculated for C_28_H_27_F_3_N_7_O [M + H]^+^: 534.2224 found: 534.2226.

*4-(6-(1-ethyl-4-(trifluoromethyl)-1H-imidazol-2-yl)-3,4-dihydroisoquinolin-2(1H)-yl)-2-phenylfuro[3,2-d]pyrimidine (****14m****).* Compound **14m** was prepared in a manner similar to that described for compound **14a**: yield 61%; ^1^H NMR (600 MHz, Chloroform-*d*) δ 8.44 – 8.32 (m, 2H), 7.73 (d, *J* = 2.2 Hz, 1H), 7.50 – 7.33 (m, 5H), 7.32 – 7.26 (m, 2H), 6.90 (s, 1H), 5.23 (d, *J* = 2.1 Hz, 2H), 4.32 (t, *J* = 5.9 Hz, 2H), 4.01 (q, *J* = 7.3 Hz, 2H), 3.05 (t, *J* = 5.9 Hz, 2H), 1.37 (t, *J* = 7.3 Hz, 3H). ^13^C NMR (126 MHz, Chloroform-*d*) δ 150.22, 147.32, 147.11, 134.43, 133.96, 132.50, 130.84 (q, *J* = 38.9 Hz), 129.19, 129.07, 128.69, 127.37, 127.27, 125.76, 125.70, 120.74 (q, *J* = 267.0 Hz), 118.42 (q, *J* = 4.0 Hz), 106.85, 46.00, 42.05, 41.20, 28.67, 27.94, 23.83, 15.25. Condition A: HPLC purity 99.20 %, t_R_ = 12.685 min. HRMS (ESI) m/z: calculated for C_27_H_23_F_3_N_5_O [M + H]^+^: 490.1849 found: 490.1847.

*4-(6-(1-ethyl-4-(trifluoromethyl)-1H-imidazol-2-yl)-3,4-dihydroisoquinolin-2(1H)-yl)-2-(2-methoxyphenyl)furo[3,2-d]pyrimidine (****14n****).* Compound **14n** was prepared in a manner similar to that described for compound **14a**: yield 60%; ^1^H NMR (600 MHz, Chloroform-*d*) δ 7.79 (d, *J* = 2.1 Hz, 1H), 7.71 (dd, *J* = 7.5, 1.8 Hz, 1H), 7.49 – 7.44 (m, 1H), 7.39 (ddd, *J* = 8.1, 7.3, 1.8 Hz, 2H), 7.36 (t, *J* = 1.3 Hz, 1H), 7.31 (d, *J* = 7.9 Hz, 1H), 7.08 – 7.01 (m, 2H), 7.00 – 6.96 (m, 1H), 5.24 (s, 2H), 4.35 (t, *J* = 5.9 Hz, 2H), 4.07 (q, *J* = 7.3 Hz, 2H), 3.88 (s, 3H), 3.09 (t, *J* = 5.9 Hz, 2H), 1.43 (t, *J* = 7.3 Hz, 3H). ^13^C NMR (126 MHz, Chloroform-*d*) δ 156.54, 149.99, 147.35, 147.14, 147.03, 134.43, 134.11, 132.16, 131.08 (q, *J* = 9.9 Hz), 130.95, 130.49, 129.39, 128.67, 127.54, 127.44, 126.98, 125.70, 125.61, 120.74 (q, *J* = 267.0 Hz), 119.45, 118.40 (q, *J* = 3.9 Hz), 110.89, 107.06, 55.02, 45.95, 42.05, 41.19, 28.68, 27.95, 15.25. Condition B: HPLC purity 99.48 %, t_R_ = 15.631 min. HRMS (ESI) m/z: calculated for C_28_H_24_F_3_N_5_O_2_ [M + H]^+^: 520.1995 found: 520.1995.

*2-(3-(tert-butyl)phenyl)-4-(6-(1-ethyl-4-(trifluoromethyl)-1H-imidazol-2-yl)-3,4-dihydroisoquinolin-2(1H)-yl)furo[3,2-d]pyrimidine (****14o****).* Compound **14o** was prepared in a manner similar to that described for compound **14a**: yield 68%; ^1^H NMR (600 MHz, Chloroform-*d*) δ 8.51 (t, *J* = 1.9 Hz, 1H), 8.25 (dt, *J* = 7.6, 1.4 Hz, 1H), 7.79 (d, *J* = 2.2 Hz, 1H), 7.52 – 7.46 (m, 2H), 7.44 – 7.39 (m, 2H), 7.38 – 7.32 (m, 2H), 6.97 (s, 1H), 5.30 (s, 2H), 4.39 (t, *J* = 5.9 Hz, 2H), 4.08 (q, *J* = 7.3 Hz, 2H), 3.12 (t, *J* = 5.9 Hz, 2H), 1.43 (d, *J* = 8.4 Hz, 12H). ^13^C NMR (151 MHz, Chloroform-*d*) δ 162.13, 150.74, 147.99, 147.65, 135.10, 134.79, 133.15, 131.43 (q, *J* = 38.7 Hz), 129.32, 127.66, 127.61, 126.63, 126.35, 126.26, 125.14, 124.71, 121.36 (q, *J* = 267.2 Hz), 119.02 (q, *J* = 4.0 Hz), 107.67, 46.56, 42.59, 41.80, 34.47, 31.04, 29.30, 28.51, 15.88. Condition A: HPLC purity 96.81 %, t_R_ = 6.123 min. HRMS (ESI) m/z: calculated for C_31_H_31_F_3_N_5_O [M + H]^+^: 546.2475 found: 546.2476.

*4-(6-(1-ethyl-4-(trifluoromethyl)-1H-imidazol-2-yl)-3,4-dihydroisoquinolin-2(1H)-yl)-2-(3-propylphenyl)furo[3,2-d]pyrimidine (****14p****).* Compound **14p** was prepared in a manner similar to that described for compound **14a**: yield 69%; ^1^H NMR (600 MHz, Chloroform-*d*) δ 8.22 – 8.13 (m, 2H), 7.68 (d, *J* = 2.2 Hz, 1H), 7.36 (d, *J* = 1.8 Hz, 1H), 7.33 – 7.24 (m, 4H), 7.19 – 7.15 (m, 1H), 6.84 (d, *J* = 2.2 Hz, 1H), 5.18 (s, 2H), 4.27 (t, *J* = 5.9 Hz, 2H), 3.97 (q, *J* = 7.3 Hz, 2H), 3.01 (t, *J* = 5.9 Hz, 2H), 2.61 (dd, *J* = 8.6, 6.7 Hz, 2H), 1.72 – 1.58 (m, 2H), 1.32 (t, *J* = 7.3 Hz, 3H), 0.89 (t, *J* = 7.4 Hz, 3H). ^13^C NMR (151 MHz, CDCl_3_) δ 159.19, 151.74, 148.00, 147.66, 142.33, 138.03, 135.13, 134.81, 133.19, 131.34 (q, *J* = 38.7 Hz), 129.56, 129.25, 127.78, 127.72, 127.55, 126.37, 126.22, 125.32, 121.40 (q, *J* = 266.9 Hz), 119.06 (q, *J* = 3.8 Hz), 107.67, 46.53, 42.48, 41.81, 37.74, 29.31, 28.55, 24.25, 15.84, 13.53. Condition B: HPLC purity 100.00 %, t_R_ = 13.054 min. HRMS (ESI) m/z: calculated for C_30_H_29_F_3_N_5_O [M + H]^+^: 532.2319 found: 532.2318.

*2-(2,6-dimethylphenyl)-4-(6-(1-ethyl-4-(trifluoromethyl)-1H-imidazol-2-yl)-3,4-dihydroisoquinolin-2(1H)-yl)furo[3,2-d]pyrimidine (****14q****).* Compound **14q** was prepared in a manner similar to that described for compound **14a**: yield 68%; ^1^H NMR (600 MHz, Chloroform-*d*) δ 7.82 (d, *J* = 2.2 Hz, 1H), 7.48 (d, *J* = 1.8 Hz, 1H), 7.40 – 7.34 (m, 2H), 7.30 (d, *J* = 8.0 Hz, 1H), 7.19 (t, *J* = 7.6 Hz, 1H), 7.10 (d, *J* = 7.6 Hz, 2H), 6.92 (s, 1H), 5.20 (s, 2H), 4.34 (t, *J* = 5.8 Hz, 2H), 4.08 (q, *J* = 7.3 Hz, 2H), 3.08 (t, *J* = 5.9 Hz, 2H), 2.16 (s, 6H), 1.44 (t, *J* = 7.3 Hz, 3H). ^13^C NMR (126 MHz, Chloroform-*d*) δ 150.70, 147.77, 135.23, 134.89, 132.39, 131.39 (q, *J* = 38.6 Hz), 129.20, 128.17, 127.18, 127.02, 126.23, 126.17, 121.26 (q, *J* = 265.9 Hz), 119.47, 118.95 (q, *J* = 4.0 Hz), 107.38, 107.29, 46.54, 42.68, 41.26, 29.21, 28.49, 24.36, 19.64, 19.52, 14.99. Condition A: HPLC purity 95.55 %, t_R_ = 4.115 min. HRMS (ESI) m/z: calculated for C_29_H_27_F_3_N_5_O [M + H]^+^: 518.2162 found: 518.2162.

*2-(2,6-dimethoxyphenyl)-4-(6-(1-ethyl-4-(trifluoromethyl)-1H-imidazol-2-yl)-3,4-dihydroisoquinolin-2(1H)-yl)furo[3,2-d]pyrimidine (****14r****).* Compound **14r** was prepared in a manner similar to that described for compound **14a**: yield 62%; ^1^H NMR (600 MHz, Chloroform-*d*) δ 7.68 (d, *J* = 2.2 Hz, 1H), 7.36 (d, *J* = 1.8 Hz, 1H), 7.31 – 7.25 (m, 2H), 7.24 – 7.16 (m, 2H), 6.80 (d, *J* = 2.2 Hz, 1H), 6.57 (d, *J* = 8.4 Hz, 2H), 5.09 (s, 2H), 4.21 (t, *J* = 5.9 Hz, 2H), 3.98 (q, *J* = 7.3 Hz, 2H), 3.65 (s, 6H), 2.97 (t, *J* = 5.9 Hz, 2H), 1.33 (t, *J* = 7.3 Hz, 3H). ^13^C NMR (126 MHz, CDCl_3_) δ 157.14, 156.91, 147.44, 147.41, 146.49, 134.59, 134.42, 132.39, 130.71 (q, *J* = 38.7 Hz), 128.56, 128.33, 127.57, 126.78, 125.68, 125.46, 120.78 (q, *J* = 267.0 Hz), 119.72, 119.20, 119.17, 118.61, 118.41 (q, *J* = 4.0 Hz), 107.17, 103.34, 55.09, 45.88, 41.96, 41.17, 28.66, 27.99, 15.21. Condition A: HPLC purity 96.55 %, t_R_ = 4.425 min. HRMS (ESI) m/z: calculated for C_29_H_27_F_3_N_5_O_3_ [M + H]^+^: 550.2061 found: 550.2062.

*2-(2,6-diethyl-4-methylphenyl)-4-(6-(1-ethyl-4-(trifluoromethyl)-1H-imidazol-2-yl)-3,4-dihydroisoquinolin-2(1H)-yl)furo[3,2-d]pyrimidine (****14s****).* Compound **14s** was prepared in a manner similar to that described for compound **14a**: yield 63%; ^1^H NMR (600 MHz, Chloroform-*d*) δ 7.74 (d, *J* = 2.1 Hz, 1H), 7.40 (d, *J* = 1.8 Hz, 1H), 7.33 – 7.28 (m, 2H), 7.21 (d, *J* = 8.0 Hz, 1H), 6.89 (s, 2H), 6.84 (s, 1H), 5.12 (s, 2H), 4.26 (t, *J* = 5.9 Hz, 2H), 4.01 (q, *J* = 7.3 Hz, 2H), 2.99 (t, *J* = 5.8 Hz, 2H), 2.34 (q, *J* = 7.6 Hz, 4H), 2.29 (s, 3H), 1.37 (t, *J* = 7.4 Hz, 3H), 1.02 (t, *J* = 7.6 Hz, 6H). ^13^C NMR (126 MHz, Chloroform-*d*) δ 147.89, 147.48, 141.03, 137.36, 134.99, 134.61, 132.45, 131.42 (q, *J* = 38.7 Hz), 129.23, 127.57, 126.26, 126.16, 126.07, 121.31 (q, *J* = 267.0 Hz), 118.97 (q, *J* = 4.1 Hz), 107.44, 46.56, 42.61, 41.75, 31.48, 30.99, 29.74, 29.25, 28.53, 26.04, 20.97, 15.82, 15.03. Condition A: HPLC purity 95.09 %, t_R_ = 15.345 min. HRMS (ESI) m/z: calculated for C_32_H_33_F_3_N_5_O [M + H]^+^: 560.2559 found: 560.2629.

*2-(3,5-diisopropylphenyl)-4-(6-(1-ethyl-4-(trifluoromethyl)-1H-imidazol-2-yl)-3,4-dihydroisoquinolin-2(1H)-yl)furo[3,2-d]pyrimidine (****14t****).* Compound **14t** was prepared in a manner similar to that described for compound **14a**: yield 64%; ^1^H NMR (600 MHz, Chloroform-*d*) δ 8.12 (d, *J* = 1.8 Hz, 2H), 7.78 (d, *J* = 2.1 Hz, 1H), 7.47 (d, *J* = 1.8 Hz, 1H), 7.41 (dd, *J* = 7.9, 1.8 Hz, 1H), 7.37 – 7.34 (m, 2H), 7.18 (t, *J* = 1.8 Hz, 1H), 6.96 (d, *J* = 2.2 Hz, 1H), 5.30 (s, 2H), 4.39 (t, *J* = 5.9 Hz, 2H), 4.07 (q, *J* = 7.3 Hz, 2H), 3.12 (t, *J* = 5.9 Hz, 2H), 3.02 (p, *J* = 6.9 Hz, 2H), 1.43 (t, *J* = 7.3 Hz, 3H), 1.34 (d, *J* = 6.9 Hz, 12H). ^13^C NMR (151 MHz, Chloroform-*d*) δ 160.60, 148.49, 148.01, 147.64, 147.60, 135.14, 134.87, 133.15, 131.41 (q, *J* = 38.7 Hz), 129.31, 127.58, 126.35, 126.24, 125.79, 121.37 (q, *J* = 267.0 Hz), 123.59, 119.02 (q, *J* = 4.0 Hz), 107.72, 46.59, 42.53, 41.80, 33.93, 29.30, 28.51, 23.75, 22.48. Condition A: HPLC purity 99.72 %, t_R_ = 15.663 min. HRMS (ESI) m/z: calculated for C_33_H_35_F_3_N_5_O [M + H]^+^: 574.2788 found: 574.2787.

*4-(6-(1-ethyl-4-(trifluoromethyl)-1H-imidazol-2-yl)-3,4-dihydroisoquinolin-2(1H)-yl)-2-(3,4,5-trifluorophenyl)furo[3,2-d]pyrimidine (****14u****).* Compound **14u** was prepared in a manner similar to that described for compound **14a**: yield 66 %; ^1^H NMR (600 MHz, Chloroform-*d*) δ 8.10 (dd, *J* = 9.1, 6.8 Hz, 2H), 7.82 (d, *J* = 2.1 Hz, 1H), 7.50 (d, *J* = 1.8 Hz, 1H), 7.43 (dd, *J* = 7.8, 1.8 Hz, 1H), 7.41 – 7.36 (m, 2H), 6.92 (d, *J* = 2.2 Hz, 1H), 5.26 (s, 2H), 4.37 (t, *J* = 5.9 Hz, 2H), 4.09 (q, *J* = 7.3 Hz, 2H), 3.12 (t, *J* = 5.9 Hz, 2H), 1.45 (t, *J* = 7.3 Hz, 3H). ^13^C NMR (126 MHz, CDCl_3_) δ 155.14, 151.08 (dd, *J* = 10.0, 3.8 Hz), 149.10 (dd, *J* = 10.0, 3.8 Hz), 147.58, 147.27, 147.03, 140.94 (t, *J* = 15.6 Hz), 138.92 (t, *J* = 15.6 Hz), 134.36, 133.73, 132.66, 130.86 (q, *J* = 38.7 Hz), 128.69, 127.18, 125.75, 125.73, 120.74 (q, *J* = 267.0 Hz), 118.45 (q, *J* = 4.0 Hz), 111.24 (q, *J* = 5.3 Hz), 111.10 (q, *J* = 5.3 Hz), 106.92, 45.95, 42.06, 41.21, 28.68, 27.92, 15.24. Condition A: HPLC purity 99.36 %, t_R_ = 7.891 min. HRMS (ESI) m/z: calculated for C_27_H_20_F_6_N_5_O [M + H]^+^: 544.1567 found: 544.1568.

*4-(6-(1-ethyl-4-(trifluoromethyl)-1H-imidazol-2-yl)-3,4-dihydroisoquinolin-2(1H)-yl)-2-(3,4,5-trimethoxyphenyl)furo[3,2-d]pyrimidine (****14v****).* Compound **14v** was prepared in a manner similar to that described for compound **14a**: yield 69%; ^1^H NMR (600 MHz, Chloroform-*d*) δ 7.78 (d, *J* = 2.2 Hz, 1H), 7.74 (s, 2H), 7.45 (d, *J* = 1.8 Hz, 1H), 7.39 (dd, *J* = 7.9, 1.8 Hz, 1H), 7.35 (d, *J* = 1.2 Hz, 1H), 7.32 (d, *J* = 7.9 Hz, 1H), 6.91 (d, *J* = 2.2 Hz, 1H), 5.26 (s, 2H), 4.35 (t, *J* = 5.9 Hz, 2H), 4.06 (q, *J* = 7.3 Hz, 2H), 3.99 (s, 6H), 3.90 (s, 3H), 3.09 (t, *J* = 5.9 Hz, 2H), 1.41 (t, *J* = 7.3 Hz, 3H). ^13^C NMR (126 MHz, Chloroform-*d*) δ 158.87, 153.08, 152.05, 148.35, 148.23, 148.01, 139.80, 135.50, 135.12, 134.01, 133.53, 132.14, 132.06, 131.77 (q, *J* = 38.8 Hz), 129.71, 128.59, 128.49, 128.06, 126.69, 121.78 (q, *J* = 267.1 Hz), 119.50 (q, *J* = 4.0 Hz), 107.98, 105.52, 60.94, 56.26, 47.00, 42.94, 42.22, 28.89, 16.25. Condition A: HPLC purity 100.00 %, t_R_ = 4.297 min. HRMS (ESI) m/z: calculated for C_30_H_29_F_3_N_5_O_4_ [M + H]^+^: 580.2166 found: 580.2166.

*4-(6-(1-ethyl-4-(trifluoromethyl)-1H-imidazol-2-yl)-3,4-dihydroisoquinolin-2(1H)-yl)-2-(naphthalen-2-yl)furo[3,2-d]pyrimidine (****14w****).* Compound **14w** was prepared in a manner similar to that described for compound **14a**: yield 67%; ^1^H NMR (600 MHz, Chloroform-*d*) δ 8.96 – 8.93 (m, 1H), 8.57 (dd, *J* = 8.6, 1.7 Hz, 1H), 8.03 – 7.98 (m, 1H), 7.92 (d, *J* = 8.6 Hz, 1H), 7.89 – 7.84 (m, 1H), 7.79 (d, *J* = 2.1 Hz, 1H), 7.57 – 7.45 (m, 3H), 7.44 – 7.33 (m, 3H), 6.97 (d, *J* = 2.1 Hz, 1H), 5.30 (s, 2H), 4.39 (t, *J* = 5.9 Hz, 2H), 4.06 (q, *J* = 7.3 Hz, 2H), 3.12 (t, *J* = 5.9 Hz, 2H), 1.41 (t, *J* = 7.3 Hz, 3H). ^13^C NMR (126 MHz, Chloroform-*d*) δ 158.89, 151.88, 147.92, 147.64, 135.53, 135.07, 134.73, 133.74, 133.20, 132.86, 131.30 (q, *J* = 38.8 Hz), 129.19, 128.57, 127.50, 127.37, 127.31, 127.15, 126.31, 126.16, 126.08, 125.50, 125.18, 121.32 (q, *J* = 267.0 Hz), 118.96 (q, *J* = 4.0 Hz), 107.64, 46.48, 42.43, 41.72, 29.21, 29.07, 28.52, 27.55, 15.75. Condition A: HPLC purity 96.44 %, t_R_ = 5.322 min. HRMS (ESI) m/z: calculated for C_31_H_25_F_3_N_5_O [M + H]^+^: 540.2006 found: 540.2008.

*2-(2,3-dihydrobenzo[b][1,4]dioxin-6-yl)-4-(6-(1-ethyl-4-(trifluoromethyl)-1H-imidazol-2-yl)-3,4-dihydroisoquinolin-2(1H)-yl)furo[3,2-d]pyrimidine (****14y****)*. Compound **14y** was prepared in a manner similar to that described for compound **14a**: yield 62%; ^1^H NMR (600 MHz, Chloroform-*d*) δ 8.02 – 7.95 (m, 2H), 7.77 (d, *J* = 2.2 Hz, 1H), 7.47 (d, *J* = 1.7 Hz, 1H), 7.41 (dd, *J* = 7.9, 1.8 Hz, 1H), 7.39 – 7.35 (m, 2H), 6.95 (d, *J* = 8.4 Hz, 1H), 6.93 – 6.91 (m, 1H), 5.26 (s, 2H), 4.36 (t, *J* = 5.9 Hz, 2H), 4.32 (s, 4H), 4.08 (q, *J* = 7.3 Hz, 2H), 3.10 (t, *J* = 5.9 Hz, 2H), 1.44 (t, *J* = 7.3 Hz, 3H). ^13^C NMR (126 MHz, CDCl_3_) δ 158.26, 147.95, 147.57, 144.89, 142.84, 135.07, 134.70, 132.97, 131.39 (q, *J* = 38.9 Hz), 129.22, 127.54, 126.34, 126.21, 121.32 (q, *J* = 267.1 Hz), 120.26, 118.96 (q, *J* = 4.0 Hz), 116.92, 116.63, 107.50, 64.16, 63.89, 46.50, 42.50, 41.77, 29.26, 28.54, 15.82. Condition A: HPLC purity 95.46 %, t_R_ = 9.376 min. HRMS (ESI) m/z: calculated for C_29_H_25_F_3_N_5_O_3_ [M + H]^+^: 548.1904 found: 548.1906.

*tert-butyl2-(4-cyclopropyl-6-methoxypyrimidin-5-yl)-4-(6-(1-ethyl-4-(trifluoromethyl)-1H-imidazol-2-yl)-3,4-dihydroisoquinolin-2(1H)-yl)-7,8-dihydropyrido[4,3-d]pyrimidine-6(5H)-carboxylate* *(****15****).* Under argon, compound **12s** (0.2 g, 0.295 mmol), (4-cyclopropyl-6-methoxypyrimidin-5-yl) boronic acid (0.074 g, 0.380 mmol), were dissolved in 1.4-dioxane/H_2_O (20.0 mL), followed by the addition of Pd(PPh_3_)_4_ (0.030 g, 0.026 mmol) and potassium carbonate (0.163 g, 1.183 mmol), stirred at 100°C for 12 h. Upon completion, the mixture was filtered and the filter cake was washed with ethyl acetate (3 × 10 mL), and then extracted with ethyl acetate (3 × 20 mL) and water (H_2_O). The combined organic phase was washed with brined (20 mL) and dried with anhydrous sodium sulfate. The solution was concentrated to give a white solid. The solid was separated by column chromatography with petroleum ether/ethyl acetate (10/10 v/v) as an eluent to obtain a white solid as target compound **15** (0.15 g, yield 60%). ^1^H NMR (600 MHz, Chloroform-*d*) δ 8.61 (s, 1H), 7.45 – 7.42 (m, 1H), 7.37 – 7.32 (m, 2H), 7.21 (d, *J* = 7.9 Hz, 1H), 4.71 (s, 2H), 4.63 (s, 2H), 4.06 (q, *J* = 7.3 Hz, 2H), 3.91 (s, 3H), 3.78 (t, *J* = 6.3 Hz, 2H), 3.72 (t, *J* = 5.7 Hz, 2H), 3.10 (t, *J* = 5.8 Hz, 2H), 2.99 (t, *J* = 6.2 Hz, 2H), 1.77 (tt, *J* = 8.3, 4.6 Hz, 1H), 1.48 (s, 9H), 1.43 (t, *J* = 7.3 Hz, 3H), 1.19 (dt, *J* = 6.6, 3.3 Hz, 2H),0.97 – 0.86 (m, 2H). HRMS (ESI) m/z: calculated for C_35_H_39_F_3_N_8_O_3_ [M + H]^+^: 677.3170 found: 677.3167.

*2-(4-cyclopropyl-6-methoxypyrimidin-5-yl)-N-(3',6'-dihydroxy-3-oxo-3H-spiro[isobenzofuran-1,9'-xanthen]-6-yl)-4-(6-(1-ethyl-4-(trifluoromethyl)-1H-imidazol-2-yl)-3,4-dihydroisoquinolin-2(1H)-yl)-7,8-dihydropyrido[4,3-d]pyrimidine-6(5H)-carbothioamide (****6-1****).* To a solution of **15** (0.2 g, 0.296 mmol) in 30 mL of dichloromethane was added TFA (0.33 g, 2.96 mmol). The resulting mixture was stirred at 40 °C for 12 h. Upon completion, the reaction mixture was concentrated and re-dissolved in dichloromethane (20 mL), followed by washing with 1.5 M NaOH (20 mL). The water phase was extracted three times with dichloromethane (3 × 50 mL). The combined organic phase was washed with brine (100 mL) and dried with anhydrous sodium sulfate. The solution was concentrated to give a yellow oil as compound **16**, which was used without purification in the next reaction. This amine (100 mg, 0.17 mmol) was added to fluorescein 6-isothiocyanate (88 mg, 0.225 mmol) in EtOH/THF(3:2, 5 mL), cooled to 0°C, and triethylamine (10 uL, 0.07 mmol) was added. The resulting orange reaction mixture was warmed to 20 °C and stirred in the dark for 12 h. The reaction was concentrated in vacuo in the dark to give an orange solid that was purifed by column chromatography with dichloromethane/ methanol (10/1 v/v) as an eluent to obtain an orange solid as target compound **6-1** (101 mg, yield 60%). ^1^H NMR (600 MHz, DMSO-*d_6_*) δ 10.17 (s, 2H), 9.80 (s, 1H), 9.30 (s, 2H), 8.63 (s, 1H), 8.01 (d, *J* = 1.4 Hz, 1H), 7.88 (d, *J* = 8.4 Hz, 1H), 7.82 (dd, *J* = 8.4, 1.8 Hz, 1H), 7.47 (d, *J* = 1.8 Hz, 1H), 7.43 (dd, *J* = 7.9, 1.8 Hz, 1H), 7.34 (d, *J* = 8.1 Hz, 1H), 7.13 (d, *J* = 1.8 Hz, 1H), 6.67 (d, *J* = 2.4 Hz, 2H), 6.63 (d, *J* = 8.6 Hz, 2H), 6.56 (dd, *J* = 8.7, 2.4 Hz, 2H), 5.24 (s, 2H), 4.69 (s, 2H), 4.17 (t, *J* = 6.3 Hz, 2H), 4.09 (m, 2H), 3.82 (s, 3H), 3.71 (t, *J* = 5.8 Hz, 2H), 2.99 (t, *J* = 6.3 Hz, 2H), 1.71 (tt, *J* = 8.1, 4.7 Hz, 1H), 1.31 (t, *J* = 7.3 Hz, 3H), 1.04 – 0.99 (m, 2H), 0.88 – 0.82 (m, 2H). ^13^C NMR (151 MHz, DMSO-*d_6_*) δ 180.11, 168.44, 168.06, 165.83, 162.73, 162.38, 159.43, 158.69, 158.05, 156.89, 153.61, 151.65, 147.66 (d, *J* = 9.3 Hz), 135.24 (d, *J* = 30.1 Hz), 129.11 (d, *J* = 27.2 Hz), 127.67, 126.97, 126.16, 125.82, 124.13, 123.11, 121.63, 121.34, 120.51, 118.75, 117.91 (d, *J* = 98.9 Hz), 116.25, 112.61, 112.39, 109.54, 102.17, 82.02, 53.80, 49.33, 47.61, 45.66, 44.95, 41.96, 31.20, 28.29, 15.76, 13.67, 10.49, 8.55. Condition A: HPLC purity 99.13 %, t_R_ = 7.316 min. HRMS (ESI) m/z: calculated for C_51_H_42_F_3_N_9_O_6_S [M + H]^+^: 966.3004 found: 966.3009.

*1-(4-(2-(4-cyclopropyl-6-methoxypyrimidin-5-yl)-4-(6-(1-ethyl-4-(trifluoromethyl)-1H-imidazol-2-yl)-3,4-dihydroisoquinolin-2(1H)-yl)-7,8-dihydropyrido[4,3-d]pyrimidin-6(5H)-yl)butyl)-3-(3',6'-dihydroxy-3-oxo-3H-spiro[isobenzofuran-1,9'-xanthen]-6-yl)thiourea (****6-2****).* To a solution of **16** (0.2 g, 0.347 mmol) in 8 mL of DMF was added *tert*-butyl (4-bromobutyl)carbamate (0.13 g, 0.52.8 mmol) and TEA (0.15 g, 1.38 mmol). The resulting mixture was stirred at 75°C for 12 h. Upon completion, it was extracted three times with ethyl acetate (3 × 50 mL). The combined organic phase was washed with and dried with anhydrous sodium sulfate. The solution was concentrated to give a yellow oil. The oil was separated by column chromatography with petroleum ether/ethyl acetate (10/20 v/v) as an eluent to obtain a yellow solid as compound **17**. To a solution of **17** in 10 mL of dichloromethane was added TFA. Upon completion, the reaction mixture was concentrated and re-dissolved in dichloromethane (20 mL), followed by washing with 1.5 M NaOH (20 mL). The water phase was extracted three times with dichloromethane (3 × 50 mL). The combined organic phase was washed with brine (100 mL) and dried with anhydrous sodium sulfate. The solution was concentrated to give a yellow oil, which was used without purification in the next reaction. This amine (100 mg, 0.154 mmol) was added to fluorescein 6-isothiocyanate (80 mg, 0.200 mmol) in EtOH/THF(3:2, 5 mL, cooled to 0°C, and triethylamine (10 uL, 0.07 mmol) was added. The resulting orange reaction mixture was warmed to 20°C and stirred in the dark for 12 h. The reaction was concentrated in vacuo in the dark to give an orange solid that was purified by column chromatography with dichloromethane/ methanol (10/1 v/v) as an eluent to obtain an orange solid as target compound **6-2** (120 mg, yield 67%). ^1^H NMR (500 MHz, DMSO-*d_6_*) δ 10.11 (s, 2H), 9.98 (s, 1H), 8.63 (s, 1H), 8.19 (s, 1H), 8.01 (s, 1H), 7.85 (d, *J* = 8.4 Hz, 1H), 7.76 (d, *J* = 8.6 Hz, 1H), 7.56 (s, 1H), 7.42 (d, *J* = 8.3 Hz, 2H), 7.35 (d, *J* = 7.9 Hz, 1H), 6.66 (d, *J* = 2.4 Hz, 2H), 6.61 (d, *J* = 8.7 Hz, 2H), 6.55 (dd, *J* = 8.7, 2.4 Hz, 2H), 4.65 (s, 2H), 4.09 (t, *J* = 7.3 Hz, 2H), 3.83 (s, 3H), 3.66 (t, *J* = 5.9 Hz, 2H), 3.55 (s, 2H), 3.46 (s, 2H), 3.09 (q, *J* = 7.3 Hz, 2H), 3.02 (t, *J* = 5.7 Hz, 2H), 2.82 (s, 2H), 1.99 (s, 2H), 1.72 (tt, *J* = 8.2, 4.8 Hz, 1H), 1.54 (s, 4H), 1.30 (t, *J* = 7.2 Hz, 3H)1.02 (p, *J* = 3.5 Hz, 2H), 0.87 (dq, *J* = 6.9, 3.3 Hz, 2H). ^13^C NMR (126 MHz, DMSO-*d_6_*) δ 179.88, 170.32, 168.23, 165.83, 162.66, 159.39, 158.20, 156.77, 153.64, 151.77, 147.71, 146.69, 135.47, 135.14, 128.99 (d, *J* = 8.3 Hz), 127.57, 126.93, 126.06, 125.04, 122.28, 121.60 (d, *J* = 4.0 Hz), 120.51 (d, *J* = 164.9 Hz), 118.99, 112.56, 109.77, 102.17, 82.15, 59.73, 56.76, 53.78, 52.71, 49.31, 45.67 (d, *J* = 12.5 Hz), 43.50, 41.94, 28.25, 26.04, 20.74, 15.76, 14.06, 13.66, 8.65. Condition E: HPLC purity 100 %, t_R_ = 19.909 min. HRMS (ESI) m/z: calculated for C_55_H_51_F_3_N_10_O_6_S [M + H]^+^: 1037.3739 found: 1037.3741.

*2-(3,6-bis(dimethylamino)xanthylium-9-yl)-4-(3-(6-(2-(4-cyclopropyl-6-methoxypyrimidin-5-yl)-4-(6-(1-ethyl-4-(trifluoromethyl)-1H-imidazol-2-yl)-3,4-dihydroisoquinolin-2(1H)-yl)-7,8-dihydropyrido[4,3-d]pyrimidin-6(5H)-yl)hexyl)-3-methylthioureido)benzoate (****6-3)***. To a solution of **16** (0.2 g, 0.347 mmol) in 8 mL of DMF was added *tert*-butyl(6-bromohexyl)(methyl)carbamate (0.15 g, 0.520 mmol) and TEA (0.15 g, 1.38 mmol). The resulting mixture was stirred at 75°C for 12 h. Upon completion, it was extracted three times with ethyl acetate (3 × 50 mL). The combined organic phase was washed with and dried with anhydrous sodium sulfate. The solution was concentrated to give a yellow oil. The oil was separated by column chromatography with petroleum ether/ethyl acetate (10/20 v/v) as an eluent to obtain a yellow solid as compound **19**. To a solution of **19** in 10 mL of dichloromethane was added TFA. Upon completion, the reaction mixture was concentrated and re-dissolved in dichloromethane (20 mL), followed by washing with 1.5 M NaOH (20 mL). The water phase was extracted three times with dichloromethane (3 × 50 mL). The combined organic phase was washed with brine (100 mL) and dried with anhydrous sodium sulfate. The solution was concentrated to give a yellow oil, which was used without purification in the next reaction. This amine (100 mg, 0.154 mmol) was added to tetramethylrhodamine-6-isothiocyanate (80 mg, 0.200 mmol) in EtOH/THF(3:2, 5 mL), cooled to 0°C, and triethylamine (10 uL, 0.07 mmol) was added. The resulting orange reaction mixture was warmed to 20°C and stirred in the dark for 12 h. The reaction was concentrated in vacuo in the dark to give an orange solid that was purified by column chromatography with dichloromethane/ methanol (10/1 v/v) as an eluent to obtain an orange solid as target compound **6-3** (107 mg, yield 62%).^1^H NMR (500 MHz, DMSO-*d*_6_) δ 9.22 (s, 1H), 8.64 (s, 1H), 8.01 (s, 1H), 7.87 – 7.73 (m, 2H), 7.47 – 7.39 (m, 2H), 7.35 (d, *J* = 8.0 Hz, 1H), 7.07 (s, 1H), 6.57 (d, *J* = 8.8 Hz, 2H), 6.53 – 6.43 (m, 4H), 4.65 (s, 2H), 4.08 (q, *J* = 7.3 Hz, 2H), 3.83 (s, 3H), 3.73 (t, *J* = 7.5 Hz, 2H), 3.66 (t, *J* = 5.8 Hz, 2H), 3.54 (s, 2H), 3.15 (s, 3H), 3.02 (t, *J* = 5.7 Hz, 2H), 2.91 (s, 13H), 2.81 (d, *J* = 5.9 Hz, 2H), 2.76 (d, *J* = 16.1 Hz, 2H), 1.72 (tt, *J* = 8.2, 4.8 Hz, 1H), 1.52 (dt, *J* = 28.5, 7.3 Hz, 4H), 1.27 (dt, *J* = 31.8, 6.1 Hz, 8H), 1.02 (p, *J* = 3.5 Hz, 2H), 0.86 (dq, *J* = 7.1, 3.4 Hz, 2H). ^13^C NMR (126 MHz, DMSO-*d*_6_) δ 180.58, 170.80, 169.14, 168.47, 166.32, 163.16, 158.71, 157.25, 152.48, 152.30, 148.17 (d, *J* = 5.8 Hz), 135.96, 135.60, 129.81 (q, *J* = 37.7 Hz), 129.51, 128.83, 128.06, 127.41, 126.54, 125.85, 124.28, 122.71 (q, *J* = 266.5 Hz), 122.08 (q, *J* = 4.4 Hz), 121.05, 119.49, 117.56, 109.46, 106.70, 98.40, 60.22, 57.64, 54.26, 49.80, 46.12, 42.42, 28.73, 27.02, 26.36, 21.22, 16.25, 14.55, 14.14, 10.90. Condition A: HPLC purity 99.72 %, t_R_ = 15.633 min. HRMS (ESI) m/z: calculated for C_62_H_67_F_3_N_12_O_4_S [M + H]^+^: 1133.5154 found: 1133.5147.


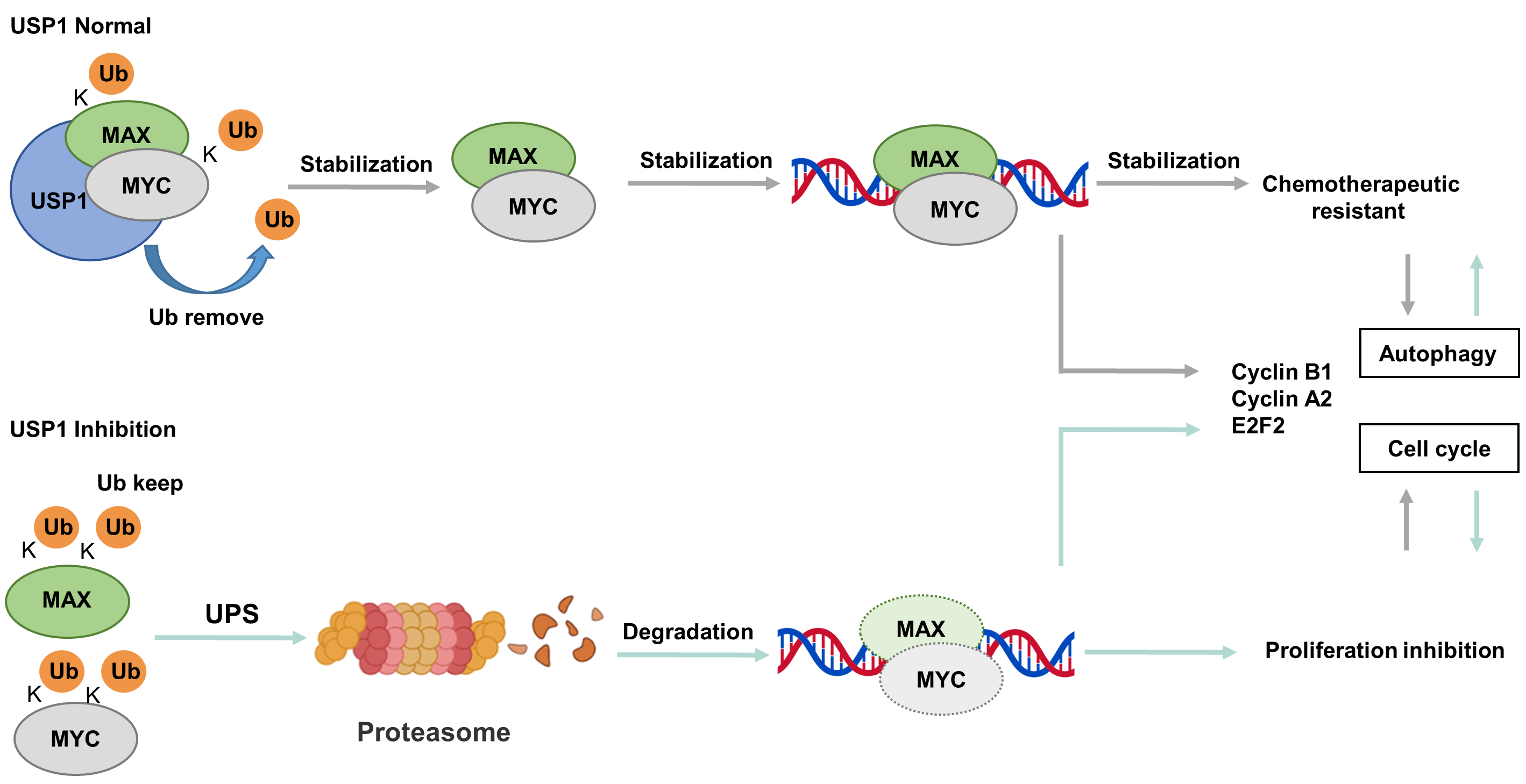


**Figure S1**. Schematic diagram of the functions and molecular mechanisms of USP1 in DLBCL.


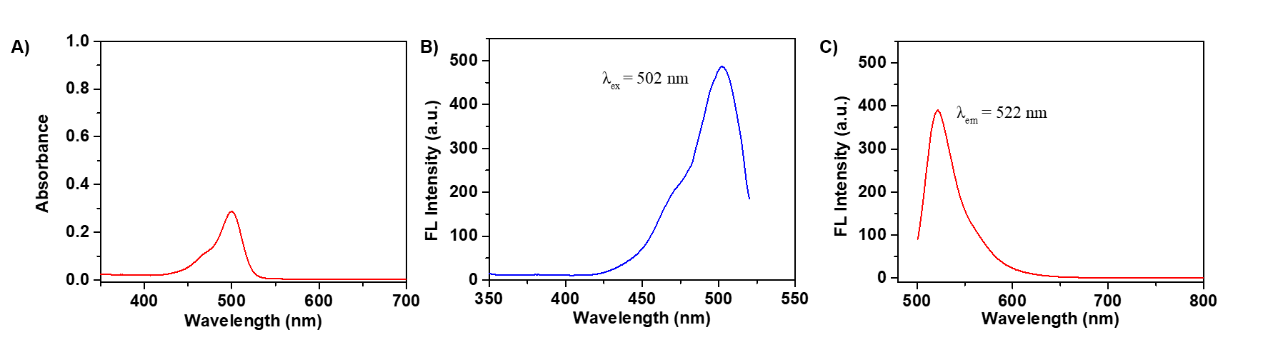


**Figure S2**. (A) Absorption spectrum of probe **6-2** in PBS; (B) Fluorescence excitation spectrum of probe **6-2** in PBS; (C) Fluorescence emission spectrum of probe **6-2** in PBS. The concentration of probe **6-2** was 5 μM in (A), (B), and (C).


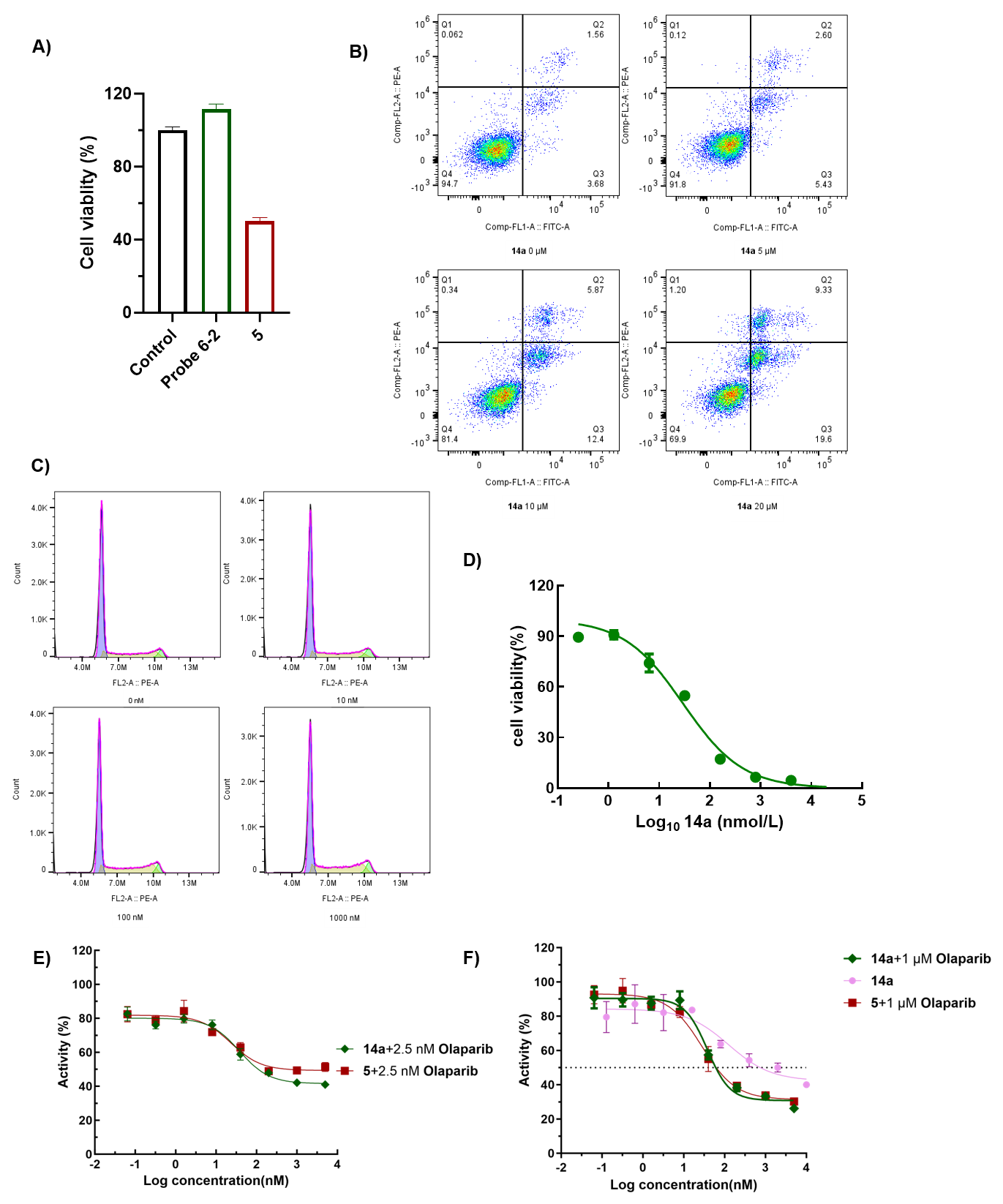


**Figure S3.** (A) CAOV-3 cell viability after treatment with probe **6-2** or **5** (up to 4 μM); (B) Apoptosis analysis of OCI-LY10 cells treated with compound **14a** for 72 h; (C) Quantification of cell cycle arrest in OCI-LY10 cells induced by compound **14a** after 72 h of treatment; (D) Effect of **14a** on the viability of TMD-8 cells; (E) The combined effects of **olaparib**/**14a** and **olaparib**/**5** on the viability of MDA-MB-436 cells; (F) Effect of **14a** and the combined effects of **olaparib**/**14a** and **olaparib**/**5** on the viability of CAOV-3 cells. Data are presented as mean ± SD.


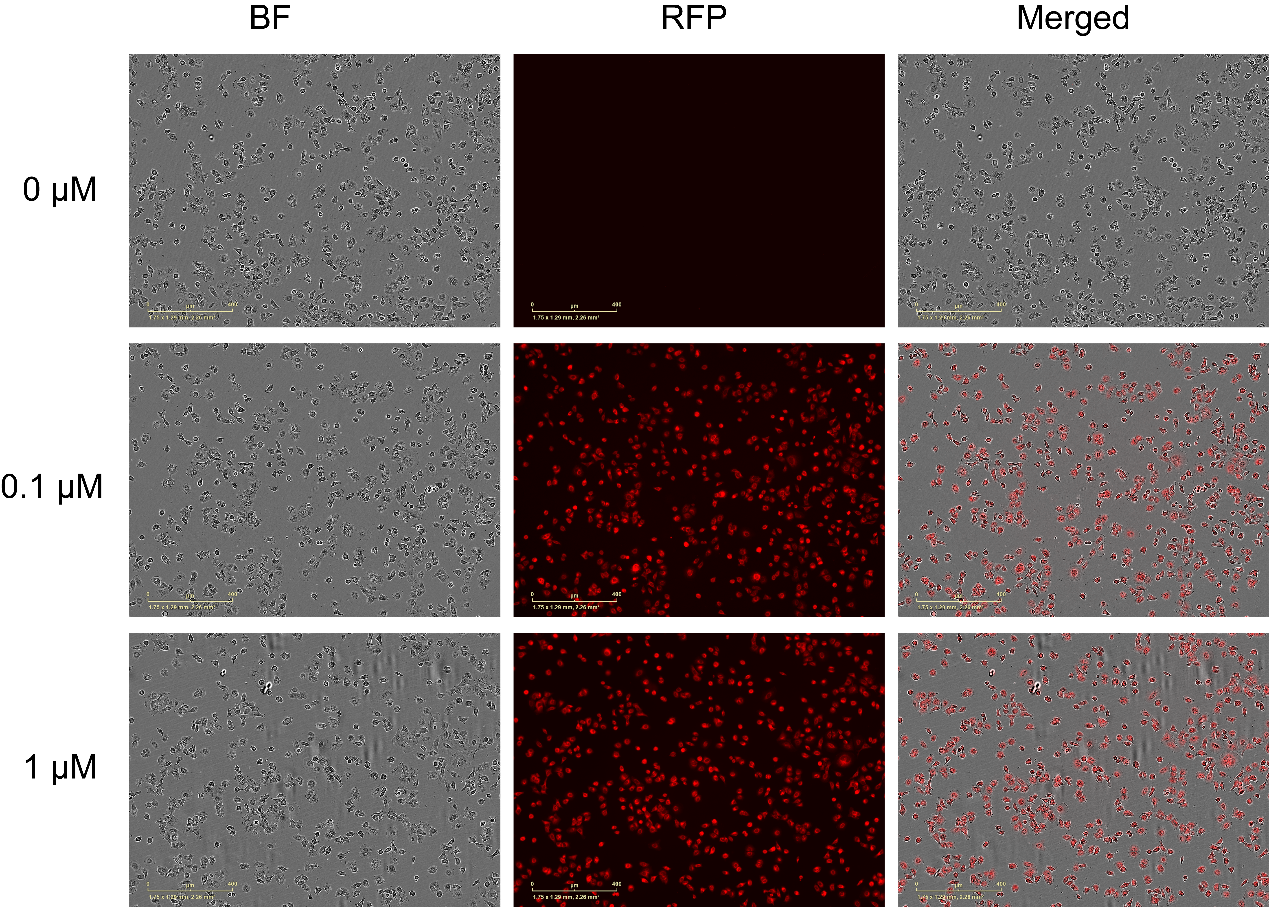


**Figure S4.** Probe **6-3** as a fluorescence tool to visualize target binding in living CAOV-3 cells.


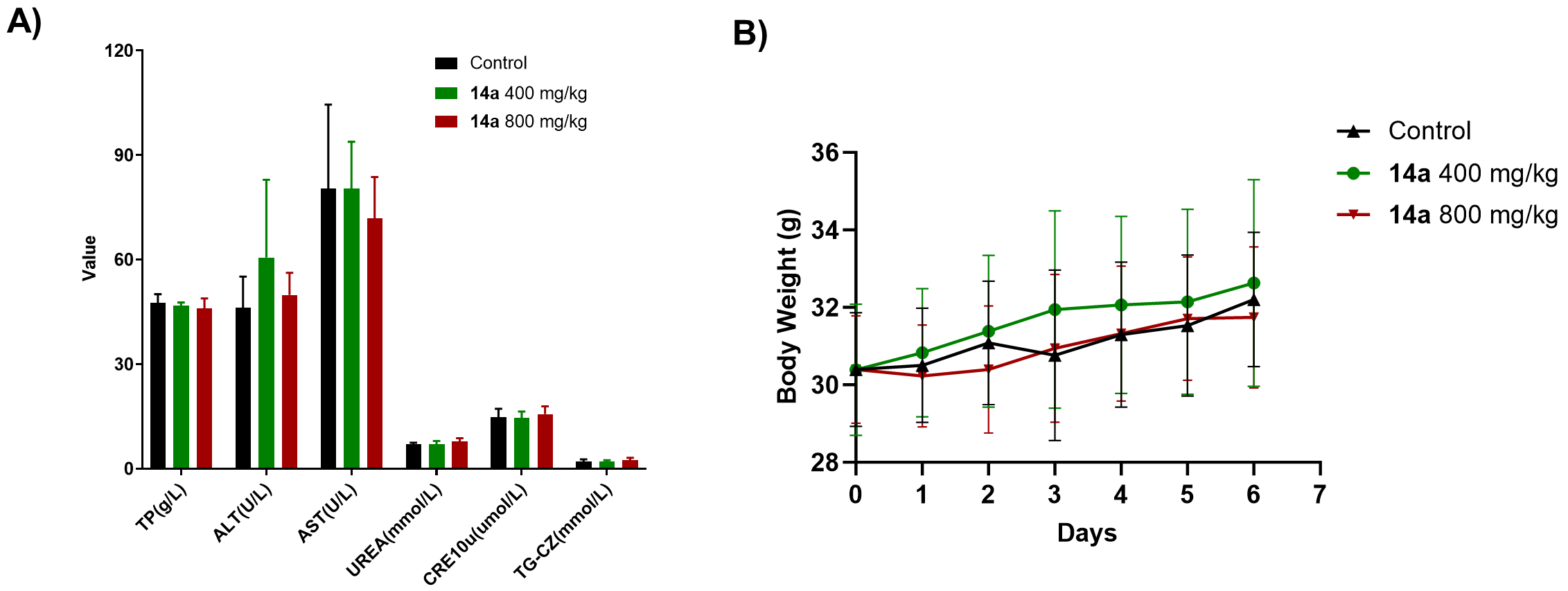


**Figure S5**. (A) Effects of compound **14a** at different doses (400 mg/kg and 800 mg/kg) on serum biochemical parameters after 24 h in mice; (B) Effects of compound **14a** with different doses (400 mg/kg and 800 mg/kg) on mice weight change for 6 days. Data are presented as mean ± SD.

**Table S3.** PBMC and HS-5 cells viability after treatment with **14a**.

| Compd. | PBMC IC_50_(μM) | HS-5 IC_50_(μM) |
| --- | --- | --- |
| **14a** | ＞10 | ＞10 |

**Table S4.** Metabolic stability in mouse liver microsomes.

| Compd. | Clint In Vitro (mL/min/gprot) | Clint In Vivo Extpl (mL/min) | Clint Hep In Vivo Extpl (mL/min) | MF % |
| --- | --- | --- | --- | --- |
| **14a** | 112 | 7.58 | 2.15 | 28.4 |

**Table S5.** Effect of **14a** on CYP450 enzymes activity.

| Compd. | CYP1A2 IC_50_(μM) | CYP2D6 IC_50_(μM) | CYP3A4 IC_50_(μM) |
| --- | --- | --- | --- |
| **14a** | ＞25 | ＞25 | ＞25 |

# 1H NMR spectra, 13C NMR spectra, LRMS, and HRMS of target compounds.


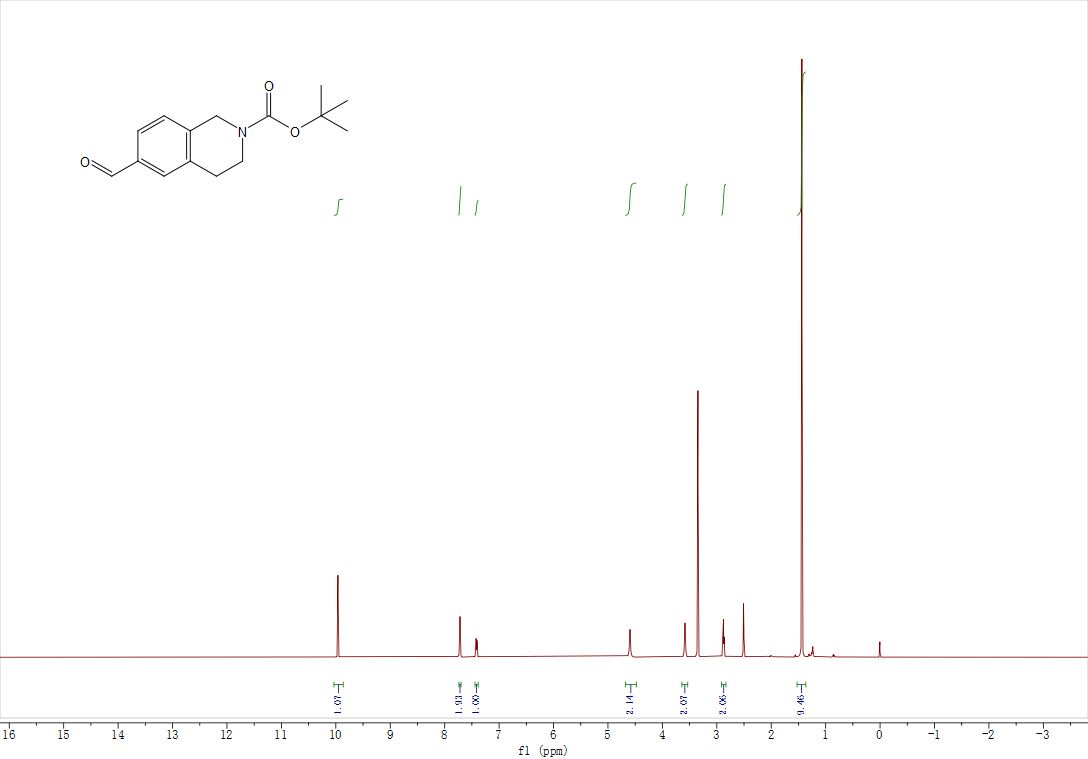


1H NMR of compound **8**

**
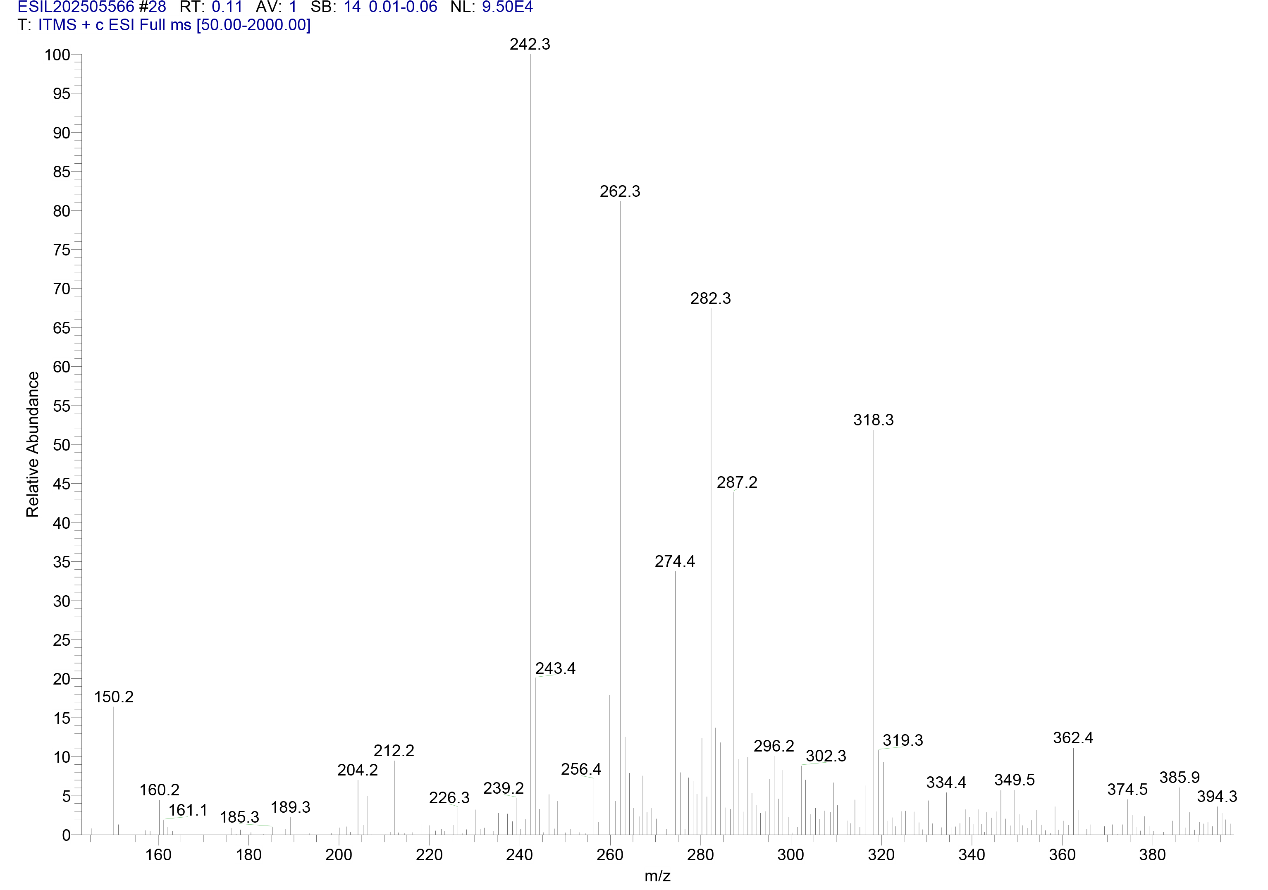
**LRMS (ESI) of compound **8**

**
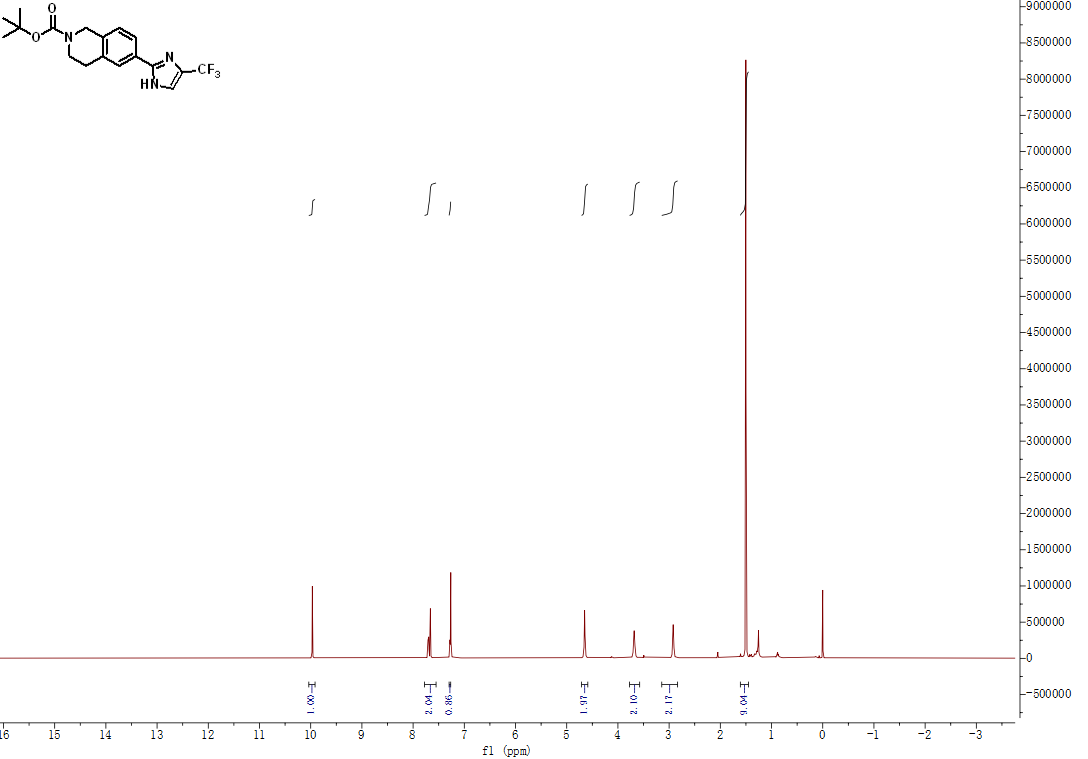
**

1H NMR of compound **9**

LRMS (ESI) of compound **9**


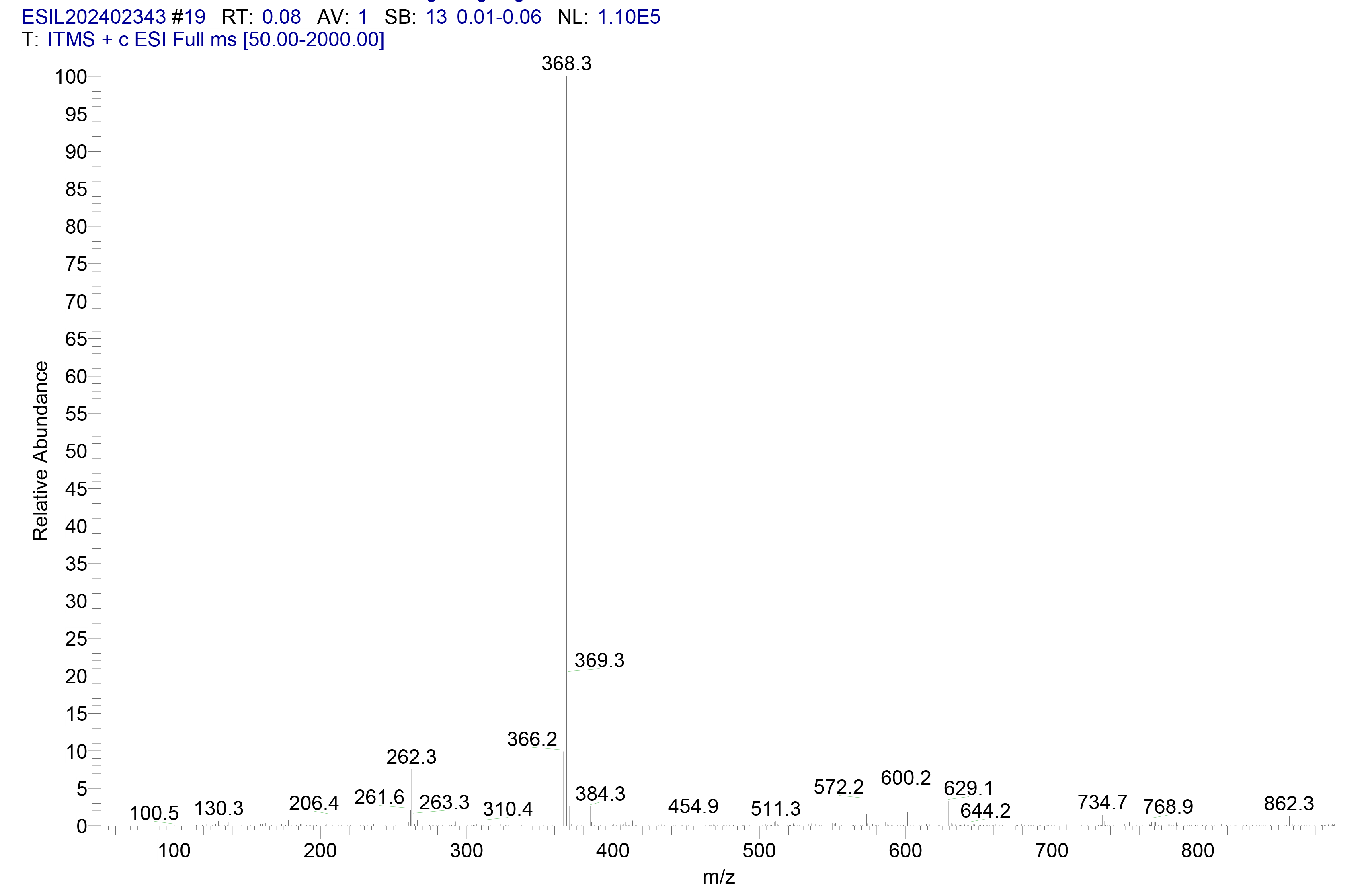


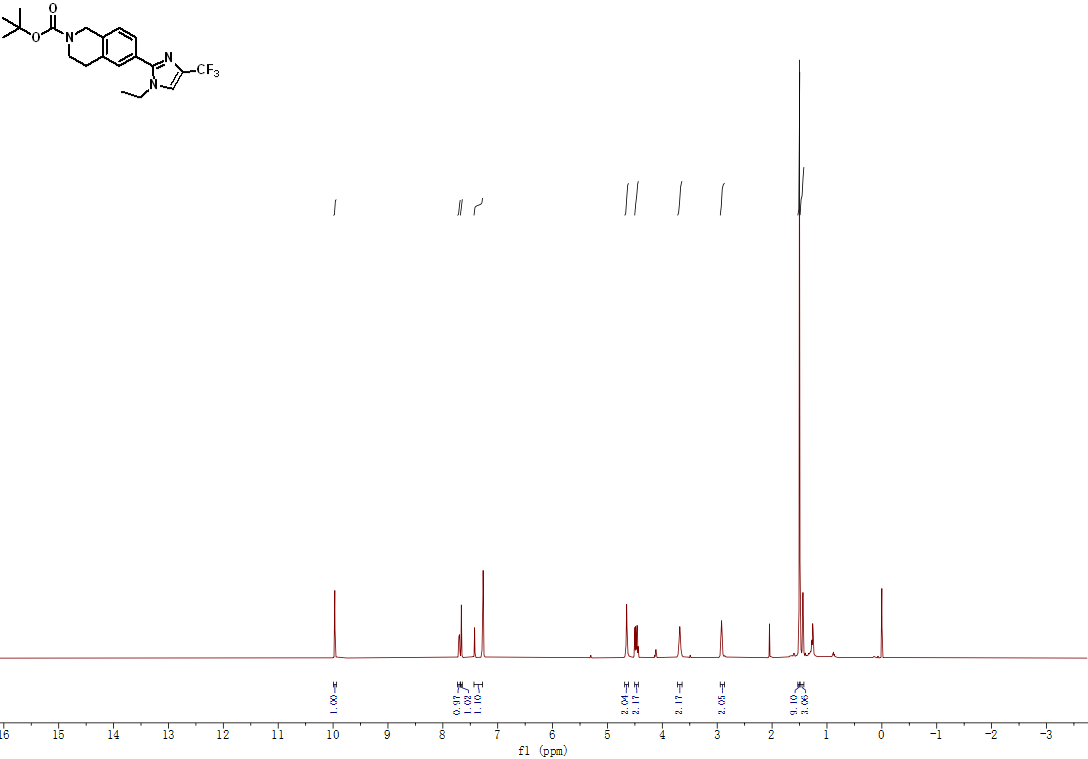
1H NMR of compound **10a**

LRMS (ESI) of compound **10a**


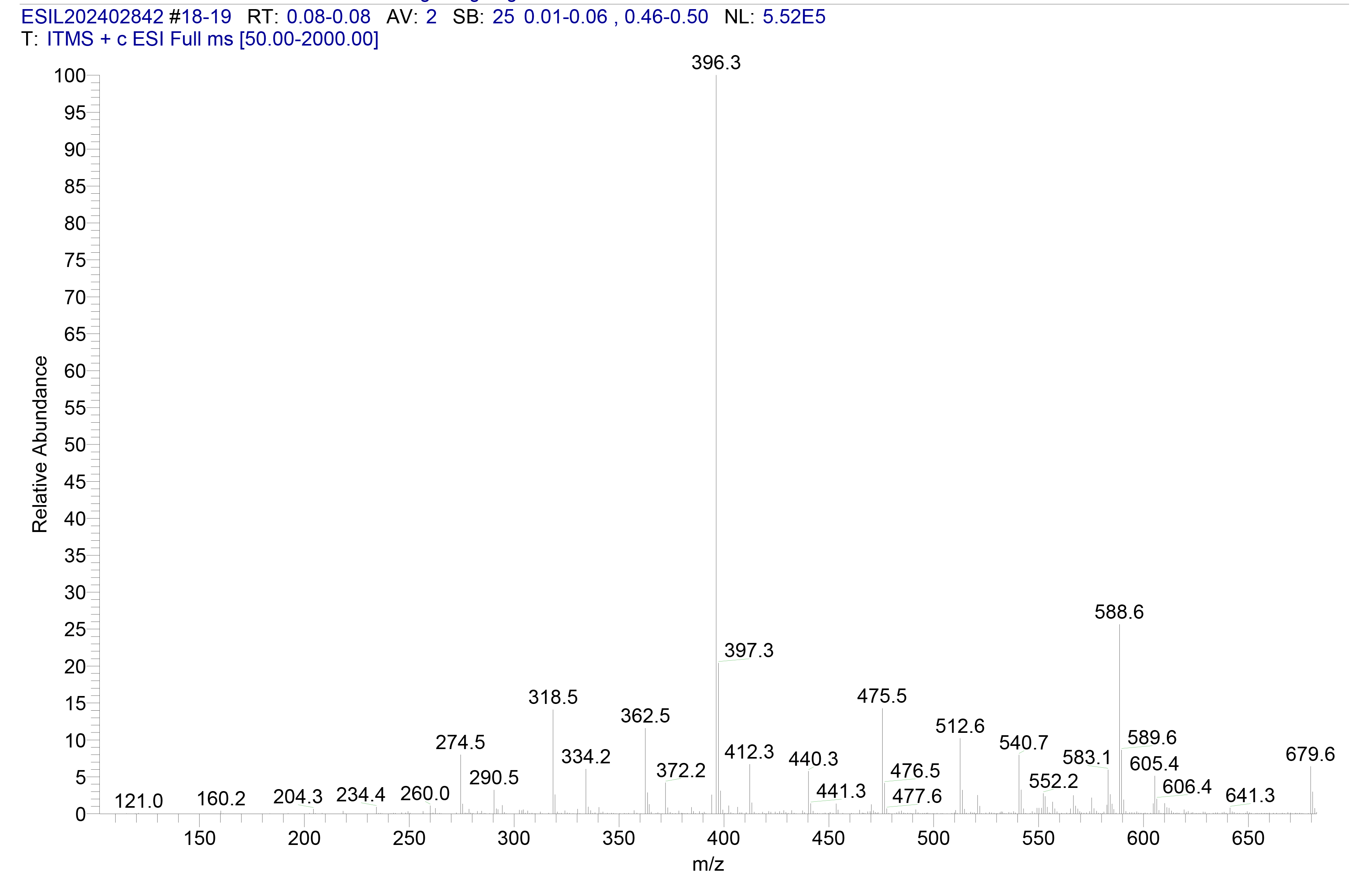

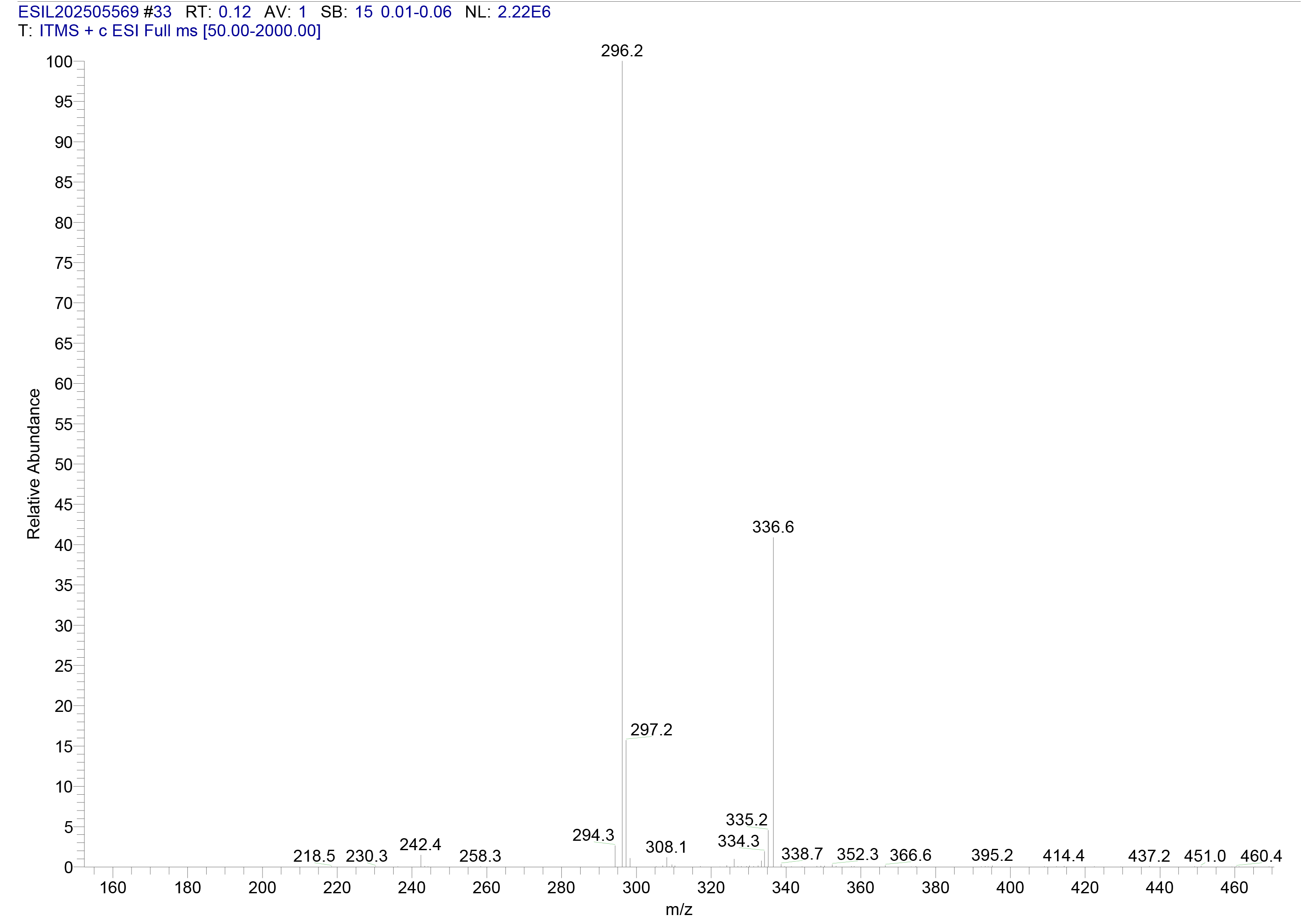


LRMS (ESI) of compound **11a**


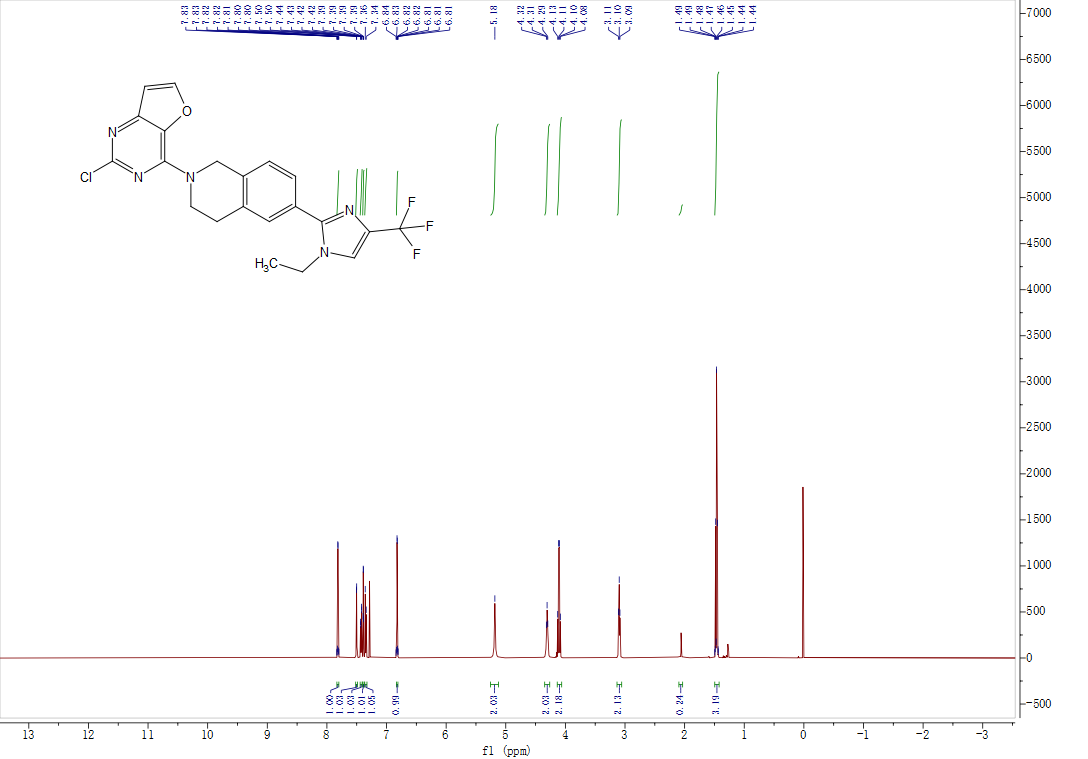


1H NMR of compound **12a**


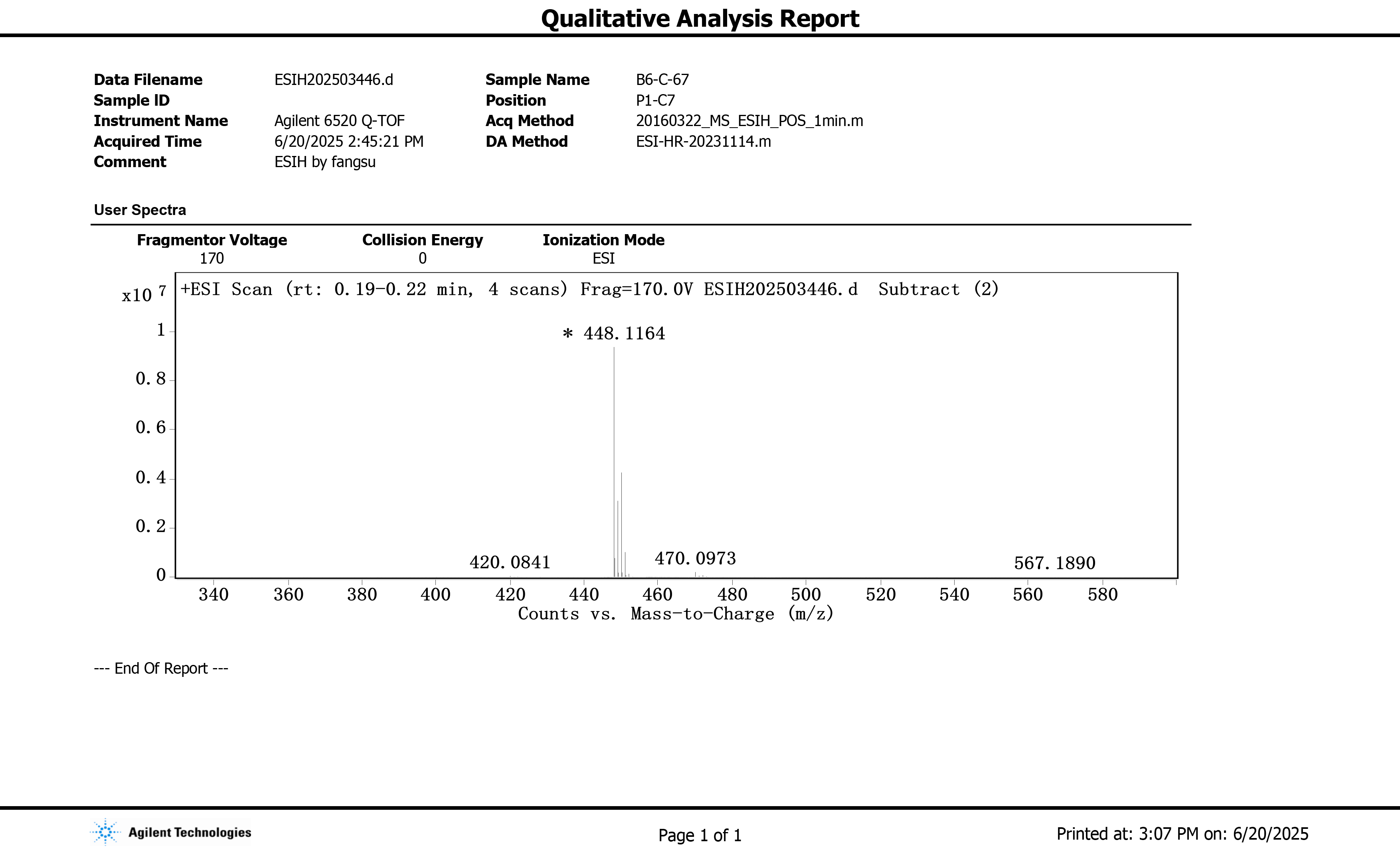


HRMS (ESI) of compound **12a**


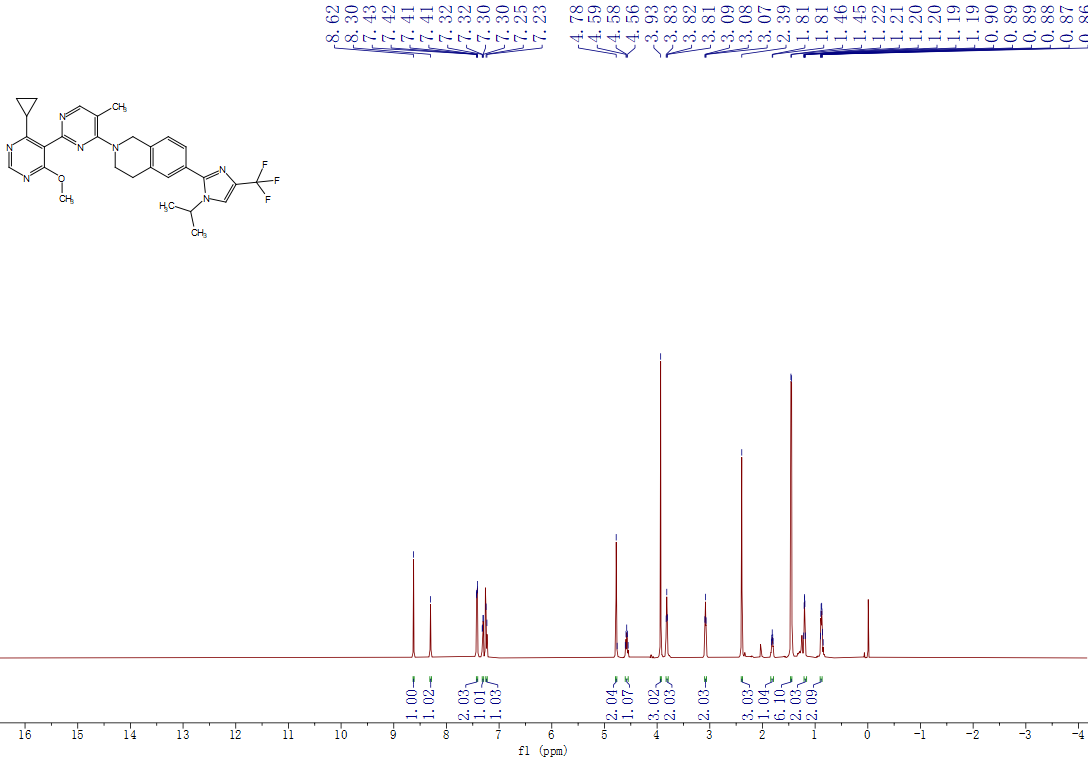


1H NMR of compound **13a**


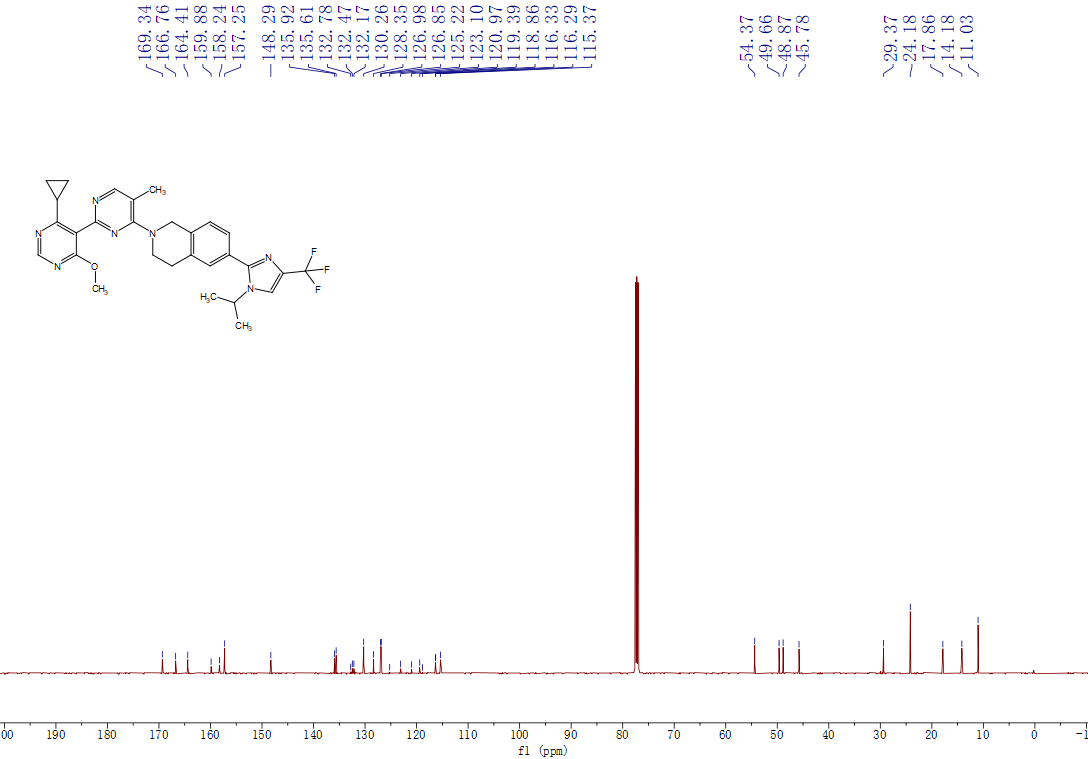


13C NMR of compound **13a**


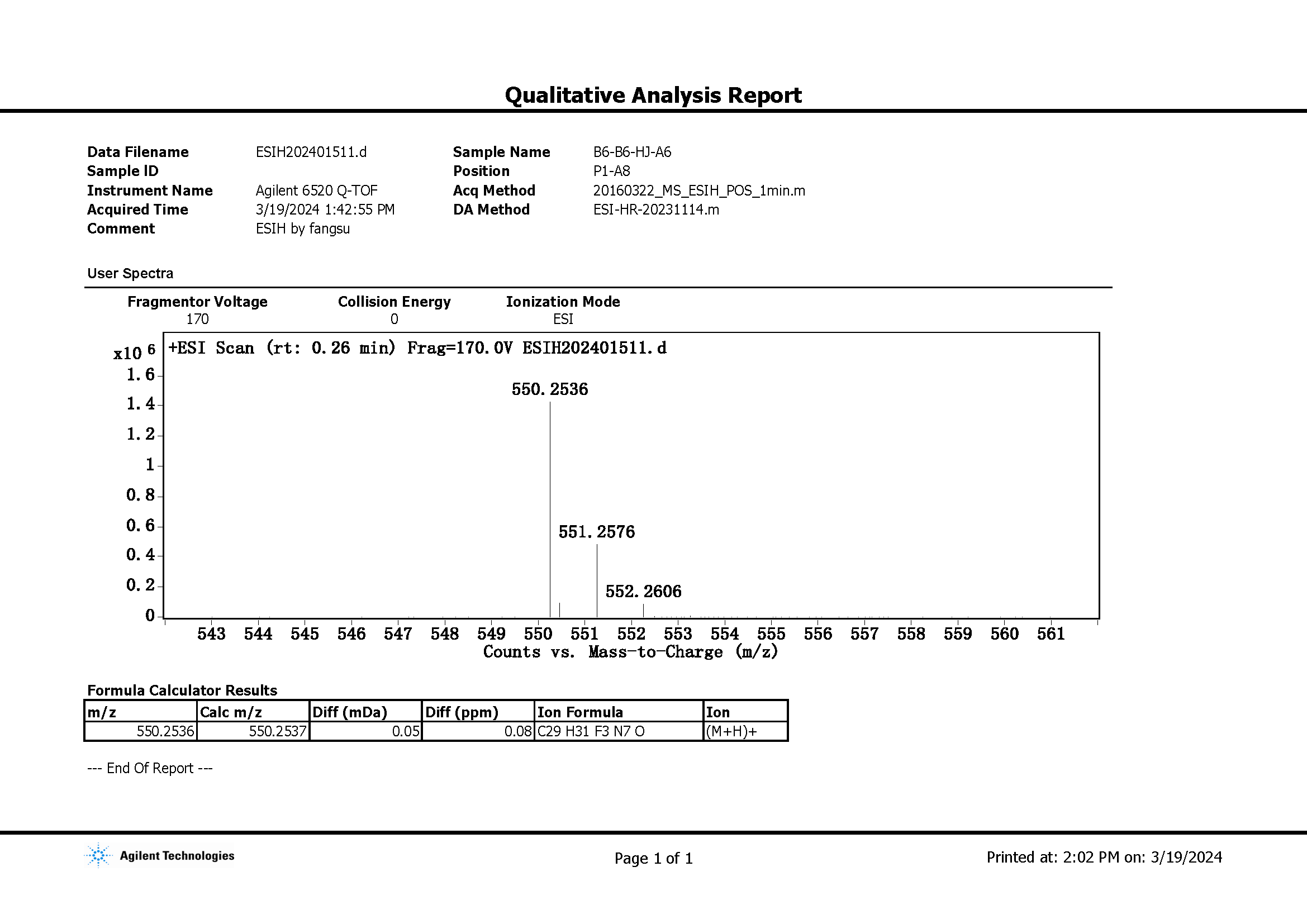


HRMS (ESI) of compound **13a**


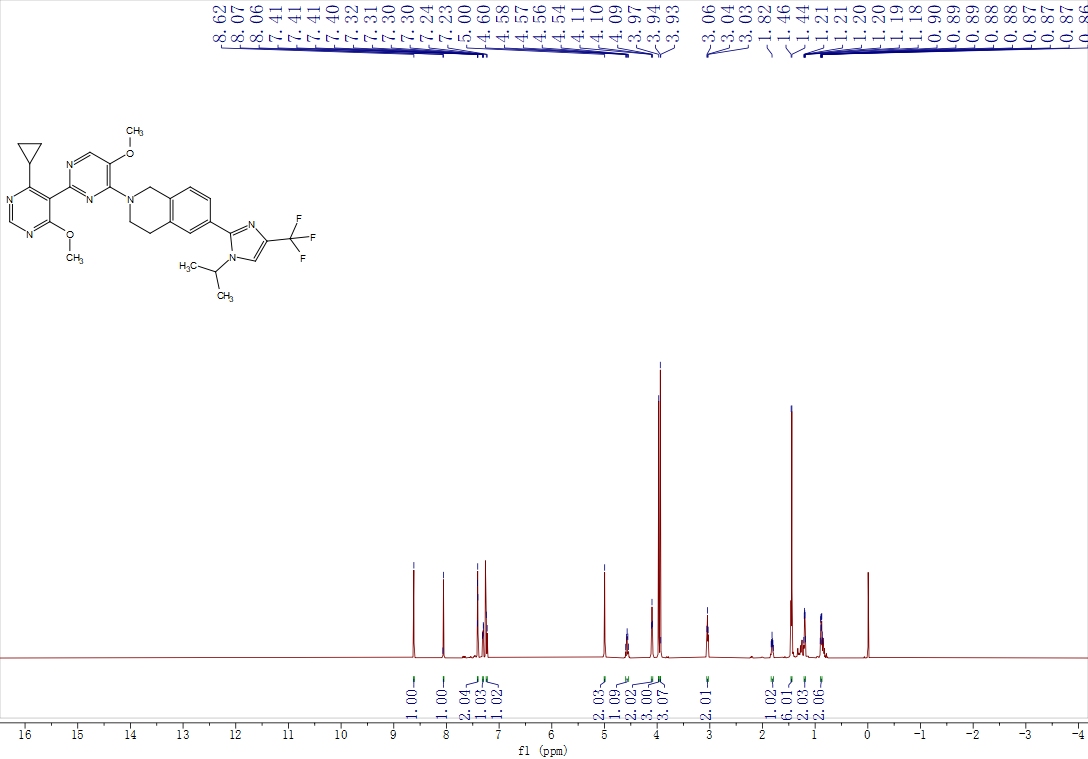


1H NMR of compound **13b**


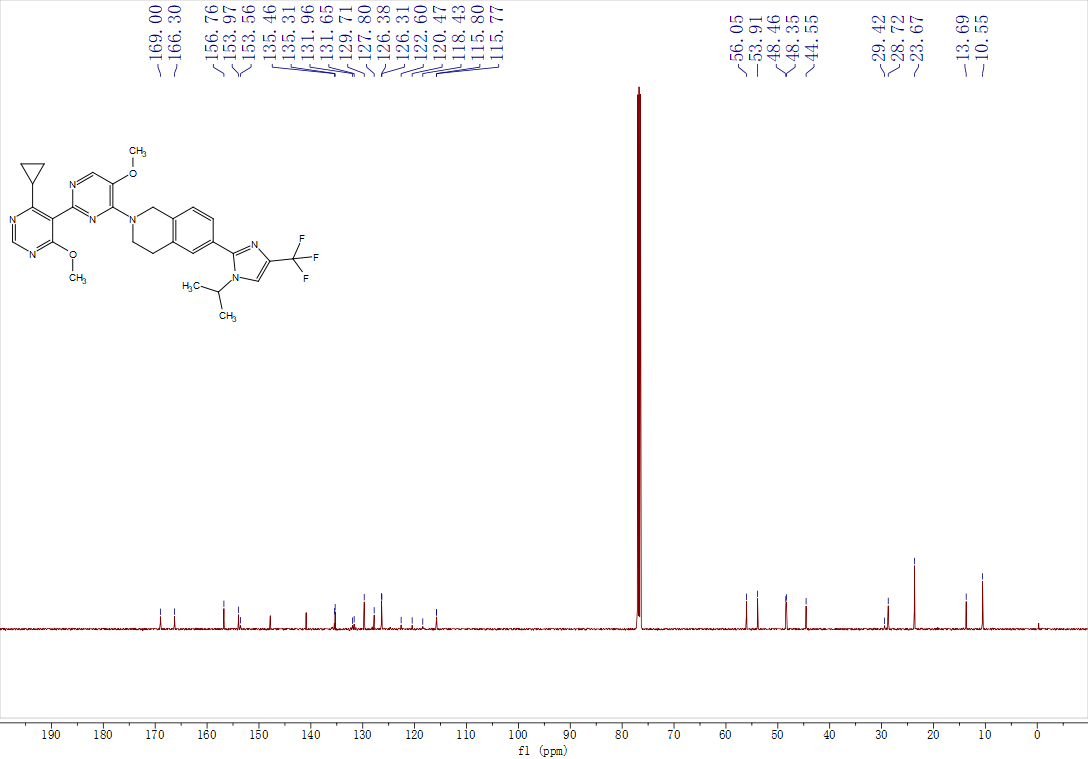


13C NMR of compound **13b**

**
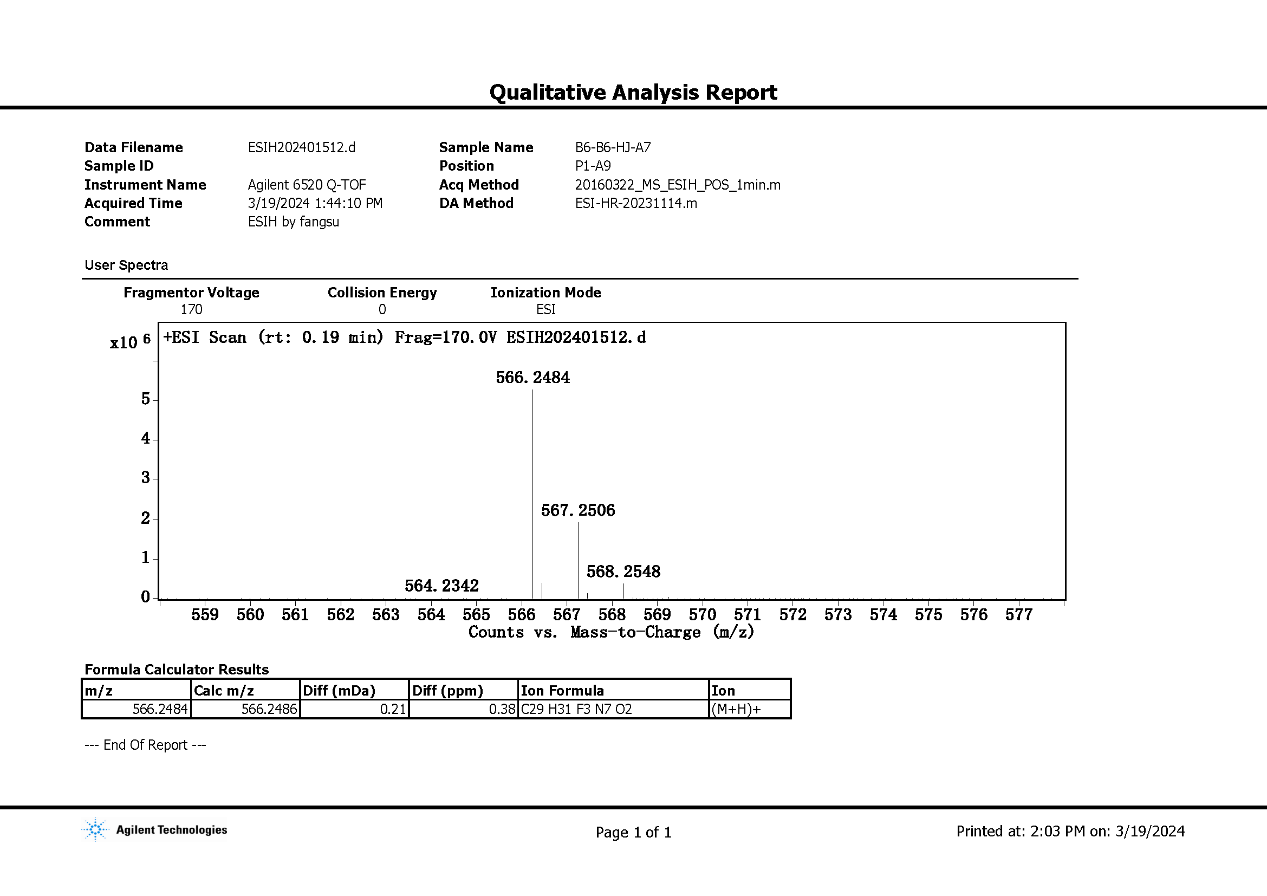
**

HRMS (ESI) of compound **13b**

**
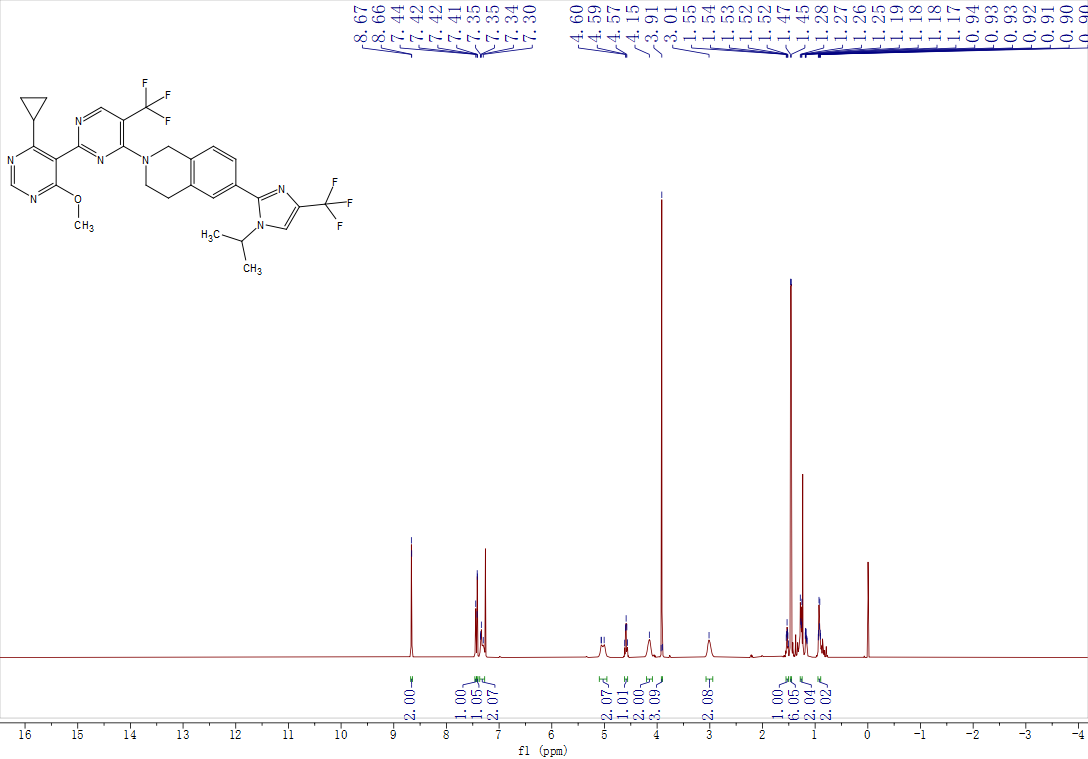
**

1H NMR of compound **13c**


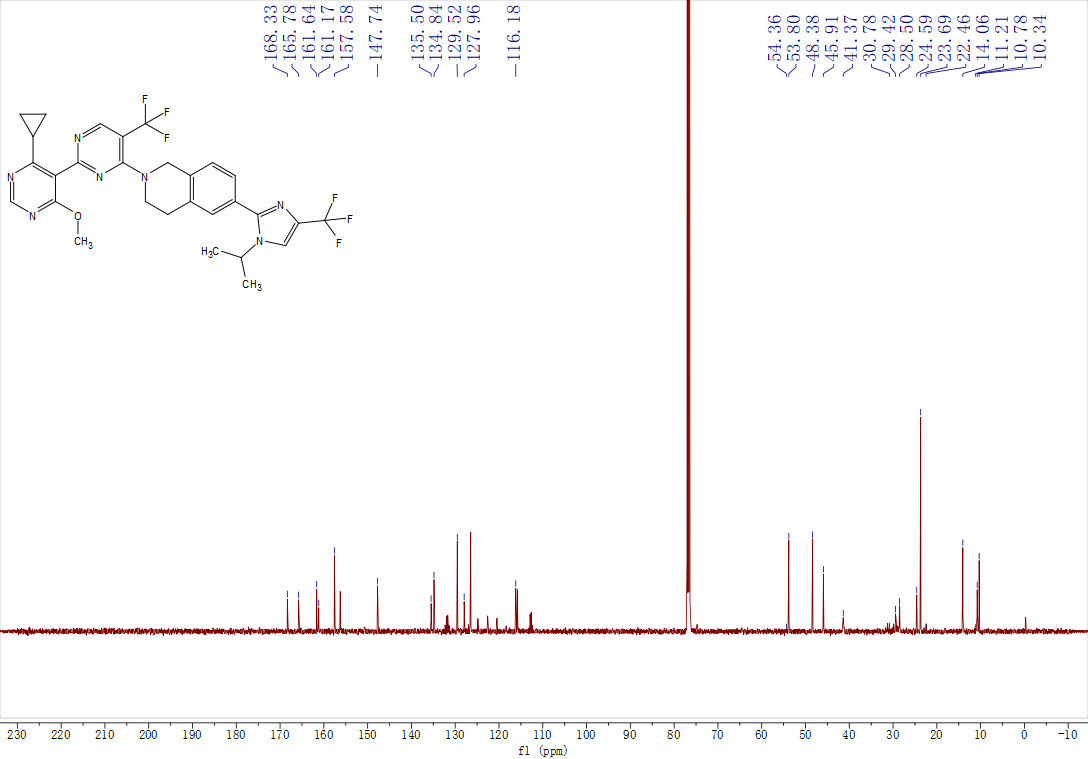


13C NMR of compound **13c**

**
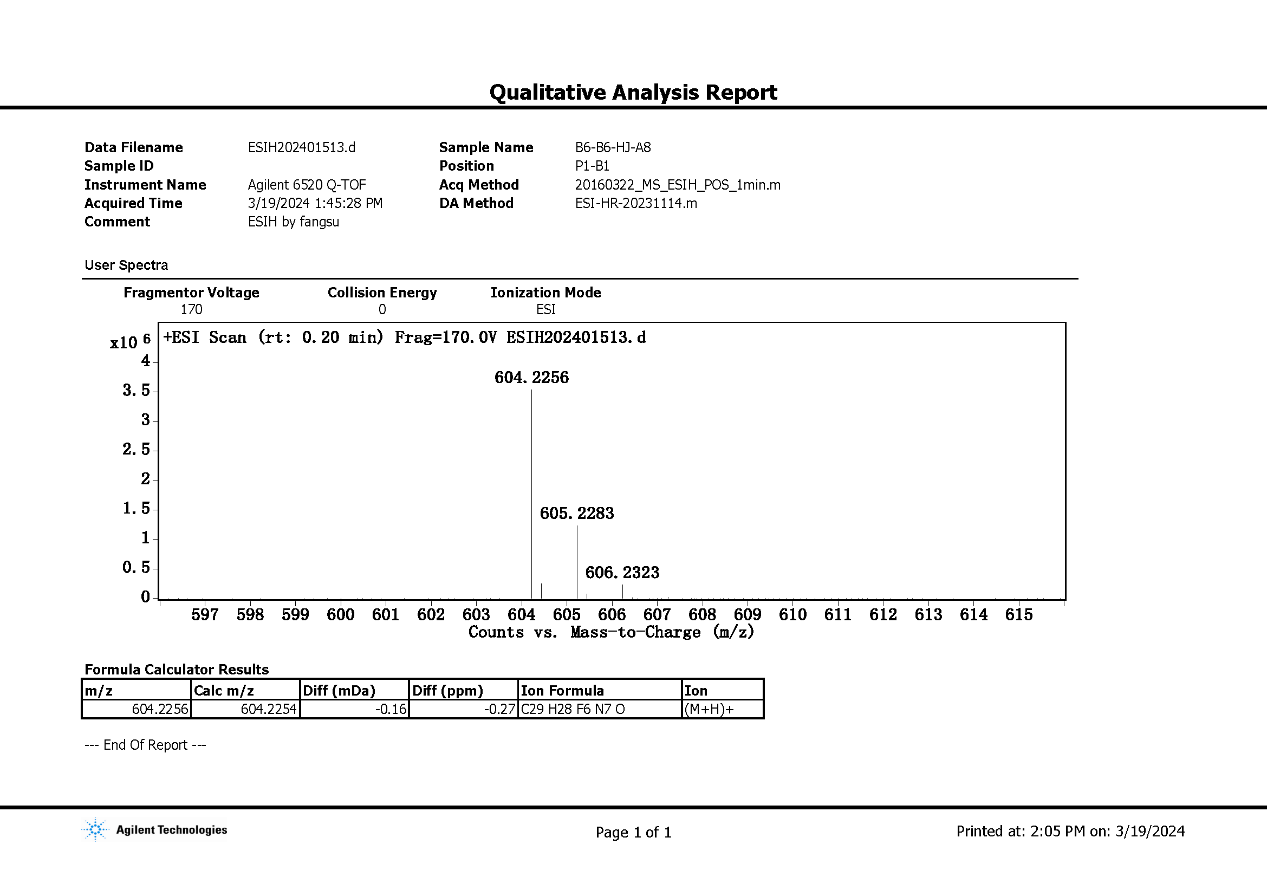
**

HRMS (ESI) of compound **13c**


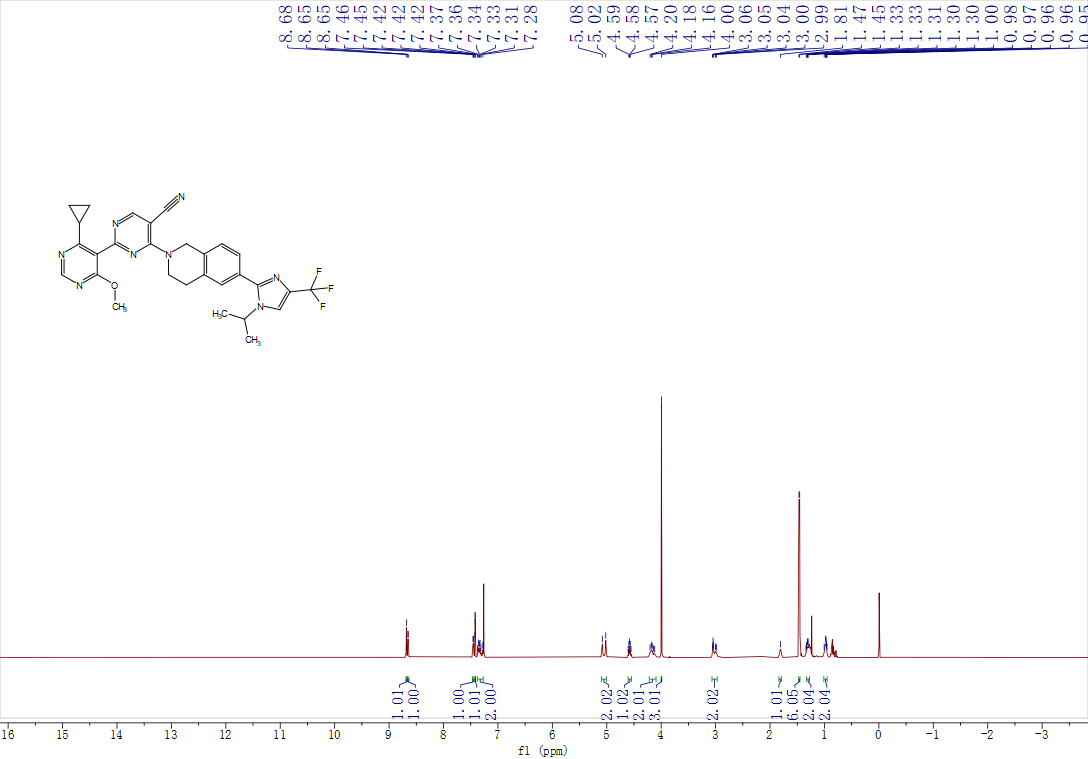


1H NMR of compound **13d**


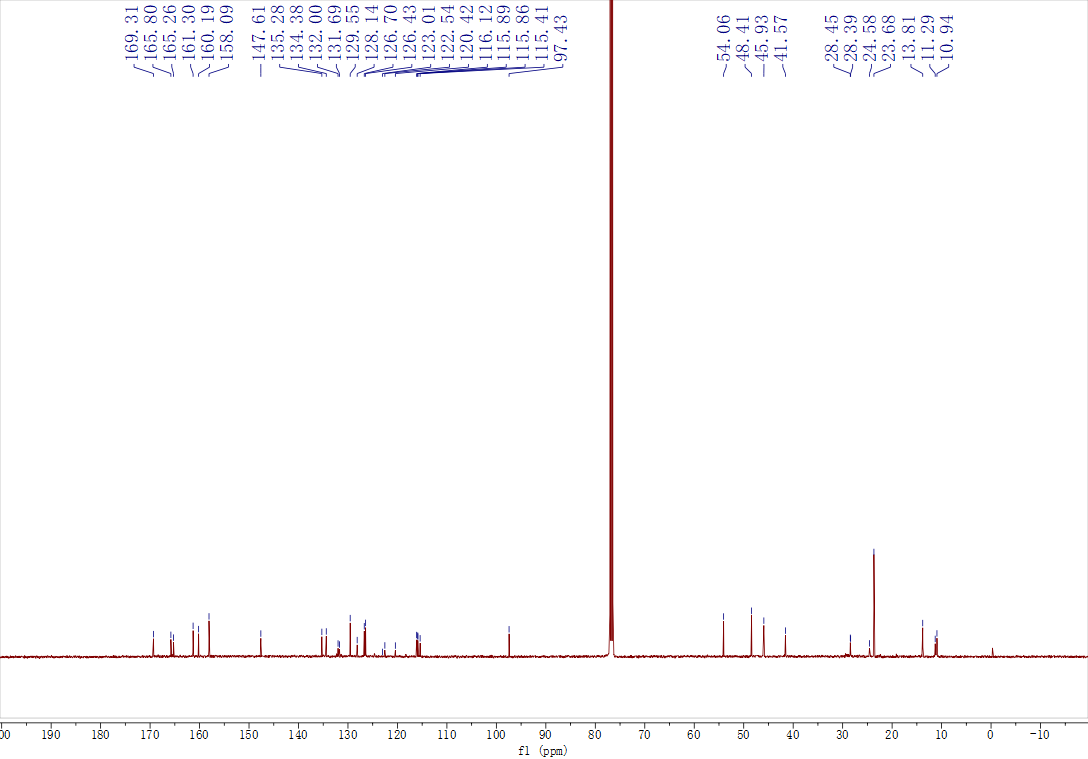


13C NMR of compound **13d**

**
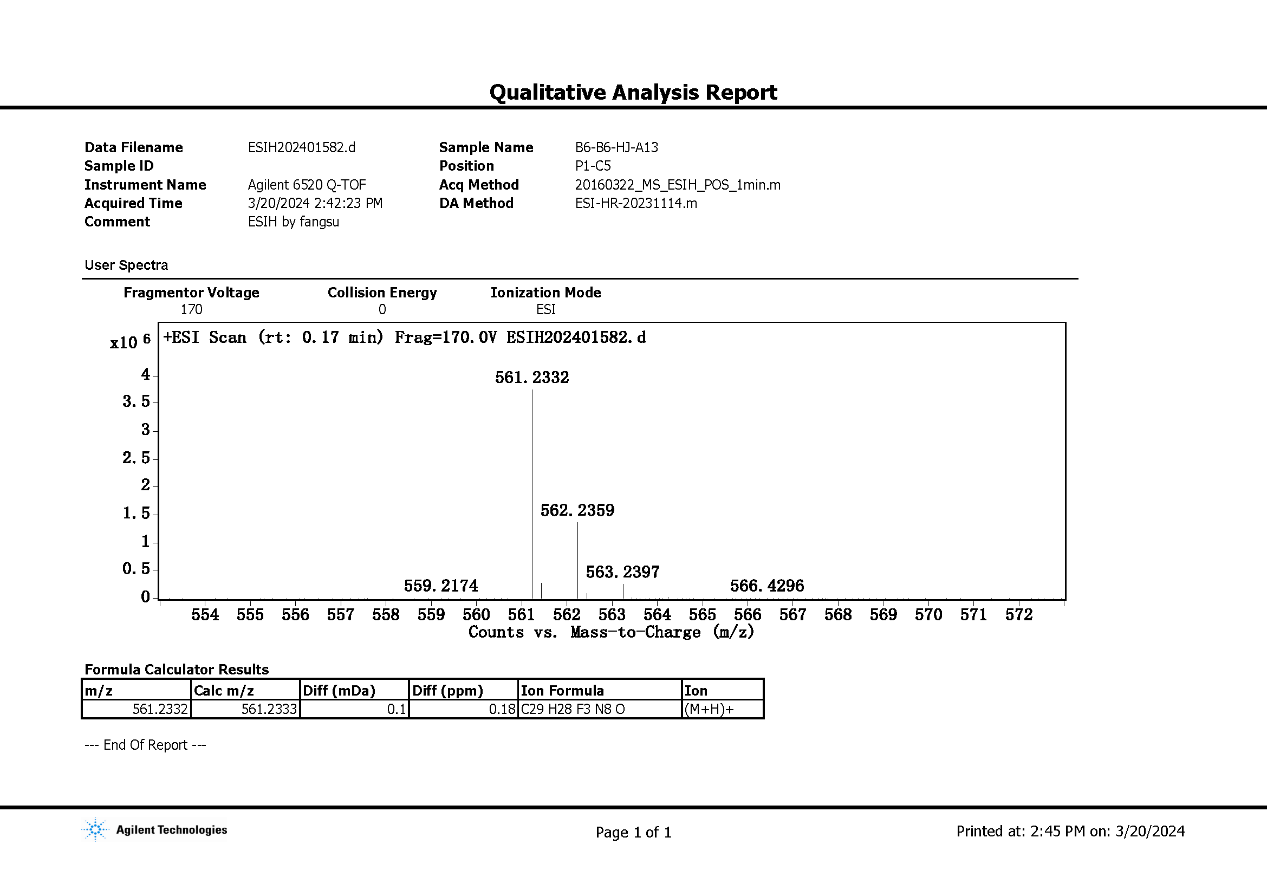
**

HRMS (ESI) of compound **13d**


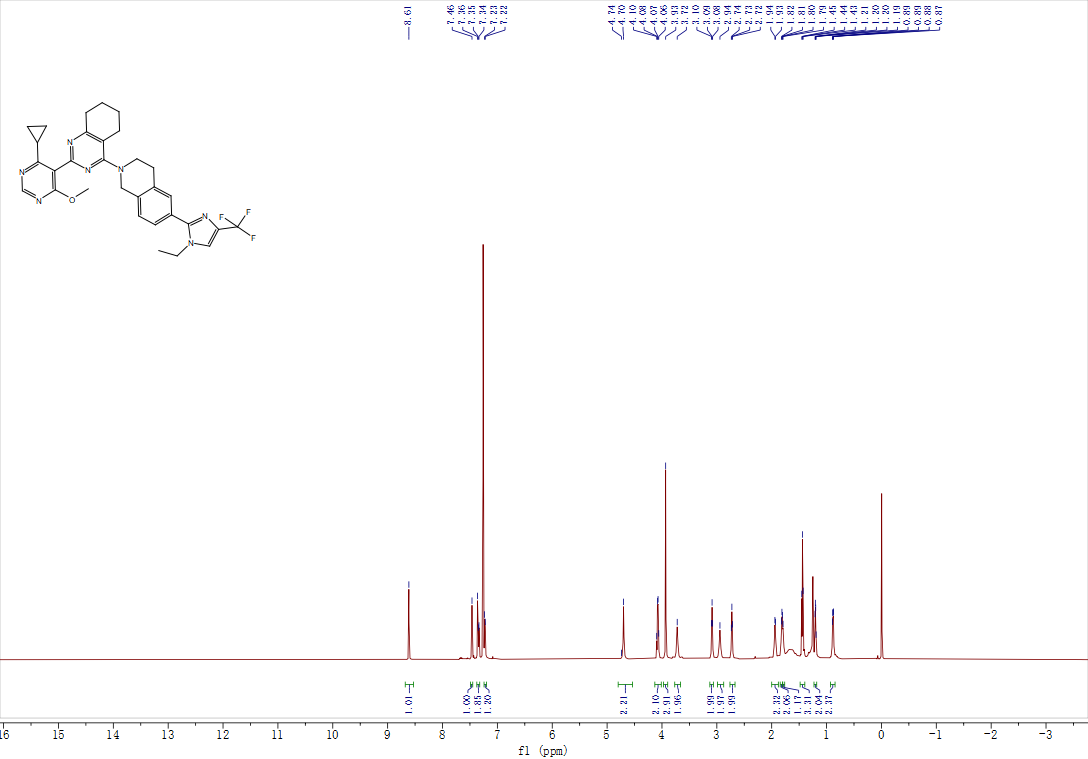


1H NMR of compound **13e**


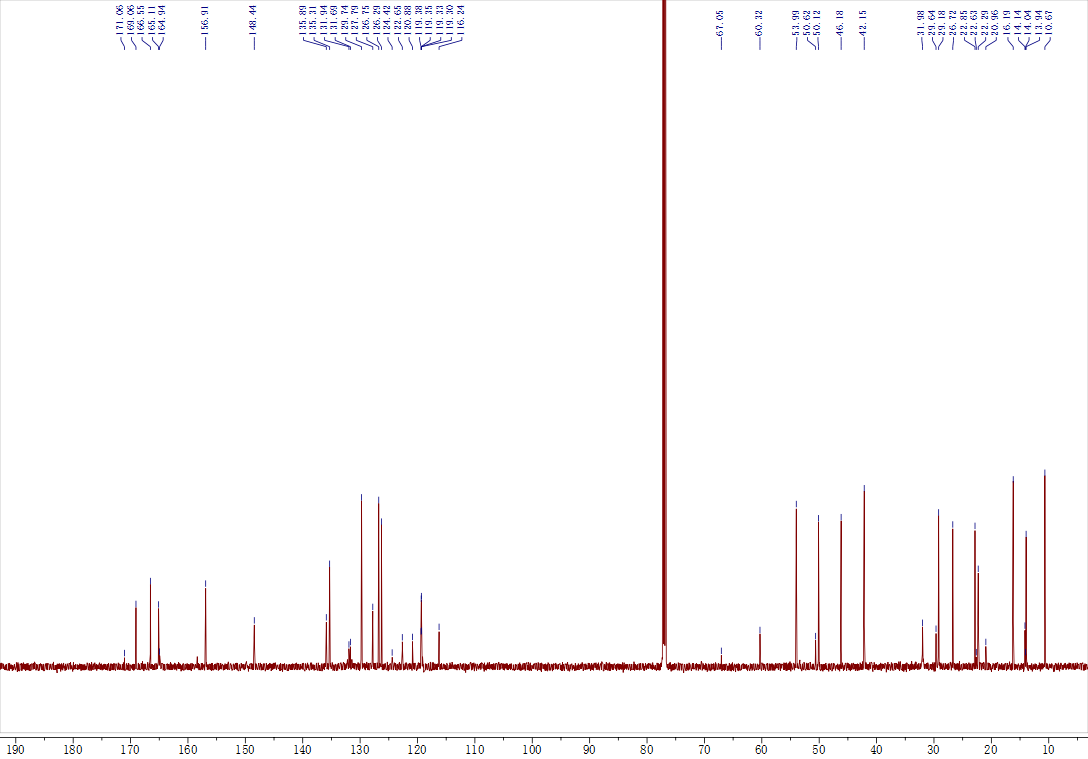


13C NMR of compound **13e**

**
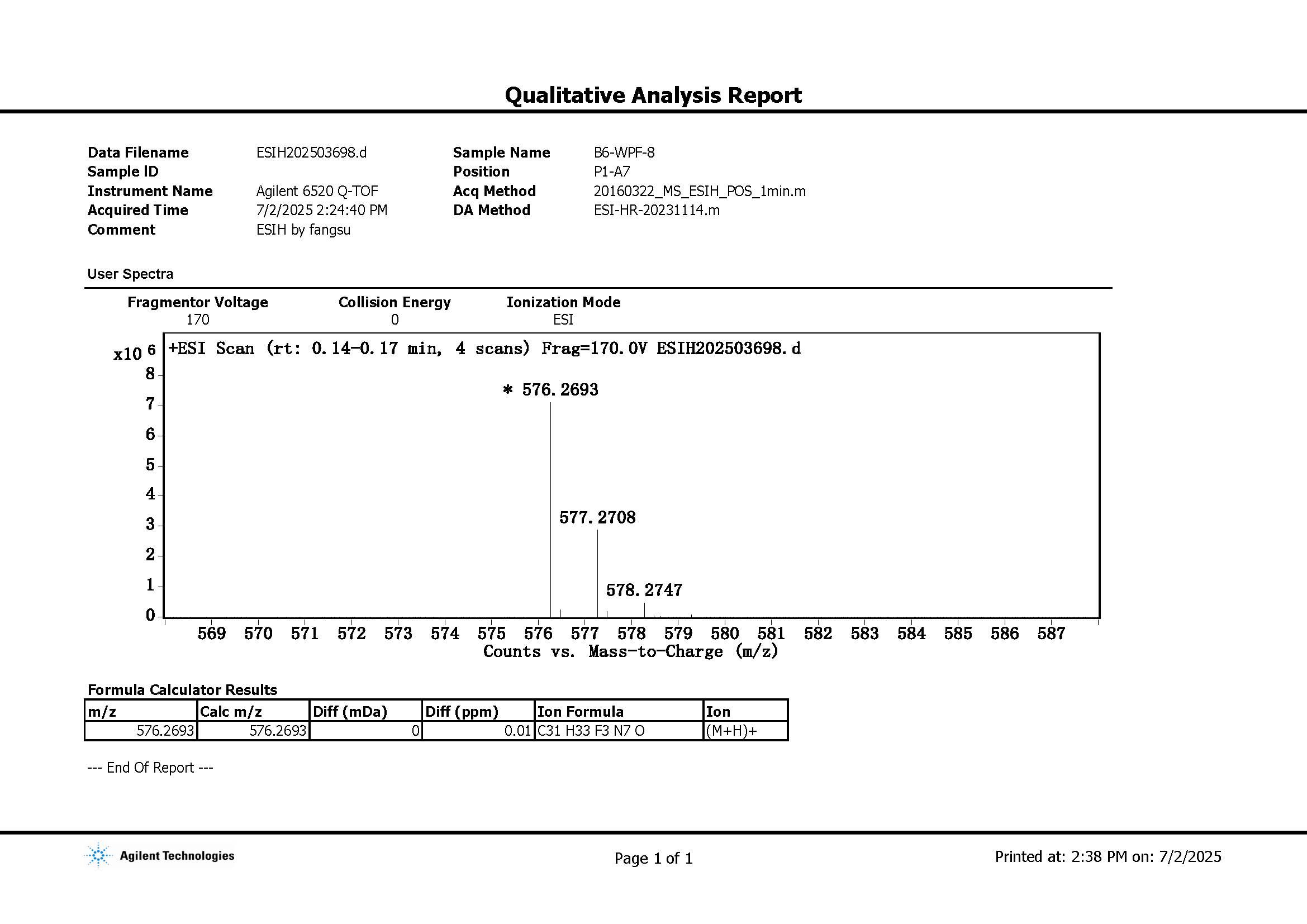
**

HRMS (ESI) of compound **13e**

**
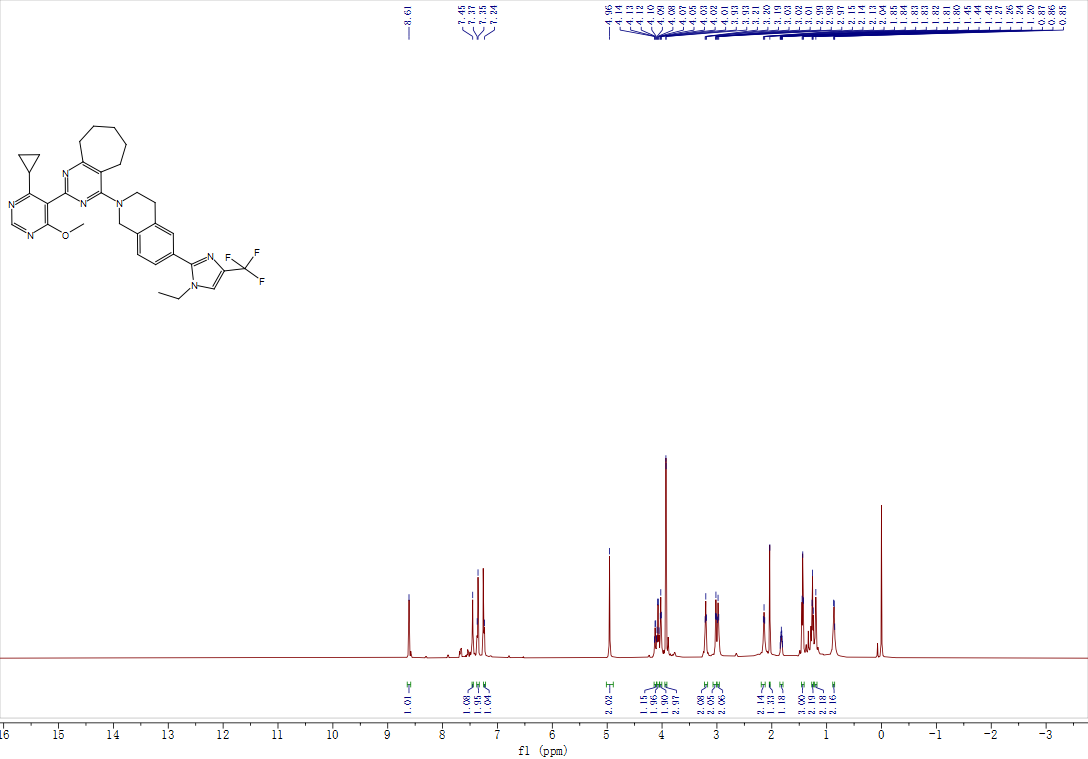
**

1H NMR of compound **13f**


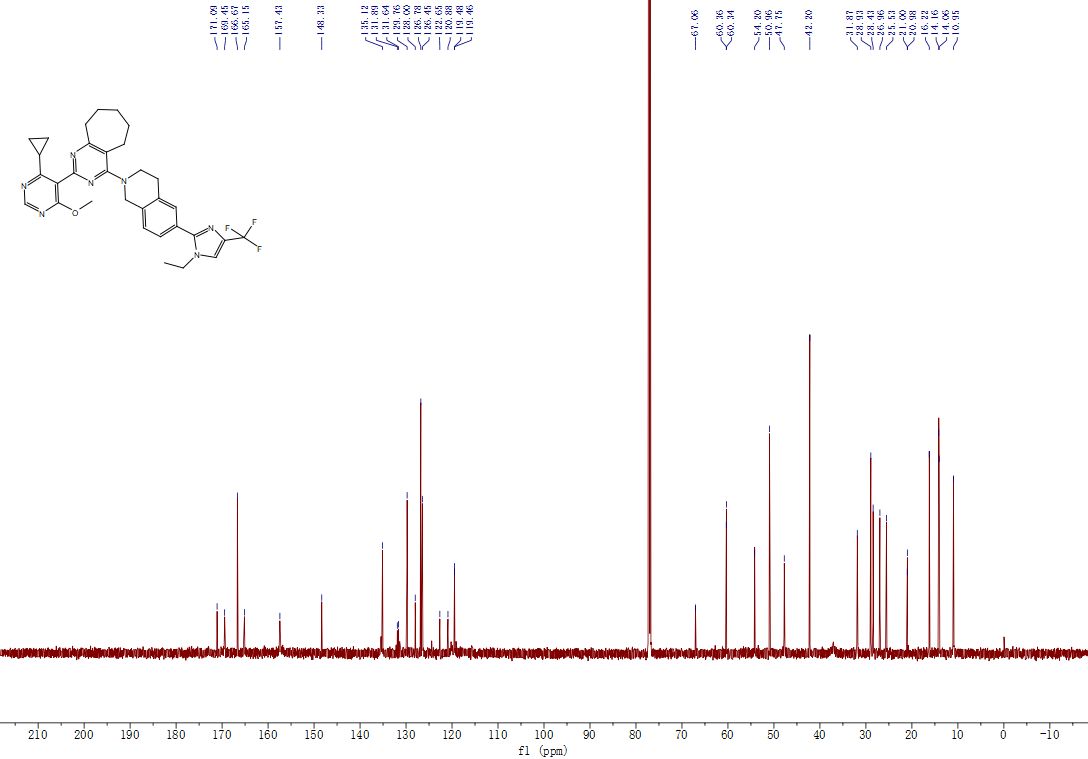


13C NMR of compound **13f**


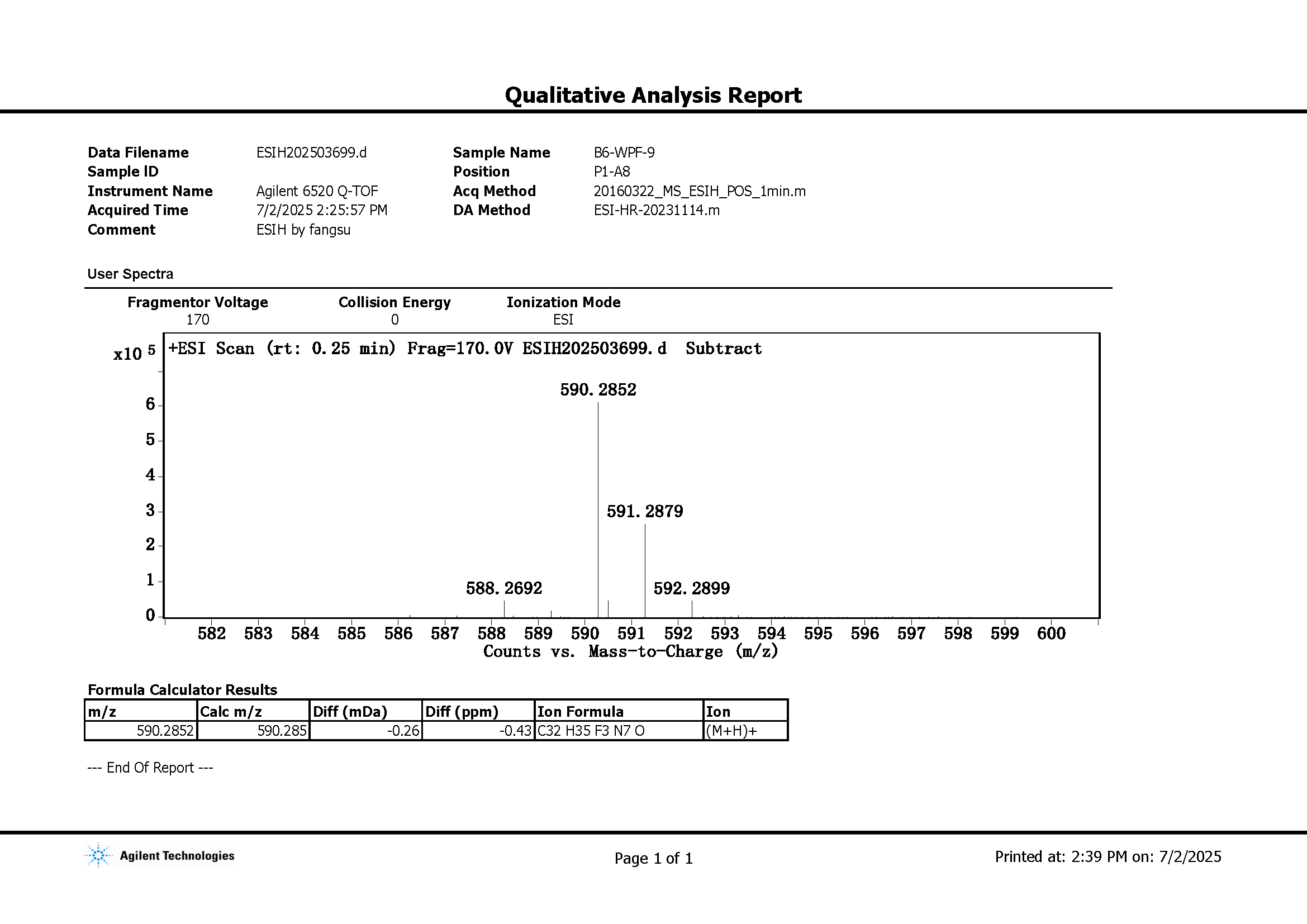


HRMS (ESI) of compound **13f**

**
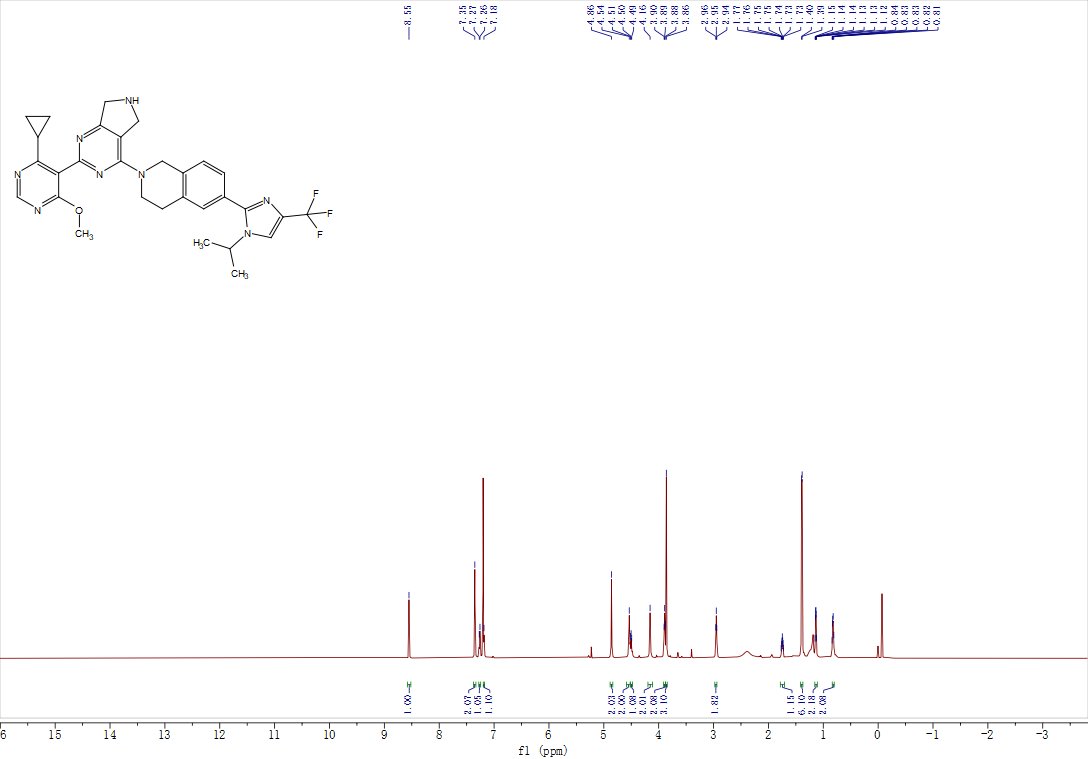
**

1H NMR of compound **13g**

**
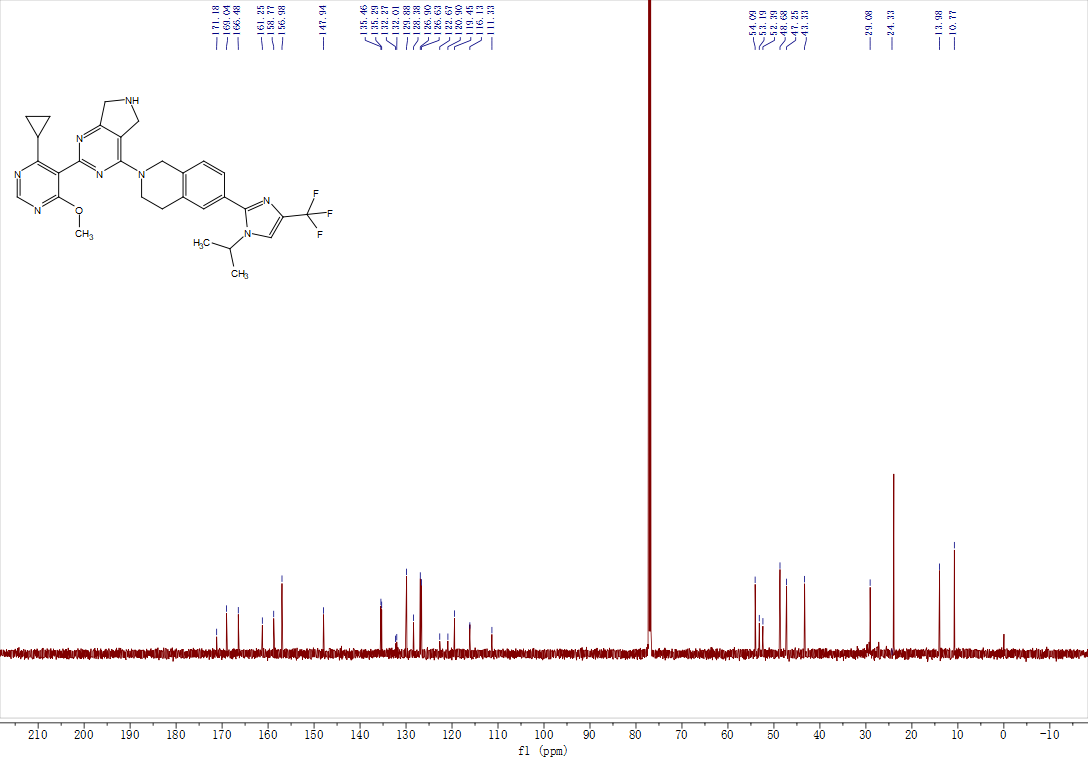
**

13C NMR of compound **13g**

**
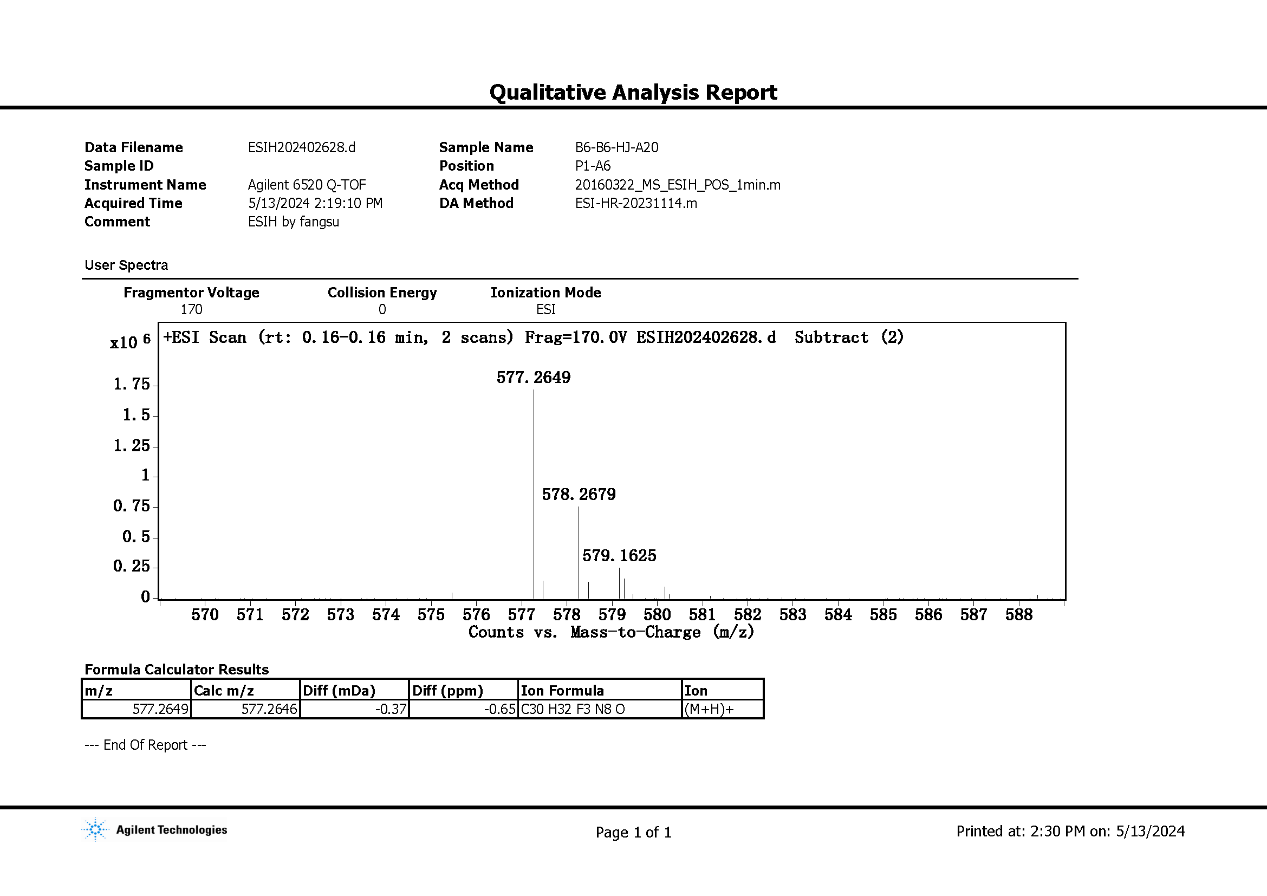
**

HRMS (ESI) of compound **13g**


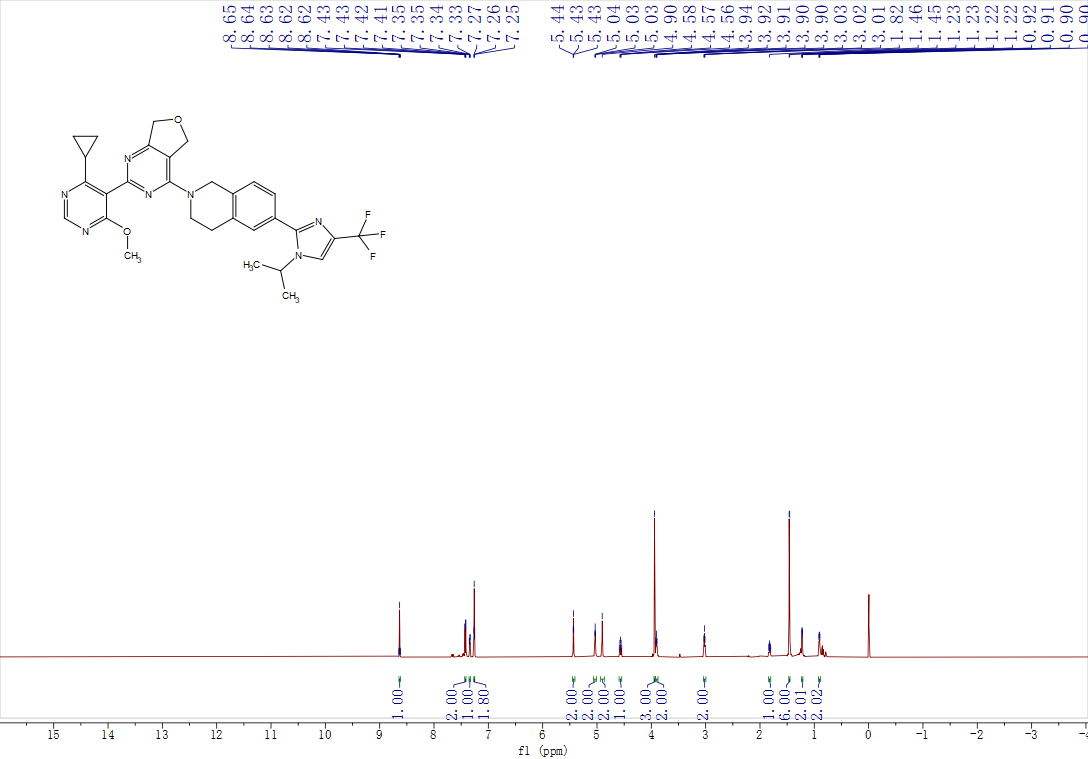


1H NMR of compound **13h**


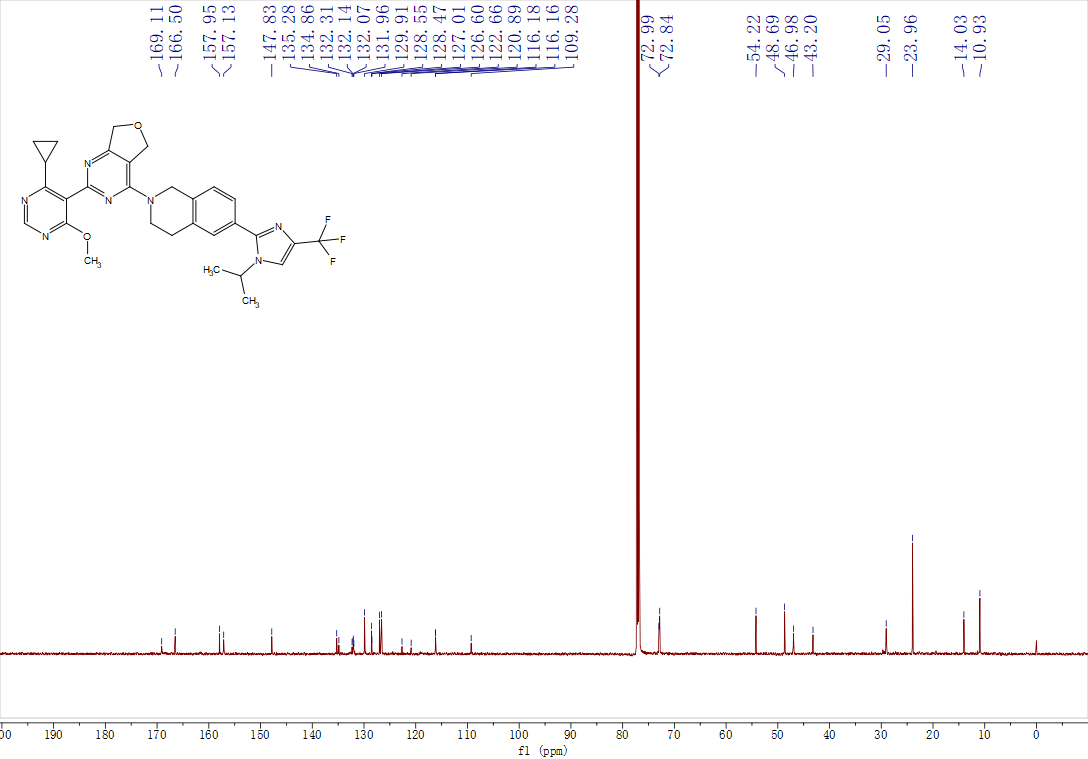


13C NMR of compound **13h
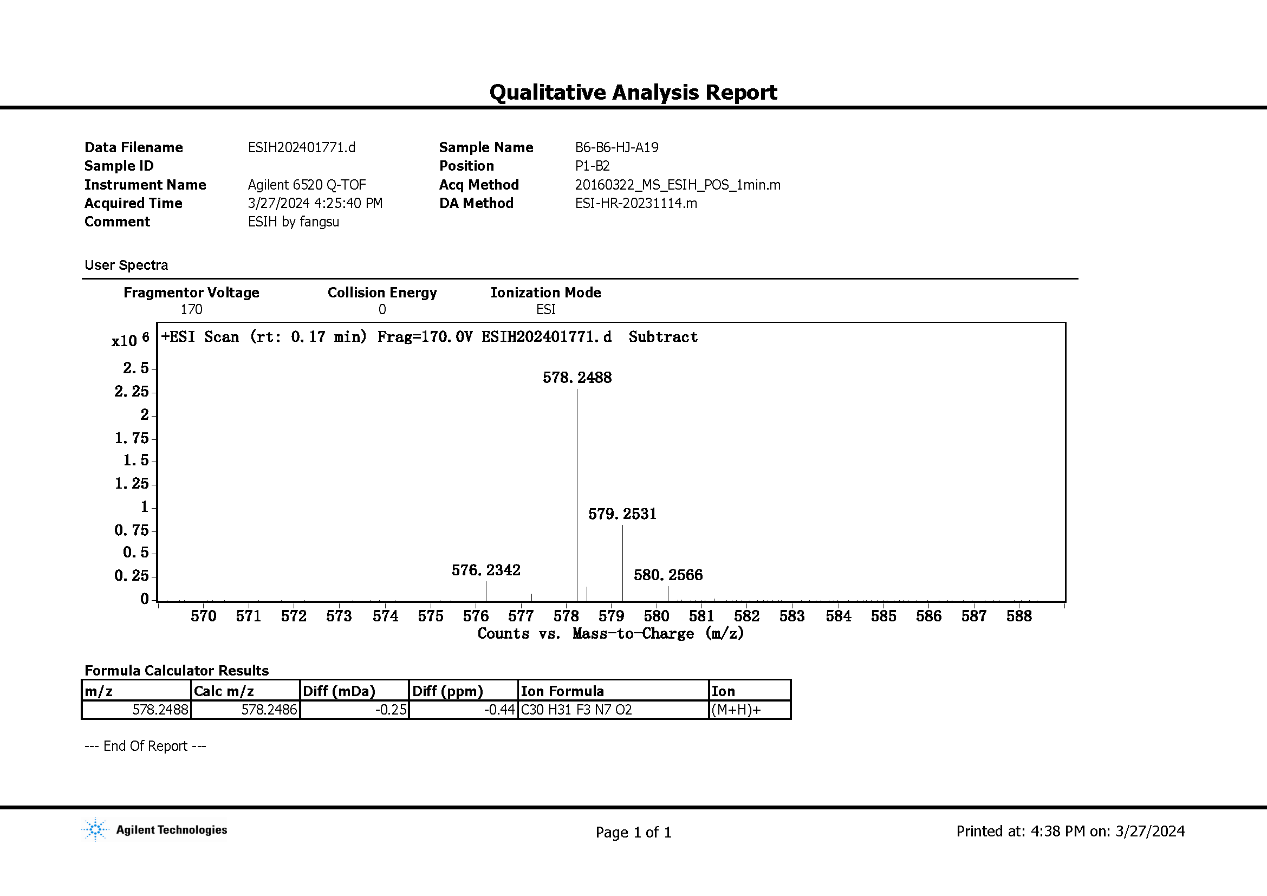
**

HRMS (ESI) of compound **13h**


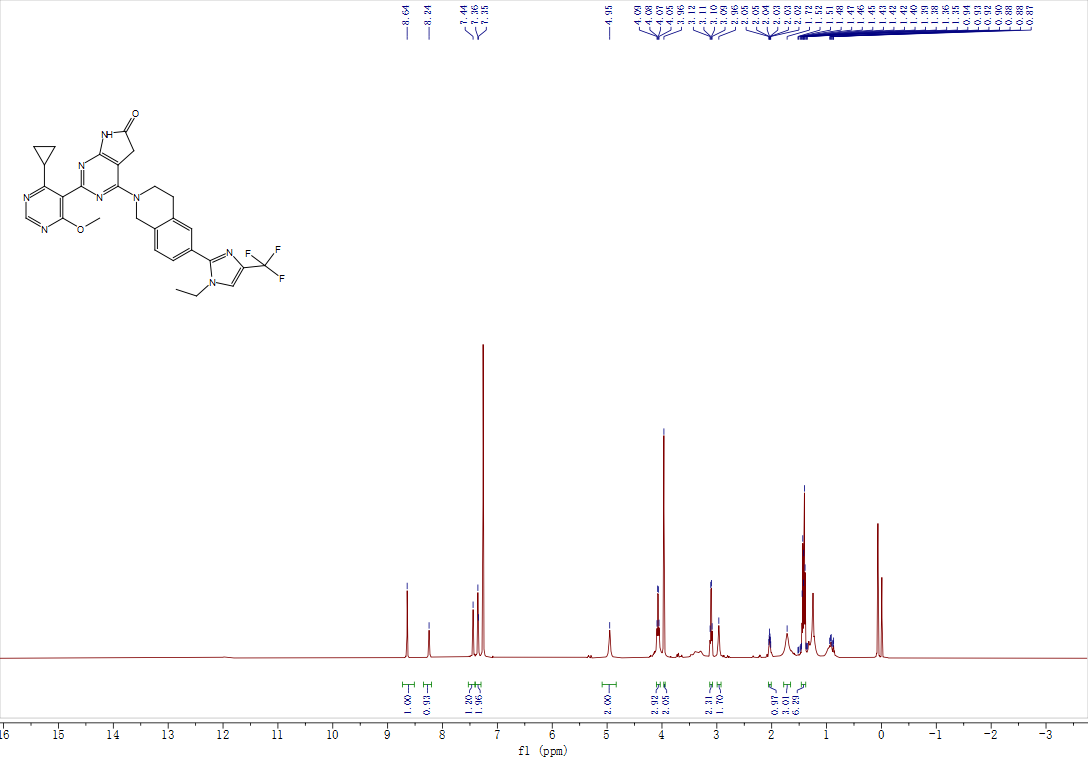


1H NMR of compound **13i**

**
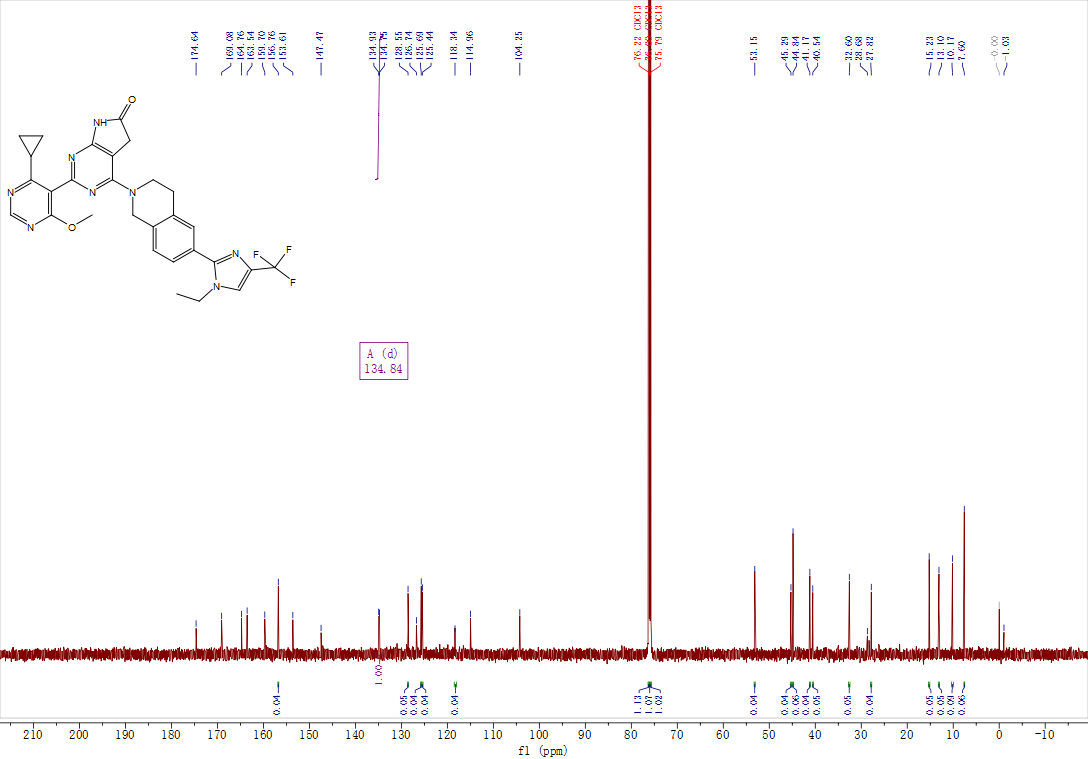
**

13C NMR of compound **13i**


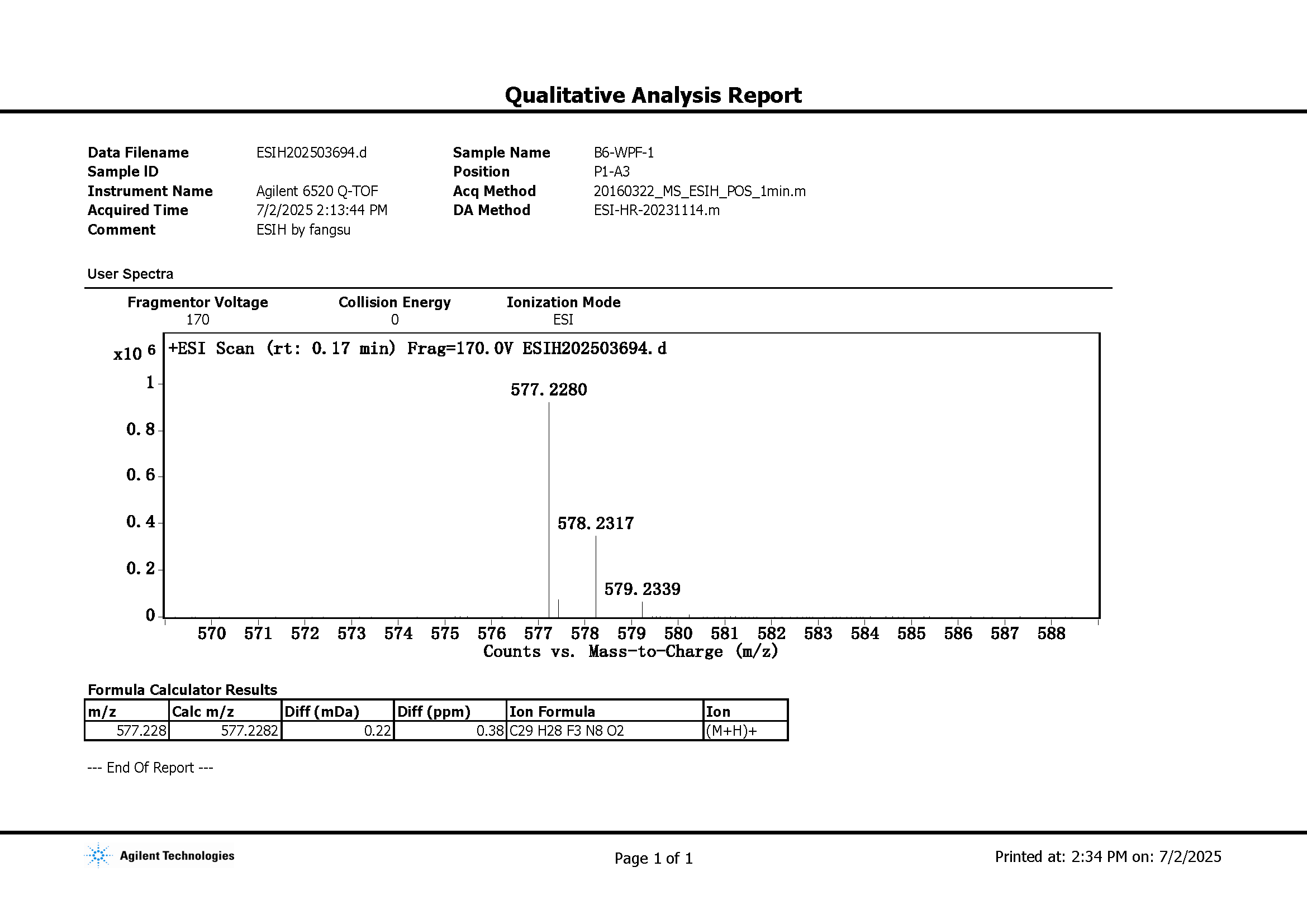


HRMS (ESI) of compound **13i**

**
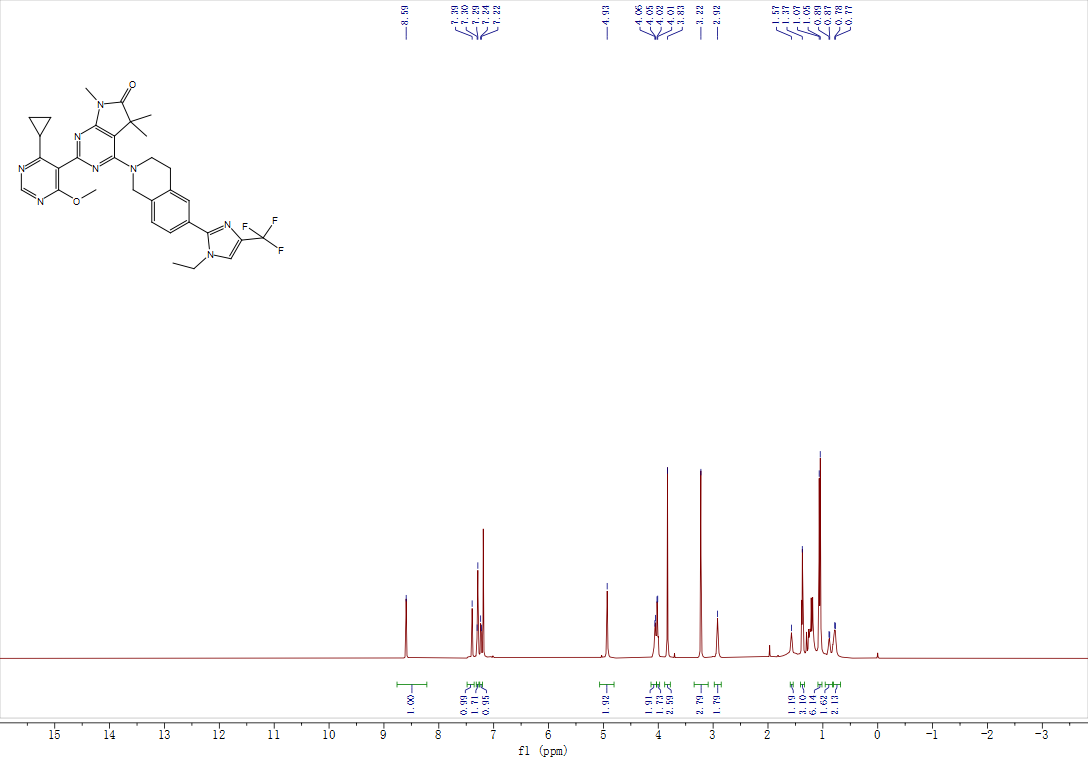
**

1H NMR of compound **13j**

13C NMR of compound **13j**


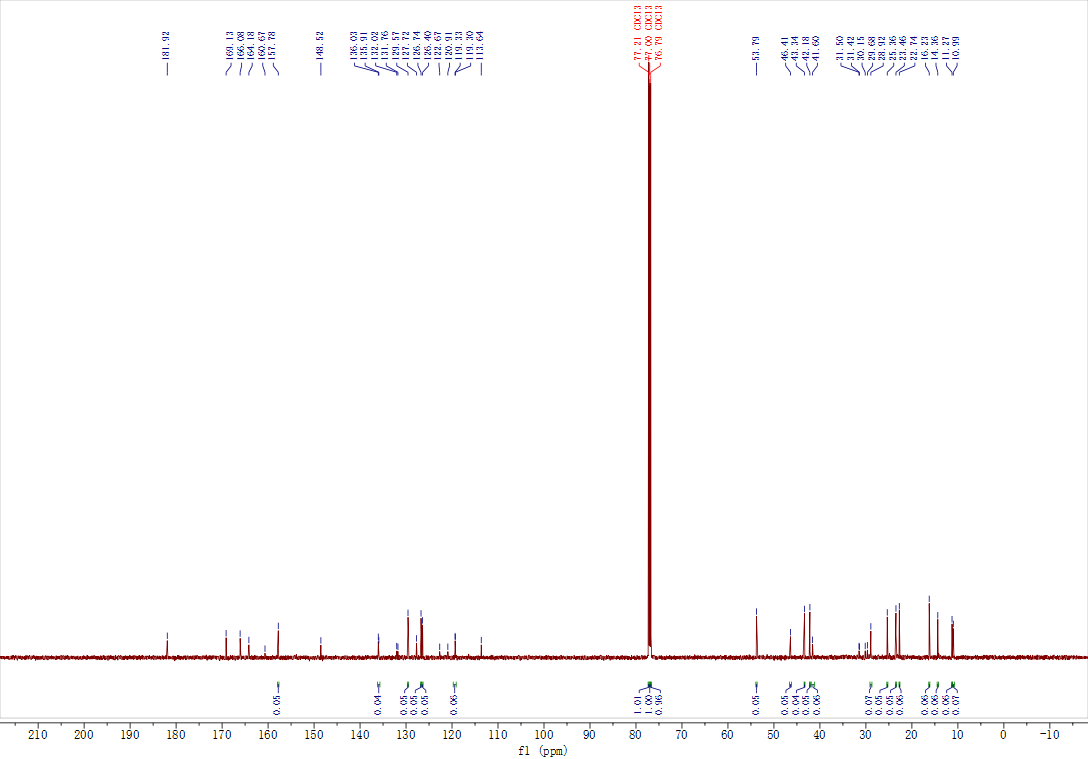

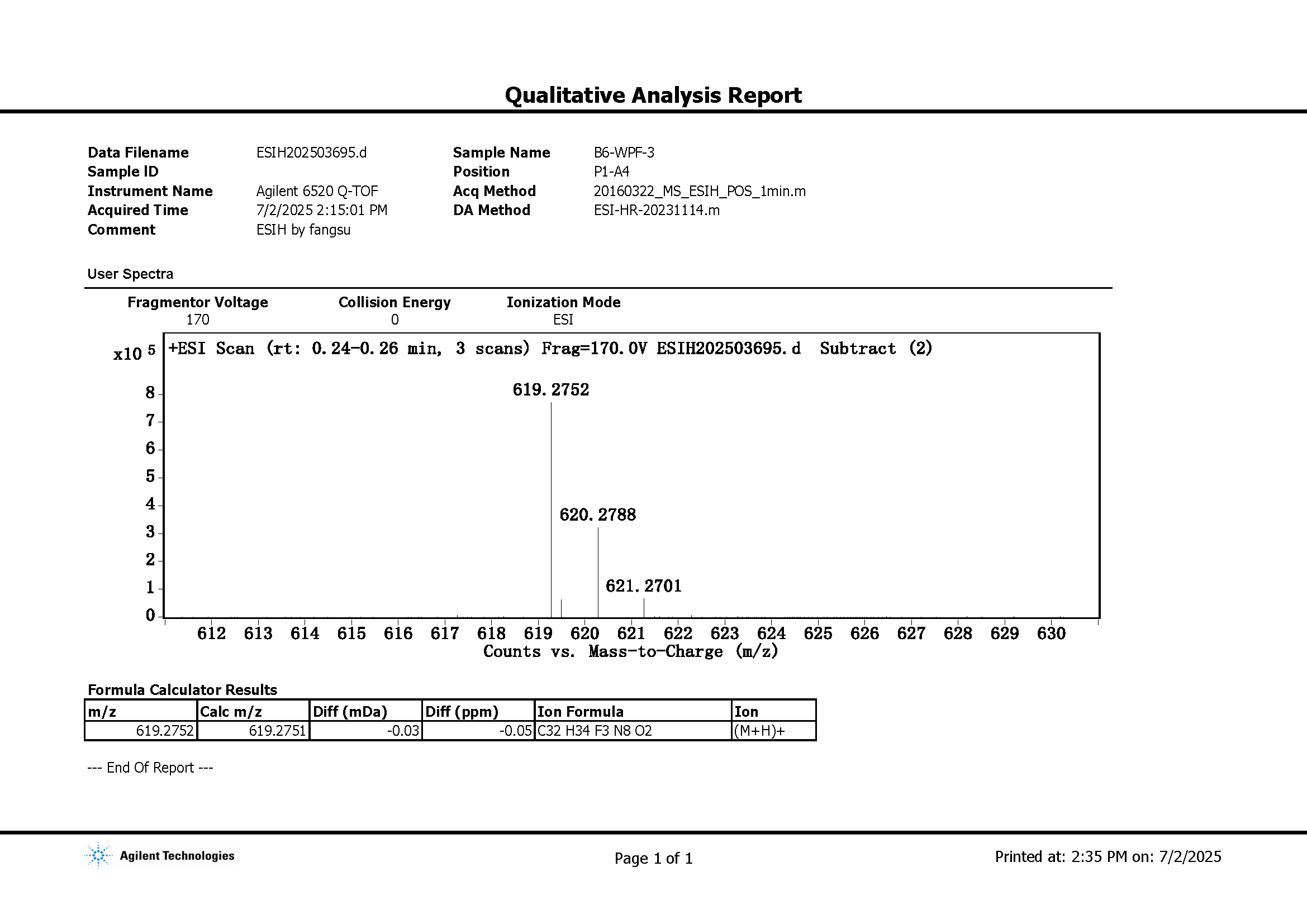


HRMS (ESI) of compound **13j**


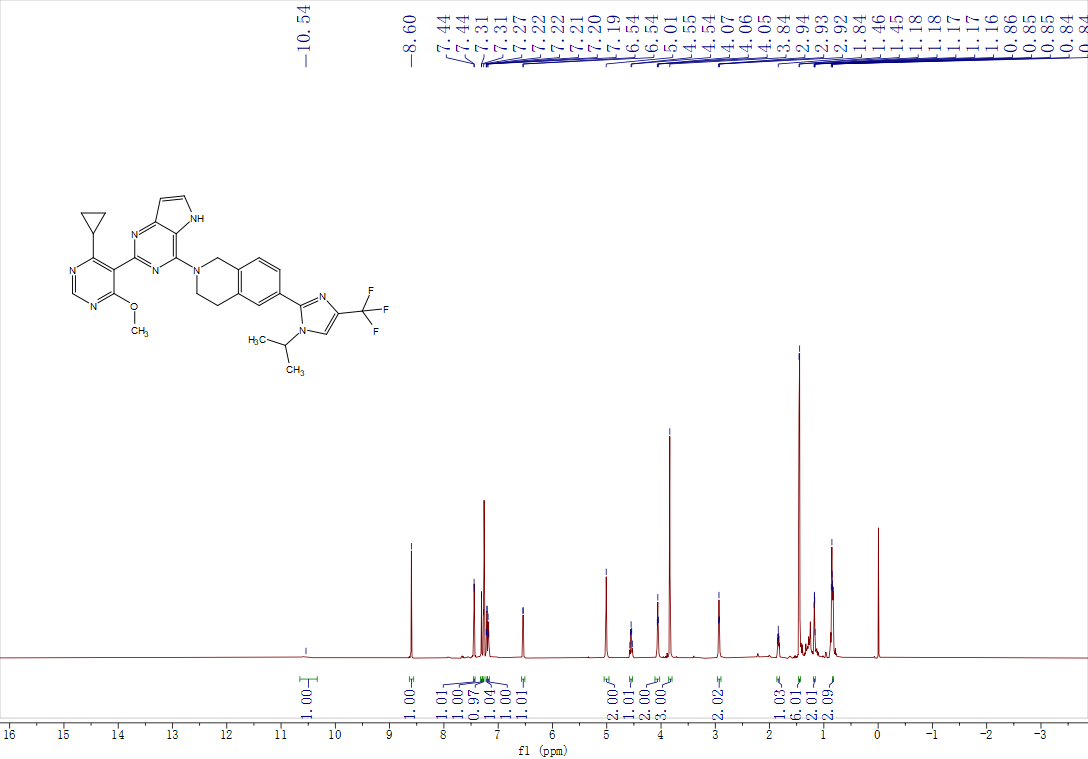


1H NMR of compound **13k**


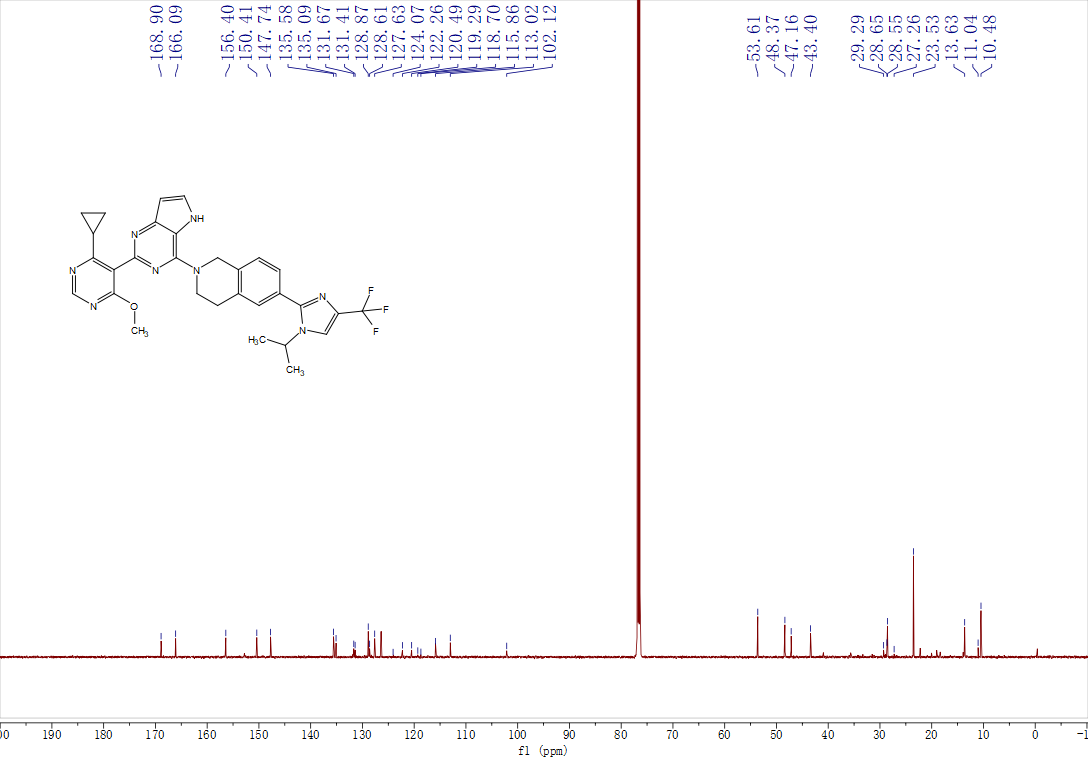


13C NMR of compound **13k**


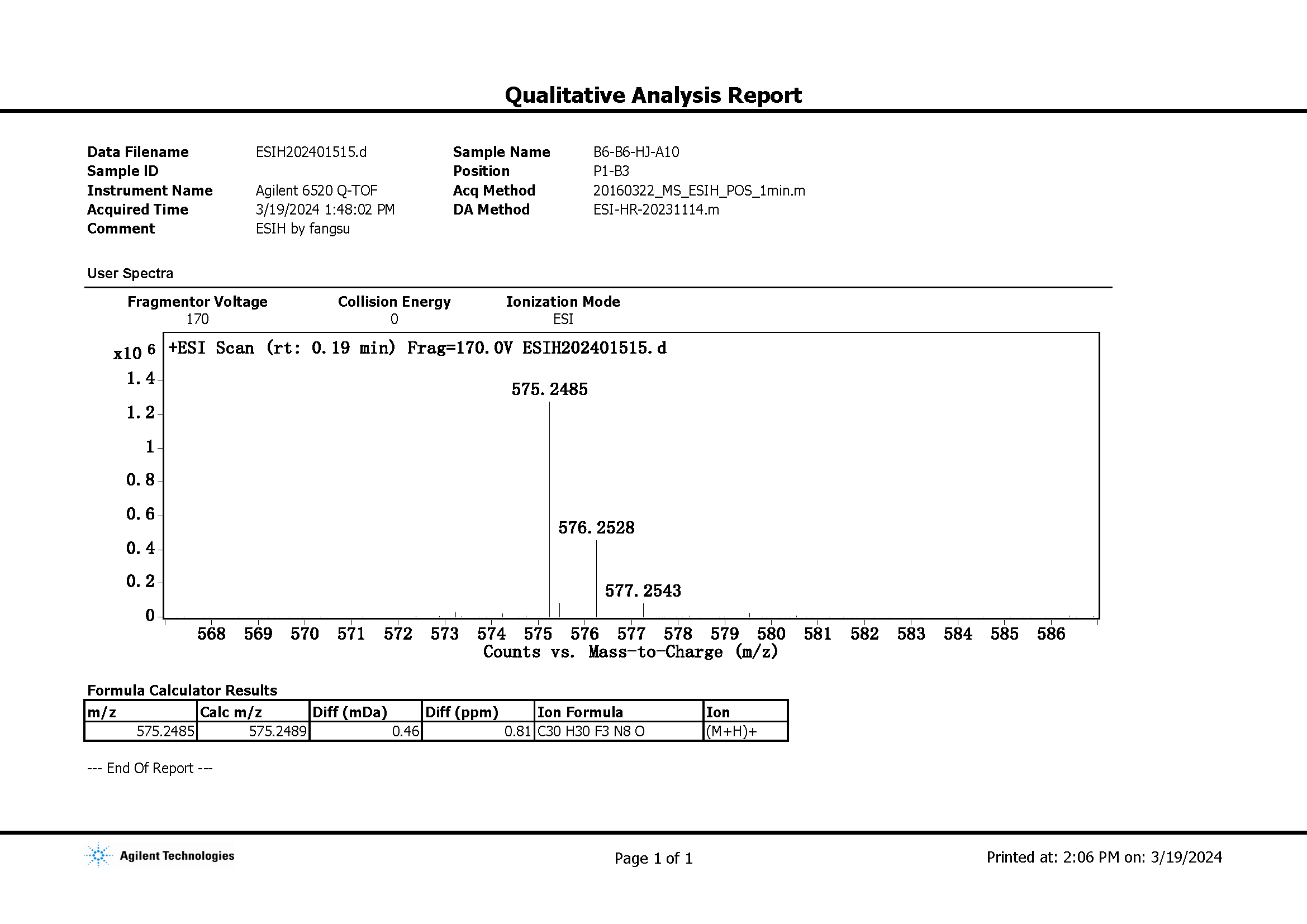


HRMS (ESI) of compound **13k**


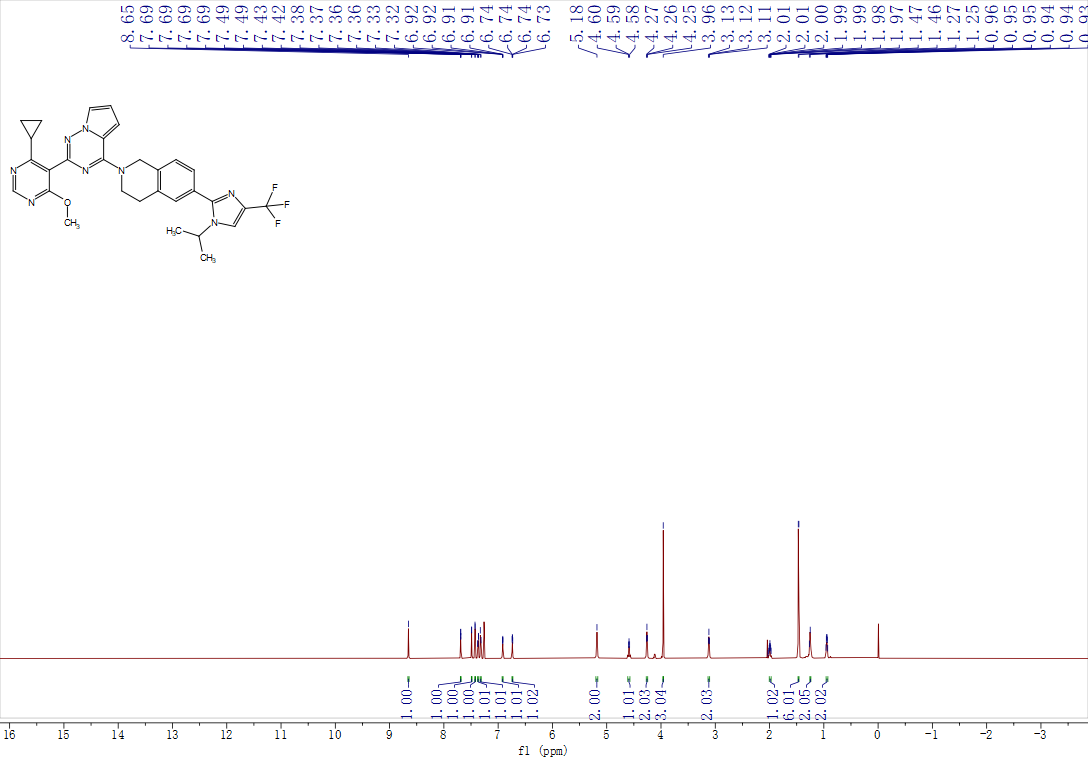


1H NMR of compound **13l**


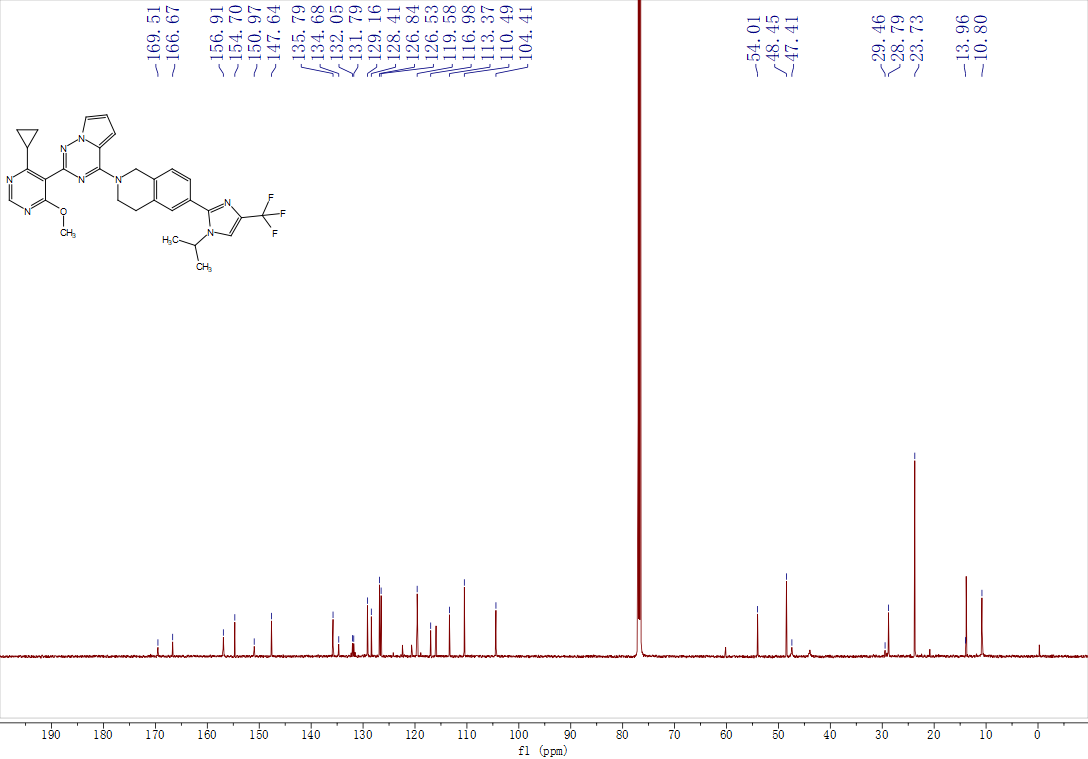


13C NMR of compound **13l**

**
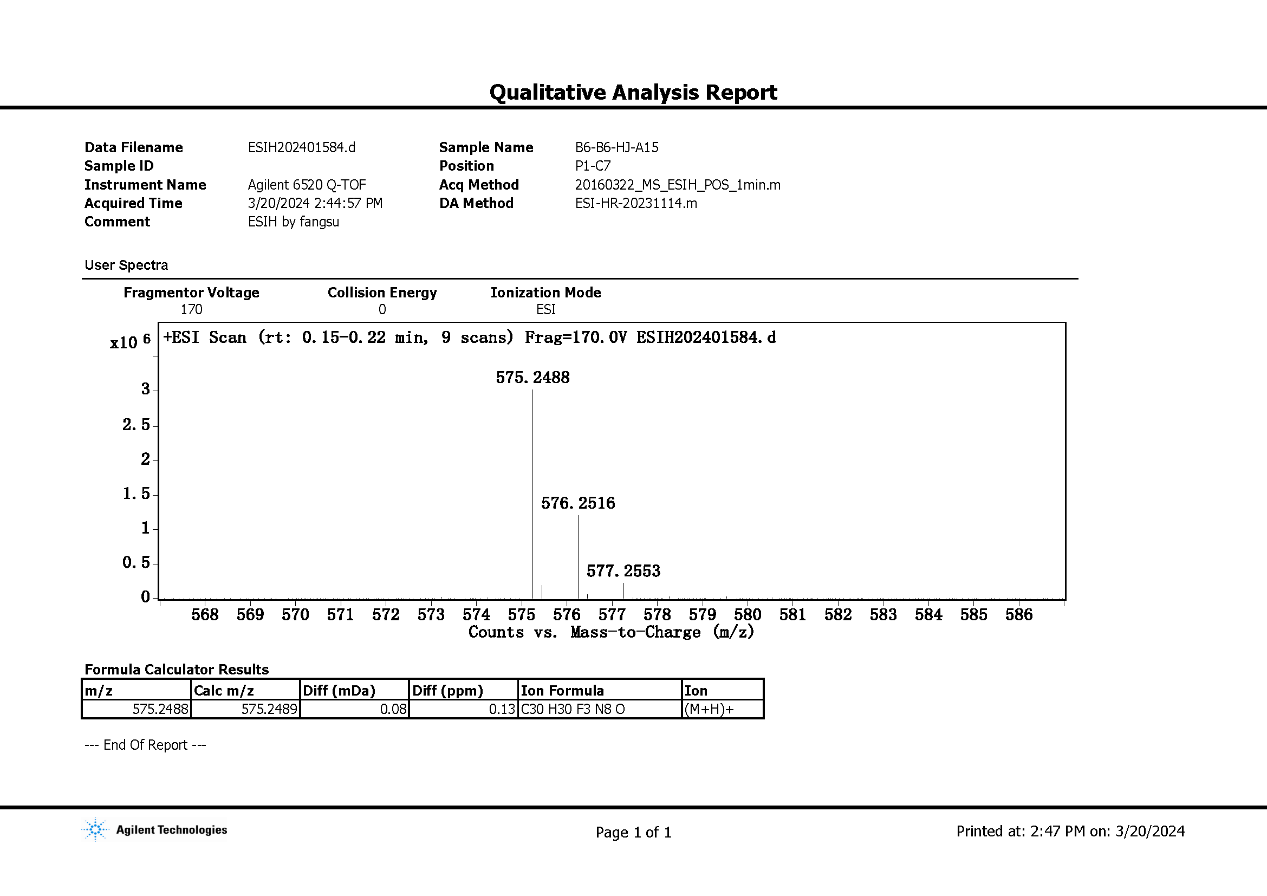
**

HRMS (ESI) of compound **13l**


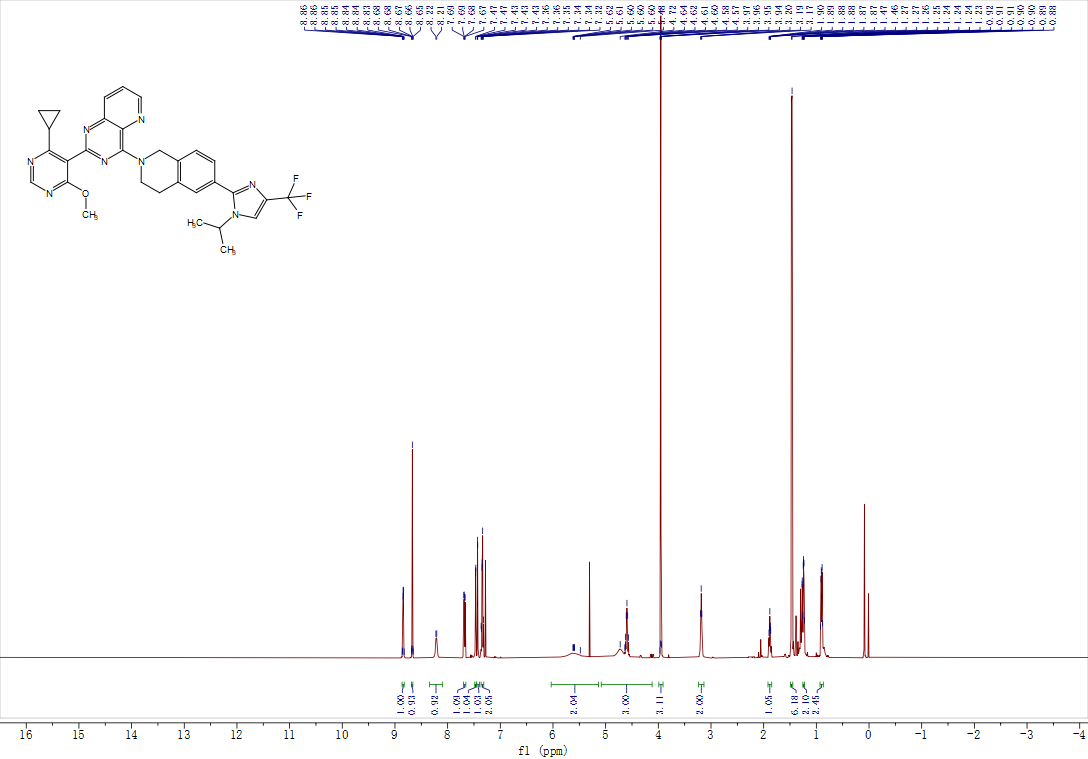


1H NMR of compound **13m**


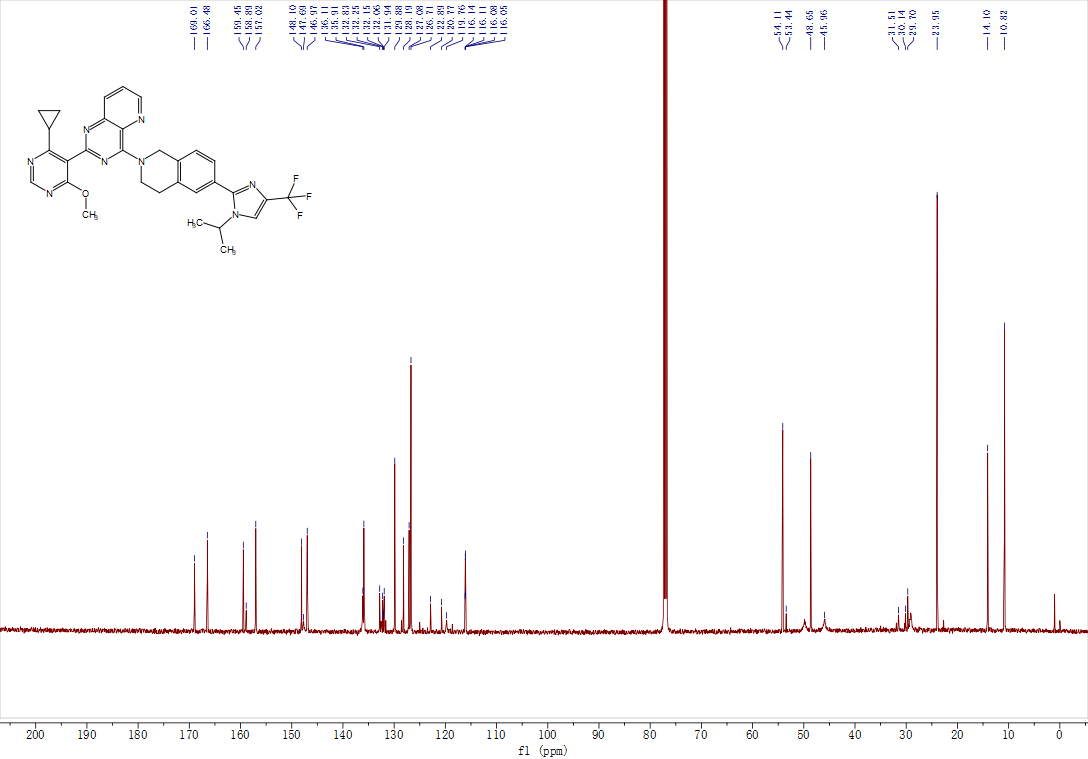


13C NMR of compound **13m**

**
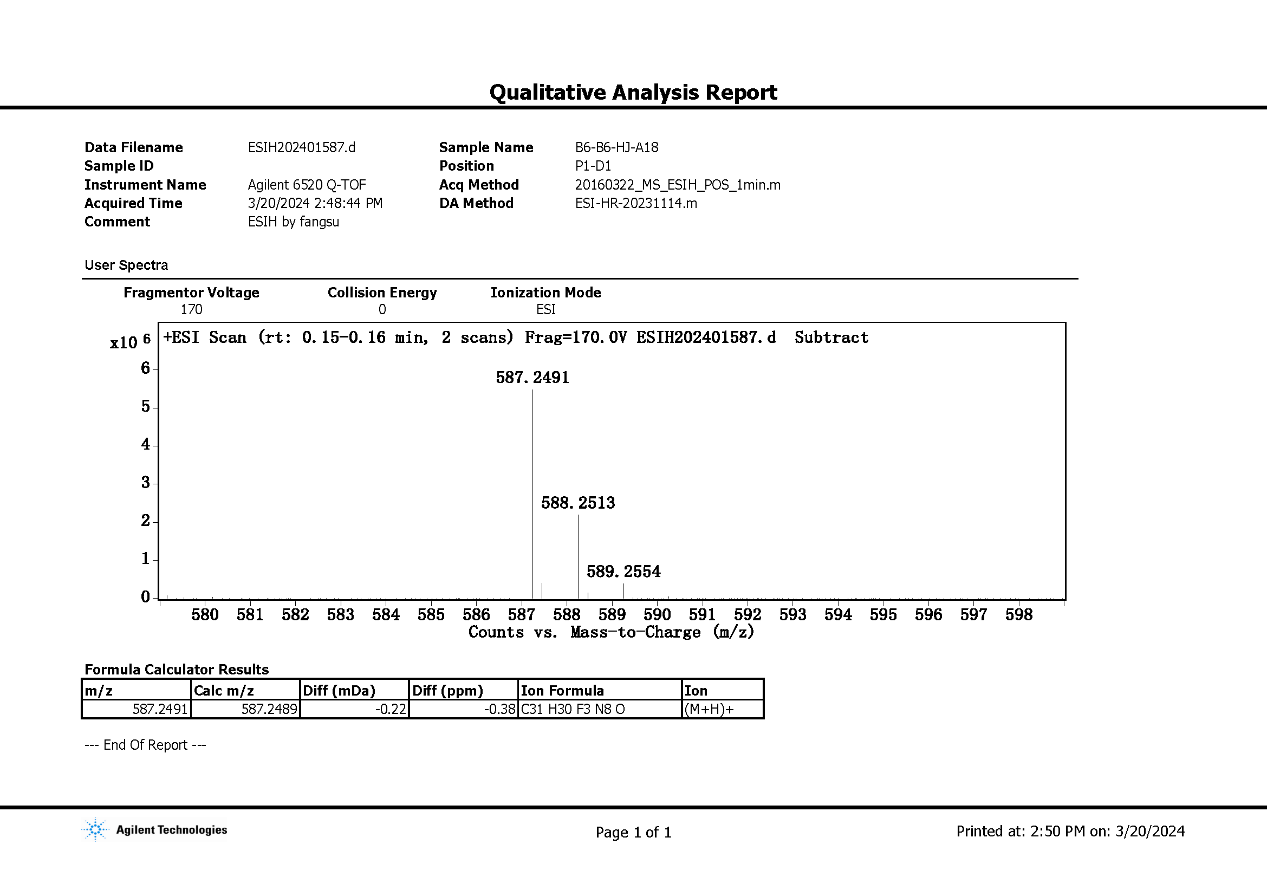
**

HRMS (ESI) of compound **13m**


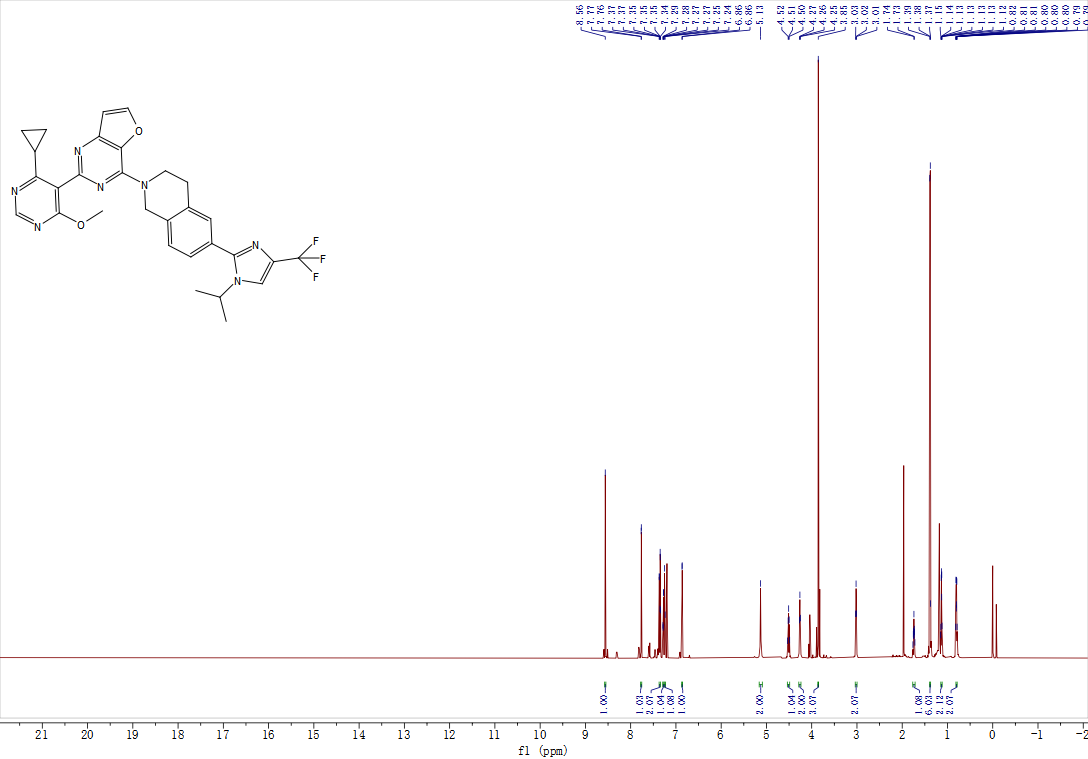


1H NMR of compound **13n**


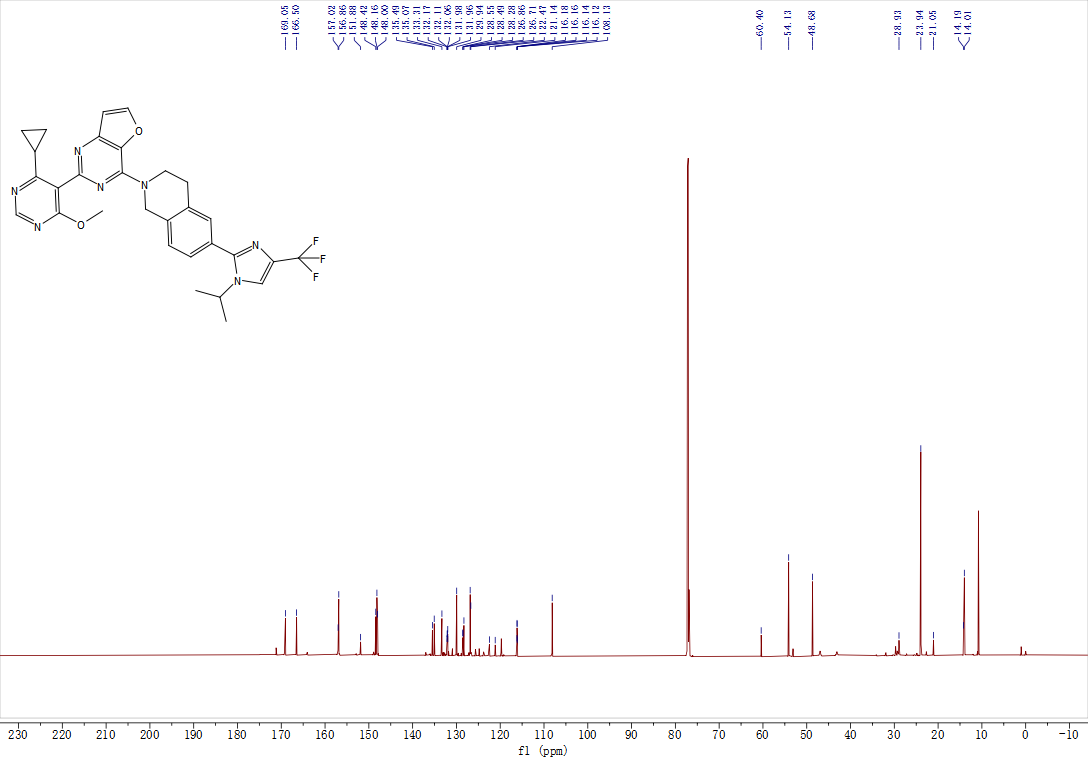


13C NMR of compound **13n**

**
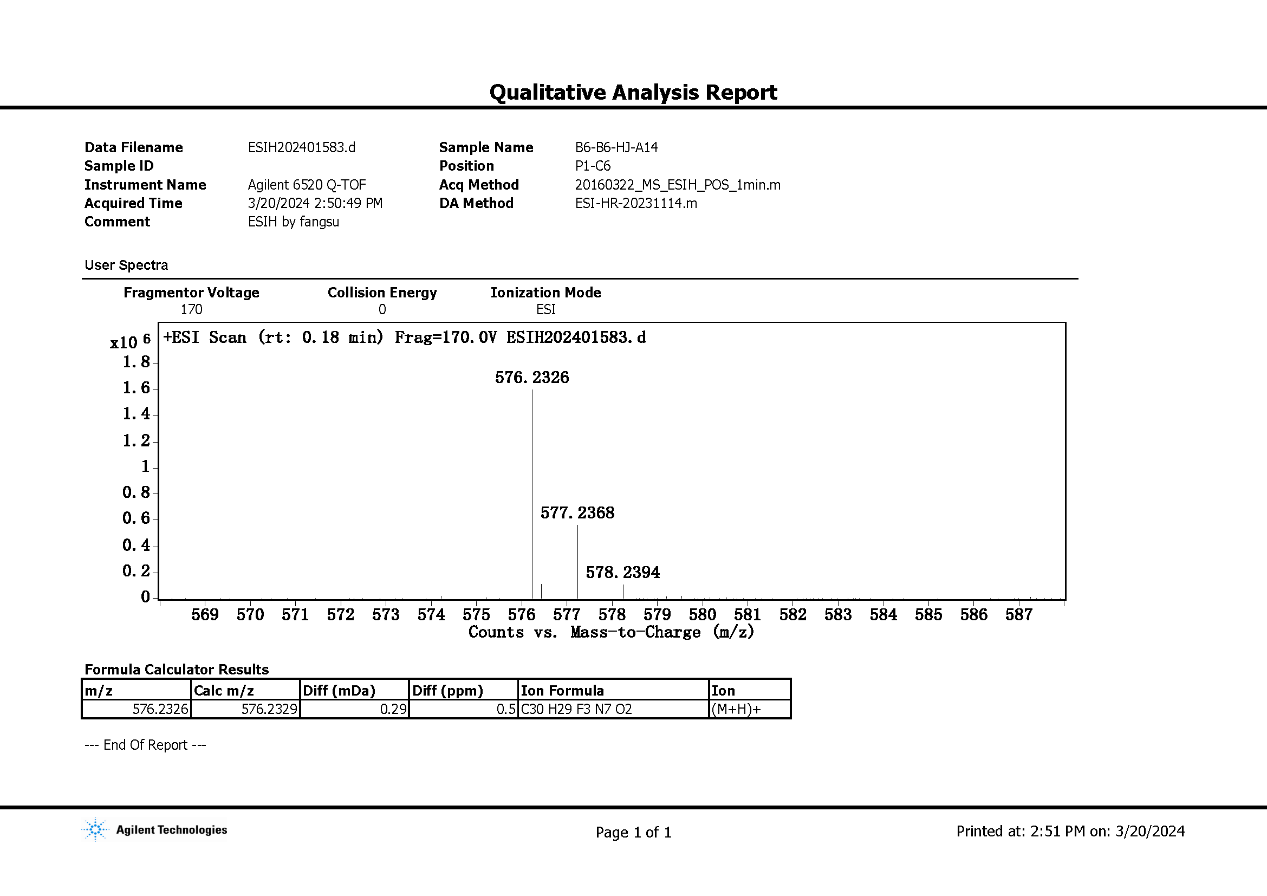
**

HRMS (ESI) of compound **13n**


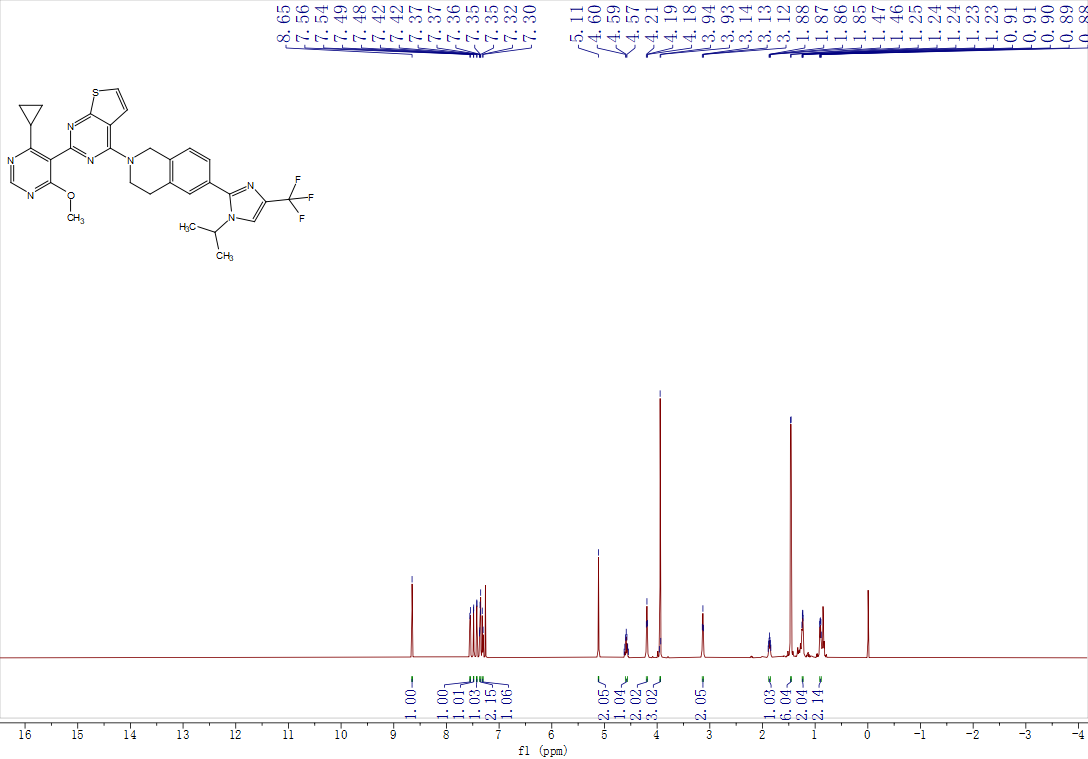


1H NMR of compound **13o**


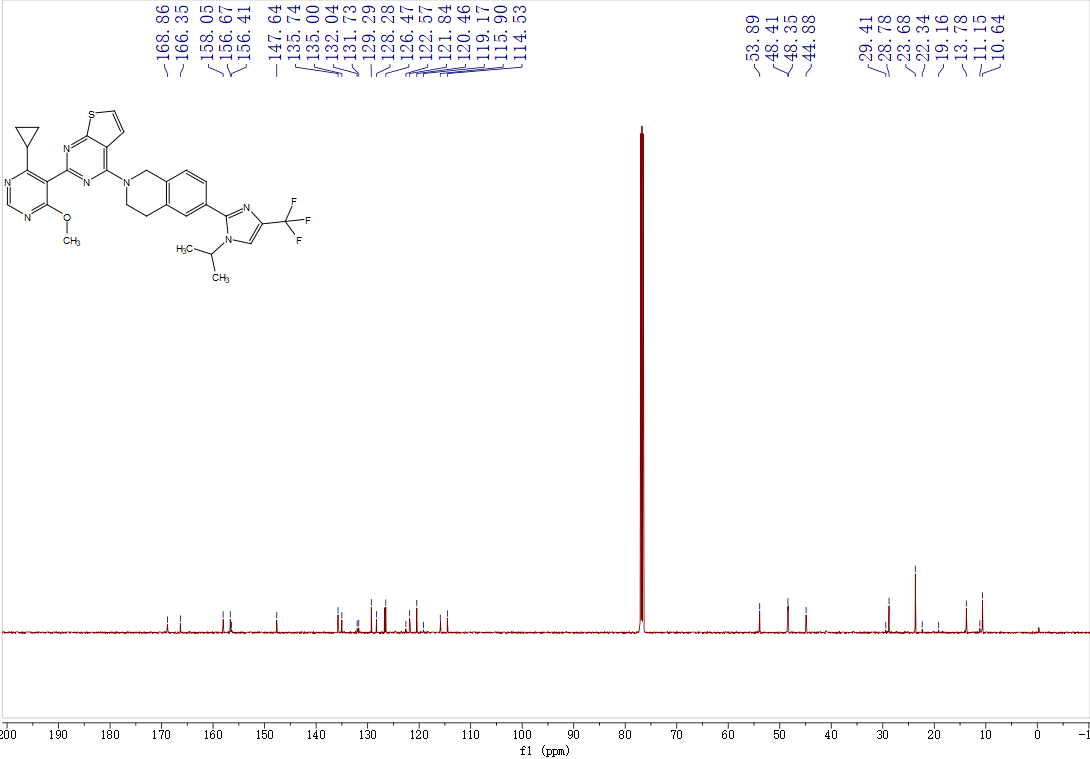


13C NMR of compound **13o**


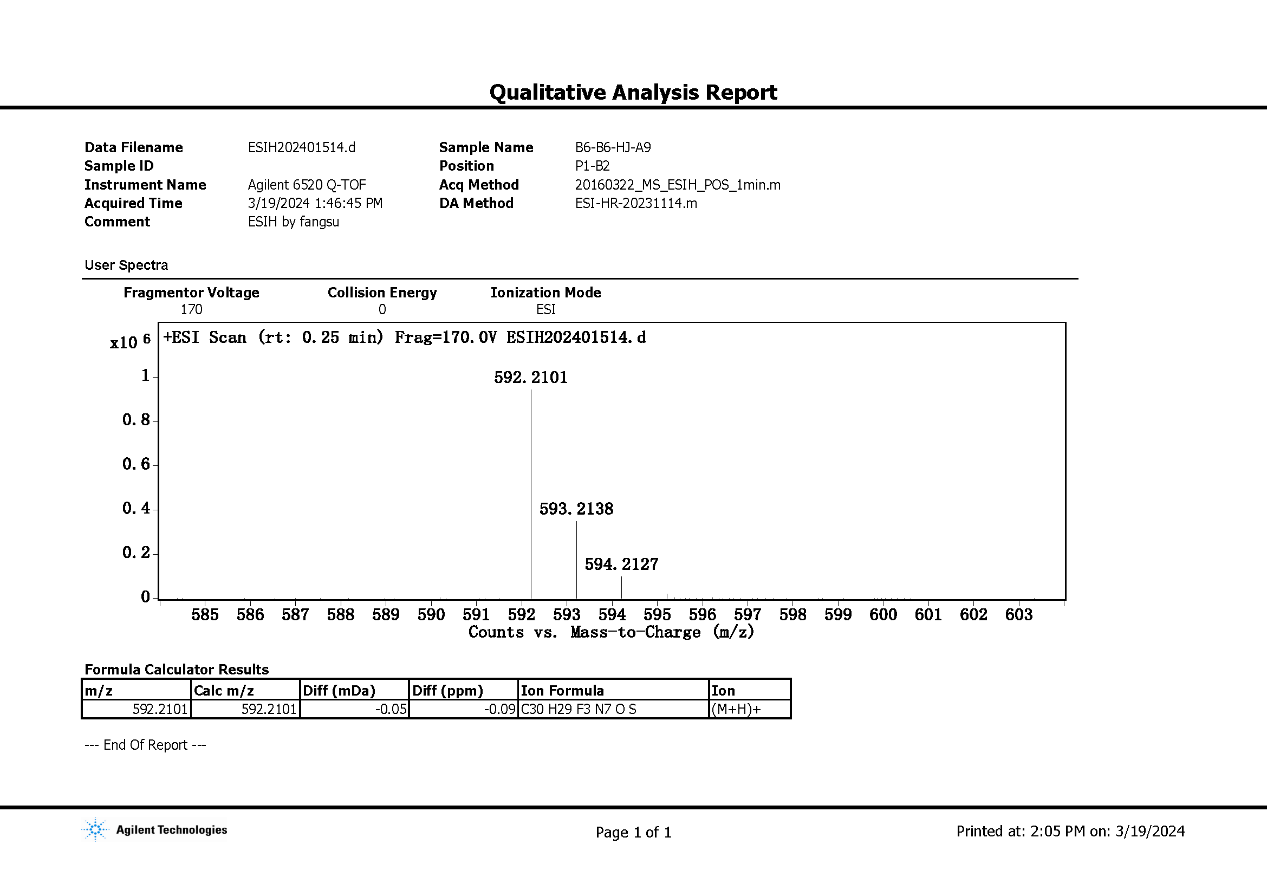


HRMS (ESI) of compound **13o**


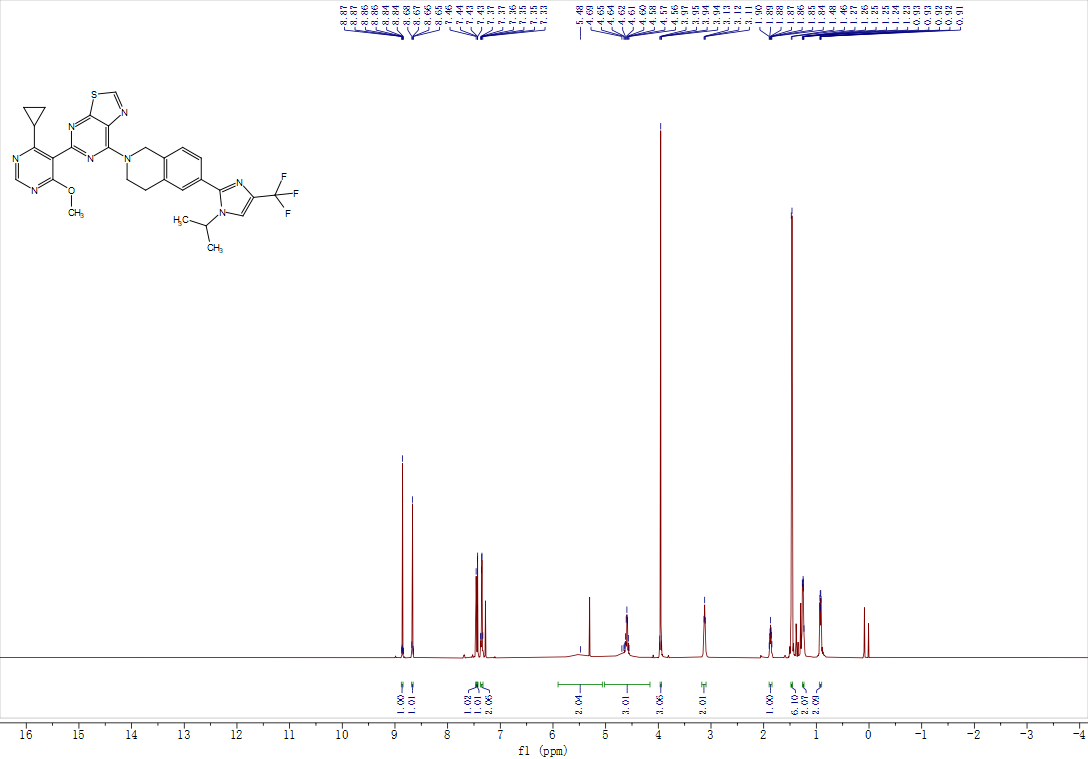


1H NMR of compound **13p**


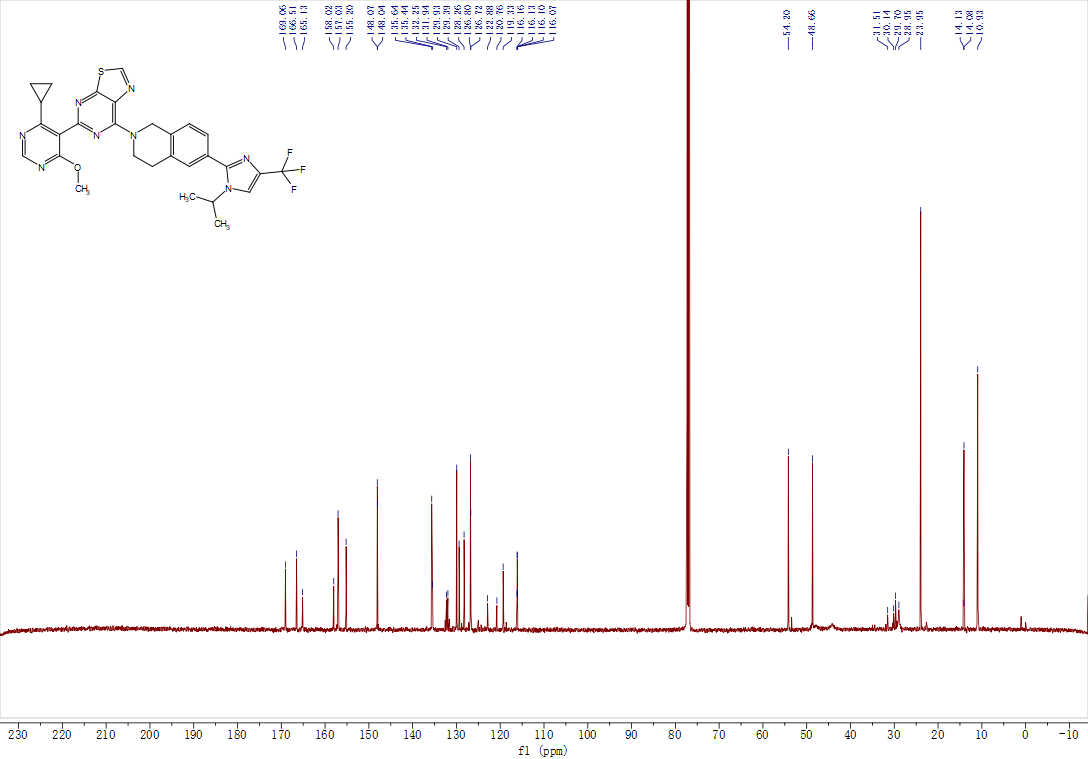


13C NMR of compound **13p**


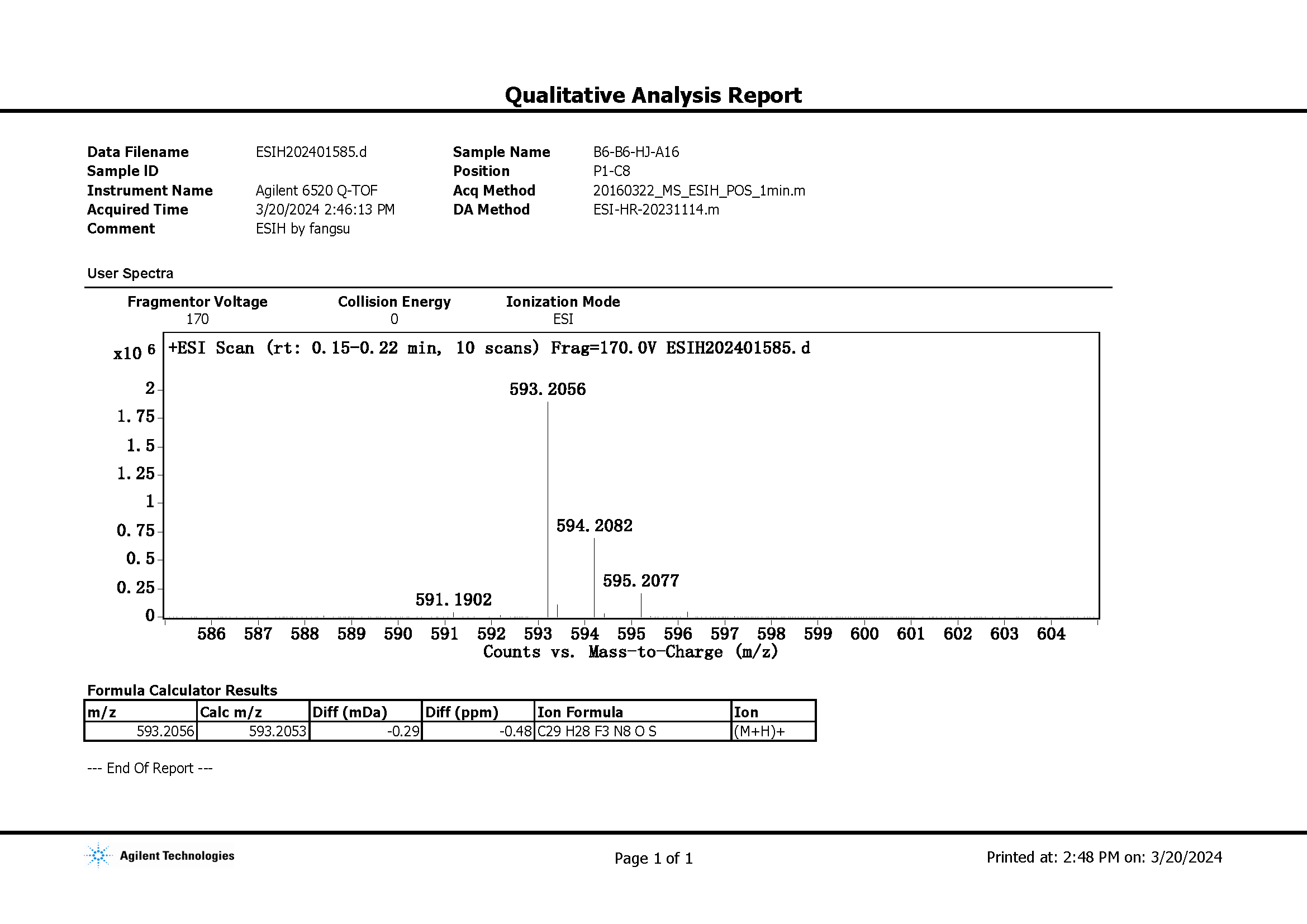


HRMS (ESI) of compound **13p**

**
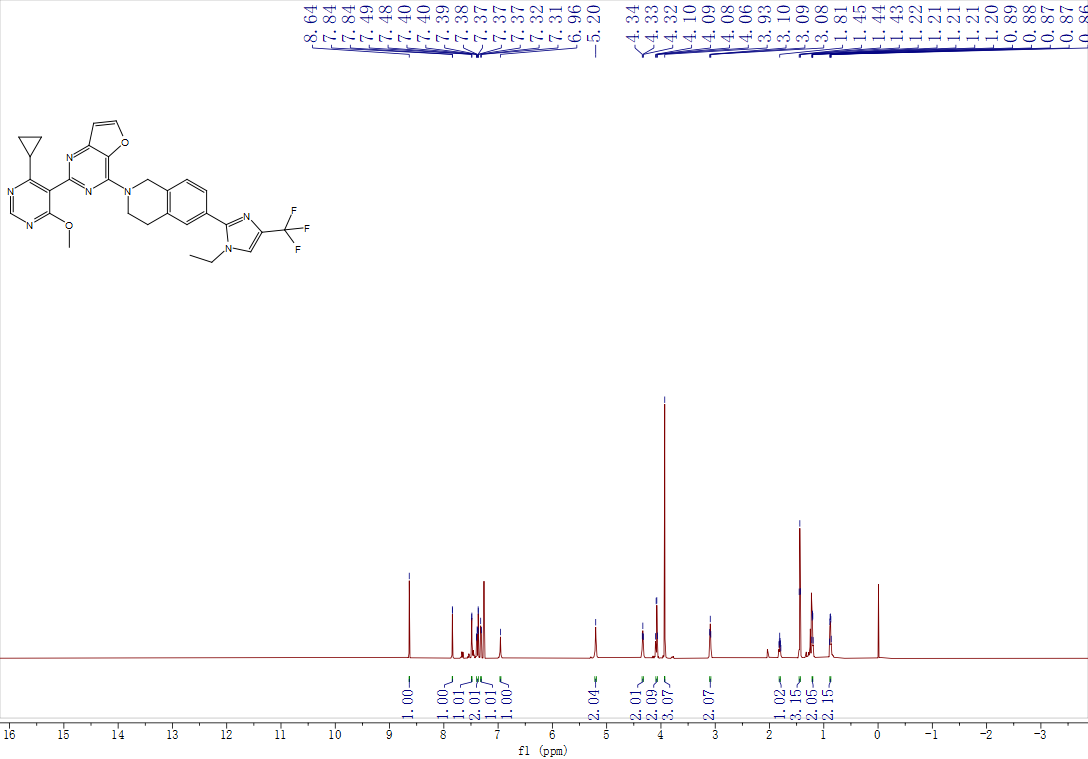
**

1H NMR of compound **14a
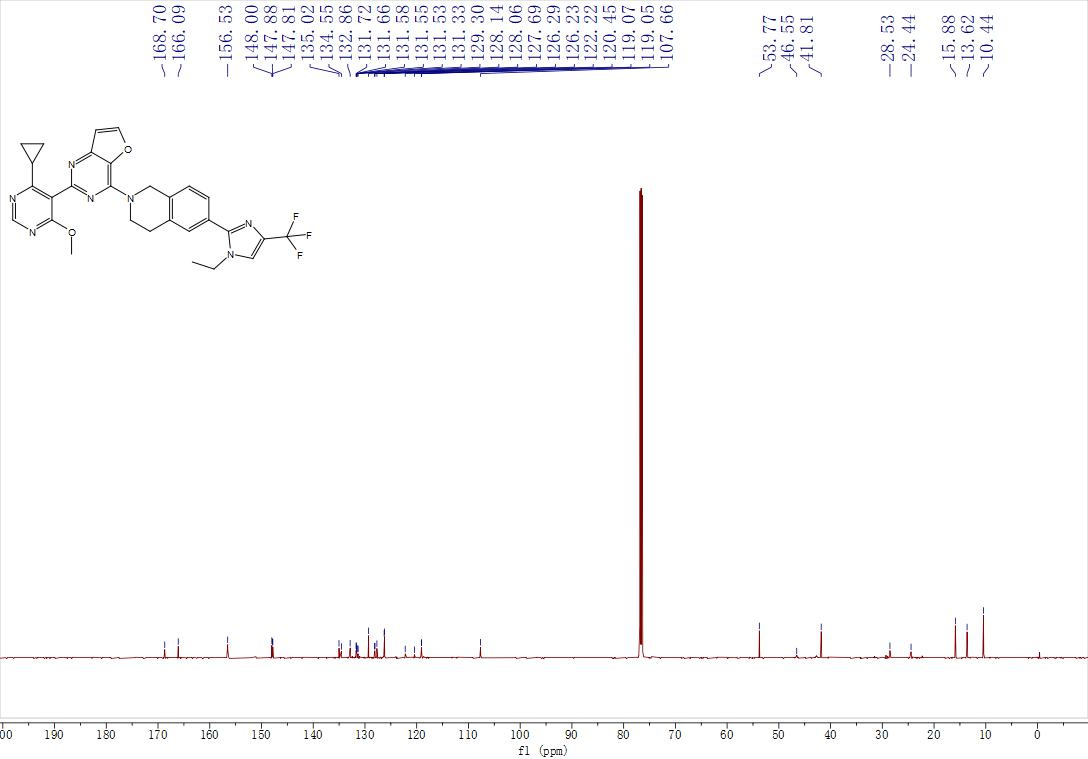
**

13C NMR of compound **14a**

**
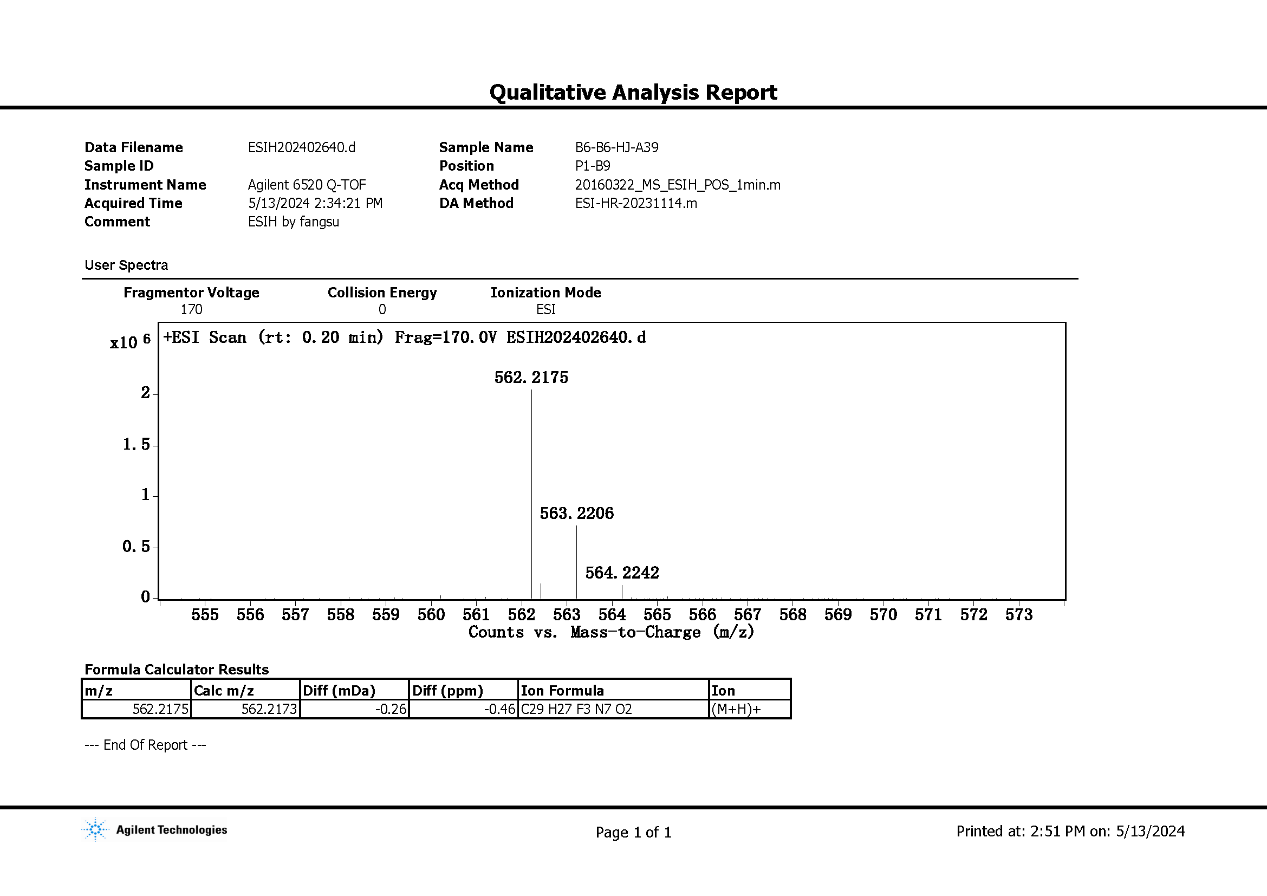
**

HRMS (ESI) of compound **14a**

**
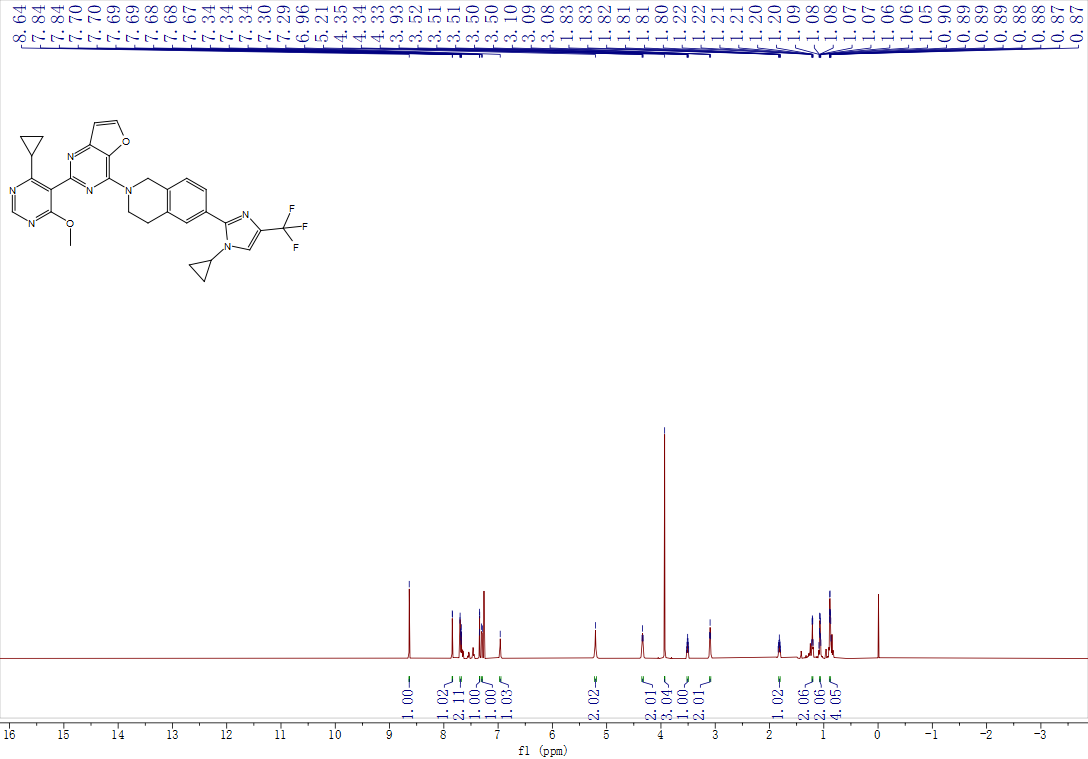
**

1H NMR of compound **14b
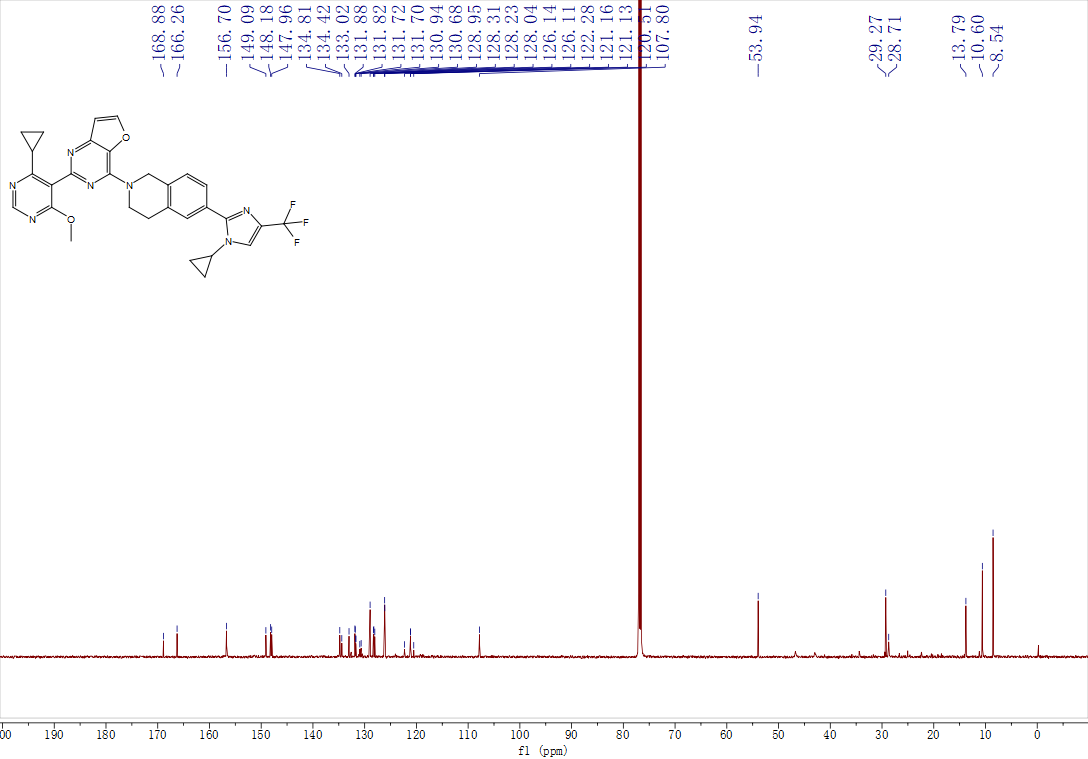
**

13C NMR of compound **14b**

**
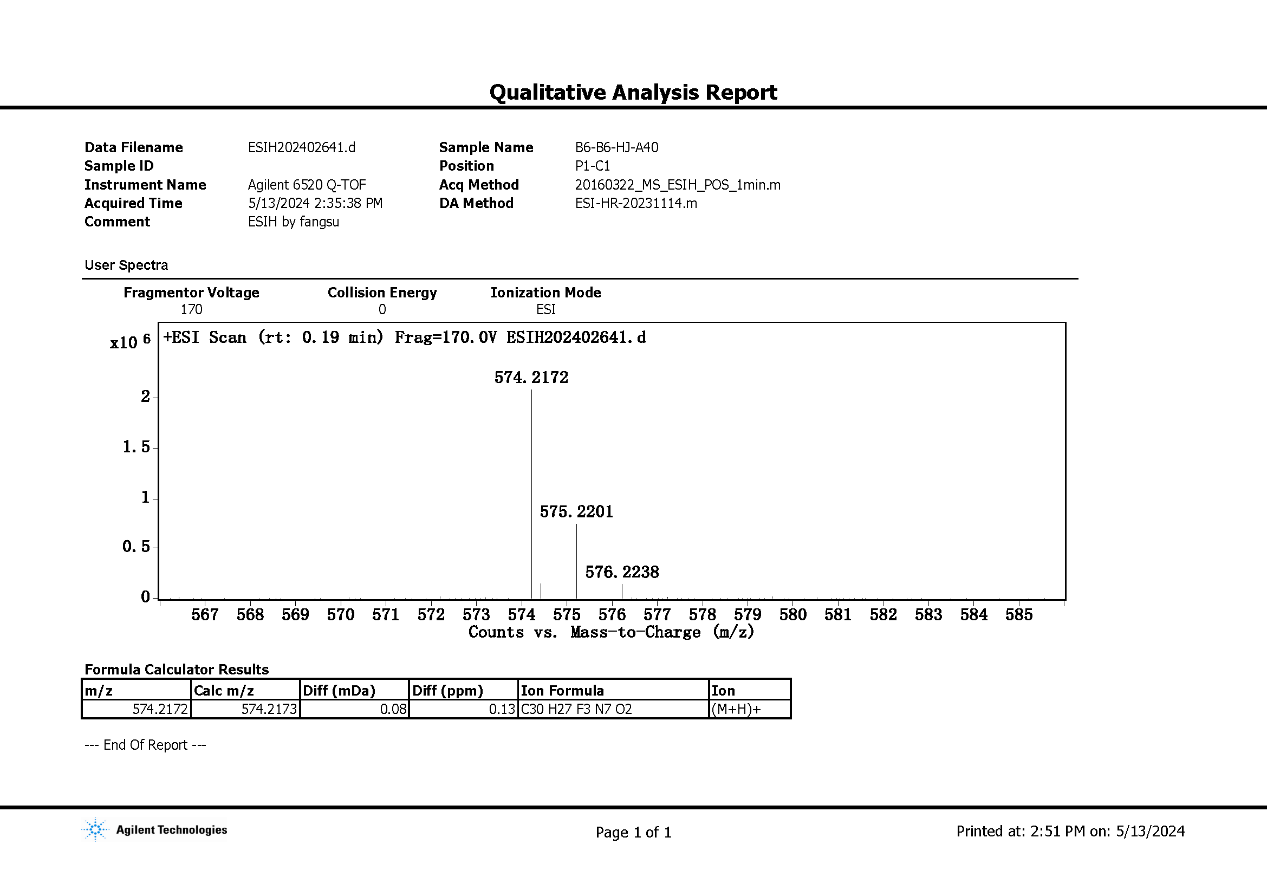
**

HRMS (ESI) of compound **14b**

**
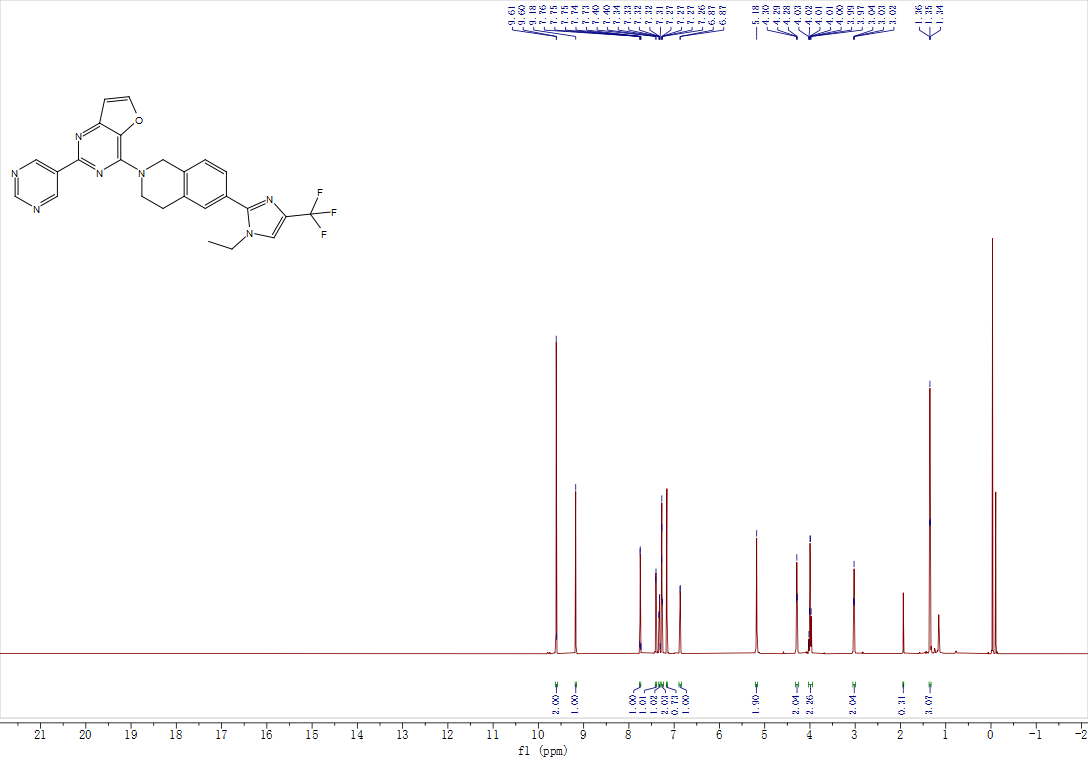
**

1H NMR of compound **14c
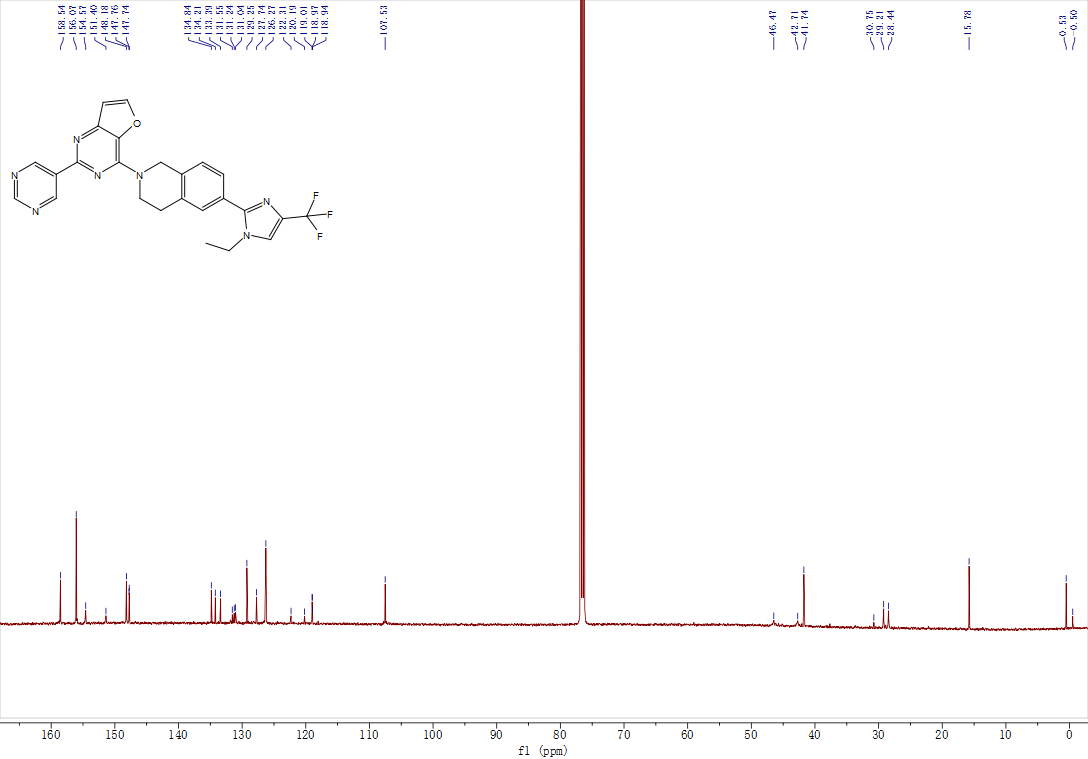
**

13C NMR of compound **14c**

**
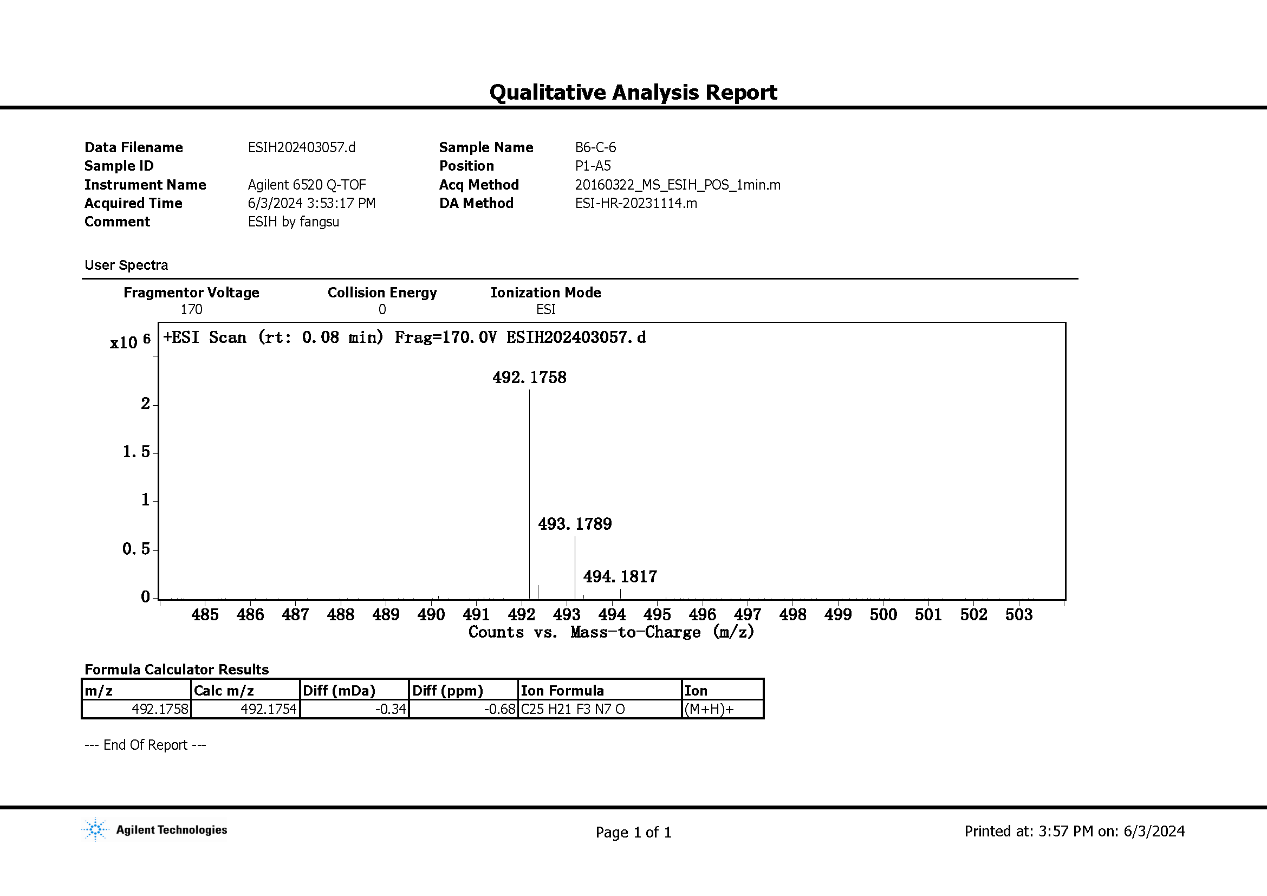
**

HRMS (ESI) of compound **14c**

**
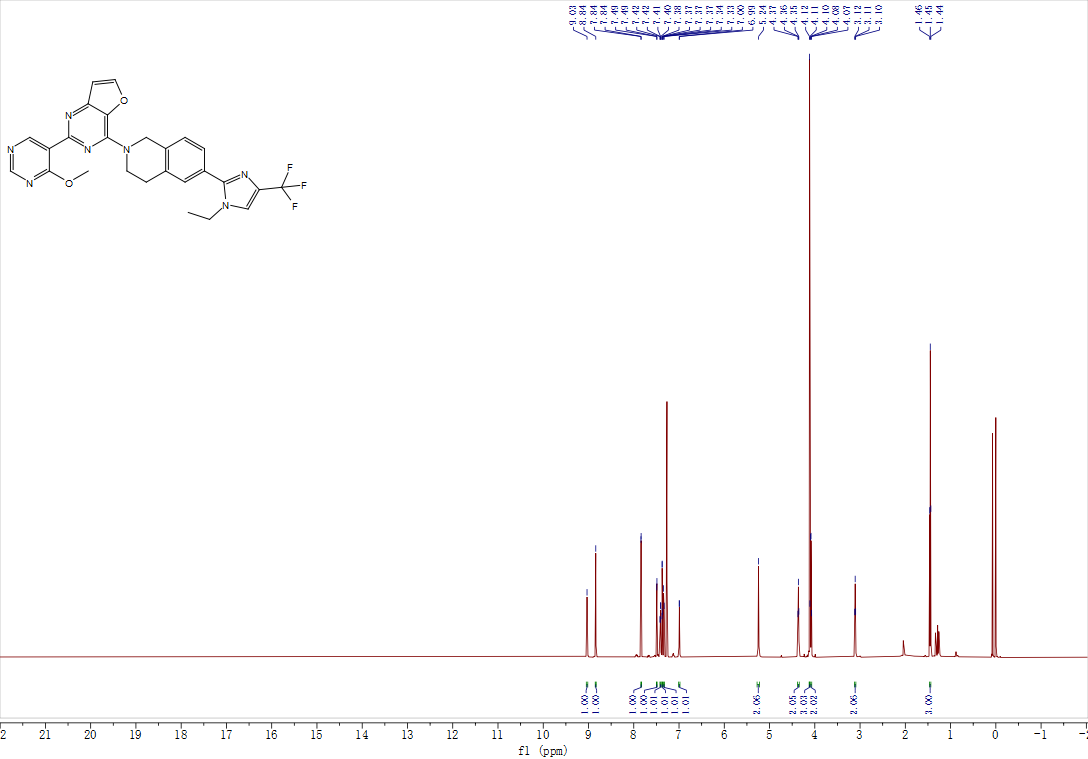
**

1H NMR of compound **14d
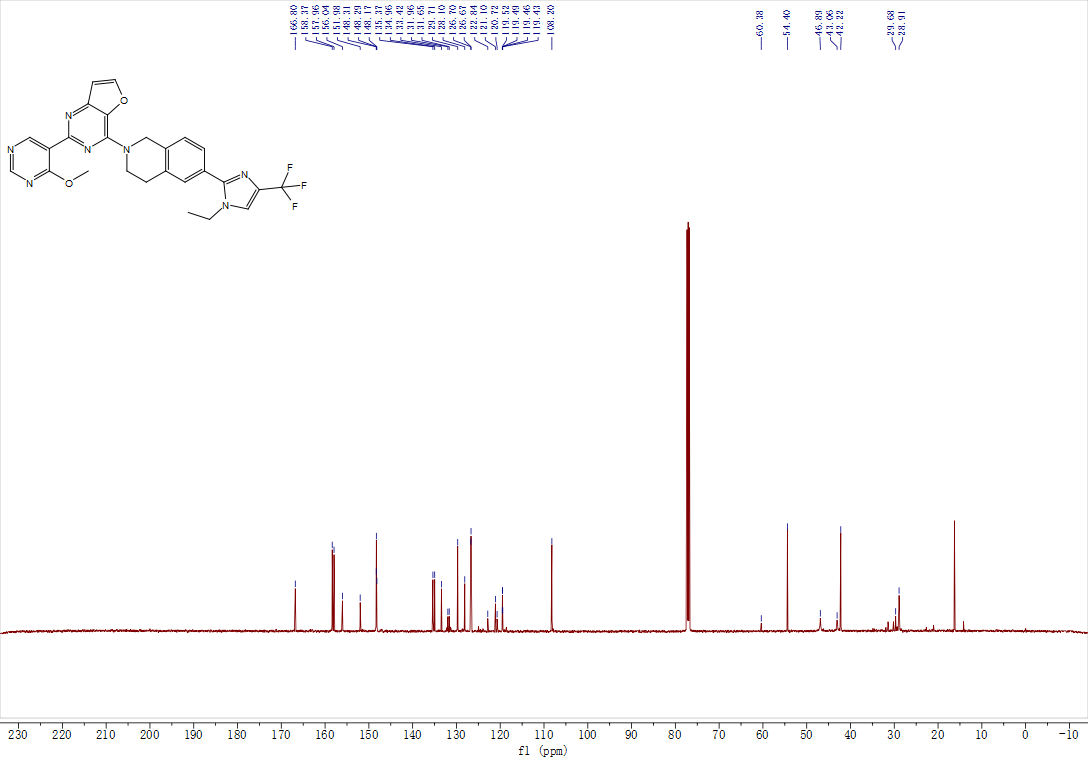
**

13C NMR of compound **14d**

**
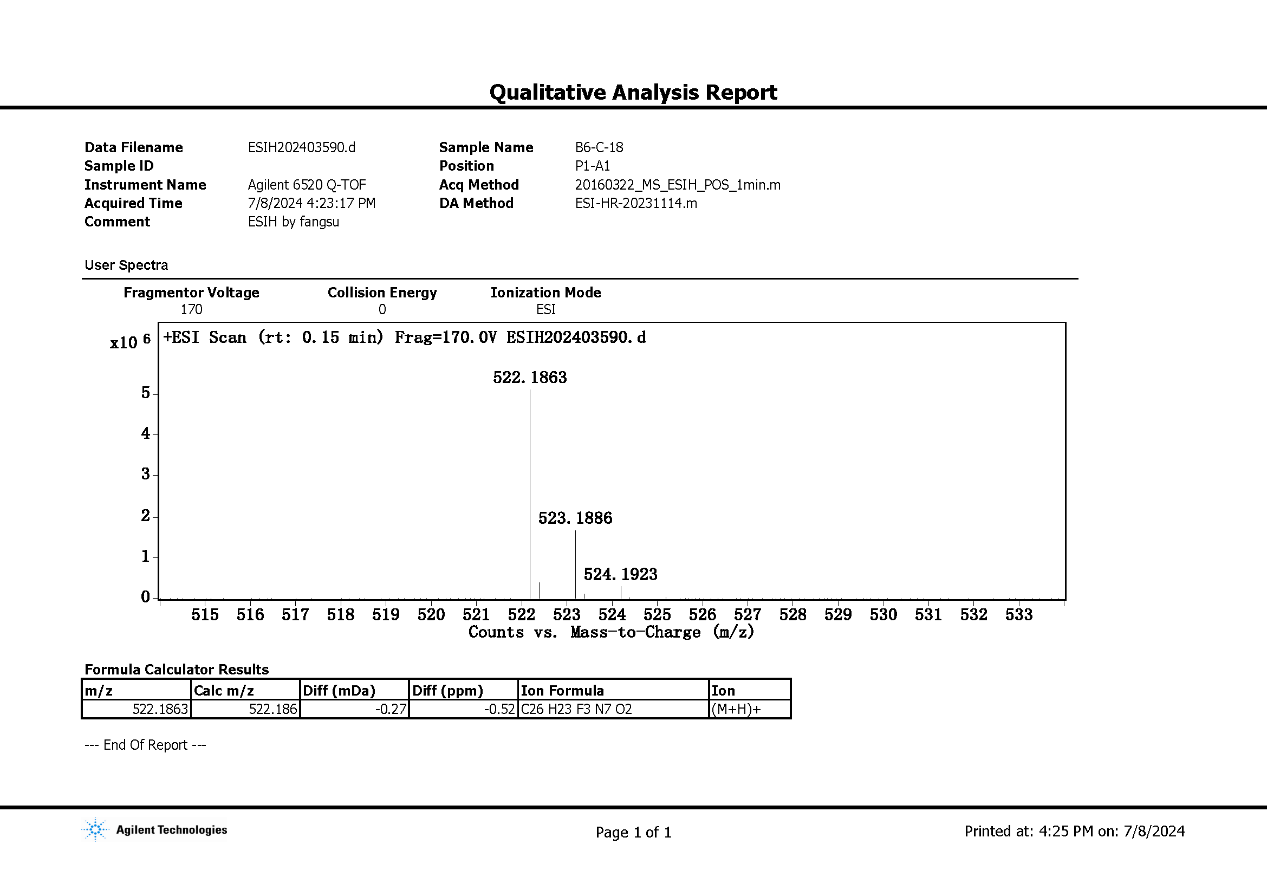
**

HRMS (ESI) of compound **14d**

**
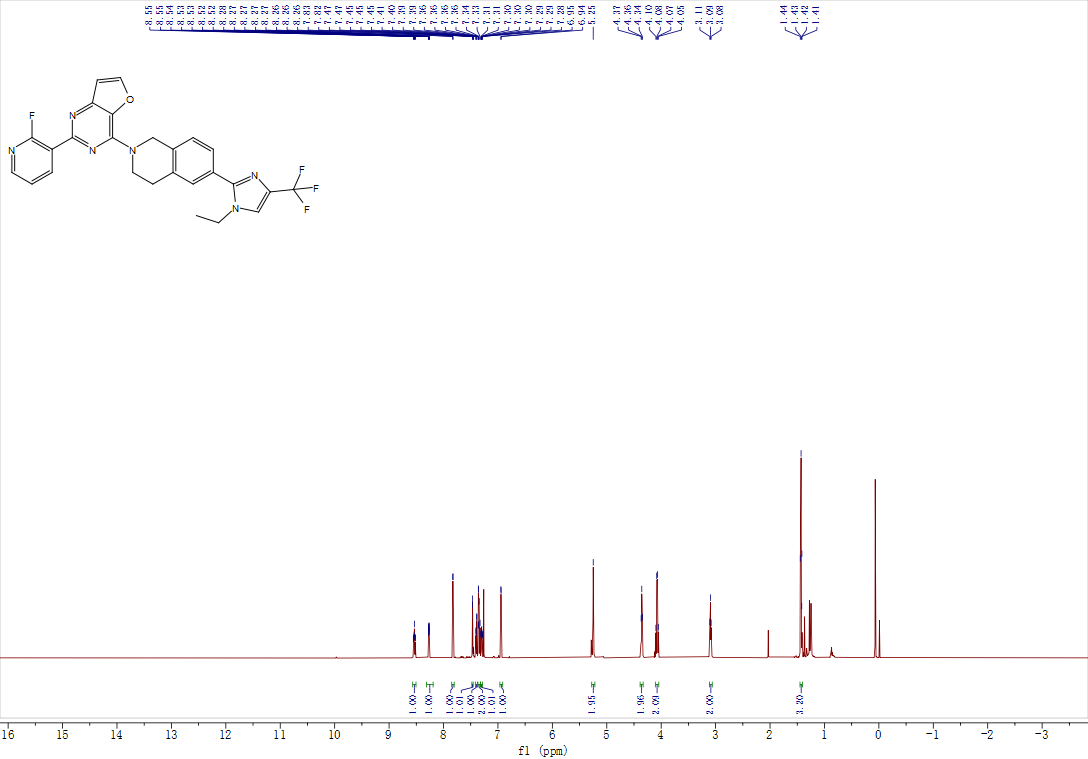
**

1H NMR of compound **14e**

**
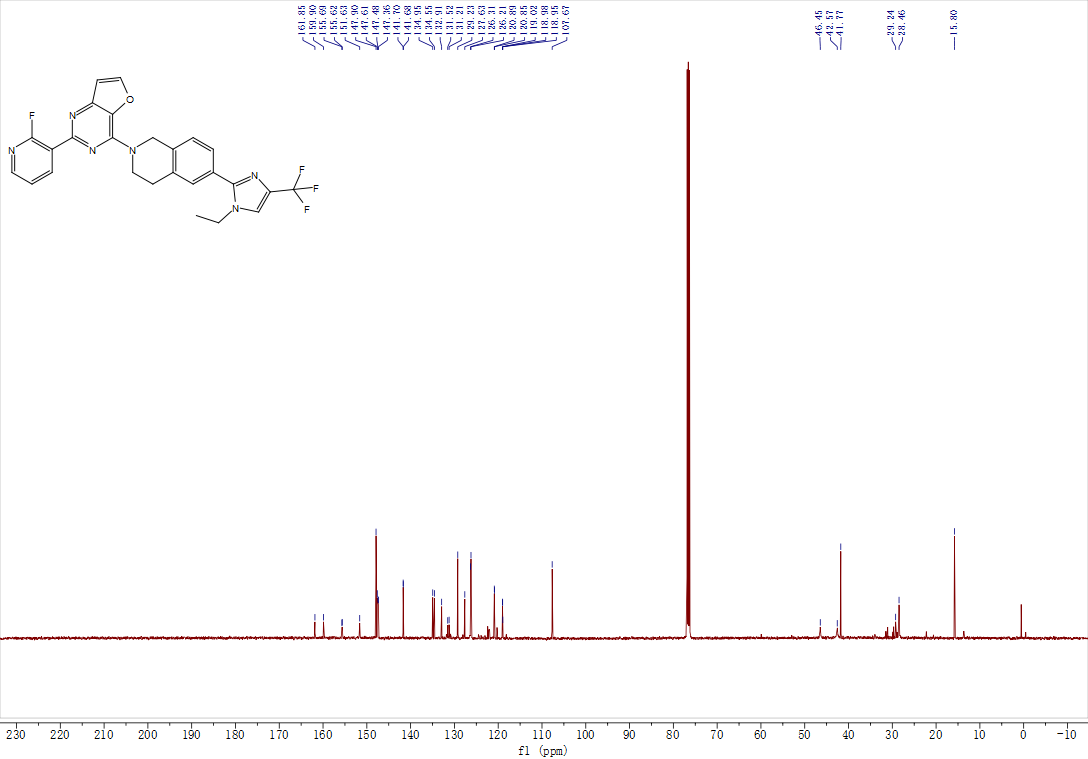
**

13C NMR of compound **14e**

**
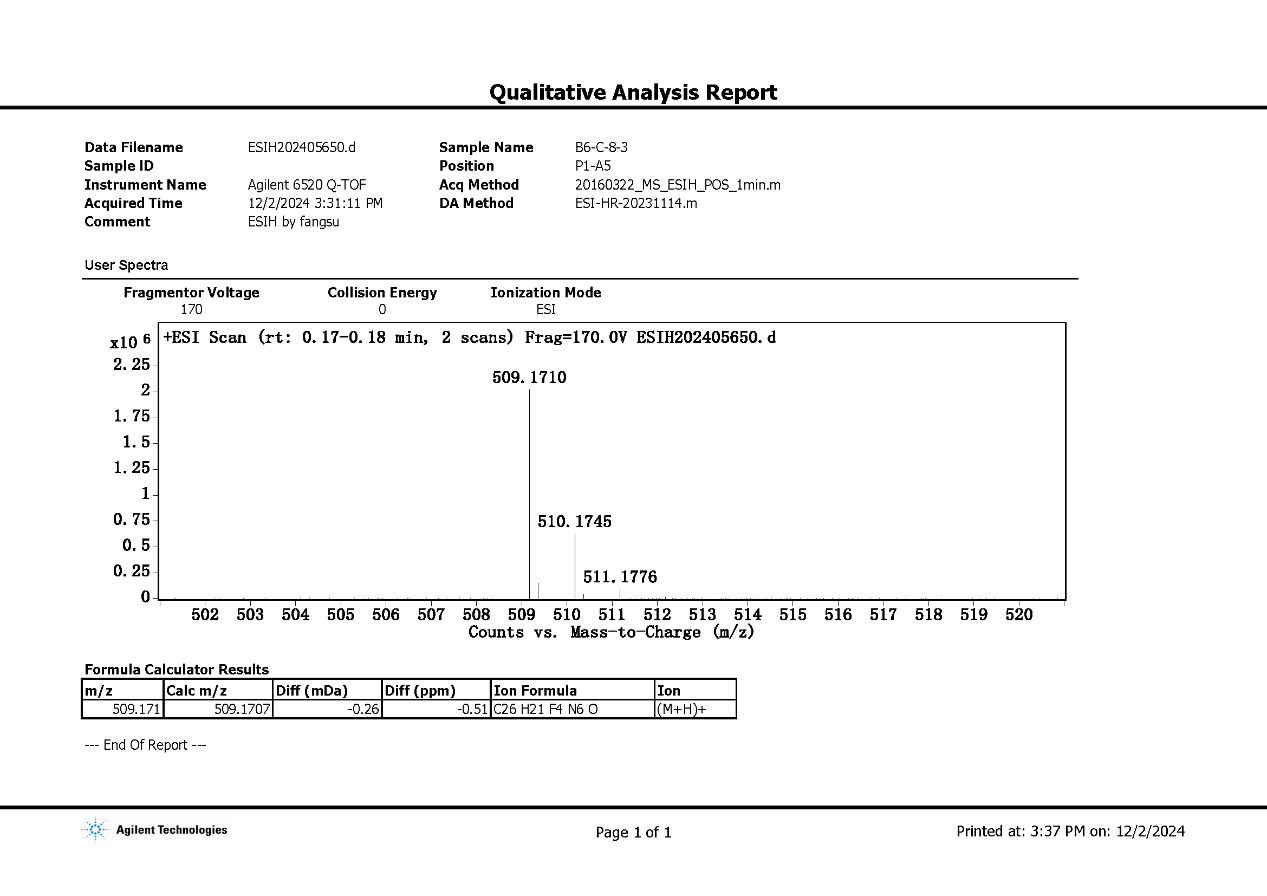
**

HRMS (ESI) of compound **14e**

**
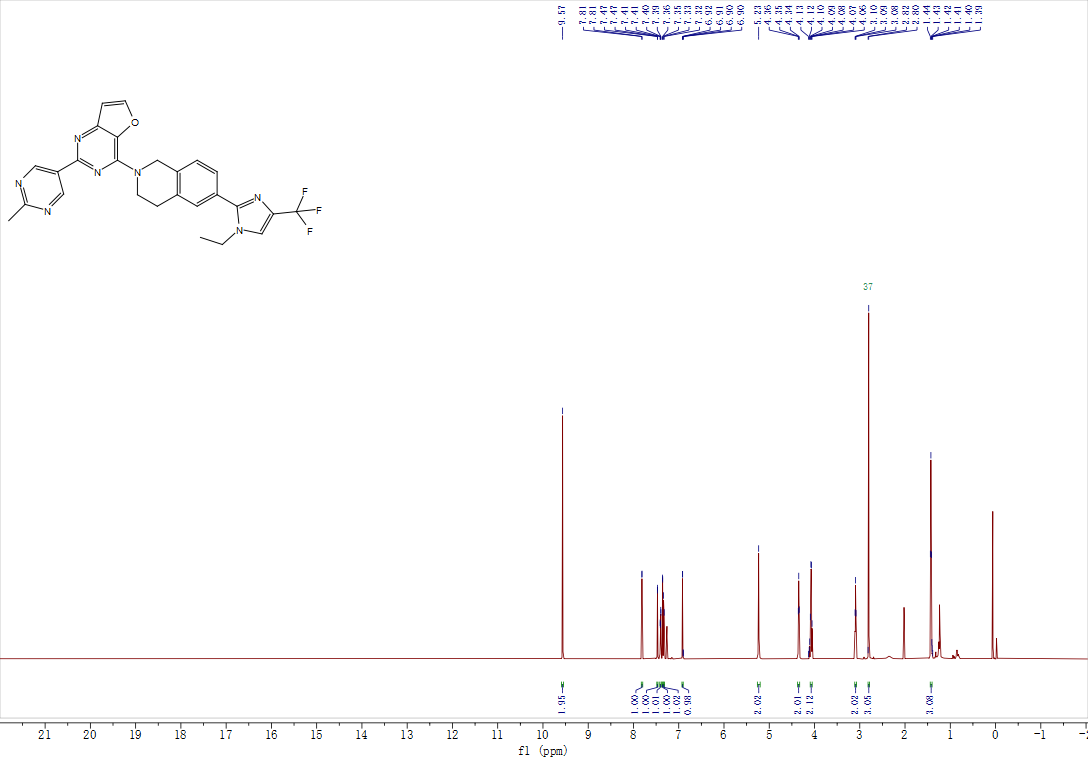
**

1H NMR of compound **14f**

**
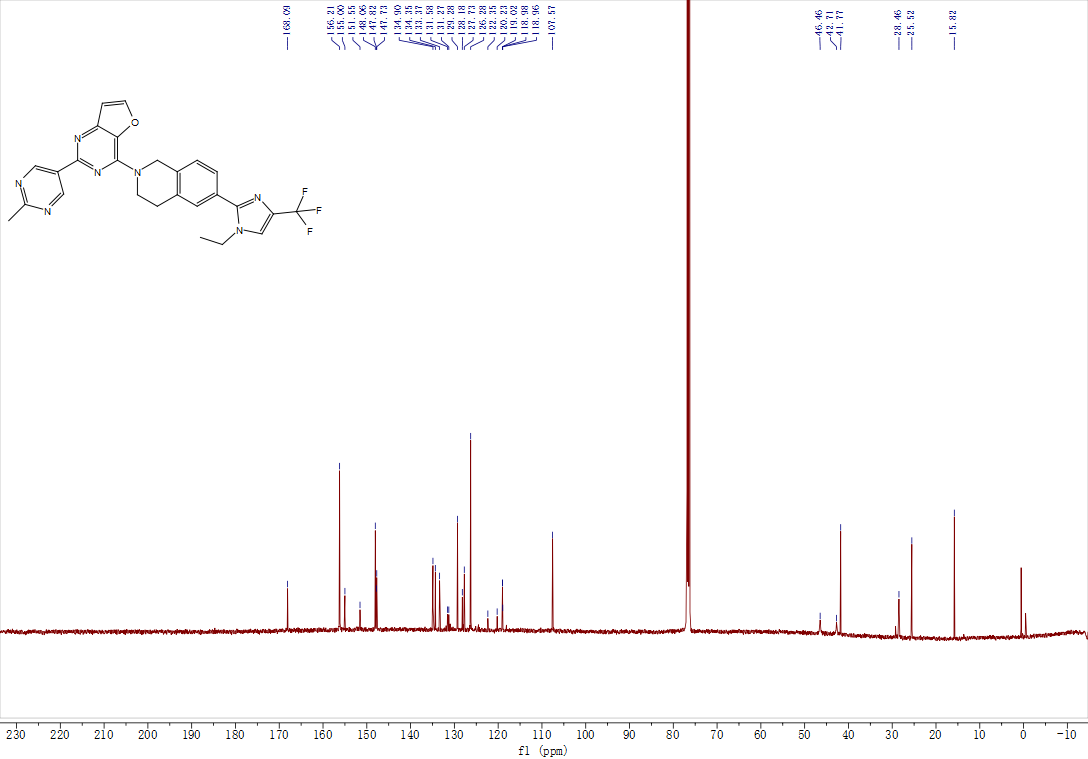
**

13C NMR of compound **14f**

**
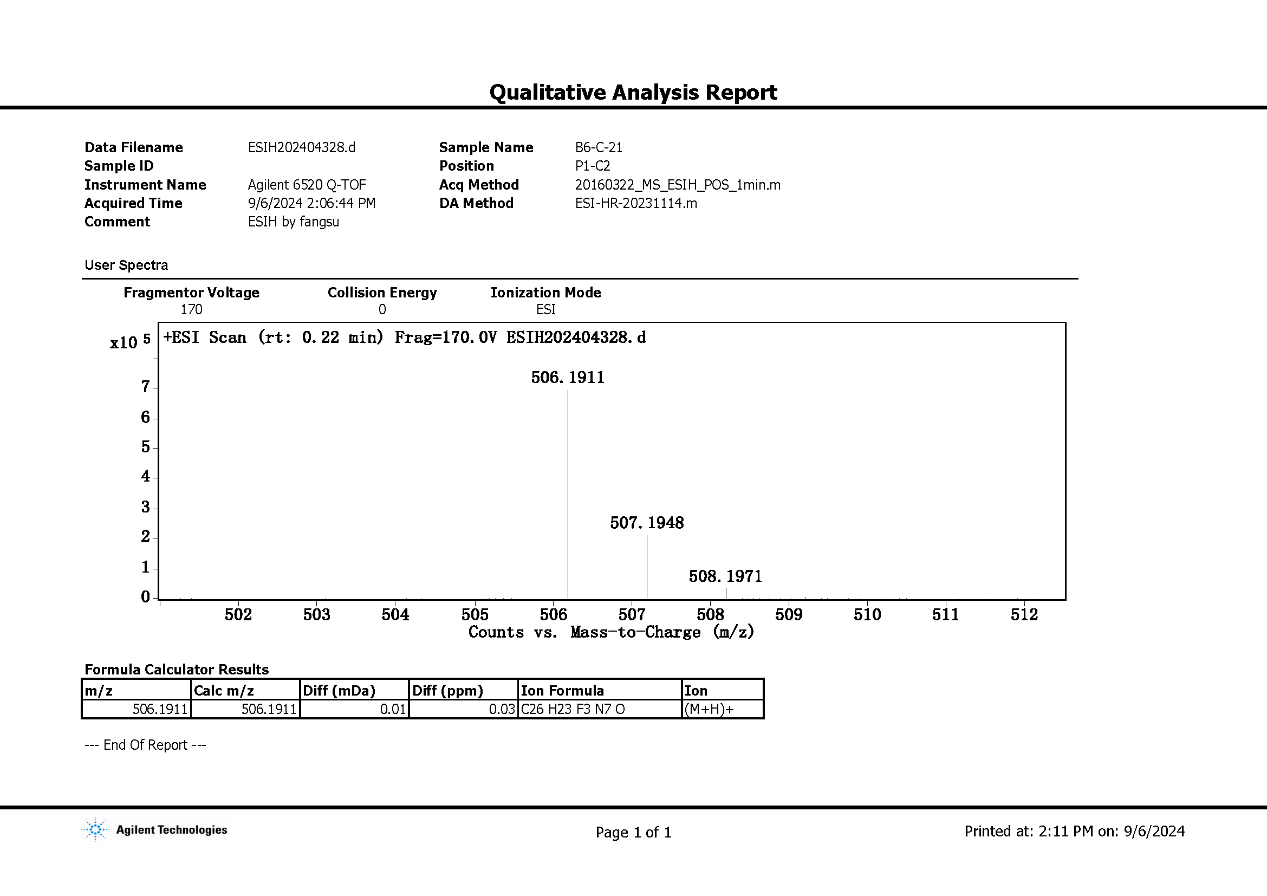
**

HRMS (ESI) of compound **14f**

**
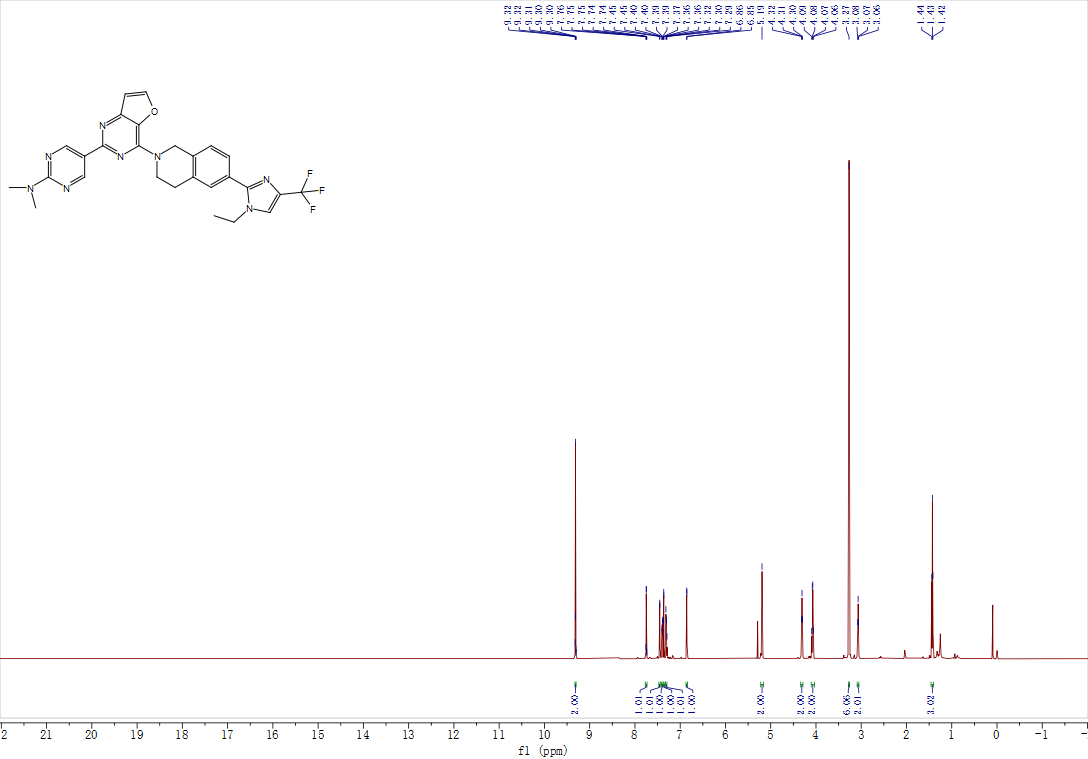
**

1H NMR of compound **14g**

**
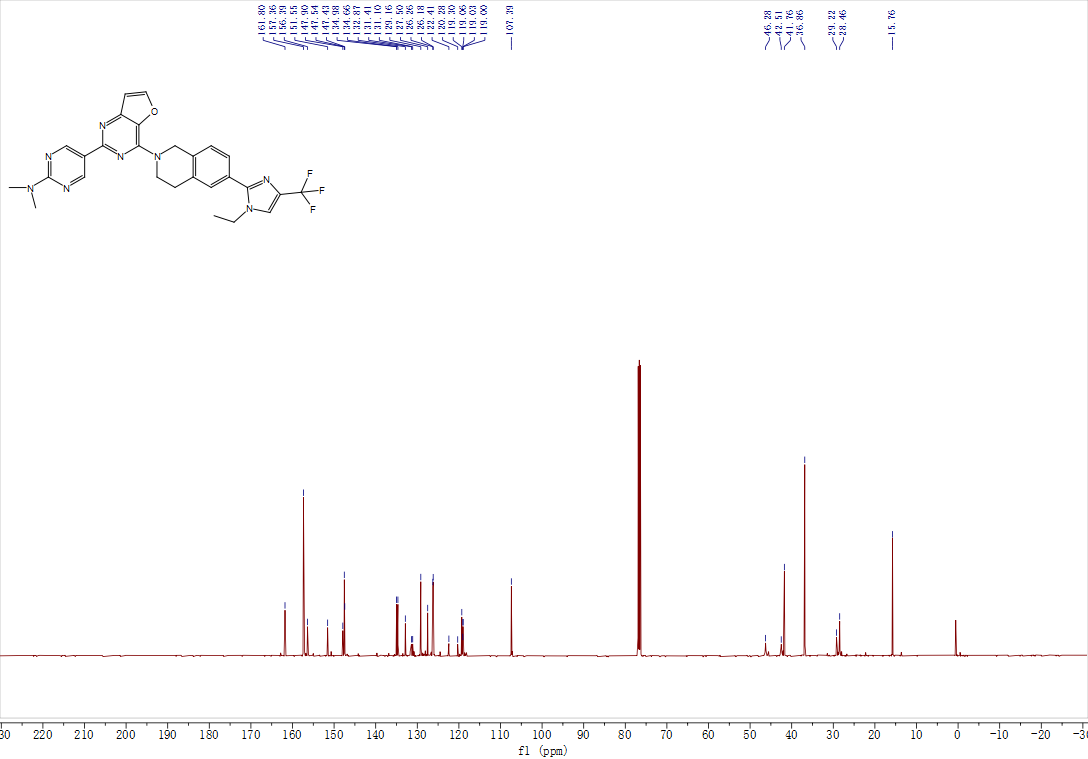
**

13C NMR of compound **14g**

**
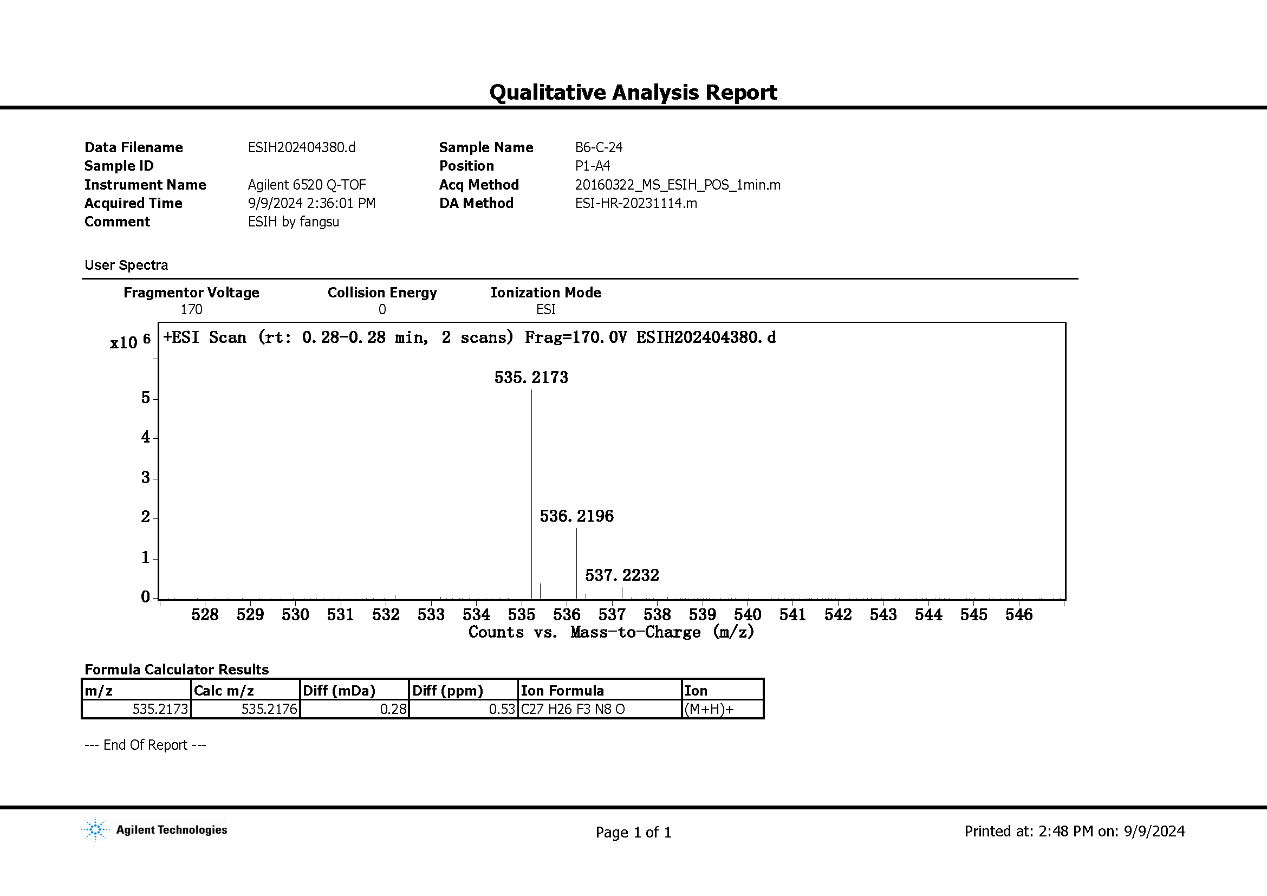
**

HRMS (ESI) of compound **14g**

**
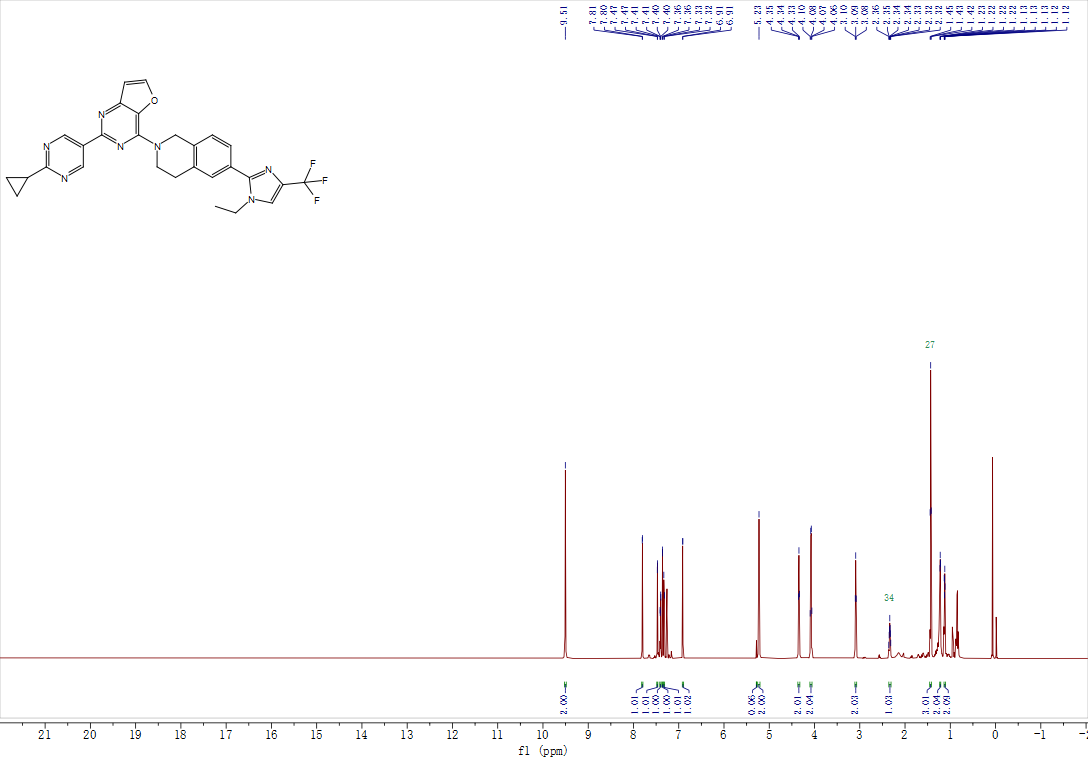
**

1H NMR of compound **14h**

**
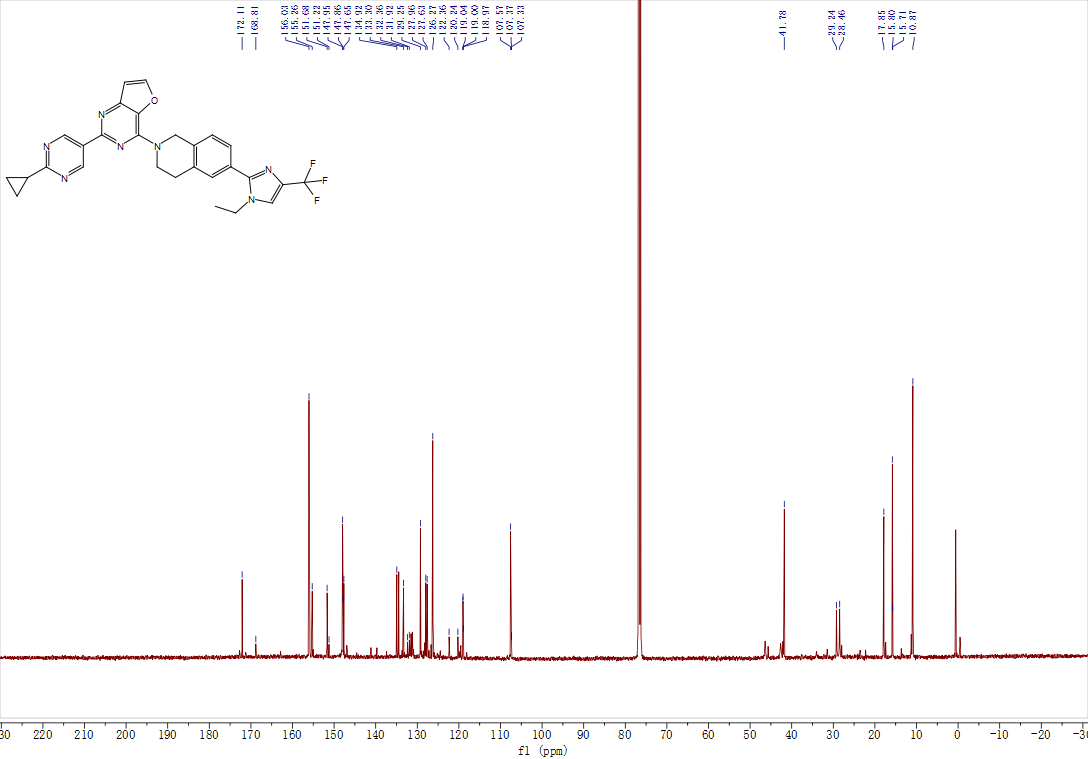
**

13C NMR of compound **14h**

**
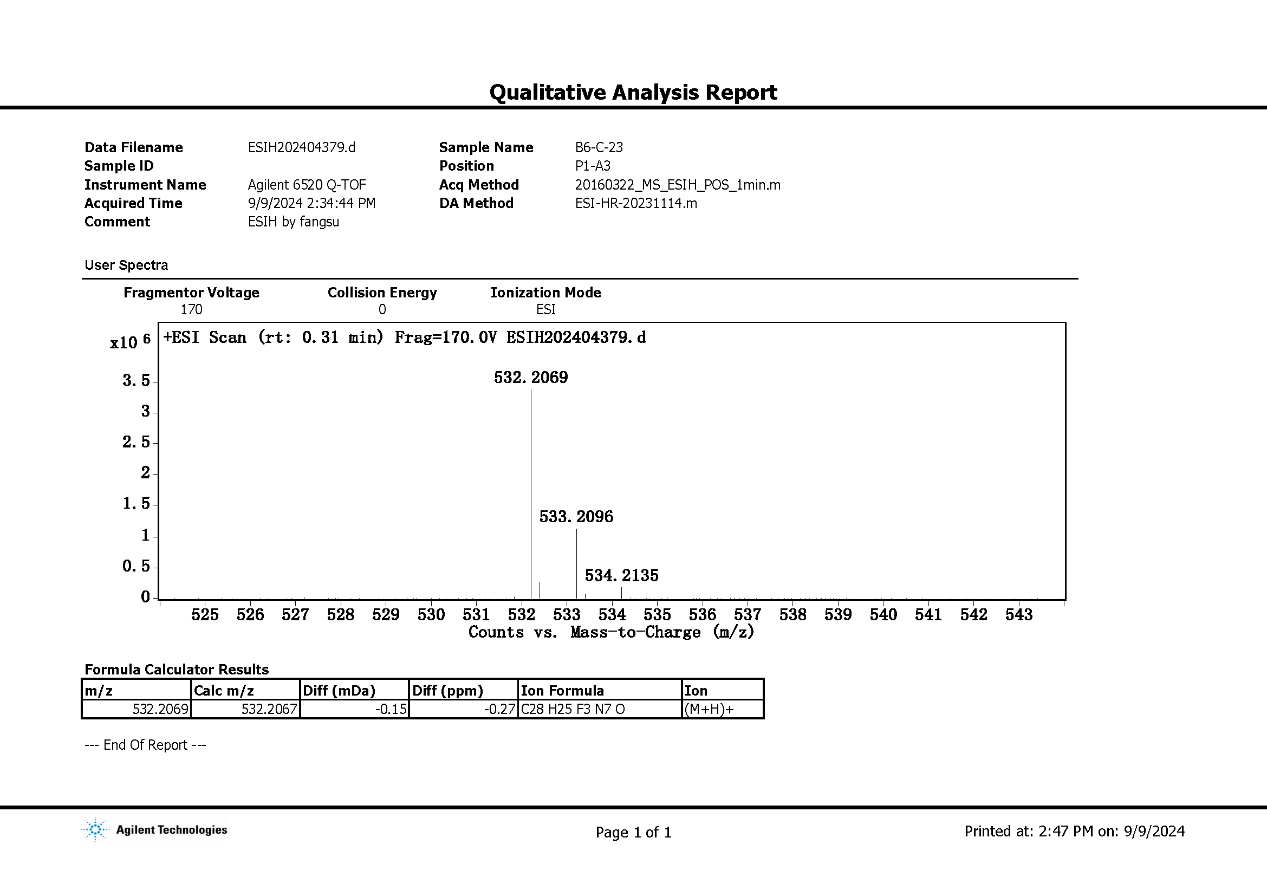
**

HRMS (ESI) of compound **14h**

**
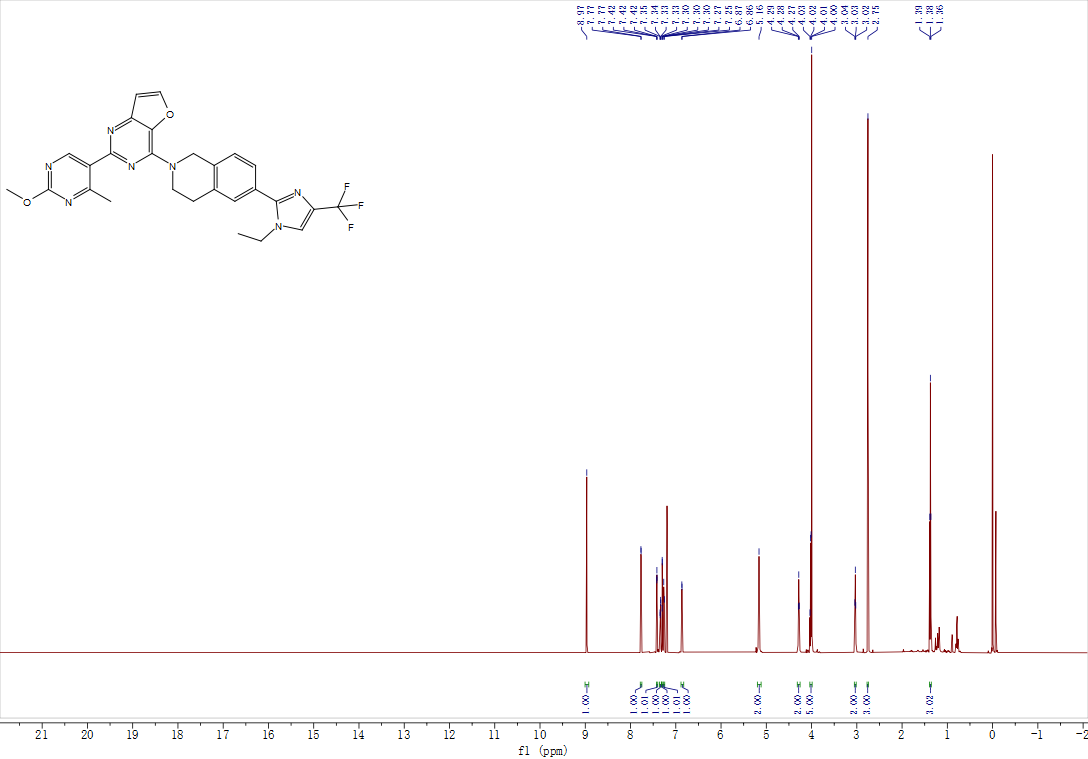
**

1H NMR of compound **14i
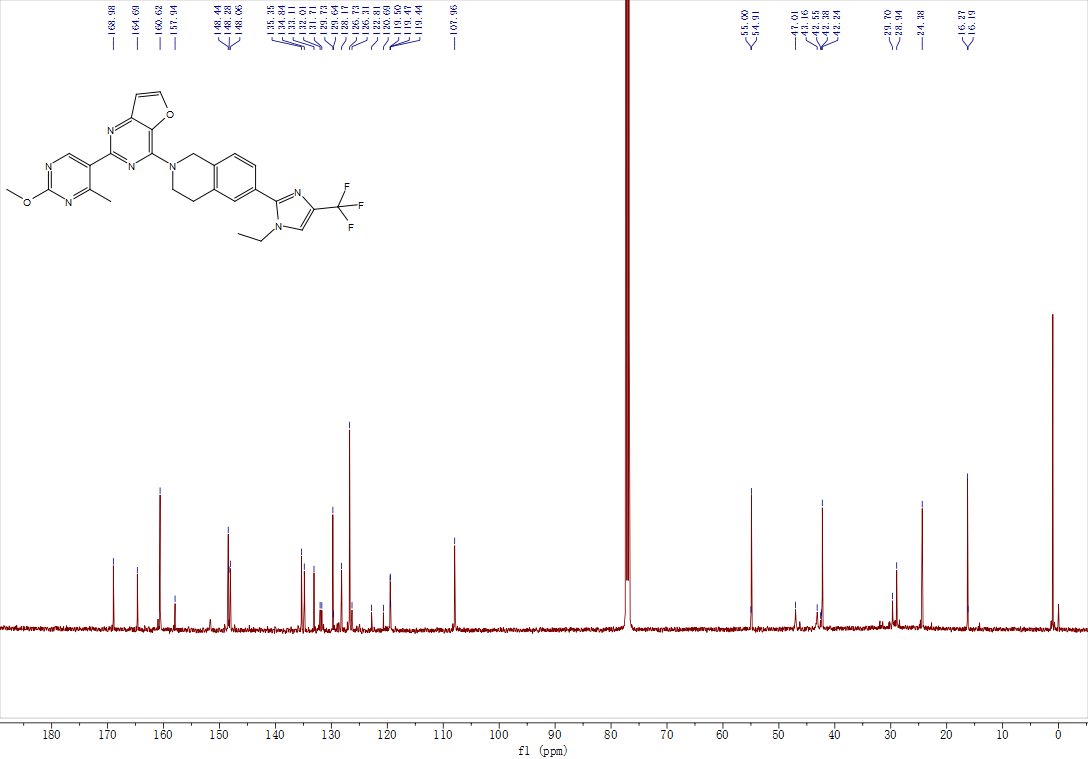
**

13C NMR of compound **14i**

**
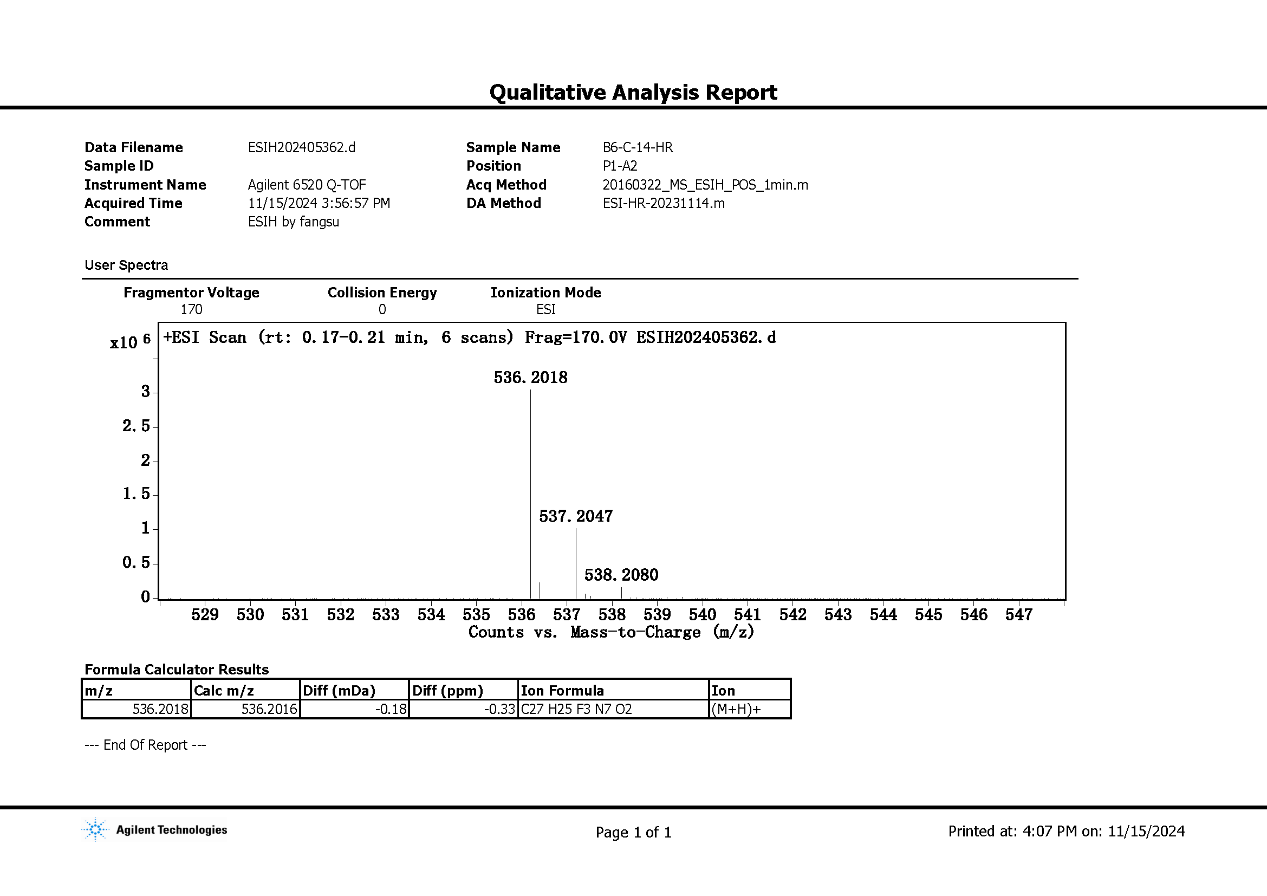
**

HRMS (ESI) of compound **14i**

**
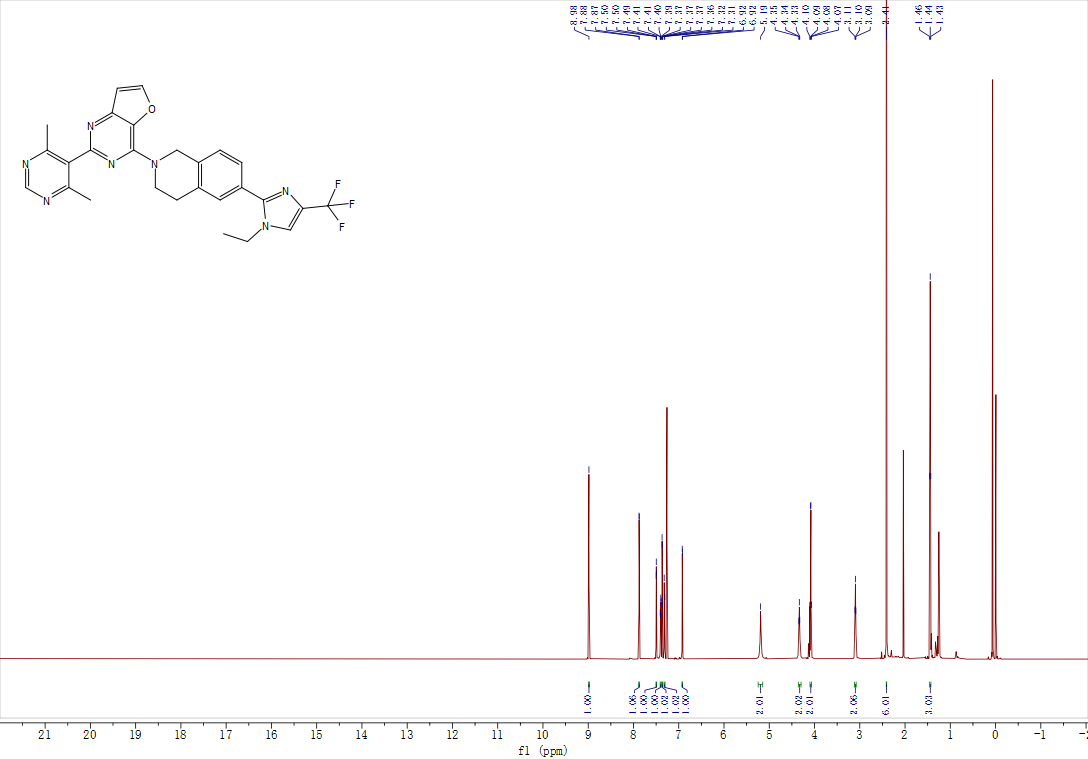
**

1H NMR of compound **14j
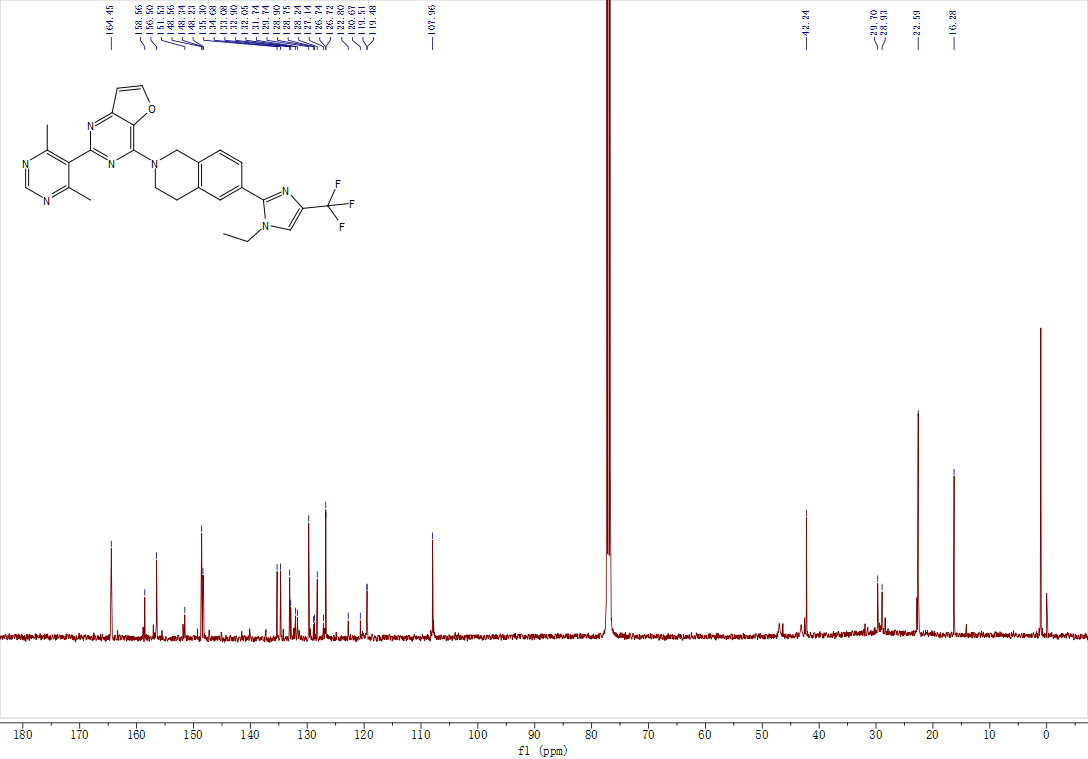
**

13C NMR of compound **14j**

**
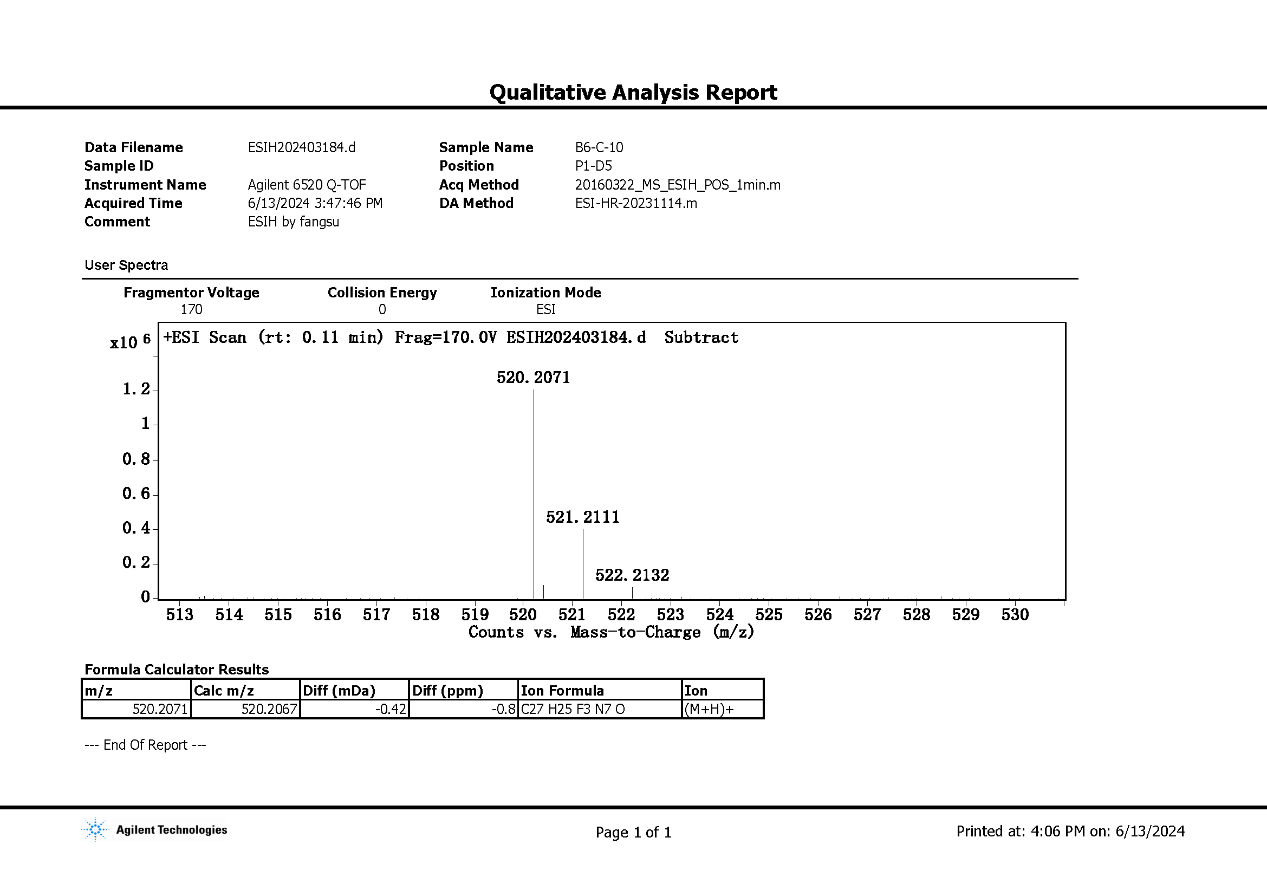
**

HRMS (ESI) of compound **14j**

**
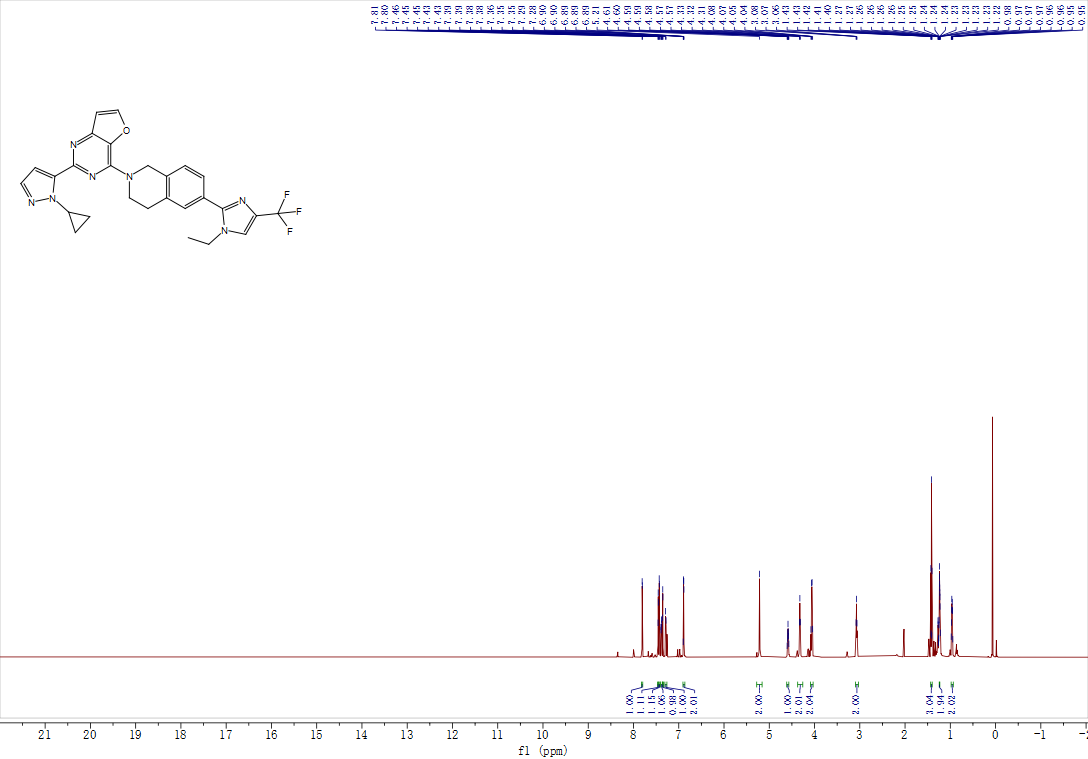
**

1H NMR of compound **14k
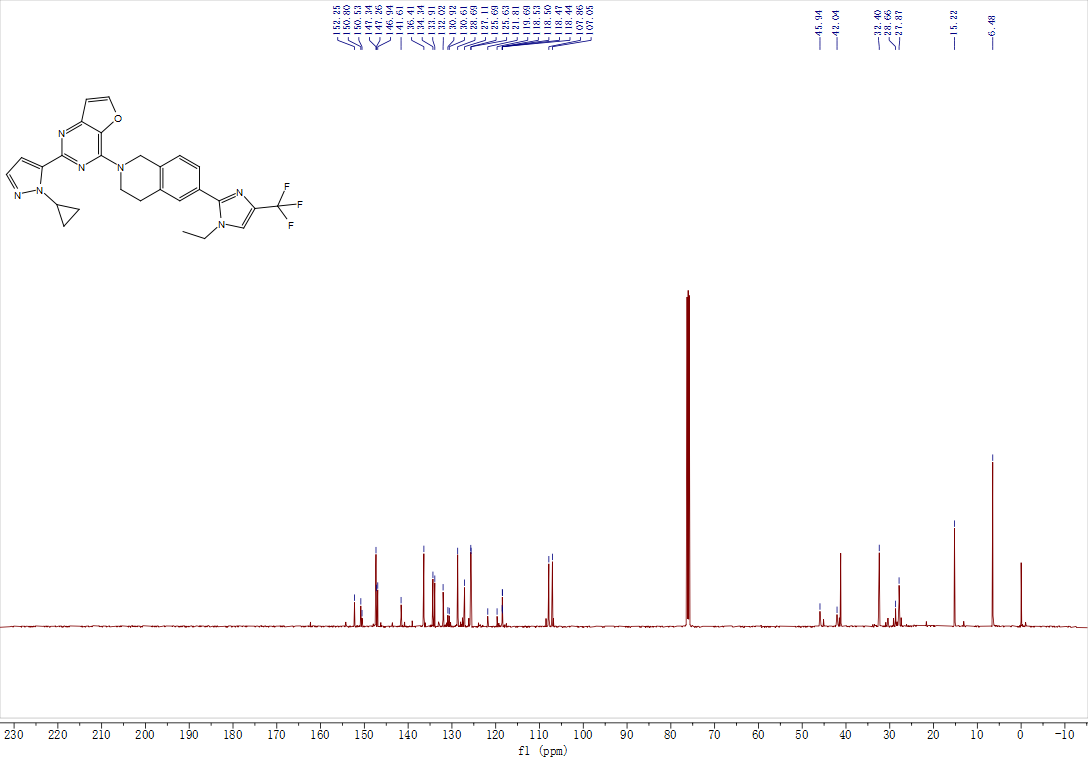
**

13C NMR of compound **14k**

**
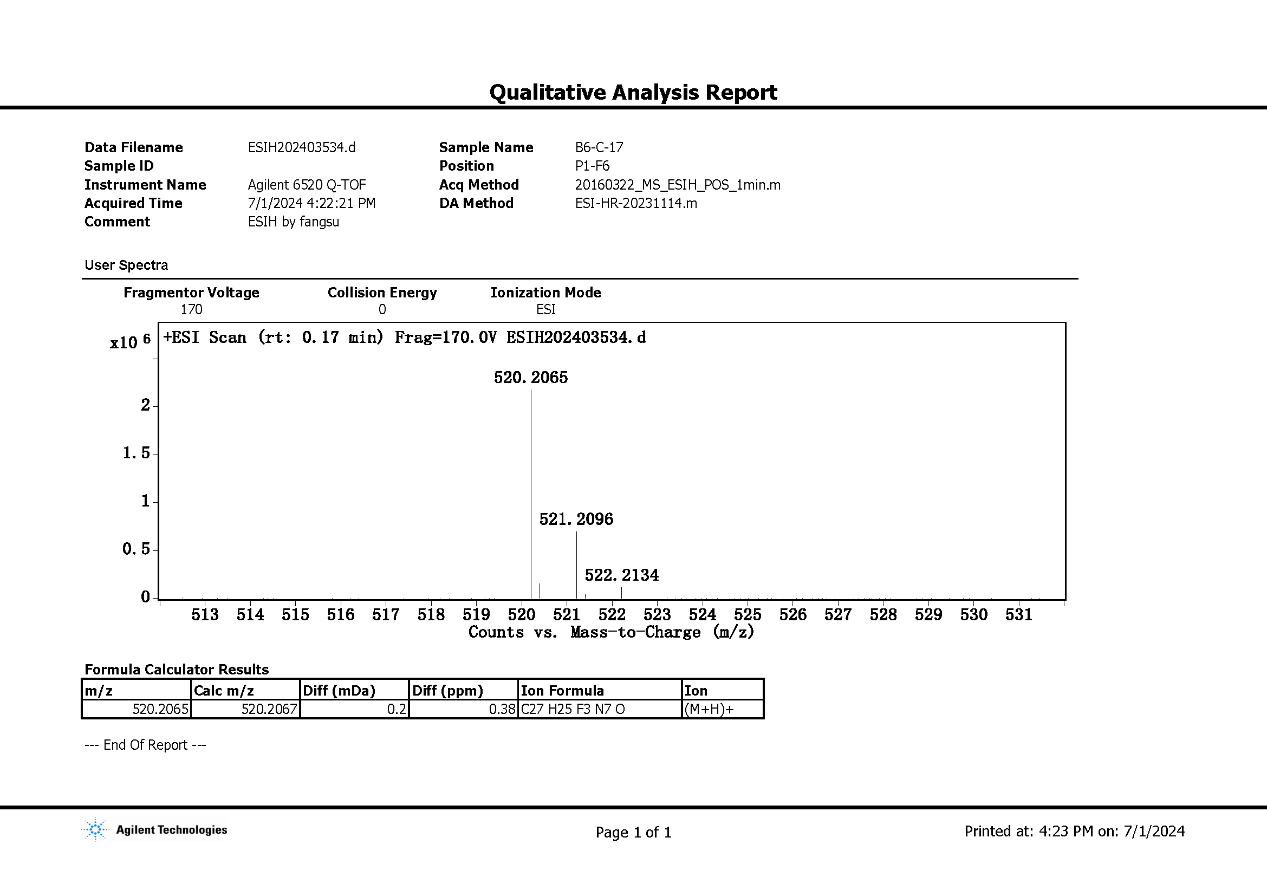
**

HRMS (ESI) of compound **14k**

**
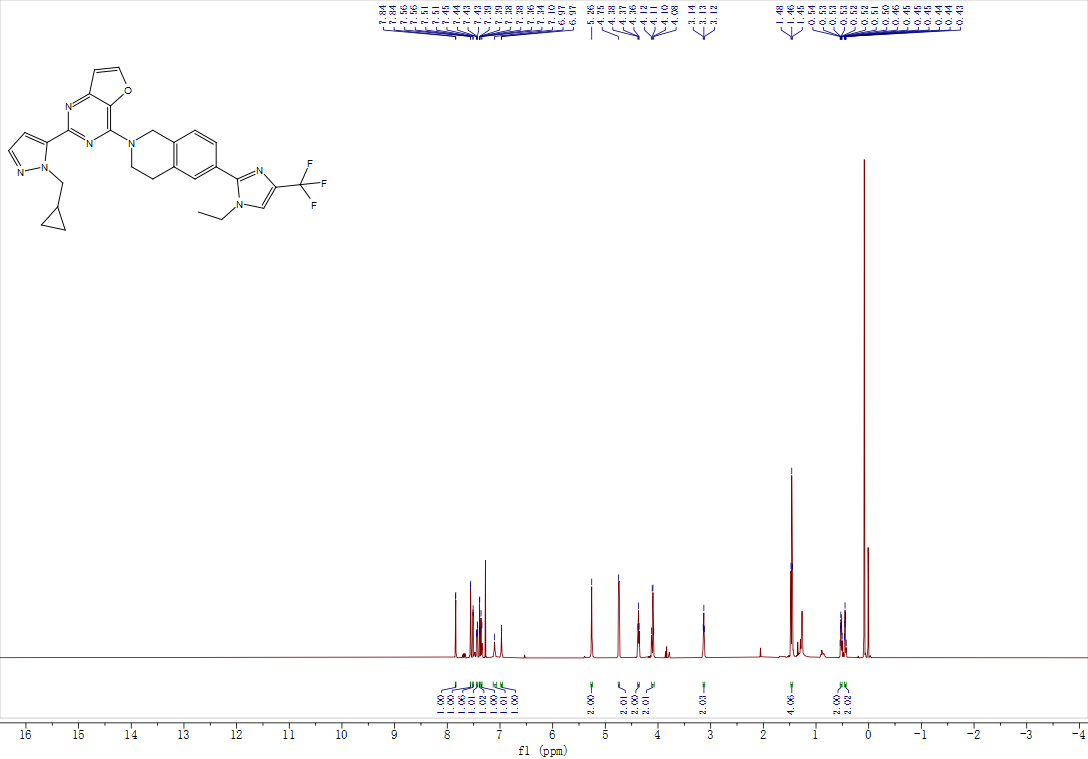
**

1H NMR of compound **14l
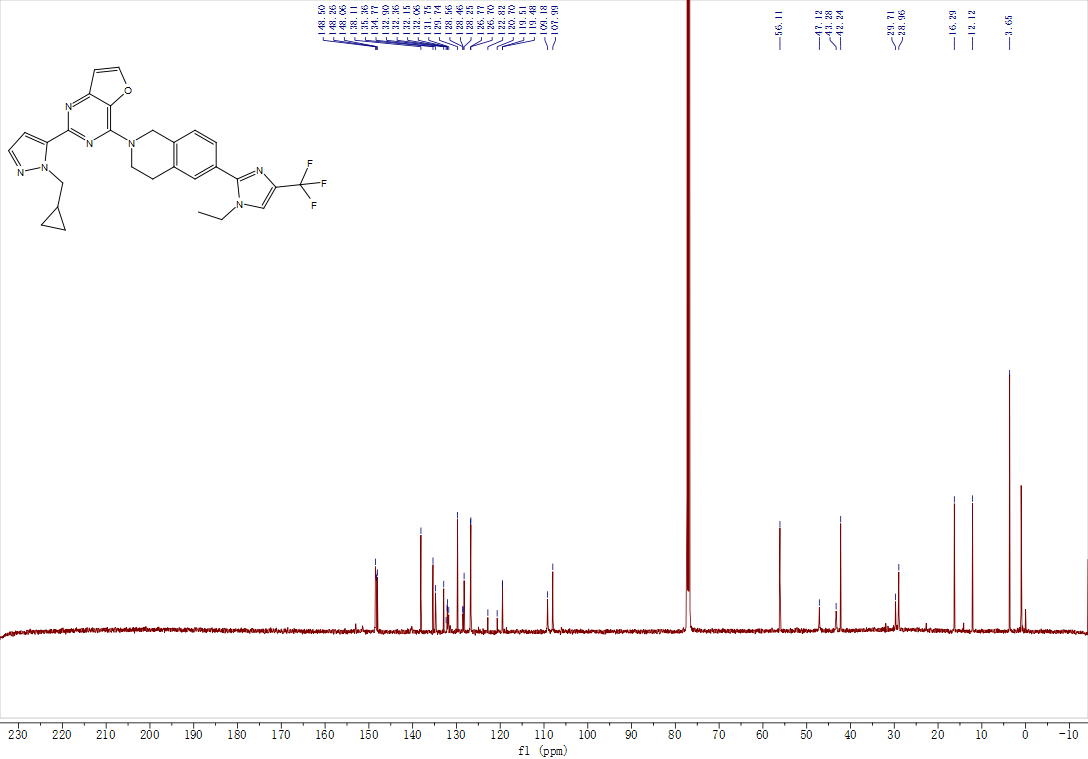
**

13C NMR of compound **14l**

**
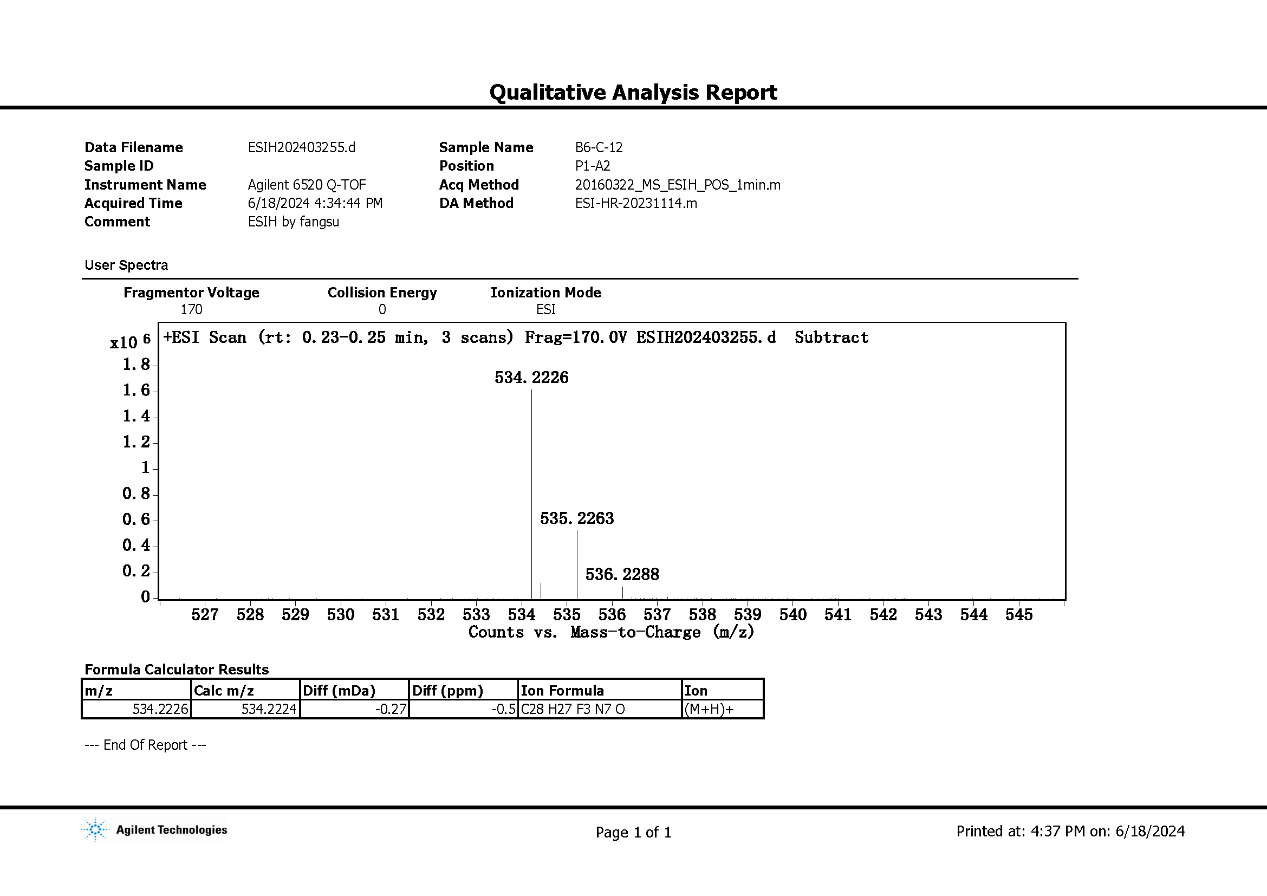
**

HRMS (ESI) of compound **14l**


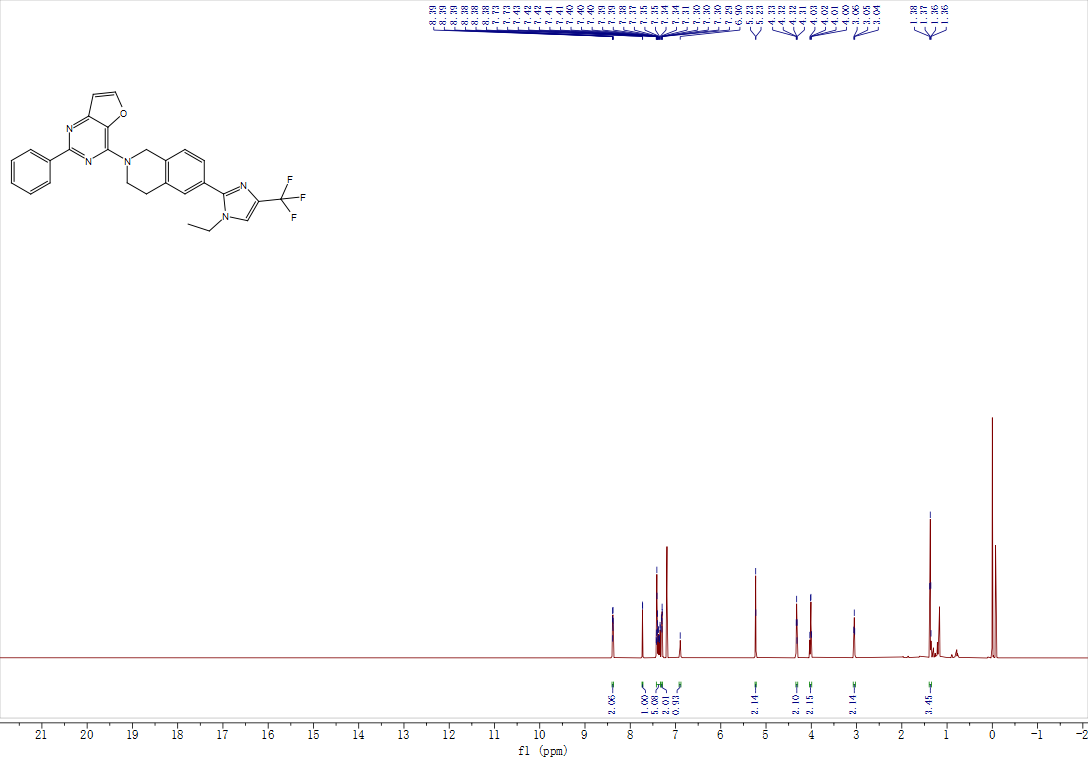


1H NMR of compound **14m**


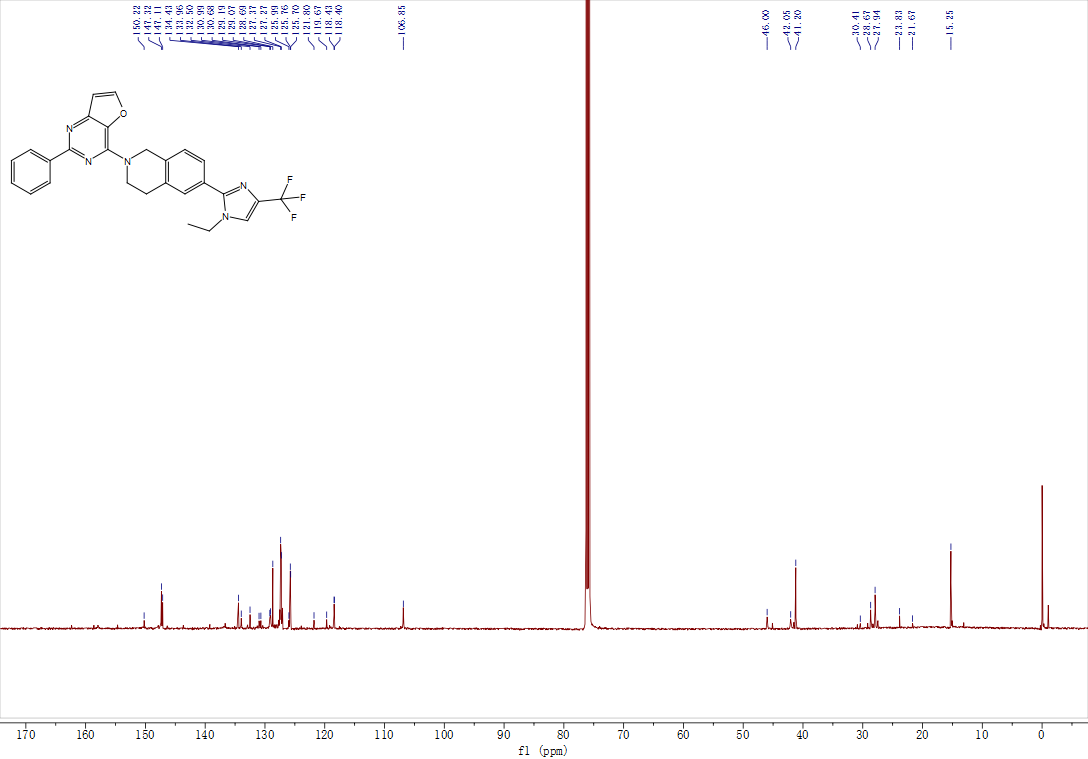


13C NMR of compound **14m**

HRMS (ESI) of compound **14m**

1H NMR of compound **14n**

13C NMR of compound **14n**

HRMS (ESI) of compound **14n**

1H NMR of compound **14o**

13C NMR of compound **14o**

HRMS (ESI) of compound **14o**

1H NMR of compound **14p**

13C NMR of compound **14p**

HRMS (ESI) of compound **14p**

1H NMR of compound **14q**

13C NMR of compound **14q**

HRMS (ESI) of compound **14q**

1H NMR of compound **14r**

13C NMR of compound **14r**

HRMS (ESI) of compound **14r**

1H NMR of compound **14s**

13C NMR of compound **14s**

HRMS (ESI) of compound **14s**

1H NMR of compound **14t**

13C NMR of compound **14t**

HRMS (ESI) of compound **14t**

1H NMR of compound **14u**

13C NMR of compound **14u**

HRMS (ESI) of compound **14u**

1H NMR of compound **14v**

13C NMR of compound **14v**

HRMS (ESI) of compound **14v**

1H NMR of compound **14w**

13C NMR of compound **14w**

HRMS (ESI) of compound **14w**

1H NMR of compound **14y**

13C NMR of compound **14y**

HRMS (ESI) of compound **14y**

1H NMR of compound **15**

HRMS (ESI) of compound **15**

1H NMR of probe **6-1**

13C NMR of probe **6-1**

HRMS (ESI) of probe **6-1**

1H NMR of probe **6-2**

13C NMR of probe **6-2**

HRMS (ESI) of probe **6-2**

1H NMR of probe **6-3**

13C NMR of probe **6-3**

HRMS (ESI) of probe **6-3**

**HPLC traces of target compounds**

Compound **13a**

Compound **13b**

Compound **13c**

Compound **13d**

Compound **13e**

Compound **13f**

Compound **13g**

Compound **13h**

Compound **13i**

Compound **13j**

Compound **13k**

Compound **13l**

Compound **13m**

Compound **13n**

Compound **13o**

Compound **13p**

Compound **14a**

Compound **14b**

Compound **14c**

Compound **14d**

Compound **14e**

Compound **14f**

Compound **14g**

Compound **14h**

Compound **14i**

Compound **14j**

Compound **14k**

Compound **14l**

Compound **14m**

Compound **14n**

Compound **14o**

Compound **14p**

Compound **14q**

Compound **14r**

Compound **14s**

Compound **14t**

Compound **14u**

Compound **14v**

Compound **14w**

Compound **14y**

Probe **6-1**

Probe **6-2**

Probe **6-3**
